# Synthese von α‐Arylacrylamiden via Lewis Base vermitteltem Aryl/Wasserstoff‐Austausch

**DOI:** 10.1002/ange.202207475

**Published:** 2022-08-29

**Authors:** Miran Lemmerer, Haoqi Zhang, Anthony J. Fernandes, Tobias Fischer, Marianne Mießkes, Yi Xiao, Nuno Maulide

**Affiliations:** ^1^ Fakultät Chemie, Institut für Organische Chemie Universität Wien Währinger Str. 38 1090 Wien Österreich; ^2^ Christian-Doppler Laboratory for Entropy-Oriented Drug Design Josef-Holaubek-Platz 1090 Wien Österreich; ^3^ Boehringer-Ingelheim RCV 1120 Wien Österreich; ^4^ CeMM Research Center for Molecular Medicine of the Austrian Academy of Sciences Lazarettgasse 14, AKH BT 25.3 1090 Wien Österreich

**Keywords:** Amide, Aromatische Substitution, C−C Bindungsbildung, Organokatalysator, Umlagerung

## Abstract

Hierin stellen wir eine neue Methode für die Synthese von α‐Arylacrylamiden vor. Die Reaktion basiert auf der Nutzung polarer *S*‐zu*‐C* Arylwanderungen, induziert durch einen Lewis‐basischen Organokatalysator. Im Unterschied zu zuvor publizierten radikalischen Arylwanderungen von Sulfonylacrylamiden, ermöglicht dieser polare Prozess eine darauffolgende Eliminierung, wodurch in Summe ein formaler Aryl/Wasserstoff‐Austausch unter Ausscheidung von SO_2_ stattfindet. Die vorgestellte Reaktion ist selektiv für elektronenarme aromatische Gruppen, während eine Vielfalt von Substituenten am Stickstoff und an der β‐Position toleriert werden, und erzeugt nützliche Bausteine für Folgereaktionen wie Zykloadditionen und Zyklisierungen. Der Reaktionsmechanismus wurde mithilfe quantenchemischer Berechnungen erforscht, die die unerwartete Rolle der Lewis Base in mehreren Schlüsselschritten darlegten.

## Graphical abstract

Eine DABCO‐induzierte, polare Arylwanderung erlaubt die Synthese von α‐Arylacrylamiden aus Sulfonylacrylimiden. Die Reaktion verläuft über einem formalen Aryl/Wasserstoff‐Austausch, einschließlich SO_2_‐Verlustes. Eine Reihe von wertvollen Bausteinen wurde synthetisiert und der zugrunde liegende Reaktionsmechanismus mithilfe quantenchemischer Berechnungen untersucht.

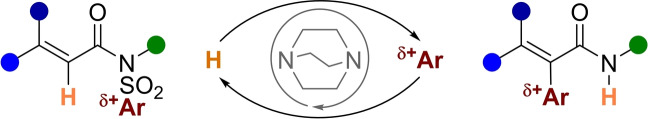

α,β‐Ungesättigte α‐Arylcarboxamide sind nützliche Bausteine in der organischen Synthese. Insbesondere bieten elektronarme Alkene vielfältige Möglichkeiten zur Inkorporation in Strukturen mit unterschiedlichen Anwendungen (Schema 1A).^[1]^ Interessanterweise, mit Blick auf die Synthese solcher Strukturen, erzeugen übergangsmetallkatalysierte Reaktionen üblicherweise die “falschen” Regioisomere. Tatsächlich ist die formale α‐C−H‐Arylierung von sekundären Acrylamiden bisher nicht möglich, da die übliche Heck‐Chemie zu β‐Arylierung führt (Schema 1B).^[2]^ Kreuzkupplungen hingegen benötigen entweder eine α‐Vorfunktionalisierung mit Halogenen (Schema 1C)^[1e]^ oder eine Vorinstallation dirigierender Gruppen.^[3]^ In der Vergangenheit stellte sich Arylwanderung als ein geeignetes Werkzeug für die Synthese von *gesättigten* α‐Arylamiden heraus.[^4^, ^5^] Die Nevado Gruppe wendete solch eine Strategie in einer Serie von Publikationen an *N*‐Aryl *n*‐Sulfonyl Methacrylimiden an (Schema 1D).^[6]^ Dabei generiert eine Radikaladdition ein α‐Radikal, das an eine Arylwanderung auslöst (Truce‐Smiles Umlagerung),^[7]^ die schlussendlich unter SO_2_‐Abspaltung zu einem *N‐*zentrierten Radikal führt. Dieselbe Arbeitsgruppe publizierte kürzlich eine enantiospezifische Variante dieser Reaktion,^[8]^ während andere Gruppen die Reaktionsmöglichkeiten mit einer Vielfalt an Radikalen erweiterten.[^9^, ^10^] Die Stärke der meisten publizierten Methoden liegt in der Wanderung elektronreicher und neutraler Arylgruppen, während es an Beispielen von Umlagerung elektronarmer Aryle mangelt. Zusätzlich scheint meist eine α‐Alkylgruppe nötig zu sein, da die bisher bekannten Transformationen nur mit einer Handvoll an α‐unsubstituierten Arylimiden bekannt sind.[^9b^, ^9h^, ^10^]

**Scheme 1:**
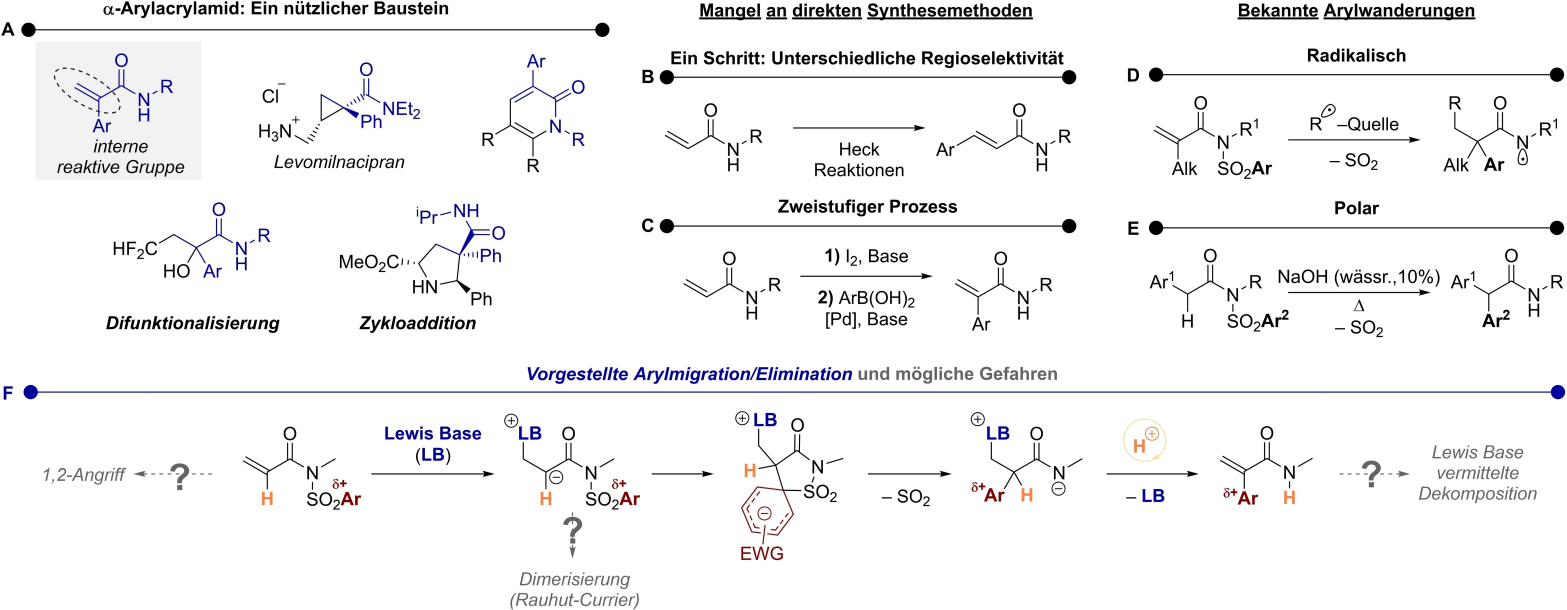
A) Beispiele für α‐Arylacrylamide, die für Zykloadditionen and Difunktionalisierungen benutzt wurden. B) Heck Reaktionen führen zu β‐Arylierung. C) Formale C−H‐α‐Arylierung benötigt einen zweistufigen Prozess. D) Berichte von α‐Arylierungen über radikalischer Wanderung, die gesättigte Amide liefern. E) Eine polare Arylwanderung von Arylacetylsulfonylimiden. F) Unsere Lewis‐Base‐induzierte Arylwanderung, gefolgt von Eliminierung. Mögliche Schwierigkeiten und Nebenreaktionen sind in grau angezeigt.

Inspiriert von den Werken von Dohmori^[11]^ und Greaney (Schema 1E),^[12]^ spekulierten wir, dass eine *anionische* Arylmigration die Synthese von α‐Arylacrylamiden erlauben würde (Schema 1F). Konkret nahmen wir an, dass eine Lewis‐Base eine 1,4‐Addition, ähnlich der Morita–Baylis–Hillman‐ oder Rauhut–Currier‐Reaktionen,[^13^, ^14^] initiieren könnte. Das so generierte Enolat könnte dann eine polare Arylwanderung via Meisenheimer‐Intermediat unter gleichzeitiger Freisetzung von SO_2_ zur Folge haben. Unserer Annahme nach könnte das daraus resultierende Amidanion an der α‐Position deprotonieren und dadurch, unter Eliminierung der Lewis Base, das gewünschte Alken freisetzen.

Dieser Plan war jedoch nicht ohne potentielle Tücken: Zum Beispiel besteht die Möglichkeit einer konkurrierenden Rauhut‐Currier Dimerisierung oder Amiddeacylierung in ähnlicher Reaktionsgeschwindigkeit wie die 1,4‐Addition, was zur möglichen Freisetzung von Sulfonamid in Lösung führen könnte und somit eine unerwünschte Nebenreaktion wäre. Es war ebenfalls unklar ob das Produkt, welches noch immer ein guter Michael‐Akzeptor ist, eher von der Lewis‐Base angegriffen wird als das Substrat, wodurch weitere unerwünschte Produkte entstehen könnten.

Wir starteten unsere Suche nach optimalen Reaktionsbedingungen mit *N‐*Acryl‐*N*‐sulfonylimid **1 a**, das eine elektronarme *p*‐Nitrophenylgruppe trägt, in Kombination mit unterschiedlichen Lewis Basen. Die Experimente zeigten, dass 1,4‐Diazabicyclo[2.2.2]octan (DABCO) die höchste Ausbeute an α‐Arylacrylamid **2 a** erbrachte (Tabelle 1, Eintrag 1, siehe Hintergrundinformationen für weitere Details). Die signifikantesten Nebenprodukte sind, wie erwartet, Dimer **1‐rc** (als Produkt einer Rauhut–Currier Reaktion) und ein 1,4‐Additionsprodukt **1‐sa** (es ist anzunehmen, dass DABCO‐vermittelte Deacylierung in situ Sulfonamid generiert, welches an das Startmaterial addiert). Weder die Addition von Alkoholen zur Mischung, um einen potentiellen Protontransfer zu erleichtern, noch der Wechsel zu anderen Lösungsmitteln erhöhten die Ausbeute von **2 a** (Einträge 2–4). Um das Ausmaß der Rauhut–Currier Nebenreaktion zu reduzieren, verringerten wir die Konzentration, was einen signifikanten positiven Einfluss auf die Reaktion hatte (Einträge 5, 6). Ermutigt durch die hohe Ausbeute unter verdünnten Bedingungen und nach längerer Reaktionszeit, testeten wir die Möglichkeit, die DABCO Menge zu reduzieren, da in Prinzip nur eine katalytische Menge nötig wäre (siehe Schema 1F). Im Falle einer substöchiometrischen Menge erzielten wir eine Ausbeute von 80 % bei 80 °C, was eine quasi‐katalytische Reaktion darstellt.^[15]^ Die Notwendigkeit an größerer Menge von DABCO könnte durch die Bildung eines DABCO‐(SO_2_)_2_ Addukts, auch bekannt als DABSO,^[16]^ erklärt werden, welches wir auch als Niederschlag während der Reaktion beobachten konnten.

**Table 1:** Optimierung von Reaktionsbedingungen.

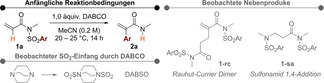				
Eintrag	Abweichung der Bedingungen	**2 a^[a]^**	**1‐rc^[a]^**	**1‐sa^[a]^**
1	keine	40 %	5 %	4 %
2	Additiv: 1.0 äquiv. BnOH	30 %	3 %	2 %
3	Lösungsmittel: MeCN:^t^BuOH 9 : 1	30 %	3 %	2 %
4	Lösungsmittel: DMF	20 %	<1 %	n.d.^[b]^
5	0.05 M	63 %	1 %	n.d.^[b]^
6	**0.05 M; 72 h**	**94 %**	**3 %**	**2 %**

Ar=*p‐*NO_2_−C_6_H_4_. [a] Die Ausbeute wurde über ^1^H NMR Spektroskopie mithilfe von Mesitylen als internem Standard bestimmt. [b] Nicht bestimmt aufgrund von Signalüberlagerung.

Für die folgende Investigation des Reaktionsumfanges, entschieden wir uns ein Raumtemperaturverfahren einzusetzen (Schema 2). Die sterische Voraussetzung von *N*‐Substituenten wurde mit *iso*‐Propyl‐ (**2 b**) und *tert*‐Butyl‐ (**2 c**) Gruppen getestet, welche zu leicht verringerten Ausbeuten führten. Allyl‐ (**2 d**) und Benzyl‐ (**2 e**) Substituenten, sowie Acetalgruppen (**2 f**) wurden unter den Reaktionsbedingungen toleriert. Mehrere Derivate von bioaktiven Verbindungen, wie Aminoester (**2 g** und **2 h**), sowie Dopamin (**2 i**) und Tryptamin (**2 j**), konnten hergestellt werden, was die breite Anwendungsmöglichkeit der Methode unterstreicht. Diese Beispiele gaben uns auch Informationen über die Toleranz von funktionellen Gruppen wie Ester (**2 g**), Sulfid (**2 h**) und Carbamat (**2 j**), die allesamt unberührt blieben. Ein angebundener α,β‐ungesättigter Ester störte auch bei 80 °C nicht, und lieferte Amid **2 k** mit 81 % Ausbeute.

**Scheme 2:**
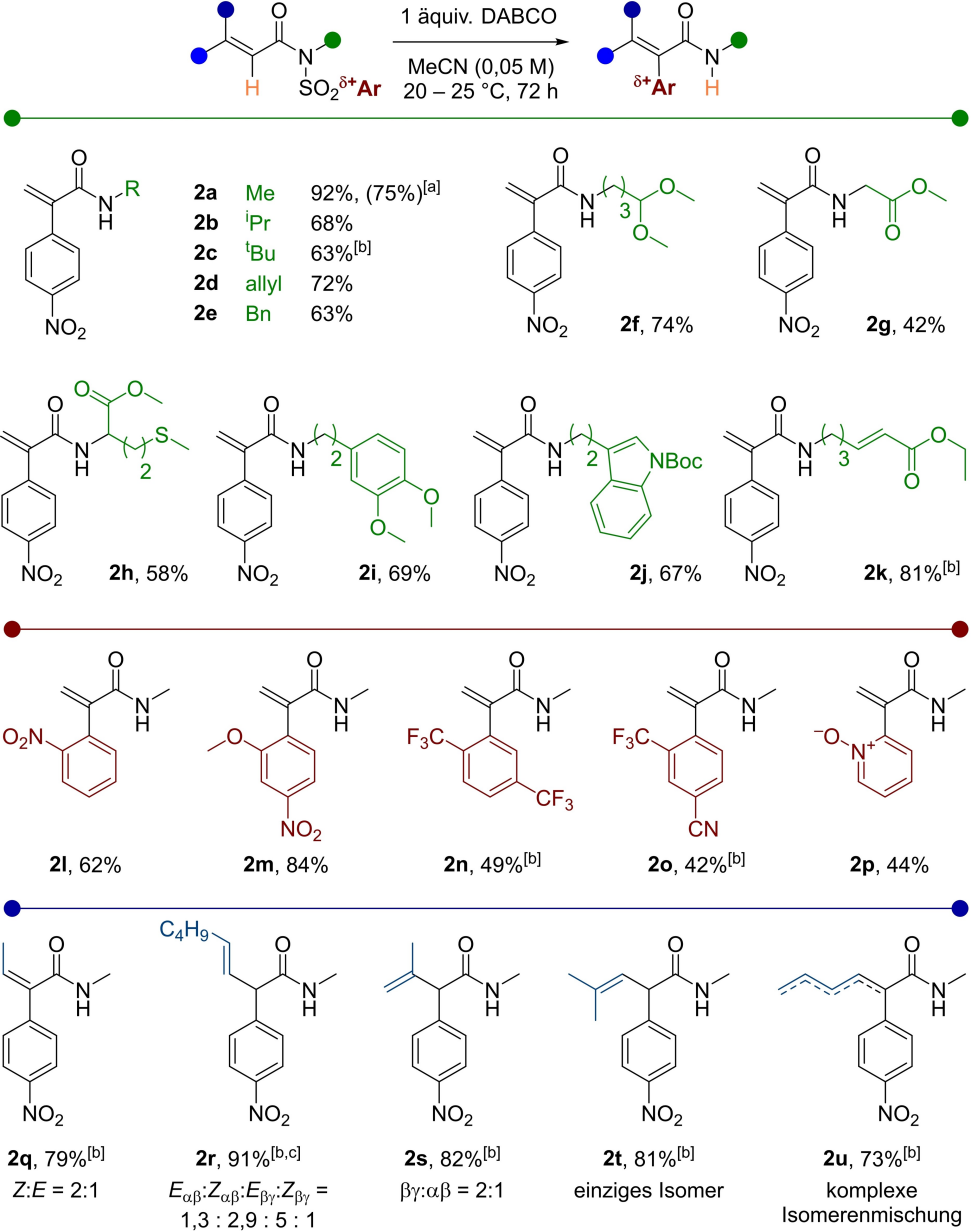
Der Reaktionsumfang in Bezug auf unterschiedliche Substituenten (0.2 mmol Ansatz). In Falle von mehreren Alkenisomeren ist das Hauptprodukt gezeigt und das Verhältnis darunter angegeben. [a] 1 mmol Ansatz. [b] Bei 80 °C durchgeführt. [c] 120 h Reaktionszeit.

Die Reaktion ist spezifisch für elektronarme *N*‐Aryl‐Sulfonamide, inklusive dem *ortho*‐Isomer von **2 a** (**2 l**). Der stark induktive Effekt der Nitrogruppe erlaubte den gleichzeitigen Einsatz der elektronschiebenden Methoxygruppe (**2 m**). Arene mit einer (**2 o**) oder zwei Trifluoromethygruppen (**2 n**), sowie Nitrile (**2 o**), sind auch geeignete Substrate. Zusätzlich konnte ein elektronenarmes Heteroaren ebenso eingesetzt werden – die entsprechende Umlagerung zum gewünschten 2‐Vinylpyridin‐*N*‐oxid (**2 p**) funktionierte mit moderater Ausbeute.

Die Methode ist nicht auf β‐unsubstituierte Acrylimide limitiert, jedoch benötigten β‐alkylsubstituierte Substrate höhere Temperaturen für die Umlagerung. Sowohl β‐Methyl‐ (**2 q**) als auch β‐*n*‐Pentyl‐ (**2 r**) Substrate führten mit hohen Ausbeuten zu den entsprechenden Produkten. Interessanterweise bildet das letztere Substrat bevorzugt ein β,γ‐ungesättigtes, und somit ein nicht konjugiertes, Amid. Diese Selektivität wurde auch bei β,β‐Dimethylacrylamid **2 s** und noch deutlicher bei **2 t** beobachtet. Abschließend formte ein α,β,γ,δ‐konjugiertes Dien eine Mischung von Aryl‐umgelagerten Isomeren **2 u** mit guter Gesamtausbeute. Siehe Hintergrundinformationen für einen weitere Reaktionen, die die Limitierungen der Methode aufzeigen.

Fasziniert von der Eliminierungsselektivität **2 r**, führten wir die Reaktion mit einer starken, nicht nukleophilen Guanidinbase (Bartonsche Base) anstelle von DABCO durch. Wie aus Schema 3A ersichtlich, lieferte die Brønsted Base das interne Alkenisomer ***iso*** **‐2 r**, jedoch in geringerer Ausbeute.

**Scheme 3:**
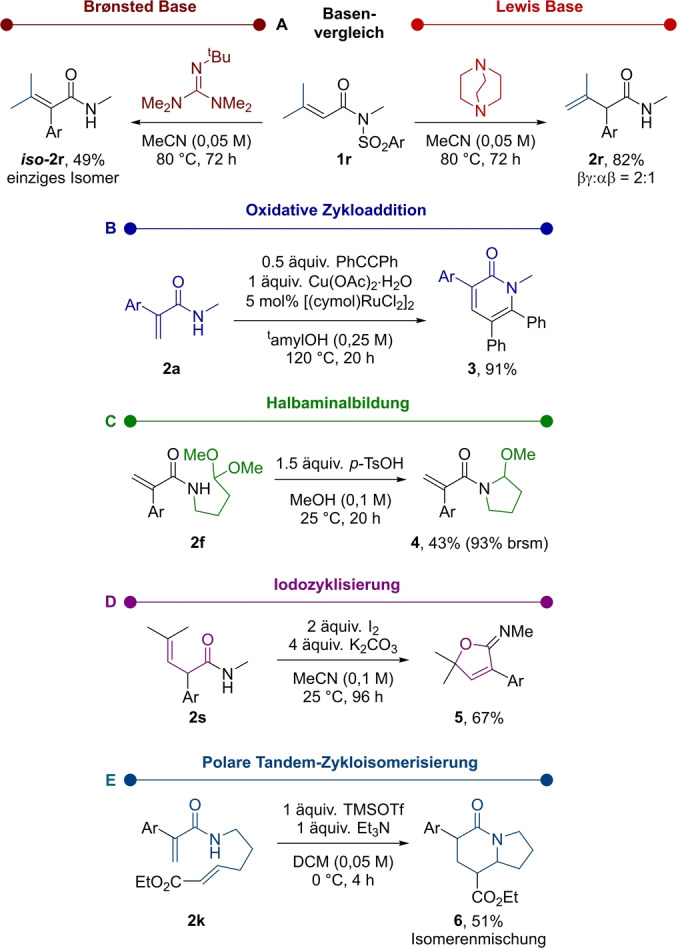
Ar=*p*NO_2_‐C_6_H_4_. A) Selektivitätsvergleich zwischen einer Brønsted Base und einer Lewis Base. B–E) Weiterführende Reaktionen unter Anwendung der neu synthetisierten α‐Arylamide.

Experimente, die die synthetische Nützlichkeit der geformten α‐Arylacrylamide zeigen, sind in Schema 3B–E angeführt. Die Ruthenium‐katalysierte, oxidative Zykloaddition von Acrylamid **2 a** und Diphenylacetylen, unter den berichteten Reaktionsbedingungen von Ackermann, führten mit hoher Ausbeute zu Pyridon **3**.^[1d]^ Zusätzlich verlief eine säurekatalysierte Halbaminalbildung reibungslos,^[17]^ und eine Iodozyklisierungs/Eliminierungssequenz von β,γ‐ungesättigtem Amid **2 s** führte zu Imidat **5** mit guter Ausbeute.

Schließlich lieferte eine polare Tandem‐Zykloisomerisierung, ausgehend vom Bisacrylprodukt **2 k**, via einer aza‐1,4‐Addition, gefolgt von einer Michael‐Addition, Aryl‐2‐oxo‐octahydroindolizin **6**, was die Möglichkeit des rapiden Anstiegs an Verbindungskomplexität, ausgehend α‐Arylacrylamiden, illustriert.^[18]^

Um den Mechanismus näher zu erforschen, wurden quantenchemische Berechnungen auf dem dichtefunktional‐theoretischen (DFT) Level durchgeführt (Abbildung 1, siehe Hintergrundinformationen für nähere Details). Das erhaltene Energieprofil geht von separaten Reagenzien, DABCO und Substrat **1 a**, aus, die den Reaktionskomplex **A** bilden. Der erste Schritt ist eine kinetisch begünstigte, leicht endergonische aza‐Michael‐Addition, die zum Betainintermediat **B** (Δ*G* ^≠^(**1 a→B**)=13.2 kcal mol^−1^) führt. Der nachfolgende Schritt ist eine Truce‐Smiles‐Umlagerung(Schritt **B→C**), die über einen frühen Meisenheimer‐Übergangszustand **TS_BC_** zu einer konzertierten, asynchronen C−C Bindungsbildung mit gleichzeitigem C−S Bindungsbruch führt.^[19]^ Dieser Schritt hat eine nur 0.8 kcal mol^−1^ höhere Barriere als die rückläufige Michael‐Addition (Schritt **B→A**) aber die hohe Exergonität der Truce‐Smiles‐Umlagerung (Δ*G*(**B→C**)=−31.1 kcal mol^−1^) lenkt die Reaktion irreversibel in Richtung der Bildung des Intermediats **C**. Im Gegensatz zum anfänglichen Reaktionsdesign ist eine direkte SO_2_‐Freisetzung oder ein SO_2_‐Transferprozess von **C** energetisch verboten.^[20]^ Wir entdeckten jedoch, dass die Enolisierung von **C** durch die Beteiligung eines zweiten Moleküls DABCO möglich war, welches an der α‐Position des Carbonyls deprotonieren kann (Δ*G* ^≠^(**C→E**)=24.3 kcal mol^−1^) um Intermediat **E** zu bilden, wo die negative Ladung im aromatischen System stark delokalisiert ist (siehe Hintergrundinformationen). Die nachfolgende *E1cB* Eliminierung involviert die Spaltung der N−C‐Bindung und Freisetzung von DABCO (Δ*G* ^≠^(**D→E**)=8.2 kcal mol^−1^), wodurch Ammoniumsulfinat **F** entsteht, das nahezu isoenergetisch zu **E** ist. Der letzte Schritt ist ein exergoner, DABCO‐vermittelter SO_2_‐Transfer (Δ*G*(**F→G**)=−7.9 kcal mol^−1^) mit gleichzeitiger Protonierung des Stickstoffs und nukleophiler DABCO‐Substitution am Schwefel.^[21]^ Der Überganszustand für diesen Schritt (**TS_FG_**) liegt nur 0.7 kcal mol^−1^ über dem Überganszustand vom vorherigen Schritt (**TS_EF_**), wodurch die Produktbildung von der Exergonität des letzten Schrittes angetrieben ist. Der letzte, thermodynamisch begünstigte SO_2_‐Transfer generiert somit Produkt **2 a** über Protonierung, zusammen mit einer (DABCO)_2_⋅SO_2_‐Spezies, welche, wie in **G** dargestellt, durch Wasserstoffbindungen stabilisiert wird. Tatsächlich wurde solch ein Protontransfer experimentell durch Verwendung von Deuterium‐markiertem **1 a** gestützt (siehe Hintergrundinformationen für weitere Details). Des Weiteren konvergiert (DABCO)_2_⋅SO_2_ während der Reaktion schließlich zur thermodynamisch stabileren DABSO‐Spezies (Δ*G*(**(DABCO)_2_** ⋅**SO_2_→DABSO**)=−12.3 kcal mol^−1^), welche auch empirisch als Niederschlag beobachtet wurde.

**Figure 1:**
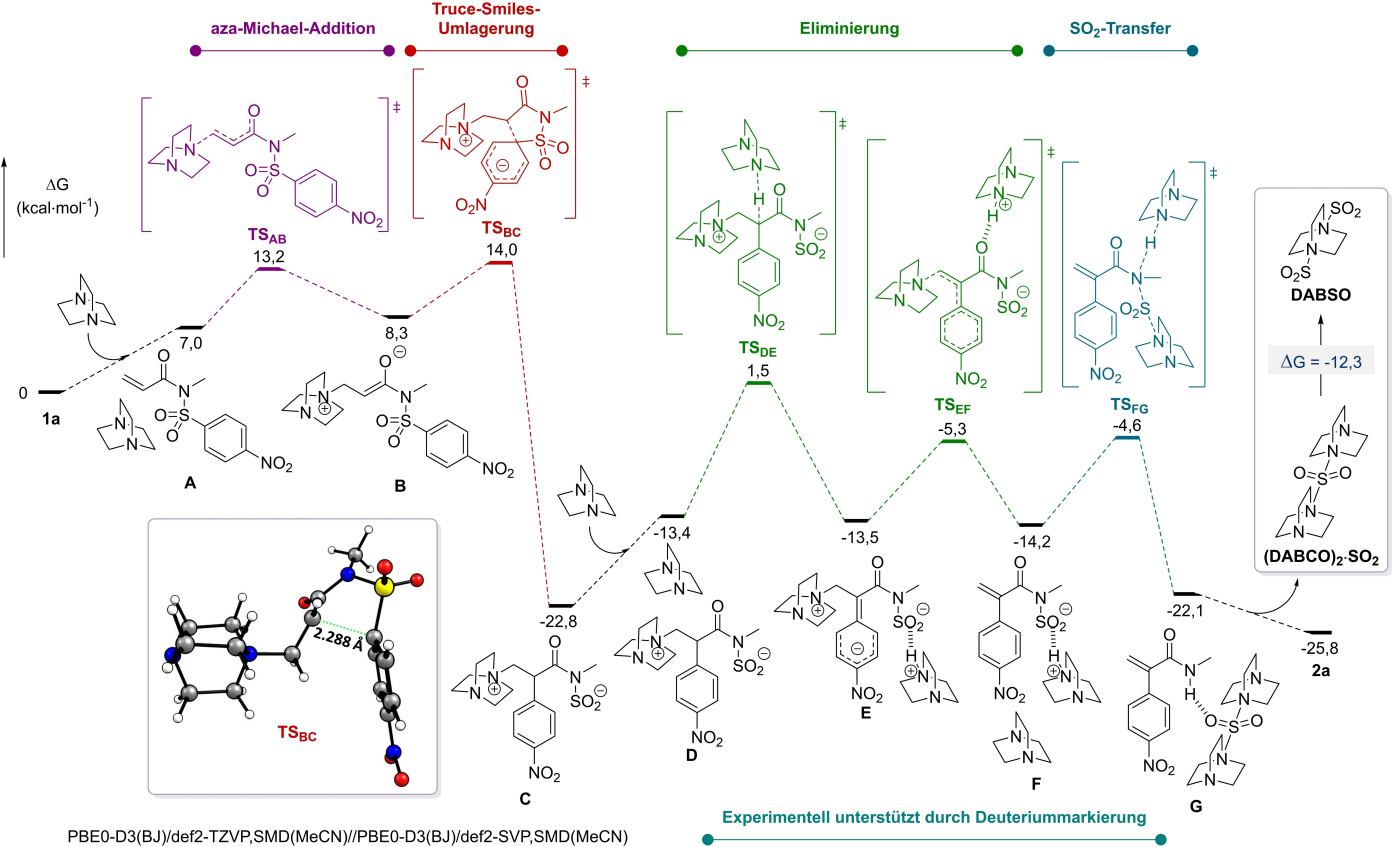
Berechnetes Reaktionsprofil (PBE0‐D3(BJ)/def2‐TZVP,SMD(MeCN)//PBE0‐D3(BJ)/def2‐SVP,SMD(MeCN)).

Hierin stellten wir eine neue Methode zur Synthese von α‐Arylamiden, basierend auf einer polaren Arylwanderung, vor. Die Lewis Base DABCO vermittelt den Prozess sogar bei Raumtemperatur, wodurch eine breite Palette an Substituenten und funktionellen Gruppen toleriert wird. Um die Nützlichkeit der Produkte zu demonstrieren, wurden die reaktive funktionelle Gruppen der Produkte für weitere Transformationen zu verschiedenen zyklischen Verbindungen verwendet. Abschließend lieferten quantenchemische Berechnungen Details über den zugrunde liegenden Mechanismus und offenbarten die Dreifachrolle von DABCO (1,4‐Addition; Protonen‐Shuttle; SO_2_ Transfer).

## Interessenkonflikt

Die Autoren erklären, dass keine Interessenkonflikte vorliegen.

## Supporting information

As a service to our authors and readers, this journal provides supporting information supplied by the authors. Such materials are peer reviewed and may be re‐organized for online delivery, but are not copy‐edited or typeset. Technical support issues arising from supporting information (other than missing files) should be addressed to the authors.

Supporting Information

## Data Availability

Die Daten, die die Ergebnisse dieser Studie unterstützen, sind in den Hintergrundinformationen zu diesem Artikel verfügbar.

## References

[1a] Z. J. Wang , H. Renata , N. E. Peck , C. C. Farwell , P. S. Coelho , F. H. Arnold , Angew. Chem. Int. Ed. 2014, 53, 6810–6813; 10.1002/anie.201402809 PMC4120663 24802161

[1b] K. Q. Zhang , Q. F. Deng , J. Luo , C. L. Gong , Z. G. Chen , W. Zhong , S. Q. Hu , H. F. Wang , ACS Catal. 2021, 11, 5100–5107;

[1c] Y. Su , M. Zhao , K. Han , G. Song , X. Li , Org. Lett. 2010, 12, 5462–5465; 21033749 10.1021/ol102306c

[1d] L. Ackermann , A. V. Lygin , N. Hofmann , Org. Lett. 2011, 13, 3278–3281; 21612195 10.1021/ol201244s

[1e] H. H. Xu , J. Song , H. C. Xu , ChemSusChem 2019, 12, 3060–3063; 30684294 10.1002/cssc.201803058

[1f] D. Wu , W. Fan , L. Wu , P. Chen , G. Liu , ACS Catal. 2022, 12, 5284–5291.

[2a] I. P. Beletskaya , A. V. Cheprakov , Chem. Rev. 2000, 100, 3009–3066; 11749313 10.1021/cr9903048

[2b] M. Oberholzer , R. Gerber , C. M. Frech , Adv. Synth. Catal. 2012, 354, 627–641.

[3a] Y. Li , C. Mück-Lichtenfeld , A. Studer , Angew. Chem. Int. Ed. 2016, 55, 14435–14438; 10.1002/anie.201608144 27735112

[3b] K. Liang , T. Li , N. Li , Y. Zhang , L. Shen , Z. Ma , C. Xia , Chem. Sci. 2020, 11, 2130–2135; 34123301 10.1039/c9sc06184c PMC8150107

[3c] J. J. Tsai , Y. H. Huang , C. M. Chou , Org. Lett. 2021, 23, 9468–9473. 34881572 10.1021/acs.orglett.1c03650

[4a] N. Gillaizeau-Simonian , E. Barde , A. Guérinot , J. Cossy , Chem. Eur. J. 2021, 27, 4004–4008; 33296109 10.1002/chem.202005129

[4b] N. Radhoff , A. Studer , Angew. Chem. Int. Ed. 2021, 60, 3561–3565; 10.1002/anie.202013275 PMC7898318 33215815

[5a] C. M. Holden , M. F. Greaney , Chem. Eur. J. 2017, 23, 8992–9008; 28401655 10.1002/chem.201700353

[5b] A. R. P. Henderson , J. R. Kosowan , T. E. Wood , Can. J. Chem. 2017, 95, 483–504;

[5c] D. M. Whalley , M. F. Greaney , Synthesis 2022, 54, 1908–1918;

[5d] S. M. Wales , R. K. Saunthwal , J. Clayden , Acc. Chem. Res. 2022, 55, 1731–1747. 35620846 10.1021/acs.accounts.2c00184 PMC9219115

[6a] W. Kong , M. Casimiro , N. Fuentes , E. Merino , C. Nevado , Angew. Chem. Int. Ed. 2013, 52, 13086–13090; 10.1002/anie.201307377 24174281

[6b] W. Kong , M. Casimiro , E. Merino , C. Nevado , J. Am. Chem. Soc. 2013, 135, 14480–14483; 24047140 10.1021/ja403954g

[6c] W. Kong , E. Merino , C. Nevado , Angew. Chem. Int. Ed. 2014, 53, 5078–5082; 10.1002/anie.201311241 24692217

[6d] W. Kong , N. Fuentes , A. García-Domínguez , E. Merino , C. Nevado , Angew. Chem. Int. Ed. 2015, 54, 2487–2491; 10.1002/anie.201409659 25597296

[6e] N. Fuentes , W. Kong , L. Fernández-Sánchez , E. Merino , C. Nevado , J. Am. Chem. Soc. 2015, 137, 964–973. 25561161 10.1021/ja5115858

[7] Für die Nutzung von *N*-zentrierten Radikalintermediaten in weiteren Transformationen, wie beispielsweise einer Oxindol-Zyklisierung, siehe Referenz [6].

[8] C. Hervieu , M. S. Kirillova , T. Suárez , M. Müller , E. Merino , C. Nevado , Nat. Chem. 2021, 13, 327–334. 33833448 10.1038/s41557-021-00668-4

[9a] S. Tang , L. Yuan , Y. L. Deng , Z. Z. Li , L. N. Wang , G. X. Huang , R. L. Sheng , Tetrahedron Lett. 2017, 58, 329–332;

[9b] Z. Ni , X. Huang , Y. Pan , Org. Lett. 2016, 18, 2612–2615; 27219900 10.1021/acs.orglett.6b01041

[9c] J. T. Yu , W. Hu , H. Peng , J. Cheng , Tetrahedron Lett. 2016, 57, 4109–4112;

[9d] F. L. Tan , R. J. Song , M. Hu , J. H. Li , Org. Lett. 2016, 18, 3198–3201; 27286238 10.1021/acs.orglett.6b01419

[9e] X. Su , H. Huang , W. Hong , J. Cui , M. Yu , Y. Li , Chem. Commun. 2017, 53, 13324–13327; 10.1039/c7cc08362a 29159337

[9f] Q. Tian , P. He , C. Kuang , Org. Biomol. Chem. 2014, 12, 6349–6353; 25027468 10.1039/c4ob01231c

[9g] L. Shi , H. Wang , H. Yang , H. Fu , Synlett 2015, 26, 688–694;

[9h] Z. He , P. Tan , C. Ni , J. Hu , Org. Lett. 2015, 17, 1838–1841; 25839912 10.1021/acs.orglett.5b00308

[9i] R. Caporaso , S. Manna , S. Zinken , A. R. Kochnev , E. R. Lukyanenko , A. V. Kurkin , A. P. Antonchick , Chem. Commun. 2016, 52, 12486–12489; 10.1039/c6cc07196a 27711354

[9j] J. K. Qiu , W. J. Hao , L. F. Kong , W. Ping , S. J. Tu , B. Jiang , Tetrahedron Lett. 2016, 57, 2414–2417;

[9k] M. Zhang , X. Ding , A. Lu , J. Kang , Y. Gao , Z. Wang , H. Li , Q. Wang , Org. Chem. Front. 2021, 8, 961–967;

[9l] S. Alazet , J. Preindl , R. Simonet-Davin , S. Nicolai , A. Nanchen , T. Meyer , J. Waser , J. Org. Chem. 2018, 83, 12334–12356; 30220207 10.1021/acs.joc.8b02068

[9m] L. Zheng , C. Yang , Z. Xu , F. Gao , W. Xia , J. Org. Chem. 2015, 80, 5730–5736; 25955879 10.1021/acs.joc.5b00677

[9n] X. F. Xia , S. L. Zhu , C. Chen , H. Wang , Y. M. Liang , J. Org. Chem. 2016, 81, 1277–1284; 26760053 10.1021/acs.joc.5b02594

[9o] K. Liu , L. C. Sui , Q. Jin , D. Y. Li , P. N. Liu , Org. Chem. Front. 2017, 4, 1606–1610;

[9p] J. T. Yu , R. Chen , J. Zhu , J. Cheng , Org. Biomol. Chem. 2017, 15, 5476–5479; 28650489 10.1039/c7ob01260h

[9q] P. Biswas , J. Guin , J. Org. Chem. 2018, 83, 5629–5638; 29696974 10.1021/acs.joc.8b00618

[9r] M. Li , C. T. Wang , Q. F. Bao , Y. F. Qiu , W. X. Wei , X. S. Li , Y. Z. Wang , Z. Zhang , J. L. Wang , Y. M. Liang , Org. Lett. 2021, 23, 751–756; 33474937 10.1021/acs.orglett.0c03973

[9s] H. Zhang , C. Pan , N. Jin , Z. Gu , H. Hu , C. Zhu , Chem. Commun. 2015, 51, 1320–1322; 10.1039/c4cc08629e 25482658

[9t] M. Hu , L. Y. Guo , Y. Han , F. L. Tan , R. J. Song , J. H. Li , Chem. Commun. 2017, 53, 6081–6084. 10.1039/c7cc02608k 28503688

[10] Für Palladium-katalysierte Heck Reaktionskaskaden unter Nutzung von α-Bromocarbonylen, siehe: J. H. Fan , J. Yang , R. J. Song , J. H. Li , Org. Lett. 2015, 17, 836–839. 25654662 10.1021/ol503660a

[11] T. Naito , R. Dohmori , M. Shimoda , Pharm. Bull. 1955, 3, 34–37. 14384465 10.1248/cpb1953.3.34

[12] H. L. Barlow , P. T. G. Rabet , A. Durie , T. Evans , M. F. Greaney , Org. Lett. 2019, 21, 9033–9035. 31674791 10.1021/acs.orglett.9b03429

[13a] K. Morita , Z. Suzuki , H. Hirose , Bull. Chem. Soc. Jpn. 1968, 41, 2815–2815;

[13b] M. E. D. Hillman , A. B. Baylis , German Patent 2155113, 1972;

[13c] M. M. Rauhut , H. Currier , U.S. Patent 3074999, 1963.

[14] Für eine *N*-heterozyklische Carben-katalysierte Arylwanderung, die β-arylierte Acrylamide (Zimtsäureamide) liefert, siehe: K. Yasui , M. Kamitani , H. Fujimoto , M. Tobisu , Org. Lett. 2021, 23, 1572–1576. 33577343 10.1021/acs.orglett.0c04281

[15] Der Einsatz der halben Menge an DABCO lieferte das Produkt nur mit fast einer stöchiometrischen Ausbeute von 53 % bei Raumtemperatur. Die Ausbeute konnte durch Erhöhung der Temperatur auf 80 °C auf 80 % gesteigert werden, wobei auch etwas mehr **1-sa** entstand (siehe Hintergrundinformationen für weitere Details).

[16] H. Woolven , C. González-Rodríguez , I. Marco , A. L. Thompson , M. C. Willis , Org. Lett. 2011, 13, 4876–4878. 21866926 10.1021/ol201957n

[17] Y. Kwon , I. Kim , S. Kim , Org. Lett. 2014, 16, 4936–4939. 25211061 10.1021/ol502465e

[18] M. Ihara , M. Tsuruta , K. Fukumoto , T. Kametani , J. Chem. Soc. Chem. Commun. 1985, 1159–1161.

[19] E. E. Kwan , Y. Zeng , H. A. Besser , E. N. Jacobsen , Nat. Chem. 2018, 10, 917–923. 30013193 10.1038/s41557-018-0079-7 PMC6105541

[20] Da die DABSO-Entstehung experimentell bestätigt werden konnte, erworgen wir die Möglichkeit einer DABCO-vermittelten, nukleophilen Substitution an SO_2_ vom Intermediat **C**. Die Suche nach solch einem SO_2_-Transfer resultierte jedoch in einer kontinuierlich ansteigenden Energie entlang der internen Reaktionskoordinate, wodurch die Wahrscheinlichkeit einer solchen Möglichkeit sehr gering ist (siehe Hintergrundinformationen für weitere Details).

[21] Ein konkurrierender Übergangszustand, der eine Protonierung am Sauerstoffatom involviert und 6.6 kcal mol^−1^ über **F** liegt, wurde ebenfalls gefunden. In dem Fall war die Reaktion jedoch endergonisch, was einen reversiblen Prozess zur Folge hätte (siehe Hintergrundinformationen für weitere Details).

