# Supplementary material for: Synthese von α‐Arylacrylamiden via Lewis Base vermitteltem Aryl/Wasserstoff‐Austausch
Source: Angew Chem Weinheim Bergstr Ger. 2022 Aug 29;134(40):e202207475. doi: 10.1002/ange.202207475 (PMC10947125; doi:10.1002/ange.202207475)

Angewandte Chemie
Eine Zeitschrift der Gesellschaft Deutscher Chemiker

Supporting Information

Synthese von α-Arylacrylamiden via Lewis Base vermitteltem Aryl/Wasserstoff-Austausch

*M. Lemmerer, H. Zhang, A. J. Fernandes, T. Fischer, M. Mießkes, Y. Xiao, N. Maulide**

Contents

General information 7

Optimisation tables 8

  Lewis base optimisation table 8

  Solvent and concentration optimisation table 9

  Substoichiometric DABCO reactions 9

Limitations of *N*–sulfonyl acrylimides 10

Starting material synthesis 11

  General Procedure A: Sulfonamide synthesis 11

  General Procedure B: Acrylimide synthesis 11

  General Procedure C: β-substituted acrylimide synthesis 11

  Previously reported sulfonamides 12

  0f: *N*-(4,4-Dimethoxybutyl)-4-nitrobenzenesulfonamide 13

  0j: *tert*-Butyl 3-(2-((4-nitrophenyl)sulfonamido)ethyl)-1*H*-indole-1-carboxylate 13

  0k: Ethyl (*E*)-6-((4-nitrophenyl)sulfonamido)hex-2-enoate 14

  0n: *N*-Methyl-2,5-bis(trifluoromethyl)benzenesulfonamide 14

  0o: *N*4-Cyano-*N*-methyl-2-(trifluoromethyl)benzenesulfonamide 15

  Sulfonyl carboximides 15

  1a: *N*-Methyl-*N*-((4-nitrophenyl)sulfonyl)acrylamide 15

  1b: *N*-Isopropyl-*N*-((4-nitrophenyl)sulfonyl)acrylamide 16

  1c: *N*-(*tert*-Butyl)-*N*-((4-nitrophenyl)sulfonyl)acrylamide 16

  1d: *N*-Allyl-*N*-((4-nitrophenyl)sulfonyl)acrylamide 17

  1e: *N*-Benzyl-*N*-((4-nitrophenyl)sulfonyl)acrylamide 17

  1f: *N*-(4,4-Dimethoxybutyl)-*N*-((4-nitrophenyl)sulfonyl)acrylamide 18

  1g: Methyl *N*-acryloyl-*N*-((4-nitrophenyl)sulfonyl)glycinate 18

  1h: Methyl *N*-acryloyl-*N*-((4-nitrophenyl)sulfonyl)methioninate 19

  1i: *N*-(3,4-Dimethoxyphenethyl)-*N*-((4-nitrophenyl)sulfonyl)acrylamide 19

  1j: *tert*-Butyl 3-(2-(*N*-((4-nitrophenyl)sulfonyl)acrylamido)ethyl)-1*H*-indole-1-carboxylate 20

  1k: Ethyl (*E*)-6-(*N*-((4-nitrophenyl)sulfonyl)acrylamido)hex-2-enoate 20

  1l: *N*-Methyl-*N*-((2-nitrophenyl)sulfonyl)acrylamide 21

  1m: *N*-((2-Methoxy-4-nitrophenyl)sulfonyl)-*N*-methylacrylamide 21

  1n: *N*-((2,5-bis(trifluoromethyl)phenyl)sulfonyl)-N-methylacrylamide 22

  1o: *N*-((4-Cyano-2-(trifluoromethyl)phenyl)sulfonyl)-*N*-methylacrylamide 22

  SI1: *N*-methyl-*N*-(pyridin-2-ylsulfonyl)acrylamide 23

  1p: 2-(*N*-acryloyl-*N*-methylsulfamoyl)pyridine 1-oxide 23

  1o: (*E*)-*N*-Methyl-*N*-((4-nitrophenyl)sulfonyl)but-2-enamide 24

1r: (*E*)-*N*-Methyl-*N*-((4-nitrophenyl)sulfonyl)oct-2-enamide 24
1s: *N*,3-Dimethyl-*N*-((4-nitrophenyl)sulfonyl)but-2-enamide 25
1t: (*E*)-*N*,4-Dimethyl-*N*-((4-nitrophenyl)sulfonyl)pent-2-enamide 25
1u: (2*E*,4*E*)-*N*-Methyl-*N*-((4-nitrophenyl)sulfonyl)hexa-2,4-dienamide 26
SI2: *N*-((4-nitrophenyl)sulfonyl)-*N*-phenylacrylamide 26
SI3: *N*-((4-Cyanophenyl)sulfonyl)-*N*-methylacrylamide 27
Characterisation of aryl migrated products 28
General Procedure D: Truce-Smiles rearrangement 28
2a: *N*-Methyl-2-(4-nitrophenyl)acrylamide 28
2b: *N*-Isopropyl-2-(4-nitrophenyl)acrylamide 29
2c: *N*-(*tert*-Butyl)-2-(4-nitrophenyl)acrylamide 29
2d: *N*-allyl-2-(4-nitrophenyl)acrylamide 30
2e: *N*-Benzyl-2-(4-nitrophenyl)acrylamide 30
2f: *N*-(4,4-dimethoxybutyl)-2-(4-nitrophenyl)acrylamide 31
2g: Methyl (2-(4-nitrophenyl)acryloyl)glycinate 31
2h: Methyl (2-(4-nitrophenyl)acryloyl)methioninate 32
2i: *N*-(3,4-Dimethoxyphenethyl)-2-(4-nitrophenyl)acrylamide 32
2j: *tert*-Butyl 3-(2-(2-(4-nitrophenyl)acrylamido)ethyl)-1H-indole-1-carboxylate 33
2k: Ethyl (*E*)-6-(2-(4-nitrophenyl)acrylamido)hex-2-enoate 33
2l: *N*-Methyl-2-(2-nitrophenyl)acrylamide 34
2m: 2-(2-Methoxy-4-nitrophenyl)-*N*-methylacrylamide 34
2n: 2-(2,5-Bis(trifluoromethyl)phenyl)-*N*-methylacrylamide 35
2o: 2-(4-Cyano-2-(trifluoromethyl)phenyl)-*N*-methylacrylamide 35
2p: 2-(*N*-Acryloyl-*N*-methylsulfamoyl)pyridine 1-oxide 36
2q: (*Z*)- and (*E*)-*N*-Methyl-2-(4-nitrophenyl)but-2-enamide 36
2r: (*E*)-N-Methyl-2-(4-nitrophenyl)oct-3-enamide 37
2s and *iso*-2s: *N*,3-Dimethyl-2-(4-nitrophenyl)but-3-enamide 37
2t: *N*,4-Dimethyl-2-(4-nitrophenyl)pent-3-enamide 38
2u: *N*-methyl-2-(4-nitrophenyl)hexadienamide [isomeric mixture] 38
SI4: 2-(4-Nitrophenyl)-*N*-phenylacrylamide 39
SI5: 2-(4-Cyanophenyl)-*N*-methylacrylamide 39
Characterisation of byproducts 40
1-rc: *N*1,*N*5-Dimethyl-2-methylene-*N*1,*N*5-bis((4-nitrophenyl)sulfonyl)pentanediamide 40
1-sa: *N*-Methyl-3-((*N*-methyl-4-nitrophenyl)sulfonamido)-*N*-((4-nitrophenyl)sulfonyl)-propanamide 40
Characterisation of cyclisation reaction products 41
3: 1-Methyl-3-(4-nitrophenyl)-5,6-diphenylpyridin-2(1*H*)-one 41

4: 1-(2-Methoxypyrrolidin-1-yl)-2-(4-nitrophenyl)prop-2-en-1-one 41
5: (*Z*)-*N*,5,5-Trimethyl-3-(4-nitrophenyl)furan-2(5*H*)-imine 42
6: Ethyl 6-(4-nitrophenyl)-5-oxooctahydroindolizine-8-carboxylate 42
Further experimental reaction investigations 44
Brønsted base comparison 44
Observation of DABSO in the reaction mixture 44
DABSO spectra comparison 45
Deuterium NMR investigation 46
SI6: *N*-methyl-*N*-((4-nitrophenyl)sulfonyl)propiolamide 48
1a-*d*3: *N*-methyl-*N*-((4-nitrophenyl)sulfonyl)acrylamide-d3 48
Computations 50
Overall mechanism 51
Computed scan for the spontaneous *N*-SO_2 bond dissociation 52
Computed scan for the direct DABCO-mediated SO_2 transfer 52
MO representation of intermediate E 52
List of coordinates (xyz) 53
NMR spectra 64
0f: *N*-(4,4-Dimethoxybutyl)-4-nitrobenzenesulfonamide 64
0j: *tert*-Butyl 3-(2-((4-nitrophenyl)sulfonamido)ethyl)-1*H*-indole-1-carboxylate 65
0k: Ethyl-(*E*)-6-((4-nitrophenyl)sulfonamido)hex-2-enoate 66
0n: *N*-Methyl-2,5-bis(trifluoromethyl)benzenesulfonamide 67
0o: *N*-4-Cyano-*N*-methyl-2-(trifluoromethyl)benzenesulfonamide 69
1a: *N*-Methyl-*N*-((4-nitrophenyl)sulfonyl)acrylamide 71
1b: *N*-Isopropyl-*N*-((4-nitrophenyl)sulfonyl)acrylamide 72
1c: *N*-(*tert*-Butyl)-*N*-((4-nitrophenyl)sulfonyl)acrylamide 73
1d: *N*-Allyl-*N*-((4-nitrophenyl)sulfonyl)acrylamide 74
1e: *N*-Benzyl-*N*-((4-nitrophenyl)sulfonyl)acrylamide 75
1f: *N*-(4,4-Dimethoxybutyl)-*N*-((4-nitrophenyl)sulfonyl)acrylamide 76
1g: Methyl-*N*-acryloyl-*N*-((4-nitrophenyl)sulfonyl)glycinate 77
1h: Methyl-*N*-acryloyl-*N*-((4-nitrophenyl)sulfonyl)methioninate 78
1i: *N*-(3,4-Dimethoxyphenethyl)-*N*-((4-nitrophenyl)sulfonyl)acrylamide 79
1j: *tert*-Butyl 3-(2-(*N*-((4-nitrophenyl)sulfonyl)acrylamido)ethyl)-1*H*-indole-1-carboxylate 80
1k: Ethyl-(*E*)-6-(*N*-((4-nitrophenyl)sulfonyl)acrylamido)hex-2-enoate 81
1l: *N*-Methyl-*N*-((2-nitrophenyl)sulfonyl)acrylamide 82
1m: *N*-((2-Methoxy-4-nitrophenyl)sulfonyl)-*N*-methylacrylamide 83
1n: *N*-((2,5-Bis(trifluoromethyl)phenyl)sulfonyl)-N-methylacrylamide 84

1o: *N*-((4-Cyano-2-(trifluoromethyl)phenyl)sulfonyl)-*N*-methylacrylamide 86
SI1: *N*-methyl-*N*-(pyridin-2-ylsulfonyl)acrylamide.. 88
1p: 2-(*N*-acryloyl-*N*-methylsulfamoyl)pyridine 1-oxide.. 89
1q: (*E*)-*N*-Methyl-*N*-((4-nitrophenyl)sulfonyl)but-2-enamide 90
1r: (*E*)-*N*-Methyl-*N*-((4-nitrophenyl)sulfonyl)oct-2-enamide 91
1s: *N*-3-Dimethyl-*N*-((4-nitrophenyl)sulfonyl)but-2-enamide.. 92
1t: (*E*)-*N*-4-Dimethyl-*N*-((4-nitrophenyl)sulfonyl)pent-2-enamide.. 93
1u: (*2E*,*4E*)-*N*-Methyl-*N*-((4-nitrophenyl)sulfonyl)hexa-2,4-dienamide.. 94
SI2: *N*-((4-Nitrophenyl)sulfonyl)-*N*-phenylacrylamide 95
SI3: *N*-((4-Cyanophenyl)sulfonyl)-*N*-methylacrylamide 96
2a: *N*-Methyl-2-(4-nitrophenyl)acrylamide.. 97
2b: *N*-Isopropyl-2-(4-nitrophenyl)acrylamide 98
2c: *N*-(*tert*-Butyl)-2-(4-nitrophenyl)acrylamide... 99
2d: *N*-Allyl-2-(4-nitrophenyl)acrylamide 100
2e: *N*-Benzyl-2-(4-nitrophenyl)acrylamide.. 101
2f: *N*-(4,4-Dimethoxybutyl)-2-(4-nitrophenyl)acrylamide.. 102
2g: Methyl-(2-(4-nitrophenyl)acryloyl)glycinate 103
2h: Methyl-(2-(4-nitrophenyl)acryloyl)methioninate 104
2i: *N*-(3,4-Dimethoxyphenethyl)-2-(4-nitrophenyl)acrylamide... 105
2j: *tert*-Butyl 3-(2-(2-(4-nitrophenyl)acrylamido)ethyl)-1H-indole-1-carboxylate 106
2k: Ethyl-(*E*)-6-(2-(4-nitrophenyl)acrylamido)hex-2-enoate 107
2l: *N*-Methyl-2-(2-nitrophenyl)acrylamide... 108
2m: 2-(2-Methoxy-4-nitrophenyl)-*N*-methylacrylamide.. 109
2n: 2-(2,5-Bis(trifluoromethyl)phenyl)-*N*-methylacrylamide 110
2o: 2-(4-Cyano-2-(trifluoromethyl)phenyl)-*N*-methylacrylamide 112
2p: 2-(3-(Methylamino)-3-oxoprop-1-en-2-yl)pyridine 1-oxide.. 114
2q: (*Z*)- and (*E*)-*N*-Methyl-2-(4-nitrophenyl)but-2-enamide 115
2r: (*E*)-*N*-Methyl-2-(4-nitrophenyl)oct-3-enamide.. 116
2s: *N*-3-Dimethyl-2-(4-nitrophenyl)but-3-enamide... 117
iso-2s: *N*-3-Dimethyl-2-(4-nitrophenyl)but-2-enamide... 118
2t: *N*-4-Dimethyl-2-(4-nitrophenyl)pent-3-enamide... 119
2u: *N*-Methyl-2-(4-nitrophenyl)hexadienamide [isomeric mixture] 120
SI4: 2-(4-Nitrophenyl)-*N*-phenylacrylamide 121
SI5: 2-(4-Cyanophenyl)-*N*-methylacrylamide 122
1-rc: *N*-Methyl-3-((*N*-methyl-4-nitrophenyl)sulfonamido)-*N*-((4-nitrophenyl)sulfonyl)-propanamide 123

1-sa: *N*-Methyl-3-((*N*-methyl-4-nitrophenyl)sulfonamido)-*N*-((4-nitrophenyl)sulfonyl)-propanamide 124

3: 1-Methyl-3-(4-nitrophenyl)-5,6-diphenylpyridin-2(1*H*)-one 125

4: 1-(2-Methoxypyrrolidin-1-yl)-2-(4-nitrophenyl)prop-2-en-1-one 126

5: (*Z*)-*N*-5,5-Trimethyl-3-(4-nitrophenyl)furan-2(5*H*)-imine 127

6: Ethyl-6-(4-nitrophenyl)-5-oxooctahydroindolizine-8-carboxylate 128

SI6: *N*-Methyl-*N*-((4-nitrophenyl)sulfonyl)propiolamide 129

1a-*d*3: *N*-methyl-*N*-((4-nitrophenyl)sulfonyl)acrylamide-d3 130

General information

Unless otherwise stated, all glassware was oven-dried before use and all reactions were performed under air. All reagents were used as received from commercial suppliers unless otherwise stated. Reaction progress was monitored by thin layer chromatography (TLC) performed on aluminium plates coated with silica gel F254 with 0.2 mm thickness. Chromatograms were visualised by fluorescence quenching with UV light at 254 nm or by staining using potassium permanganate. Flash column chromatography was performed on an Isolera 4 or Select medium pressure chromatography system (Biotage) using silica gel 60 (230-400 mesh, Merck and co.). Neat infrared spectra were recorded using a Perkin-Elmer Spectrum 100 FT-IR spectrometer. Wavenumbers (ν_{max}) are reported in cm^{-1}. Mass spectra were obtained using a Bruker maXis UHR-TOF (Qq-TOF) spectrometer, using electrospray ionization (ESI). All ^{1}H NMR and ^{13}C NMR spectra were recorded using a Bruker AV-400, AV-500, AV-600 or AV-700 spectrometer at 300K. Chemical shifts are given in parts per million (ppm, δ), referenced to the solvent residual peak of $CDCl_3$, defined at δ = 7.26 ppm (^{1}H NMR) and δ = 77.16 (^{13}C NMR), or CD_3OD, defined at δ = 3.31 ppm (^{1}H NMR). Coupling constants are quoted in Hz (*J*). ^{1}H NMR splitting patterns are designated as singlet (s), doublet (d), triplet (t), quartet (q) as they appeared in the spectrum. If the appearance of a signal differs from the expected splitting pattern, the observed pattern is designated as apparent (app). Splitting patterns that could not be interpreted or easily visualized are designated as multiplet (m) or broad (b).

Optimisation tables

Lewis base optimisation table

Table S1: Lewis base optimisation.

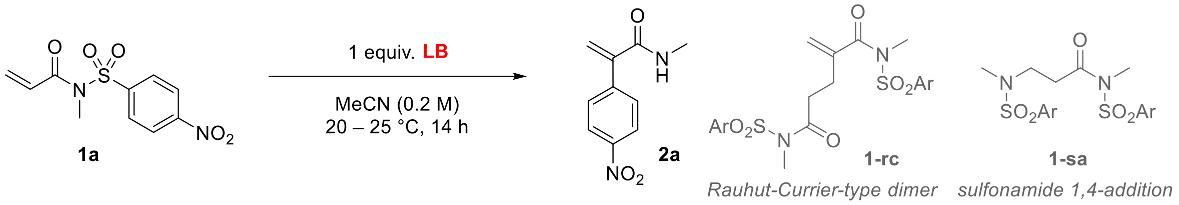

Entry	LB	Yield [%][a]	Yield 1-rc [%][a]	Yield 1-sa [%][a]
1	DABCO	40	5	4
2	DBU	9	<1	n.d.[c]
3	DMAP	12	<1	n.d.[c]
4	quinuclidine	32	1	14
5	3-quinuclidinol	22	5	5
6	*N*,*N*-dimethylglycine	<1	<1	<1
7	*N*-benzyl-piperidine-4-ol	<1	<1	<1
8	*N*-methylmorpholine	<1	<1	<1
9	*N*-methylpyrrolidine	35	5	6
10	PEt_3, reaction under argon	<1	<1	<1
11	PCy_3, reaction under argon	<1	<1	<1
12	PPh_3, reaction under argon	<1	n.d.[c]	n.d.[c]
13	thiazole	<1	<1	<1
14	pyridine	<1	<1	<1
15	tetrahydrothiophene	<1	<1	<1
16	-	<1[b]	<1	<1
17	DABCO, reaction under argon	31	4	4

*Reactions were carried out at 0.1 mmol scale. [a] Yield was measured by 1H NMR using mesitylene as an internal standard. [b] Even after two weeks, no degradation of **1a** was observed in the absence of any Lewis base. [c] Not determined due to peak overlap.*

Solvent and concentration optimisation table

Table S2: Solvent and concentration optimization.

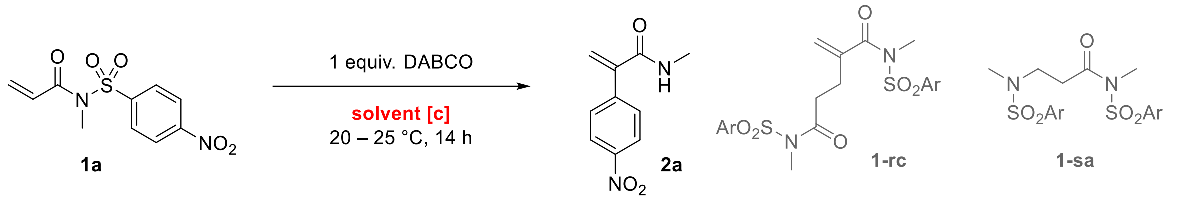

Rauhut-Currier-type dimer   *sulfonamide 1,4-addition*

Entry	Solvent	c [M]	Yield [%][a]	Yield 1a-rc [%][a]	Yield 1a-sa [%][a]
1	1,4-dioxane	0.2	18	22	1
2	DMF	0.2	20	<1	n.d.[b]
3	acetone	0.2	39	6	n.d.[b]
4	$MeNO_2$	0.2	<1	<1	<1
5	MeCN/HFIP 4/1	0.2	<1	n.d.[b]	n.d.[b]
6	MeCN/^{i}PrOH 3/1	0.2	26	3	3
7	MeCN/^{i}PrOH 9/1	0.2	31	6	3
8	MeCN/^{t}BuOH 9/1	0.2	30	3	2
9	MeCN + 1 equiv. BHT	0.2	29	4	n.d.[b]
10	MeCN + 1 equiv. BnOH	0.2	30	3	2
11	MeCN	0.05	63	1	n.d.[b]
12	MeCN, reaction for 72 h	0.05	94	3	2
13	MeCN, reaction at 40 °C	0.05	87	n.d.[b]	n.d.[b]

Reactions were carried out at 0.1 mmol scale. [a] Yield was measured by 1H NMR using mesitylene as an internal standard. [b] Not determined due to peak overlap.

Substoichiometric DABCO reactions

Table S3: Screening of DABCO loading.

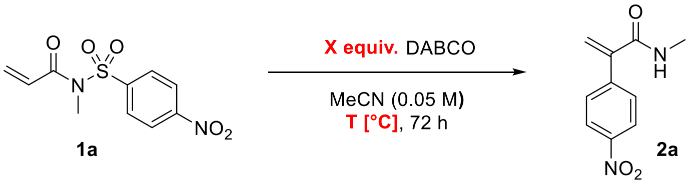

Entry	Equiv.	Temperature [°C]	Yield [%][a]
1	0.5	20 – 25	53
2	0.5	80	80
3	0.25	80	68

Reactions were carried out at 0.2 mmol scale. [a] Yield was measured by ^{1}H NMR using mesitylene as an internal standard.

Limitations of *N*–sulfonyl acrylimides

The following compounds yielded the aryl migrated products, unless otherwise stated, in up to 15 % at 20 – 25, 80 or 140 °C (microwave), respectively. At elevated temperatures, the cleavage of the sulfonamide bound, presumingly initiated by the 1,2 attack of DABCO, becomes predominant.

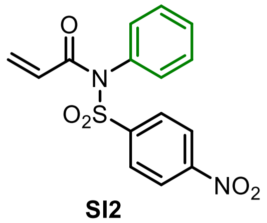

SI2
yielded **SI4**, 28%

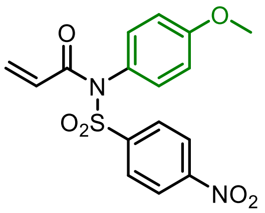

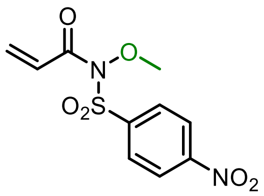

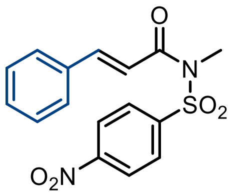

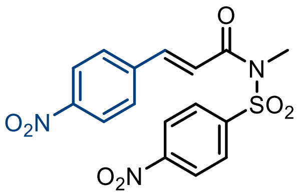

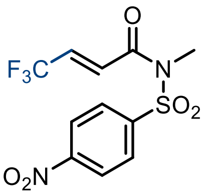

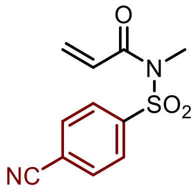

SI3
yielded **SI5**, 33%

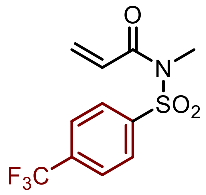

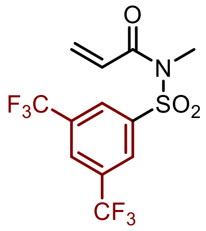

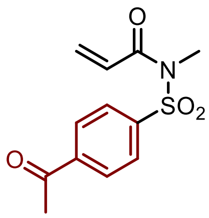

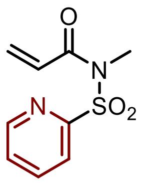

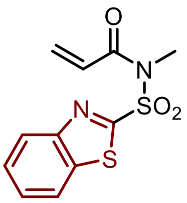

Starting material synthesis

General Procedure A: Sulfonamide synthesis

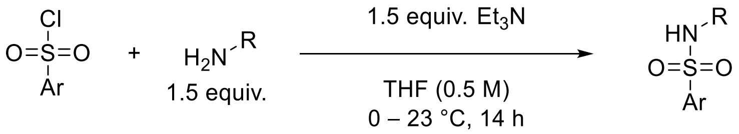

To sulfonyl chloride (1.0 equiv.) in THF (0.5 M) was added primary amine (1.5 equiv.) and Et_3N (1.5 equiv.). The solution was stirred for 14 h, upon which it was diluted with EtOAc. The mixture was washed with aqueous HCl (1 M) and brine. The organic phase was dried over $MgSO_4$, filtered and the solvent was removed under reduced pressure to afford the sulfonamide which was used directly without further purification.

General Procedure B: Acrylimide synthesis

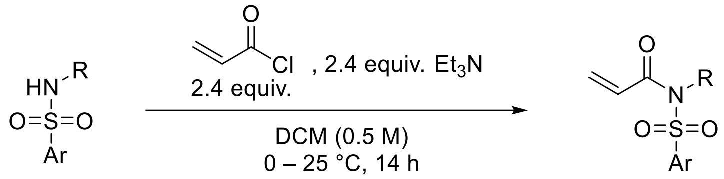

To sulfonamide (1.0 equiv.) in DCM (0.25 M) in a flame-dried Schlenk flask under argon at 0 °C were added Et_3N (2.4 equiv.) and acryloyl chloride (2.4 equiv.) dropwise. The mixture was stirred at 23 °C for 14 h, upon which EtOAc was added and the mixture was transfered to a seperatory funnel. The mixture was washed with saturated, aqueous $NaHCO_3$ solution and brine. The organic phase was dried over $MgSO_4$, filtered and the solvent was removed under reduced pressure. Purification by column chromatography with EtOAc in heptane afforded the desired products.

General Procedure C: β-substituted acrylimide synthesis

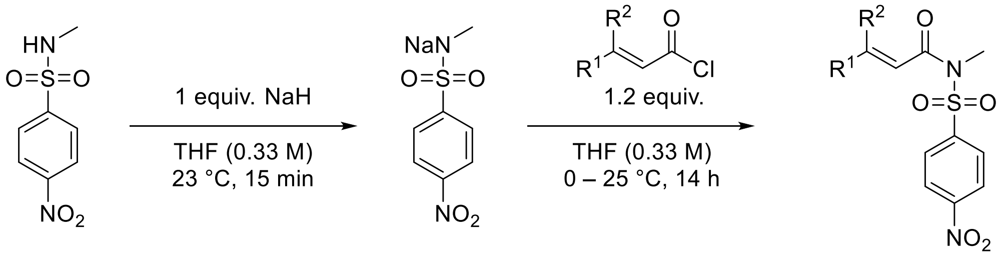

To *N*-Methyl-*N*-((4-nitrophenyl)sulfonyl)amide (1.0 equiv.) in THF (0.25 M) in a flame-dried Schlenk-flask under argon was added NaH (1.0 equiv.). The mixture was stirred at 23 °C for 15 min, upon which acyl chloride (1.2 equiv.) was added and the formed suspension was stirred for 14 h. Then, EtOAc was added and the mixture was transfered to a seperatory funnel. The mixture was washed with saturated, aqueous $NaHCO_3$ solution and brine. The organic phase was dried over $MgSO_4$, filtered and the solvent was removed under reduced pressure. Purification by column chromatography with EtOAc in heptane afforded the desired products.

Previously reported sulfonamides

The following sulfonamides have been synthesized according to the procedures in the references specified bellow.[1,2,3,4,5,6,7,8]

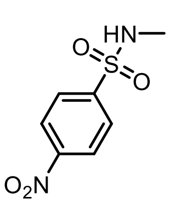

0a[1]

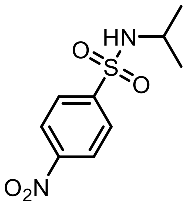

0b[2]

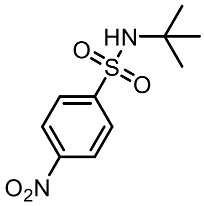

0c[3]

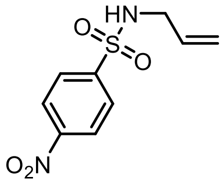

0d[4]

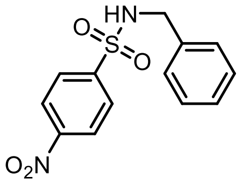

0e[1]

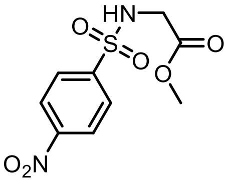

0g[5]

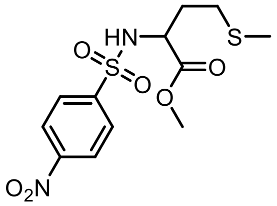

0h[6]

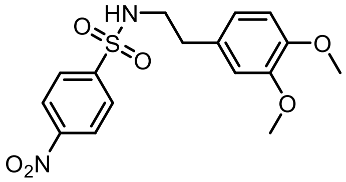

0i[7]

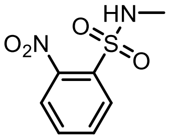

0l[1]

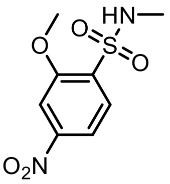

0m[1]

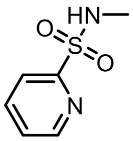

0p[8]

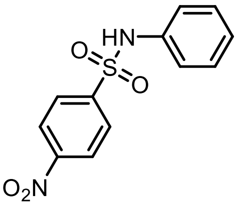

SI0a[1]

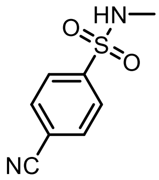

SI0b[9]

---

[1] S. Johnson, E. Kovács, M. F. Greaney, *Chem. Commun.* **2020**, *56*, 3222–3224.
[2] A. Saidykhan, R. D. Bowen, R. T. Gallagher, W. H. C. Martin, *Tet. Lett.* **2015**, *56*, 66–68.
[3] A. C. Brueckner, E. N. Hancock, E. J. Anders, M. M. Tierney, H. R. Morgan, K. A. Scott, A. A. Lamar, *Org. Biomol. Chem.* **2016**, *14*, 4387–4392.
[4] X. Zhang, B. Cao, S. Yu, X. Zhang, *Angew. Chem. Int. Ed.* **2010**, *49*, 4047–4050.
[5] D. Pulido, F. Albericio, M. Royo, *Org. Lett.* **2014**, *16*, 1318–1321.
[6] C. Loro, R. Sala, M. Penso, F. Foschi, *Adv. Synth. Catal.* **2021**, *363*, 3983–3994.
[7] R. Pingaew, S. Prachayasittikul, S. Ruchirawat, V. Prachayasittikul, *Med Chem Res* **2013** *22*, 267–277.
[8] C.R. Gonçalves, M. Lemmerer, C. J. Teskey, P. Adler, D. Kaiser, B. Maryasin, L. González, N. Maulide *J. Am. Chem. Soc.* **2019**, *141, 46*, 18437–18443.
[9] S. Che, Q. Zhu, Z. Luo, Y. Lian, Z. Zhao, *Synth. Commun.* **2021**, *51*, 935–942.

0f: *N*-(4,4-Dimethoxybutyl)-4-nitrobenzenesulfonamide

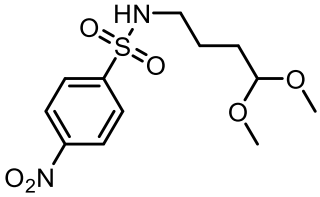

Following General Procedure A using 4,4-dimethoxybutan-1-amine (2.22 g, 10.0 mmol), the purification by column chromatography on silica (5 – 50% EtOAc in heptane) afforded compound **0f** in 97% (3.09 g) yield as a yellow solid.

^{1}H NMR (400 MHz, $CDCl_3$): δ 8.40 – 8.33 (m, 2H), 8.07 – 8.02 (m, 2H), 5.06 (t, *J* = 6.0 Hz, 1H), 4.30 (t, *J* = 4.9 Hz, 1H), 3.31 (s, 6H), 3.04 (app. q, *J* = 6.4 Hz, 2H), 1.65 – 1.58 (m, 4H) ppm.

^{13}C NMR (150 MHz, $CDCl_3$): δ 150.2, 146.3, 128.4 (2C), 124.5 (2C), 104.4, 53.6 (2C), 43.3, 29.9, 24.5 ppm.

IR (neat) ν_{max}: 3286, 2956, 1529, 1349, 1163, 735, 611 cm^{-1}.

HRMS (ESI$^+$): exact mass calculated for $[M+Na]^+$ ($C_{12}H_{18}N_2NaO_6S^+$) requires *m/z* 341.0778, found *m/z* 341.0775.

0j: *tert*-Butyl 3-(2-((4-nitrophenyl)sulfonamido)ethyl)-1*H*-indole-1-carboxylate

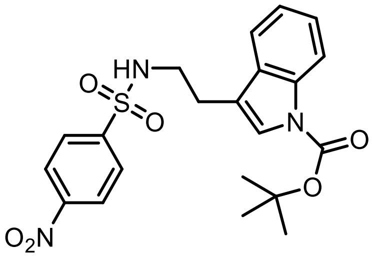

Following General Procedure A using *tert*-butyl 3-(2-aminoethyl)-1H-indole-1-carboxylate (1.12 g, 4.30 mmol), the purification by column chromatography on silica (5 – 80% EtOAc in heptane), afforded compound 0j in 88% (1.68 g) yield as an off-white solid.

^{1}H NMR (400 MHz, $CDCl_3$): δ 8.14 – 8.06 (m, 2H), 8.01 (d, *J* = 7.3 Hz, 1H), 7.84 – 7.76 (m, 1H), 7.35 – 7.23 (m, 3H), 7.19 – 7.12 (m, 1H), 4.54 (t, *J* = 5.8 Hz, 1H), 3.40 (app. q, *J* = 6.2 Hz, 2H), 2.89 (t, *J* = 6.3 Hz, 2H), 1.69 (s, 9H) ppm.

^{13}C NMR (100 MHz, $CDCl_3$): δ 149.9, 149.6, 145.5, 129.6, 128.0 (2C), 125.0, 124.1 (2C), 124.0, 122.8, 118.6, 115.9, 115.6, 84.4, 42.7, 28.3 (3C), 25.6 ppm. (2 quaternary carbons did not relax)

IR (neat) ν_{max}: 3295, 2928, 1730, 1530, 1348, 1157, 745 cm^{-1}.

HRMS (ESI$^+$): exact mass calculated for $[M+Na]^+$ ($C_{21}H_{23}N_3NaO_6S^+$) requires *m/z* 468.1200, found *m/z* 468.1197.

0k: Ethyl (*E*)-6-((4-nitrophenyl)sulfonamido)hex-2-enoate

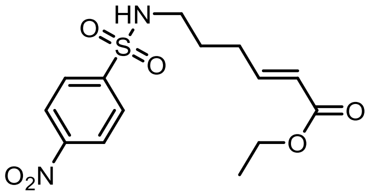

Following the procedure reported by Takemoto *et al.*,[9] using *N*-p-nitrophenylsulfonyl pyrrolidone (1.62 g, 6.00 mmol), diisobutylaluminum hydride (1.2 M in toluene, 5.00 mL, 6.00 mmol) and ethyl (Triphenylphosphoranylidene)acetate (3.14 g, 9.00 mmol), the purification by column chromatography on silica (20 – 50% EtOAc in heptane) afforded compound **0k** in 36% (743 mg) yield as a yellow solid.

^{1}H NMR (400 MHz, $CDCl_3$): δ 8.37 (d, *J* = 8.8 Hz, 2H), 8.09 – 8.02 (m, 2H), 6.84 (dt, *J* = 15.6, 6.9 Hz, 1H), 5.77 (d, *J* = 15.7 Hz, 1H), 4.74 (d, *J* = 5.5 Hz, 1H), 4.17 (q, *J* = 7.1 Hz, 2H), 3.04 (q, *J* = 6.8 Hz, 2H), 2.23 (td, *J* = 8.3, 1.2 Hz, 2H), 1.69 (p, *J* = 7.2 Hz, 2H), 1.28 (t, *J* = 7.1 Hz, 3H) ppm.

^{13}C NMR (100 MHz, $CDCl_3$): δ 166.5, 150.3, 146.8, 146.0, 128.4 (2C), 124.6 (2C), 122.7, 60.6, 42.8, 29.0, 28.3, 14.4 ppm.

IR (neat) $ν_{max}$: 3294, 2928, 1714, 1529, 1349, 1160, 906, 726, 609 cm^{-1}.

HRMS (ESI$^+$): exact mass calculated for $[M+Na]^+$ ($C_{14}H_{18}N_2NaO_6S^+$) requires *m/z* 365.0778, found *m/z* 365.0769.

0n: *N*-Methyl-2,5-bis(trifluoromethyl)benzenesulfonamide

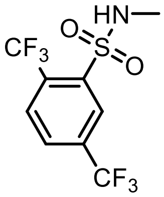

Following General Procedure A using 2,5-bis(trifluoromethyl)benzenesulfonyl chloride (375 mg, 1.20 mmol), compound **0n** was obtained in 91% (337 mg) yield as a white solid.

^{1}H NMR (400 MHz, $CDCl_3$): δ 8.49 (s, 1H), 8.05 (d, *J* = 8.2 Hz, 1H), 7.98 (d, *J* = 8.2 Hz, 1H), 4.64 (bs, 1H), 2.75 (s, 3H) ppm.

^{13}C NMR (100 MHz, $CDCl_3$): δ 139.6, 134.8 (q, *J* = 34.1 Hz), 131.0 (q, *J* = 32.4 Hz), 129.9 – 129.5 (m, 2C), 129.2 – 129.0 (m), 122.6 (q, *J* = 273.4 Hz), 122.4 (q, J = 274.6 Hz), 29.6 ppm.

^{19}F NMR (377 MHz, $CDCl_3$): δ -58.5, -63.3 ppm.

IR (neat) $ν_{max}$: 3346, 1420, 1331, 1309, 1266, 1191, 1129, 1032, 585 cm^{-1}.

HRMS (ESI$^+$): exact mass calculated for $[M+Na]^+$ ($C_9H_7F_6NNaO_2S^+$) requires *m/z* 329.9994, found *m/z* 329.9995.

[9] T. Azuma, A. Murata, Y. Kobayashi, T. Inokuma, Y. Takemoto, *Org. Lett.* **2014**, *16*, 4256−4259.

0o: *N*4-Cyano-*N*-methyl-2-(trifluoromethyl)benzenesulfonamide

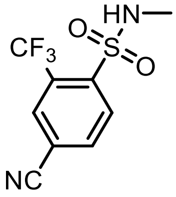

Into an oven dried 8 mL vial containing a magnetic stir bar were added 4-bromo-*N*-methyl-2-(trifluoromethyl)-benzenesulfonamide (1.27 g, 4.00 mmol) and CuCN (1.43 g, 16.0 mmol) followed by 4 mL anhydrous DMF. The vial was capped and heated to 150 °C for 24 h. After cooling to 20 – 25 °C, the mixture was diluted with 100 mL EtOAc and the mixture washed with aqueous NH_4OH solution (5%, 3 x 30 mL) and brine (30 mL). The organic phase was dried over $MgSO_4$, filtered and the solvent was removed under reduced pressure. Purification by column chromatography on silica (10 – 40% EtOAc in heptane), afforded compound **0o** in 54% (568 mg) yield as a white solid.

^{1}H NMR (400 MHz, CDCl$_3$): δ 8.37 (d, *J* = 8.2 Hz, 1H), 8.15 (s, 1H), 8.02 (d, *J* = 8.2 Hz, 1H), 4.75 (d, *J* = 3.7 Hz, 1H), 2.74 (d, *J* = 5.0 Hz, 3H) ppm.

^{13}C NMR (100 MHz, CDCl$_3$): δ 142.3, 136.0, 132.9, 132.1 (q, *J* = 6.4 Hz), 129.2 (d, *J* = 34.2 Hz), 123.4 (q, *J* = 274.7 Hz), 117.1, 116.2, 29.5 ppm.

^{19}F NMR (377 MHz, CDCl$_3$): δ -58.6 ppm.

IR (neat) ν_{max}: 3339, 2237, 1475, 1406, 1335, 1197, 1150, 1073, 1039, 595 cm^{-1}.

HRMS (ESI$^+$): exact mass calculated for [M+Na]$^+$ ($C_9H_7F_3N_2NaO_2S^+$) requires *m/z* 287.0073, found *m/z* 287.0063.

Sulfonyl carboximides

1a: *N*-Methyl-*N*-((4-nitrophenyl)sulfonyl)acrylamide

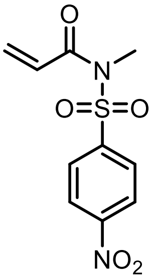

Following General Procedure B using *N*-Methyl-*N*-((4-nitrophenyl)sulfonyl) amide (432 mg, 2.00 mmol), the purification by column chromatography on silica (5 – 20% EtOAc in heptane) afforded compound **1a** in 46% yield (250 mg) as a white solid.

^{1}H NMR (400 MHz, CDCl$_3$): δ 8.39 (d, *J* = 8.8 Hz, 2H), 8.11 (d, *J* = 8.8 Hz, 2H), 6.91 (dd, *J* = 17.0, 10.4 Hz, 1H), 6.43 (dd, *J* = 17.0, 1.2 Hz, 1H), 5.88 (dd, *J* = 10.4, 1.2 Hz, 1H), 3.38 (s, 3H) ppm.

^{13}C NMR (100 MHz, CDCl$_3$): δ 165.9, 150.8, 144.6, 132.9, 129.2 (2C), 127.9, 124.6 (2C), 33.5 ppm.

IR (neat) ν_{max}: 1698, 1528, 1348, 1178, 1079, 842, 750, 743, 710, 678, 631.

HRMS (ESI$^+$): exact mass calculated for [M+Na]$^+$ ($C_{10}H_{10}N_2NaO_5S^+$) requires *m/z* 293.0203, found *m/z* 293.0201.

1b: *N*-Isopropyl-*N*-((4-nitrophenyl)sulfonyl)acrylamide

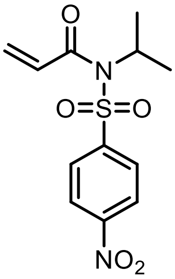

Following General Procedure B using *N*-isopropyl-*N*-((4-nitrophenyl)sulfonyl)acrylamide (1.22 g, 5.00 mmol), the purification by column chromatography on silica (20 – 80% EtOAc in heptane) afforded compound **1b** in 46% yield (692 mg) as a white solid.

^{1}H NMR (400 MHz, $CDCl_3$): δ 8.39 (d, *J* = 9.0 Hz, 2H), 8.14 (d, *J* = 9.0 Hz, 2H), 6.74 (dd, *J* = 16.7, 10.4 Hz, 1H), 6.36 (dd, *J* = 16.7, 1.4 Hz, 1H), 5.80 (dd, *J* = 10.4, 1.4 Hz, 1H), 4.55 (m, 1H), 1.52 (d, *J* = 6.9 Hz, 6H) ppm.

^{13}C NMR (100 MHz, $CDCl_3$): δ 166.3, 150.5, 146.0, 131.6, 129.7, 129.1 (2C), 124.5 (2C), 53.5, 21.5 (2C) ppm.

IR (neat) ν_{max}: 3110, 2349, 1678, 1530, 975, 855, 576 cm^{-1}.

HRMS (ESI$^+$): exact mass calculated for $[M+Na]^+$ ($C_{12}H_{14}N_2NaO_5S^+$) requires m/z 321.0516, found m/z 321.0509.

1c: *N*-(*tert*-Butyl)-*N*-((4-nitrophenyl)sulfonyl)acrylamide

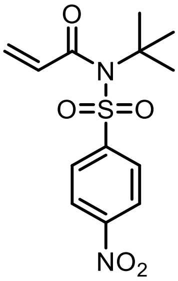

Following General Procedure B using *N*-(*tert*-butyl)-4-nitrobenzenesulfonamide (1.29 g, 5.00 mmol), the purification by column chromatography on silica (5 – 30% EtOAc in heptane) afforded compound **1c** in 33% yield (515 mg) as a white solid.

^{1}H NMR (400 MHz, $CDCl_3$): δ 8.37 (d, *J* = 8.5 Hz, 2H), 8.17 (d, *J* = 8.5 Hz, 2H), 6.58 (dd, *J* = 16.9, 9.9 Hz, 1H), 6.48 (d, *J* = 16.8 Hz, 1H), 6.00 (d, *J* = 9.8 Hz, 1H), 1.42 (s, 9H) ppm.

^{13}C NMR (100 MHz, $CDCl_3$): δ 170.6, 150.2, 148.4, 134.7, 132.9, 129.1 (2C), 124.4 (2C), 62.6, 30.1 (3C) ppm.

IR (neat) ν_{max}: 1704, 1530, 1347, 1156, 1086, 979, 737, 630 cm^{-1}.

HRMS (ESI$^+$): exact mass calculated for $[M+Na]^+$ ($C_{13}H_{16}N_2NaO_5S^+$) requires *m/z* 335.0672, found *m/z* 335.0671.

1d: *N*-Allyl-*N*-((4-nitrophenyl)sulfonyl)acrylamide

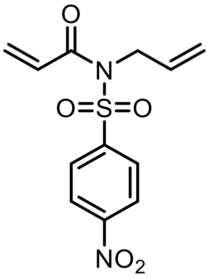

Following General Procedure B using *N*-allyl-4-nitrobenzenesulfonamide (1.21 g, 5.00 mmol), the purification by column chromatography on silica (5 – 40% EtOAc in heptane) afforded compound **1d** in 64% yield (945 mg) as a white solid.

^{1}H NMR (400 MHz, CDCl$_3$): δ 8.43 – 8.34 (m, 2H), 8.24 – 8.13 (m, 2H), 6.64 (dd, *J* = 16.7, 10.3 Hz, 1H), 6.43 (dd, *J* = 16.7, 1.4 Hz, 1H), 5.99 – 5.81 (m, 2H), 5.33 (dd, *J* = 13.7, 5.0 Hz, 2H), 4.55 (dt, *J* = 5.1, 1.5 Hz, 2H) ppm.

^{13}C NMR (100 MHz, CDCl$_3$): δ 165.6, 150.7, 145.1, 133.4, 132.3, 130.0 (2C), 127.4, 124.3 (2C), 119.1, 48.9 ppm.

IR (neat) ν_{max}: 3108, 1688, 1527, 1346, 1166, 743, 605 cm^{-1}.

HRMS (ESI$^+$): exact mass calculated for [M+Na]$^+$ ($C_{12}H_{12}N_2NaO_5S^+$) requires *m/z* 319.0359, found *m/z* 319.0347.

1e: *N*-Benzyl-*N*-((4-nitrophenyl)sulfonyl)acrylamide

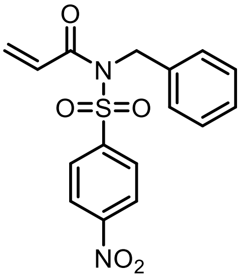

Following General Procedure B using *N*-benzyl-4-nitrobenzenesulfonamide (1.14 g, 3.90 mmol), the purification by column chromatography on silica (10 – 25% EtOAc in heptane) afforded compound **1e** in 41% yield (548 mg) as a white solid.

^{1}H NMR (400 MHz, CDCl$_3$): δ 8.27 (d, *J* = 8.9 Hz, 2H), 7.93 (d, *J* = 8.9 Hz, 2H), 7.42 – 7.28 (m, 5H), 6.72 (dd, *J* = 16.6, 10.4 Hz, 1H), 6.44 (d, *J* = 16.6 Hz, 1H), 5.83 (d, *J* = 10.4 Hz, 1H), 5.16 (s, 2H) ppm.

^{13}C NMR (100 MHz, CDCl$_3$): δ 165.9, 150.6, 145.1, 135.8, 133.5, 129.7 (C2), 129.1 (2C), 128.4, 127.74 (2C), 127.67 (2C), 124.2, 49.9 ppm.

IR (neat) ν_{max}: 1692, 1529, 1348, 1163, 1126, 974, 854, 736 cm^{-1}.

HRMS (ESI$^+$): exact mass calculated for [M+Na]$^+$ ($C_{16}H_{14}N_2NaO_5S^+$) requires m/z 369.0516, found m/z 369.0518.

1f: *N*-(4,4-Dimethoxybutyl)-*N*-((4-nitrophenyl)sulfonyl)acrylamide

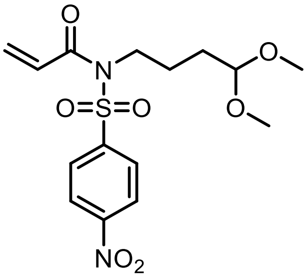

Following General Procedure B using **0f** (1.59 g, 5.00 mmol), the purification by column chromatography on silica (5 – 50% EtOAc in heptane) afforded compound **1f** in 48% yield (886 mg) as a white solid.

^{1}H NMR (400 MHz, $CDCl_3$): δ 8.37 (d, *J* = 8.7 Hz, 2H), 8.14 (d, *J* = 8.7 Hz, 2H), 6.75 (dd, *J* = 16.6, 10.4 Hz, 1H), 6.43 (dd, *J* = 16.6, 1.0 Hz, 1H), 5.86 (dd, *J* = 10.4, 1.0 Hz, 1H), 4.40 (t, *J* = 5.4 Hz, 1H), 3.95 – 3.85 (m, 2H), 3.35 (s, 6H), 1.84 (m, 2H), 1.69 (m, 2H) ppm.

^{13}C NMR (100 MHz, $CDCl_3$): δ 165.5, 150.7, 145.2, 133.2, 129.6 (2C), 127.5, 124.4 (2C), 104.3, 53.4 (2C), 46.9, 29.7, 25.7 ppm.

IR (neat) ν_{max}: 2939, 1687, 1532, 1367, 1164, 742 cm^{-1}.

HRMS (ESI$^+$): exact mass calculated for $[M+Na]^+$ ($C_{15}H_{20}N_2NaO_7S^+$) requires *m/z* 395.0883, found *m/z* 395.0877.

1g: Methyl *N*-acryloyl-*N*-((4-nitrophenyl)sulfonyl)glycinate

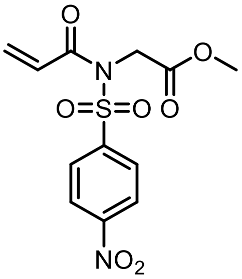

Following General Procedure B using ((4-nitrophenyl)sulfonyl)glycinate (411 mg, 1.50 mmol), the purification by column chromatography on silica (20 – 45% EtOAc in heptane) afforded compound **1g** in 54% yield (263 mg) as a white solid.

^{1}H NMR (400 MHz, $CDCl_3$): δ 8.40 (d, *J* = 8.8 Hz, 2H), 8.29 (d, *J* = 8.8 Hz, 2H), 6.71 (dd, *J* = 16.6, 10.4 Hz, 1H), 6.42 (d, *J* = 16.6 Hz, 1H), 5.85 (d, *J* = 11.3 Hz, 1H), 4.69 (s, 2H), 3.80 (s, 3H) ppm.

^{13}C NMR (100 MHz, $CDCl_3$): δ 168.6, 165.0, 150.9, 144.7, 133.8, 130.0 (2C), 126.9, 124.4 (2C), 53.1, 46.8 ppm.

IR (neat) ν_{max}: 1744, 1530, 1352, 1165, 1138, 1085, 979, 556 cm^{-1}.

HRMS (ESI$^+$): exact mass calculated for $[M+Na]^+$ ($C_{12}H_{12}N_2NaO_7S^+$) requires m/z 351.0257, found m/z 351.0261.

1h: Methyl *N*-acryloyl-*N*-((4-nitrophenyl)sulfonyl)methioninate

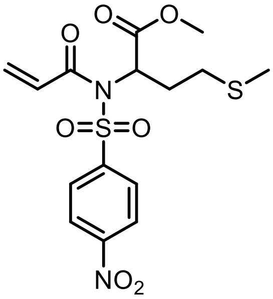

Following General Procedure B using methyl-((4-nitrophenyl)sulfonyl)methioninate (1.05 g, 3.00 mmol), the purification by column chromatography on silica (0 – 40% EtOAc in heptane) afforded compound **1h** in 80% yield (964 mg) as a yellow oil.

^{1}H NMR (400 MHz, $CDCl_3$): δ 8.39 (d, *J* = 8.9 Hz, 2H), 8.29 (d, *J* = 8.9 Hz, 2H), 6.84 (dd, *J* = 16.6, 10.5 Hz, 1H), 6.36 (dd, *J* = 16.6, 1.0 Hz, 1H), 5.81 (dd, *J* = 10.5, 1.0 Hz, 1H), 5.22 (dd, *J* = 8.1, 4.8 Hz, 1H), 3.70 (s, 3H), 2.77 – 2.56 (m, 3H), 2.32 – 2.17 (m, 1H), 2.13 (s, 3H) ppm.

^{13}C NMR (100 MHz, $CDCl_3$): δ 169.7, 165.0, 150.8, 144.9, 133.5, 129.9 (2C), 127.7, 124.4 (2C), 59.1, 53.0, 31.2, 29.6, 15.4 ppm.

IR (neat) ν_{max}: 1741, 1687, 1531, 1347, 1157, 742, 610, 576 cm^{-1}.

HRMS (ESI$^+$): exact mass calculated for $[M+Na]^+$ ($C_{15}H_{18}N_2NaO_7S_2^+$) requires *m/z* 425.0448, found *m/z* 425.0449.

1i: *N*-(3,4-Dimethoxyphenethyl)-*N*-((4-nitrophenyl)sulfonyl)acrylamide

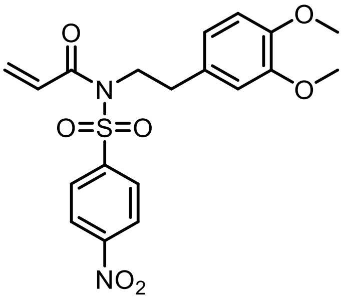

Following General Procedure B using *N*-(3,4-dimethoxyphenethyl)-4-nitrobenzenesulfonamide (1.10 g, 3.00 mmol), the purification by column chromatography on silica (5 – 50% EtOAc in heptane) afforded compound **1i** in 22% yield (283 mg) as a yellow solid.

^{1}H NMR (400 MHz, $CDCl_3$): δ 8.31 (d, *J* = 8.8 Hz, 2H), 8.06 (d, *J* = 8.8 Hz, 2H), 6.82 – 6.70 (m, 3H), 6.60 (dd, *J* = 16.6, 10.4 Hz, 1H), 6.34 (dd, *J* = 16.6, 0.8 Hz, 1H), 5.77 (dd, *J* = 10.4, 0.8 Hz, 1H), 4.10 – 4.00 (m, 2H), 3.84 (s, 3H), 3.84 (s, 3H), 3.05 – 2.93 (m, 2H) ppm.

^{13}C NMR (100 MHz, $CDCl_3$): δ 165.6, 150.5, 149.2, 148.2, 145.0, 132.6, 129.8, 129.4 (2C), 127.3, 124.3 (2C), 121.1, 112.3, 111.5, 56.0 (2C), 48.7, 36.3 ppm.

IR (neat) ν_{max}: 1687, 1533, 1514, 1349, 1161, 1028, 742, 618 cm^{-1}.

HRMS (ESI$^+$): exact mass calculated for $[M+Na]^+$ ($C_{19}H_{20}N_2NaO_7S^+$) requires *m/z* 443.0883, found *m/z* 443.0866.

1j: *tert*-Butyl 3-(2-(*N*-((4-nitrophenyl)sulfonyl)acrylamido)ethyl)-1*H*-indole-1-carboxylate

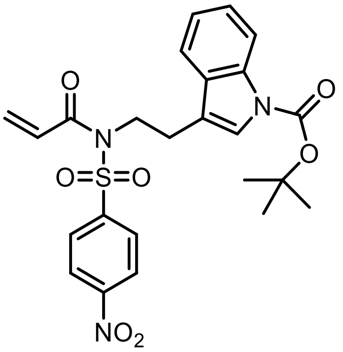

Following General Procedure B using **0j** (445 mg, 1.00 mmol), the purification by column chromatography on silica (5 – 80% EtOAc in heptane) afforded compound **1j** in 60% yield (300 mg) as a yellow solid.

^{1}H NMR (400 MHz, CDCl$_3$): δ 8.28 (d, *J* = 8.8 Hz, 2H), 8.09 (d, *J* = 7.4 Hz, 1H), 8.04 (d, *J* = 8.8 Hz, 2H), 7.63 (d, *J* = 7.6 Hz, 1H), 7.41 (s, 1H), 7.36 – 7.22 (m, 2H), 6.83 (dd, *J* = 16.6, 10.4 Hz, 1H), 6.47 (d, *J* = 16.6 Hz, 1H), 5.87 (d, *J* = 10.4 Hz, 1H), 4.20 – 4.05 (m, 2H), 3.22 – 3.10 (m, 2H), 1.68 (s, 9H) ppm.

^{13}C NMR (100 MHz, CDCl$_3$): δ 165.5, 150.6, 149.7, 144.9, 135.6, 133.0, 130.1, 129.2 (2C), 127.8, 124.9, 124.3 (2C), 123.9, 122.9, 119.0, 116.2, 115.5, 84.0, 47.2, 28.3 (3C), 26.0 ppm.

IR (neat) ν_{max}: 2980, 1729, 1688, 1532, 1370, 1154, 742 cm^{-1}.

HRMS (ESI$^+$): exact mass calculated for $[M+Na]^+$ ($C_{24}H_{25}N_3NaO_7S^+$) requires *m/z* 522.1305, found *m/z* 522.1305.

1k: Ethyl (*E*)-6-(*N*-((4-nitrophenyl)sulfonyl)acrylamido)hex-2-enoate

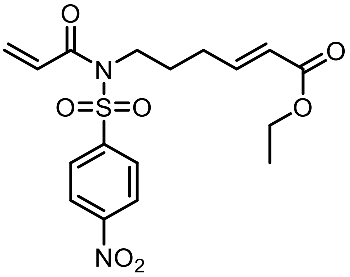

Following General Procedure B using **0k** (719 mg, 2.10 mmol), the purification by column chromatography on silica (10 – 30% EtOAc in heptane) afforded compound **1k** in 73% yield (610 mg) as a white solid.

^{1}H NMR (400 MHz, CDCl$_3$): δ 8.37 (d, *J* = 8.8 Hz, 2H), 8.10 (d, *J* = 8.7 Hz, 2H), 6.98 – 6.88 (m, 1H), 6.78 (dd, *J* = 16.6, 10.4 Hz, 1H), 6.42 (d, *J* = 16.6 Hz, 1H), 5.92 – 5.82 (m, 2H), 4.18 (q, *J* = 7.1 Hz, 2H), 3.88 – 3.81 (m, 2H), 2.30 (app. q, *J* = 7.2 Hz, 2H), 1.98 – 1.88 (m, 2H), 1.28 (t, *J* = 7.1 Hz, 3H) ppm.

^{13}C NMR (100 MHz, CDCl$_3$): δ 166.4, 165.4, 150.7, 146.6, 145.0, 133.3, 129.3 (2C), 127.6, 124.5 (2C), 122.7, 60.5, 46.5, 29.3, 28.6, 14.4 ppm.

IR (neat) ν_{max}: 2924, 1713, 1686, 1530, 1348, 1157, 977, 854, 741, 576 cm^{-1}.

HRMS (ESI$^+$): exact mass calculated for $[M+Na]^+$ ($C_{17}H_{20}N_2NaO_7S^+$) requires *m/z* 419.0883, found *m/z* 419.0870.

1l: *N*-Methyl-*N*-((2-nitrophenyl)sulfonyl)acrylamide

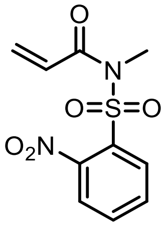

Following General Procedure B using *N*-methyl-2-nitrobenzenesulfonamide (108 mg, 0.50 mmol). Purification by column chromatography on silica (10 – 25% EtOAc in heptane), afforded compound **1k** in 50% yield (66.9 mg) as a white solid.

^{1}H NMR (400 MHz, CDCl$_3$): δ 8.46 – 8.34 (m, 1H), 7.78 (m, 3H), 6.68 (dd, *J* = 16.7, 10.4 Hz, 1H), 6.43 (dd, *J* = 16.7, 1.5 Hz, 1H), 5.87 (dd, *J* = 10.4, 1.5 Hz, 1H), 3.44 (s, 3H) ppm.

^{13}C NMR (100 MHz, CDCl$_3$): δ 165.8, 148.1, 134.8, 134.4, 132.9, 132.8, 132.3, 127.5, 124.8, 33.7 ppm.

IR (neat) ν_{max}: 1693, 1541, 1404, 1362, 1169, 1093, 715, 601 cm^{-1}.

HRMS (ESI$^+$): exact mass calculated for [M+Na]$^+$ ($C_{10}H_{10}N_2NaO_5S^+$) requires m/z 293.0203, found m/z 293.0200.

1m: *N*-((2-Methoxy-4-nitrophenyl)sulfonyl)-*N*-methylacrylamide

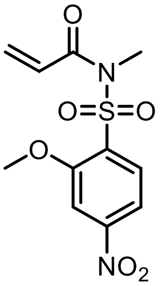

Following General Procedure B using 2-methoxy-*N*-methyl-4-nitrobenzenesulfonamide (492 mg, 2.00 mmol), the purification by column chromatography on silica (0 – 30% EtOAc in heptane) afforded compound **1k** in 23% yield (137 mg) as a white solid.

^{1}H NMR (400 MHz, CDCl$_3$): δ 8.22 (d, *J* = 8.6 Hz, 1H), 7.96 (dd, *J* = 8.6, 1.9 Hz, 1H), 7.85 (d, *J* = 2.0 Hz, 1H), 7.00 (dd, *J* = 16.7, 10.5 Hz, 1H), 6.41 (dd, *J* = 16.8, 1.4 Hz, 1H), 5.80 (dd, *J* = 10.4, 1.4 Hz, 1H), 4.00 (s, 3H), 3.27 (s, 3H) ppm.

^{13}C NMR (100 MHz, CDCl$_3$): δ 166.3, 157.4, 152.3, 132.6, 132.2, 131.3, 128.8, 115.5, 107.8, 57.1, 33.3 ppm.

IR (neat) ν_{max}: 3105, 1693, 1530, 1347, 1201, 1154, 733, 607 cm^{-1}.

HRMS (ESI$^+$): exact mass calculated for [M+Na]$^+$ ($C_{11}H_{12}N_2NaO_6S^+$) requires *m/z* 323.0314, found *m/z* 323.0308

1n: *N*-((2,5-bis(trifluoromethyl)phenyl)sulfonyl)-N-methylacrylamide

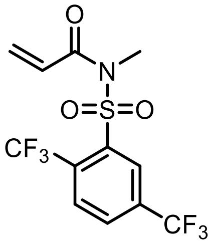

Following General Procedure B using **0n** (307 mg, 1.00 mmol), the purification by column chromatography on silica (5 – 20 % EtOAc in heptane) afforded compound **1n** in 67% yield (241 mg) as a white solid.

^{1}H NMR (400 MHz, CDCl$_3$): δ 8.71 (s, 1H), 8.01 (s, 2H), 6.65 (dd, J = 16.7, 10.4 Hz, 1H), 6.42 (d, J = 16.7 Hz, 1H), 5.87 (d, J = 10.4 Hz, 1H), 3.44 (s, 3H) ppm.

^{13}C NMR (176 MHz, CDCl$_3$): δ 165.8, 139.8, 134.9 (q, *J* = 33.7 Hz, 2C), 133.1, 131.2 (q, *J* = 3.7 Hz), 130.6 (q, *J* = 3.4 Hz), 129.3 (q, *J* = 6.3 Hz), 127.2, 122.5 (q, *J* = 274.7 Hz), 122.0 (q, *J* = 274.4 Hz) 33.3 ppm.

^{19}F NMR (377 MHz, CDCl$_3$): δ -58.8, -63.3 ppm.

IR (neat) ν_{max}: 1697, 1405, 1365, 1331, 1309, 1269, 1133, 1034, 730 cm^{-1}.

HRMS (ESI$^+$): exact mass calculated for [M+Na]$^+$ ($C_{12}H_9F_6NNaO_3S^+$) requires *m/z* 384.0100, found *m/z* 384.0103.

1o: *N*-((4-Cyano-2-(trifluoromethyl)phenyl)sulfonyl)-*N*-methylacrylamide

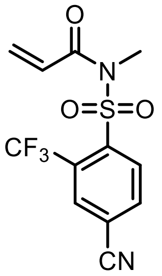

Following General Procedure B using **0o** (137 mg, 0.52 mmol), the purification by column chromatography on silica (0 – 40% EtOAc in heptane) afforded compound **1o** in 66% yield (108 mg) as a white solid.

^{1}H NMR (400 MHz, CDCl$_3$): δ 8.60 (d, *J* = 8.3 Hz, 1H), 8.15 – 8.03 (m, 2H), 6.60 (dd, *J* = 16.6, 10.3 Hz, 1H), 6.42 (d, *J* = 16.6 Hz, 1H), 5.89 (d, *J* = 10.3 Hz, 1H), 3.47 (s, 3H) ppm.

^{13}C NMR (176 MHz, CDCl$_3$): δ 165.8, 142.3, 135.8, 135.1, 133.7, 131.7 (q, *J* = 6.4 Hz), 128.9 (q, *J* = 34.7 Hz), 126.8, 121.6 (q, *J* = 274.7 Hz), 117.9, 116.0, 33.2 (app.m) ppm.

^{19}F NMR (659 MHz, CDCl$_3$): δ -58.8 ppm.

IR (neat) ν_{max}: 2922, 2852, 2239, 1685, 1618, 1357, 1312, 1269, 1163, 1089, 742, 654, 567 cm^{-1}.

HRMS (ESI$^+$): exact mass calculated for [M+Na]$^+$ ($C_{12}H_9F_3N_2NaO_3S^+$) requires *m/z* 341.0178, found *m/z* 341.0179.

SI1: *N*-methyl-*N*-(pyridin-2-ylsulfonyl)acrylamide

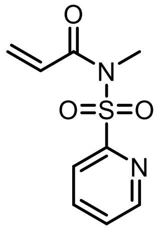

Following General Procedure C using **0p** (1033 mg, 6.00 mmol), the purification by column chromatography on silica (0 – 50% EtOAc in heptane) afforded compound **SI1** in 48% yield (655 mg) as a pale yellow oil.

^{1}H NMR (600 MHz, $CDCl_3$): δ 8.68 (d, *J* = 4.5 Hz, 1H), 8.08 (d, *J* = 7.8 Hz, 1H), 7.96 (td, *J* = 7.8, 1.6 Hz, 1H), 7.55 (ddd, *J* = 7.8, 4.5, 0.7 Hz, 1H), 7.10 (dd, *J* = 16.7, 10.5 Hz, 1H), 6.37 (dd, *J* = 16.7, 1.6 Hz, 1H), 5.77 (dd, *J* = 10.5, 1.6 Hz, 1H), 3.38 (s, 3H).

^{13}C NMR (151 MHz, $CDCl_3$): δ 166.7, 156.6, 150.5, 138.3, 131.0, 129.0, 127.7, 122.8, 33.8.

IR (neat) ν_{max}:1687, 1616, 1579, 1402, 1355,1173, 1015, 909, 789,623, 575 cm^{-1}.

HRMS (ESI$^+$): exact mass calculated for $[M+Na]^+$ ($C_9H_{10}N_2NaO_3S^+$) requires m/z 249.0305, found m/z 249.0301.

1p: 2-(*N*-acryloyl-*N*-methylsulfamoyl)pyridine 1-oxide

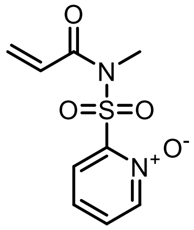

This procedure was inspired by Caron *et al.*[10] To a solution of **SI1** (453 mg, 2.00 mmol, 1.0 equiv.) and urea hydrogen peroxide (395 mg, 4.20 mmol, 2.1 equiv.) in dry DCM (20 mL) in a round-bottom flask at 0 °C with stirring was added trifluoroacetic anhydride (0.56 mL, 4.0 mmol, 2.0 equiv.) dropwise. The reaction mixture was allowed to warm to 25 °C and stirred for 16 h, upon which saturated aqueous $Na_2S_2O_3$ solution (10 mL) was added to quench the excess peroxide. The mixture was stirred for further 15 mins and poured into aqueous 1M HCl solution (25 mL). The phases were separated and the aqueous phase was extracted with DCM (2 x 25 mL). The combined organic phase was washed with saturated aqueous $NaHCO_3$ solution (25 mL), dried over Na_2SO_4, filtered and the solvent was removed under reduced pressure. Purification by column chromatography on silica (50 – 100% EtOAc in heptane) afforded compound **1p** as an off-white solid (113 mg, 23 %).

^{1}H NMR (600 MHz, $CDCl_3$): δ 8.18 (app. Dd, *J* = 11.7, 4.0 Hz, 2H), 7.51 – 7.46 (m, 1H), 7.43 (td, *J* = 7.9, 0.9 Hz, 1H), 6.84 (dd, *J* = 16.8, 10.5 Hz, 1H), 6.41 (dd, *J* = 16.8, 1.3 Hz, 1H), 5.83 (dd, *J* = 10.5, 1.3 Hz, 1H), 3.45 (d, *J* = 6.3 Hz, 3H).

^{13}C NMR (151 MHz, $CDCl_3$): δ 166.5, 146.1, 141.2, 131.9, 129.6, 129.4, 128.2, 125.2, 34.3.

IR (neat) ν_{max}: 3109, 1689, 1617, 1593, 1425, 1359, 1150, 848, 733, 557 cm^{-1}.

---

[10] S. Caron, N. M Do, J. E. Sieser, *Tet. Lett.* **2000**, *41*, 2299-2302.

HRMS (ESI$^+$): exact mass calculated for $[M+Na]^+$ ($C_9H_{10}N_2NaO_4S^+$) requires *m/z* 265.0254, found *m/z* 265.0250.

1o: (*E*)-*N*-Methyl-*N*-((4-nitrophenyl)sulfonyl)but-2-enamide

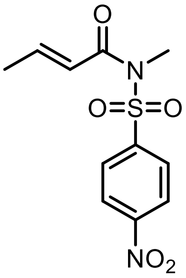

Following General Procedure C using *N*-methyl-4-nitrobenzenesulfonamide (649 mg, 3.00 mmol) and (*E*)-2-butenoyl chloride (383 µL, 3.60 mmol) the purification by column chromatography on silica (5 – 35% EtOAc in heptane) afforded compound **1p** in 80% yield (679 mg) as a white solid.

^{1}H NMR (400 MHz, CDCl$_3$): δ 8.41 – 8.35 (m, 2H), 8.15 – 8.07 (m, 2H), 7.03 (dq, *J* = 13.9, 7.0 Hz, 1H), 6.58 (dq, *J* = 15.0, 1.6 Hz, 1H), 3.37 (s, 3H), 1.93 (dd, *J* = 7.0, 1.7 Hz, 3H) ppm.

^{13}C NMR (100 MHz, CDCl$_3$): δ 166.0, 150.7, 148.2, 144.9, 129.2 (2C), 124.5 (2C), 122.1, 33.5, 18.8 ppm.

IR (neat) ν_{max}: 1684, 1634, 1527, 1347, 1158, 1081, 938, 735, 617, 582 cm^{-1}.

HRMS (ESI$^+$): exact mass calculated for $[M+Na]^+$ ($C_{11}H_{12}N_2NaO_5S^+$) requires *m/z* 307.0359, found *m/z* 307.0353.

1r: (*E*)-*N*-Methyl-*N*-((4-nitrophenyl)sulfonyl)oct-2-enamide

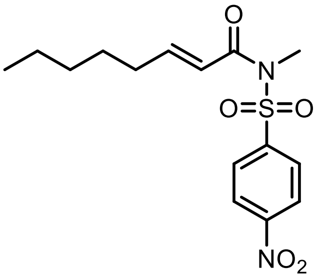

Following General Procedure C using *N*-methyl-4-nitrobenzenesulfonamide (1.08 g, 5.00 mmol) and *trans*-2-octenoic acid chloride (6.00 mmol; generated according to a reported procedure[11]), the purification by column chromatography on silica (5% EtOAc in heptane) afforded compound **1q** in 12% yield (205 mg) as a colourless oil.

^{1}H NMR (400 MHz, CDCl$_3$): δ 8.38 (d, *J* = 8.8 Hz, 2H), 8.11 (d, *J* = 8.8 Hz, 2H), 7.03 (dt, *J* = 15.1, 7.0 Hz, 1H), 6.54 (d, *J* = 15.1 Hz, 1H), 3.38 (s, 3H), 2.29 – 2.16 (m, 2H), 1.51 – 1.38 (m, 2H), 1.36 – 1.22 (m, 4H), 0.88 (t, *J* = 6.8 Hz, 3H) ppm.

^{13}C NMR (100 MHz, CDCl$_3$): δ 166.1, 153.3, 150.7, 144.9, 129.2 (2C), 124.5 (2C), 120.5, 33.5, 32.9, 31.4, 27.7, 22.5, 14.1 ppm.

IR (neat) ν_{max}: 2958, 2929, 2857, 1687, 1634, 1532, 1349, 1178, 740 cm^{-1}.

[11] K. Biswas, S. Woodward, *Tetrahedron: Asymmetry* **2008**, *19*, 1702–1708.

HRMS (ESI$^+$): exact mass calculated for $[M+Na]^+$ ($C_{15}H_{20}N_2NaO_5S^+$) requires *m/z* 363.0985, found *m/z* 363.0978.

1s: *N*,3-Dimethyl-*N*-((4-nitrophenyl)sulfonyl)but-2-enamide

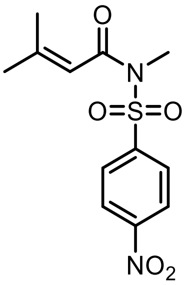

Following General Procedure C using *N*-methyl-4-nitrobenzenesulfonamide (649 mg, 3.00 mmol) and 3-methylcrotonic acid chloride (601 mg, 6.00 mmol; generated by a reported method[12]), the purification by column chromatography on silica (0 – 30% EtOAc in heptane) afforded compound **1r** in 20% yield (181 mg) as a white solid.

^{1}H NMR (400 MHz, $CDCl_3$): δ 8.41 – 8.33 (m, *J* = 9.1, 2.1 Hz, 2H), 8.15 – 8.07 (m, 2H), 6.21 – 6.11 (m, 1H), 3.37 (s, 3H), 1.97 (d, *J* = 1.0 Hz, 3H), 1.92 (d, *J* = 1.0 Hz, 3H) ppm.

^{13}C NMR (100 MHz, $CDCl_3$): δ 166.3, 158.8, 150.6, 145.2, 129.2 (2C), 124.3 (2C), 116.5, 33.6, 27.8, 21.1 ppm.

IR (neat) ν_{max}: 1688, 1531, 1349, 1158, 739, 620, 583 cm^{-1}.

HRMS (ESI$^+$): exact mass calculated for $[M+Na]^+$ ($C_{12}H_{14}N_2NaO_5S^+$) requires *m/z* 321.0516, found *m/z* 321.0515.

1t: (*E*)-*N*,4-Dimethyl-*N*-((4-nitrophenyl)sulfonyl)pent-2-enamide

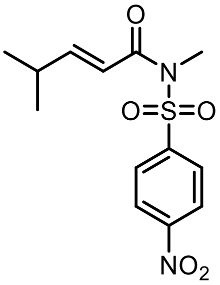

Following General Procedure C using *N*-methyl-4-nitrobenzenesulfonamide (2.16 g, 10.0 mmol) and 4-methyl-2-pentenoic acid chloride (generated by a reported method[13] from 1.31 mL, 11.0 mmol 4-methyl-2-pentenoic acid), the purification by column chromatography on silica (5 – 15% EtOAc in heptane), afforded compound **1s** in 94% yield (2.95 g) as an off-white solid.

^{1}H NMR (400 MHz, $CDCl_3$): δ 8.42 – 8.34 (m, 2H), 8.14 – 8.08 (m, 2H), 6.99 (dd, *J* = 15.2, 6.8 Hz, 1H), 6.48 (dd, *J* = 15.2, 1.3 Hz, 1H), 3.39 (s, 3H), 2.54 – 2.43 (m, 1H), 1.05 (d, *J* = 6.8 Hz, 6H) ppm.

^{13}C NMR (100 MHz, $CDCl_3$): δ 166.4, 158.9, 150.7, 145.0, 129.3 (2C), 124.5, 118.0 (2C), 33.5, 31.7, 21.3 (2C) ppm.

---

[12] A. R. Katritzky, N. K. Meher, S. K. Singh, *J. Org. Chem.* **2005**, 70, 7792–7794.
[13] G. S. Reddy, *ChemRxiv* **2020**, 10.26434/chemrxiv.13298210.v1.

IR (neat) ν_{max}: 3108, 2963, 2871, 1685, 1632, 1531, 1348, 1161, 740 cm^{-1}.

HRMS (ESI$^+$): exact mass calculated for $[M+Na]^+$ ($C_{13}H_{16}N_2NaO_5S^+$) requires *m/z* 335.0672, found *m/z* 335.0673.

1u: (2*E*,4*E*)-*N*-Methyl-*N*-((4-nitrophenyl)sulfonyl)hexa-2,4-dienamide

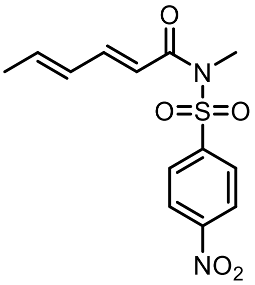

Following General Procedure C using *N*-methyl-4-nitrobenzenesulfonamide (2.16 g, 10.0 mmol) and sorbic acid chloride (generated by a reported method[14] from 1.65 g, 11.0 mmol potassium sorbate), the purification by column chromatography on silica (10% EtOAc in heptane) afforded compound **1t** in 56% yield (1.74 g) as a yellow solid.

^{1}H NMR (400 MHz, $CDCl_3$): δ 8.43 – 8.32 (m, 2H), 8.15 – 8.05 (m, 2H), 7.36 – 7.27 (m, 1H), 6.52 (d, *J* = 14.7 Hz, 1H), 6.31 – 6.17 (m, 2H), 3.38 (s, 3H), 1.92 – 1.85 (d, *J* = 5.2 Hz, 3H) ppm.

^{13}C NMR (100 MHz, $CDCl_3$): δ 166.5, 150.6, 148.2, 144.9, 142.7, 130.0, 129.2 (2C), 124.5 (2C), 117.7, 33.5, 19.0 ppm.

IR (neat) ν_{max}: 3107, 1678, 1634, 1602, 1529, 1348, 1169, 1072, 1000, 737 cm^{-1}.

HRMS (ESI$^+$): exact mass calculated for $[M+Na]^+$ ($C_{13}H_{14}N_2NaO_5S^+$) requires *m/z* 333.0516, found *m/z* 333.0513.

SI2: *N*-((4-nitrophenyl)sulfonyl)-*N*-phenylacrylamide

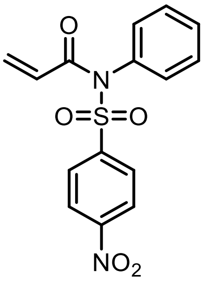

Following General Procedure B using 4-nitro-*N*-phenylbenzenesulfonamide (1.39 g, 5.00 mmol), the purification by flash column chromatography (10–25% EtOAc in heptane) gave compound **SI2** in 94% yield (1.56 g) as a yellow powder.

^{1}H NMR (400 MHz, $CDCl_3$): δ 8.40 (d, *J* = 9.0 Hz, 2H), 8.25 (d, *J* = 9.0 Hz, 2H), 7.66 – 7.45 (m, 3H), 7.27 (d, *J* = 1.5 Hz, 1H), 7.25 (d, *J* = 1.9 Hz, 1H), 6.40 (dd, *J* = 16.7, 1.5 Hz, 1H), 5.78 (dd, *J* = 16.7, 10.4 Hz, 1H), 5.65 (dd, *J* = 10.4, 1.5 Hz, 1H) ppm.

^{13}C NMR (100 MHz, $CDCl_3$): δ 165.1, 150.9, 144.5, 135.1, 132.7, 130.8 (2C), 130.7, 130.29 (2C), 130.26 (2C), 127.8, 124.1 (2C) ppm.

[14] A. Sib, R. A. M. Gulder, *Angew. Chem. Int. Ed.* **2018**, *57*, 14650–14653.

IR (neat) ν_{max}: 1656, 1485, 1369, 1349, 1152, 1025, 742, 570 cm^{-1}.

HRMS (ESI^+): exact mass calculated for $[M+Na]^+$ ($C_{15}H_{12}N_2NaO_5S^+$) requires m/z 355.0359, found m/z 355.0352.

SI3: *N*-((4-Cyanophenyl)sulfonyl)-*N*-methylacrylamide

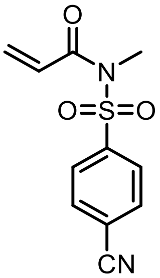

Following General Procedure B using 4-cyano-*N*-methylbenzenesulfonamide (589 mg, 3.00 mmol), the purification by column chromatography on silica (5 – 40% EtOAc in heptane) afforded compound **SI3** in 63% yield (470 mg) as an off-white solid.

^{1}H NMR (400 MHz, $CDCl_3$): δ 8.07 – 7.99 (m, 2H), 7.88 – 7.82 (m, 2H), 6.91 (dd, *J* = 16.7, 10.4 Hz, 1H), 6.41 (dd, *J* = 16.7, 1.4 Hz, 1H), 5.86 (dd, *J* = 10.4, 1.4 Hz, 1H), 3.35 (s, 3H) ppm.

^{13}C NMR (100 MHz, $CDCl_3$): δ 165.9, 143.0, 133.1 (2C), 132.7, 128.4 (2C), 128.0, 117.7, 117.1, 33.5 ppm.

IR (neat) ν_{max}: 2234, 1684, 1400, 1352, 1157, 1081, 724, 635, 588, 567 cm^{-1}.

HRMS (ESI^+): exact mass calculated for $[M+Na]^+$ ($C_{11}H_{10}N_2NaO_3S^+$) requires *m/z* 273.0304, found *m/z* 273.0304.

Characterisation of aryl migrated products

General Procedure D: Truce-Smiles rearrangement

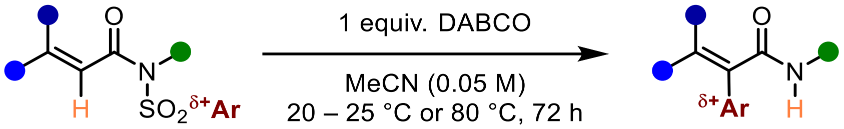

To an oven-dried 4 or 8 mL vial containing a magnetic stir bar was added sulfonyl carboxamide (1.0 equiv.). The compound was dissolved in dry MeCN (0.05 M) and DABCO (1.0 equiv.) was added. The vial was capped and the mixture stirred at 20 – 25 °C or 80 °C in an oil bath for 72 h, upon which it was diluted with DCM (20 mL) and the solution transfered to a separatory funnel. The vial was washed with saturated, aqueous $NaHCO_3$ solution (10 mL) and transfered to the same separatory funnel. The phases were separated and the aqueous phase was extracted with DCM (2 x 10 mL). The combined organic phases were dried over $MgSO_4$, filtered and the solvent was removed under reduced pressure. The mixture was purified via column chromatography to afford the pure product.

2a: *N*-Methyl-2-(4-nitrophenyl)acrylamide

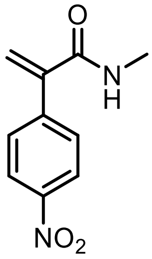

Following General procedure D at 20 – 25 °C using **1a** (54.1 mg, 0.20 mmol), the purification by column chromatography on silica (20 – 80% EtOAc in heptane) afforded compound **2a** in 92% yield (37.9 mg) as a white solid.

^{1}H NMR (400 MHz, $CDCl_3$): δ 8.23 (d, *J* = 7.7 Hz, 2H), 7.60 (d, *J* = 7.7 Hz, 2H), 6.11 (s, 1H), 5.78 (s, 1H), 5.72 (bs, 1H), 2.93, (s, 3H) ppm.

^{13}C NMR (150 MHz, $CDCl_3$): δ 167.5, 147.9, 143.8, 143.4, 129.0 (2C), 124.0 (2C), 123.1, 26.9 ppm.

IR (neat) ν_{max}: 3299, 1649, 1609, 1596, 1407, 1344, 857.

HRMS (ESI$^+$): exact mass calculated for $[M+H]^+$ ($C_{10}H_{11}N_2O_3^+$) requires *m/z* 207.0764, found *m/z* 207.0766.

2b: *N*-Isopropyl-2-(4-nitrophenyl)acrylamide

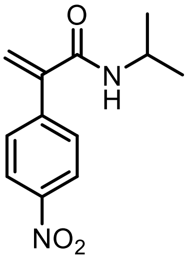

Following General Procedure D at 20 – 25 °C using **1b** (59.7 mg, 0.20 mmol), the purification by column chromatography on silica (20 – 80% EtOAc in heptane) afforded compound **2b** in 68% yield (31.9 mg) as a white solid.

^{1}H NMR (400 MHz, $CDCl_3$): δ 8.24 (d, *J* = 8.9 Hz, 2H), 7.60 (d, *J* = 8.8 Hz, 2H), 6.05 (s, 1H), 5.78 (s, 1H), 5.51 (s, 1H), 1.55 (m, 1H), 1.21 (d, *J* = 6.6 Hz, 6H) ppm.

^{13}C NMR (100 MHz, $CDCl_3$): δ 166.2, 147.9, 144.2, 143.3, 128.8 (2C), 124.0 (2C), 122.6, 42.2, 22.8 (2C) ppm.

IR (neat) v_{max}: 3307, 2980, 1638, 1510, 1343, 1231, 944, 857 cm^{-1}.

HRMS (ESI$^+$): exact mass calculated for $[M+Na]^+$ ($C_{12}H_{14}N_2NaO_5S^+$) requires m/z 257.0897, found m/z 257.0888.

2c: *N*-(*tert*-Butyl)-2-(4-nitrophenyl)acrylamide

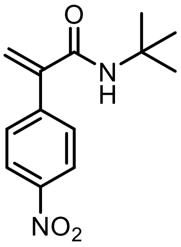

Following General Procedure D at 80 °C using **1c** (31.2 mg, 0.10 mmol), the purification by column chromatography on silica (0 – 50% EtOAc in heptane) afforded compound **2c** in 63% yield (15.8 mg) as a white solid.
[At 20 – 25 °C using **1c** (62.5 mg, 0.20 mmol) in 49% yield (24.2 mg).]

^{1}H NMR (400 MHz, $CDCl_3$): δ 8.23 (d, *J* = 8.8 Hz, 2H), 7.60 (d, *J* = 8.8 Hz, 2H), 5.98 (s, 1H), 5.74 (s, 1H), 5.55 (bs, 1H), 1.41 (s, 9H) ppm.

^{13}C NMR (100 MHz, $CDCl_3$): δ 166.5, 147.8, 145.0, 143.5, 128.7 (2C), 124.0 (2C), 121.8, 52.1, 28.9 (3C) ppm.

IR (neat) v_{max}: 3259, 3068, 2977, 1644, 1593, 1545, 1514, 1364, 860 cm^{-1}.

HRMS (ESI$^+$): exact mass calculated for $[M+Na]^+$ ($C_{13}H_{16}N_2NaO_3^+$) requires *m/z* 271.1053, found *m/z* 271.1054.

2d: *N*-allyl-2-(4-nitrophenyl)acrylamide

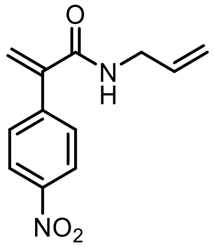

Following General Procedure D at 20 – 25 °C using **1d** (59.3 mg, 0.20 mmol), the purification by column chromatography on silica (15 – 50% EtOAc in heptane) afforded compound **2d** in 72% (33.2 mg) yield as a white solid.

^{1}H NMR (400 MHz, CDCl$_3$): δ 8.23 (d, *J* = 8.8 Hz, 2H), 7.61 (d, *J* = 8.8 Hz, 2H), 6.11 (s, 1H), 5.93 – 5.76 (m, 2H), 5.80 (s, 1H), 5.24 – 5.13 (m, 2H), 4.04 – 3.95 (m, 2H) ppm.

^{13}C NMR (100 MHz, CDCl$_3$): δ 166.7, 147.9, 143.7, 143.2, 133.7, 128.9 (2C), 124.0 (2C), 123.2, 117.2, 42.5 ppm.

IR (neat) ν_{max}: 3271, 1642, 1595, 1515, 1344, 856 cm^{-1}.

HRMS (ESI$^+$): exact mass calculated for $[M+Na]^+$ ($C_{12}H_{12}N_2NaO_3^+$) requires *m/z* 255.0740, found *m/z* 255.0736.

2e: *N*-Benzyl-2-(4-nitrophenyl)acrylamide

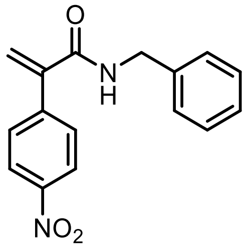

Following General Procedure 20 – 25 °C using **1e** (69.3 mg, 0.20 mmol), the purification by column chromatography on silica (5 – 40% EtOAc in heptane) afforded compound **2e** in 63% yield (35.3 mg) as a white solid.

^{1}H NMR (400 MHz, CDCl$_3$): δ 8.23 (d, *J* = 8.7 Hz, 2H), 7.61 (d, *J* = 8.7 Hz, 2H), 7.40 – 7.27 (m, 5H), 6.13 (s, 1H), 6.00 (bs, 1H), 5.82 (s, 1H), 4.57 (d, *J* = 5.7 Hz, 2H) ppm.

^{13}C NMR (100 MHz, CDCl$_3$): δ 166.7, 148.0, 143.7, 143.2, 137.8, 129.1 (2C), 128.9 (2C), 128.0 (3C), 124.0 (2C), 123.3, 44.2 ppm.

IR (neat) ν_{max}: 3284, 1662, 1513, 1345, 858, 700 cm^{-1}.

HRMS (ESI$^+$): exact mass calculated for $[M+Na]^+$ ($C_{16}H_{14}N_2NaO_3^+$) requires *m/z* 305.0902, found *m/z* 305.0901.

2f: *N*-(4,4-dimethoxybutyl)-2-(4-nitrophenyl)acrylamide

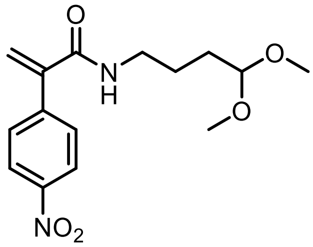

Following General Procedure D at 20 – 25 °C using **1f** (74.5 mg, 0.20 mmol), the purification by column chromatography on silica (15 – 80% EtOAc in heptane) afforded compound **2f** in 74% yield (45.8 mg) as a white solid.

^{1}H NMR (400 MHz, $CDCl_3$): δ 8.20 (d, *J* = 8.2 Hz, 2H), 7.59 (d, *J* = 8.2 Hz, 2H), 6.06 (bs, 2H), 5.77 (s, 1H), 4.37 – 4.31 (m, 1H), 3.38 (app.q, *J* = 6.4 Hz, 2H), 3.29 (s, 6H), 1.68 – 1.59 (m, 4H) ppm.

^{13}C NMR (100 MHz, $CDCl_3$): δ 166.9, 147.8, 144.0, 143.4, 128.9 (2C), 123.9 (2C), 122.7, 104.4, 53.4 (2C), 39.8, 30.1, 24.3 ppm.

IR (neat) ν_{max}: 1520, 1345, 903, 859, 726, 648 cm^{-1}.

HRMS (ESI$^+$): exact mass calculated for $[M+Na]^+$ ($C_{15}H_{20}N_2NaO_5^+$) requires *m/z* 331.1264, found *m/z* 331.1256.

2g: Methyl (2-(4-nitrophenyl)acryloyl)glycinate

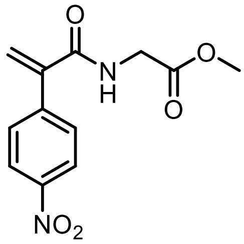

Following General Procedure D at 20 – 25 °C using **1g** (65.7 mg, 0.20 mmol), the purification by column chromatography on silica (20 – 50% EtOAc in heptane) afforded compound **2g** in 42% yield (22.2 mg) as a white solid.

^{1}H NMR (400 MHz, $CDCl_3$): δ 8.25 (d, *J* = 8.9 Hz, 2H), 7.64 (d, *J* = 8.9 Hz, 2H), 6.25 (s, 1H), 6.18 (s, 1H), 5.86 (s, 1H), 4.17 (d, *J* = 5.3 Hz, 2H), 3.79 (s, 3H) ppm.

^{13}C NMR (100 MHz, $CDCl_3$): δ 170.3, 167.0, 148.0, 143.1, 142.9, 129.0 (2C), 124.1 (2C), 123.9, 52.7, 41.7 ppm.

IR (neat) ν_{max}: 1748, 1657, 1597, 1517, 1344, 1209, 858, 709 cm^{-1}.

HRMS (ESI$^+$): exact mass calculated for $[M+Na]^+$ ($C_{12}H_{12}N_2NaO_5^+$) requires *m/z* 287.0638, found *m/z* 287.0634.

2h: Methyl (2-(4-nitrophenyl)acryloyl)methioninate

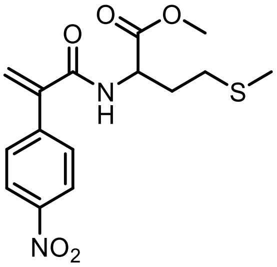

Following General Procedure D at 20 – 25 °C using **1h** (80.5 mg, 0.20 mmol), the purification by column chromatography on silica (0 – 40% EtOAc in heptane) afforded compound **2h** in 58% yield (39.5 mg) as a white solid.

^{1}H NMR (400 MHz, CDCl$_3$): δ 8.24 (d, *J* = 8.8 Hz, 2H), 7.62 (d, *J* = 8.8 Hz, 2H), 6.56 (d, *J* = 7.5 Hz, 1H), 6.17 (s, 1H), 5.85 (s, 1H), 4.89 – 4.79 (m, 1H), 3.79 (s, 3H), 2.53 (t, *J* = 7.3 Hz, 2H), 2.27 – 2.18 (m, 1H), 2.10 – 2.02 (m, 1H), 2.08 (s, 3H) ppm.

^{13}C NMR (100 MHz, CDCl$_3$): δ 172.3, 166.7, 148.0, 143.2, 142.9, 129.0 (2C), 124.0 (2C), 123.9, 52.9, 52.2, 31.2, 30.3, 15.7 ppm.

IR (neat) ν_{max}: 3284, 1744, 1669, 1516, 1344, 1208, 859 cm^{-1}.

HRMS (ESI$^+$): exact mass calculated for [M+Na]$^+$ ($C_{15}H_{18}N_2NaO_5S^+$) requires *m/z* 361.0829, found *m/z* 361.0823.

2i: *N*-(3,4-Dimethoxyphenethyl)-2-(4-nitrophenyl)acrylamide

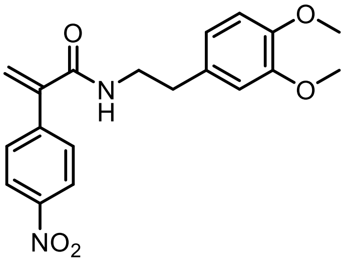

Following General Procedure D at 20 – 25 °C using **1i** (84.1 mg, 0.20 mmol), the purification by column chromatography on silica (10 – 50% EtOAc in heptane) afforded compound **2i** in 69% yield (49.0 mg) as a white solid.

^{1}H NMR (400 MHz, CDCl$_3$): δ 8.17 (d, *J* = 8.7 Hz, 2H), 7.47 (d, *J* = 8.7 Hz, 2H), 6.77 (d, *J* = 8.0 Hz, 1H), 6.73–6.64 (m, 2H), 6.04 (s, 1H), 5.73 (s, 1H), 5.67 (bs, 1H), 3.86 (s, 3H), 3.84 (s, 3H), 3.66 – 3.59 (m, 2H), 2.82 (t, *J* = 6.8 Hz, 2H) ppm.

^{13}C NMR (100 MHz, CDCl$_3$): δ 166.7, 149.3, 148.1, 147.9, 143.8, 143.2, 130.9, 128.9 (2C), 123.9 (2C), 123.1, 120.8, 112.0, 111.4, 56.1, 56.0, 41.0, 35.0 ppm.

IR (neat) ν_{max}: 3345, 2935, 1663, 1513, 1343, 1261, 1234, 1027, 858 cm^{-1}.

HRMS (ESI$^+$): exact mass calculated for [M+Na]$^+$ ($C_{19}H_{20}N_2NaO_5^+$) requires *m/z* 379.1264, found *m/z* 379.1257.

2j: *tert*-Butyl 3-(2-(2-(4-nitrophenyl)acrylamido)ethyl)-1H-indole-1-carboxylate

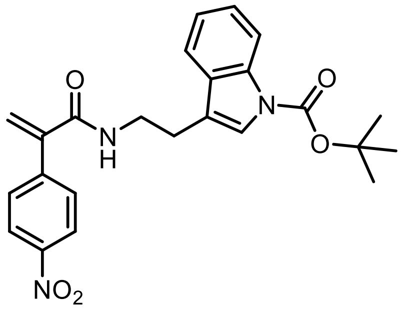

Following General Procedure D at 20 – 25 °C using **1j** (99.9 mg, 0.20 mmol), the purification by column chromatography on silica (5 – 50% EtOAc in heptane) afforded compound **2j** in 67% yield (58.2 mg) as a yellow oil.

^{1}H NMR (400 MHz, CDCl$_3$): δ 8.19 – 8.06 (m, 3H), 7.55 – 7.38 (m, 4H), 7.33 (app.t, *J* = 6.6 Hz, 1H), 7.22 (app.t, *J* = 6.6 Hz, 1H), 6.08 (s, 1H), 5.75 (bs, 1H), 5.72 (s, 1H), 3.71 (app.q, *J* = 6.4 Hz, 2H), 2.99 (t, *J* = 6.4 Hz, 2H), 1.67 (s, 9H) ppm.

^{13}C NMR (100 MHz, CDCl$_3$): δ 166.7, 149.8, 147.9, 143.7, 143.2, 135.6, 130.4, 128.9 (2C), 124.8, 123.9 (2C), 123.49, 123.47, 122.8, 118.9, 117.4, 115.6, 84.1, 39.9, 28.3 (3C), 24.9 ppm.

IR (neat) ν_{max}: 3297, 2979, 1728, 1664, 1516, 1372, 1342, 1154, 833, 746 cm^{-1}.

HRMS (ESI$^+$): exact mass calculated for $[M+Na]^+$ ($C_{24}H_{25}N_3NaO_5^+$) requires *m/z* 458.1686, found *m/z* 458.1681.

2k: Ethyl (*E*)-6-(2-(4-nitrophenyl)acrylamido)hex-2-enoate

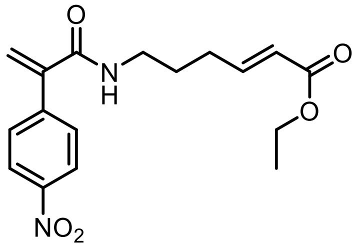

Following General Procedure D at 80 °C using **1k** (39.6 mg, 0.10 mmol), the purification by column chromatography on silica (10 – 50% EtOAc in heptane) afforded compound **2k** in 78% yield (26.0 mg) as a white solid.

^{1}H NMR (600 MHz, CDCl$_3$): δ 8.24 – 8.20 (m, 2H), 7.61 – 7.56 (m, 2H), 6.92 (dt, *J* = 15.6, 6.9 Hz, 1H), 6.08 (s, 1H), 5.87 – 5.80 (m, 2H), 5.78 (s, 1H), 4.17 (q, *J* = 7.1 Hz, 2H), 3.39 (q, *J* = 6.7 Hz, 2H), 2.29 – 2.23 (m, 2H), 1.77 – 1.71 (m, 2H), 1.27 (t, *J* = 7.1 Hz, 3H) ppm.

^{13}C NMR (150 MHz, CDCl$_3$): δ 166.9, 166.5, 147.9, 147.5, 143.8, 143.2, 128.9 (2C), 124.0 (2C), 123.1, 122.3, 60.4, 39.6, 29.7, 28.1, 14.4 ppm.

IR (neat) ν_{max}: 3301, 2924, 2853, 1716, 1649, 1518, 1345, 859, 733 cm^{-1}.

HRMS (ESI$^+$): exact mass calculated for $[M+Na]^+$ ($C_{17}H_{20}N_2NaO_5^+$) requires *m/z* 355.1264, found *m/z* 355.1263.

2l: *N*-Methyl-2-(2-nitrophenyl)acrylamide

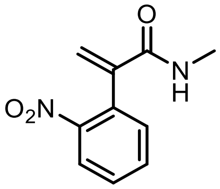

Following General Procedure D at 20 – 25 °C using **1l** (54.1 mg, 0.20 mmol), the purification by column chromatography on silica (5 – 60% EtOAc in heptane) afforded compound **2l** in 62% yield (25.4 mg) as a white solid.

^{1}H NMR (400 MHz, $CDCl_3$): δ 8.08 (d, *J* = 8.1 Hz, 1H), 7.66 (app. t, *J* = 8.1 Hz, 1H), 7.58 – 7.51 (m, 1H), 7.44 (d, *J* = 7.6 Hz, 1H), 6.10 (s, 1H), 5.75 (bs, 1H), 5.58 (s, 1H), 2.87 (d, *J* = 4.9 Hz, 3H) ppm.

^{13}C NMR (100 MHz, $CDCl_3$): δ 166.6, 148.1, 143.3, 133.7, 133.3, 132.7, 129.7, 124.8, 121.8, 26.8 ppm.

IR (neat) ν_{max}: 3319, 1651, 1618, 1522, 1347 cm^{-1}.

HRMS (ESI$^+$): exact mass calculated for $[M+Na]^+$ ($C_{10}H_{10}N_2NaO_3^+$) requires *m/z* 229.0584, found *m/z* 229.0586.

2m: 2-(2-Methoxy-4-nitrophenyl)-*N*-methylacrylamide

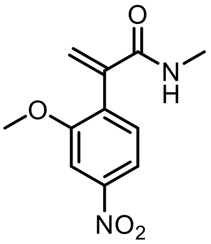

Following General Procedure D at 20 – 25 °C using **1m** (60.1 mg, 0.20 mmol), the purification by flash column chromatography (20 – 80% EtOAc in heptane) afforded compound **2m** in 84% yield (39.8 mg) as a white solid.

^{1}H NMR (400 MHz, $CDCl_3$): δ 7.80 (dd, *J* = 8.3, 2.1 Hz, 1H), 7.68 (d, *J* = 2.0 Hz, 1H), 7.33 (d, *J* = 8.3 Hz, 1H), 6.16 (s, 1H), 5.53 (s, 1H), 5.50 (s, 1H), 3.83 (s, 3H), 2.80 (d, *J* = 4.9 Hz, 3H) ppm.

^{13}C NMR (100 MHz, $CDCl_3$): δ 167.2, 157.5, 149.3, 141.4, 133.8, 131.4, 124.6, 116.3, 106.2, 56.5, 26.8 ppm.

IR (neat) ν_{max}: 3296, 1510, 1340, 1251, 955, 863, 806, 736 cm^{-1}.

HRMS (ESI$^+$): exact mass calculated for $[M+Na]^+$ ($C_{11}H_{12}N_2NaO_4Na^+$) requires m/z 259.0689, found m/z 259.0689.

2n: 2-(2,5-Bis(trifluoromethyl)phenyl)-*N*-methylacrylamide

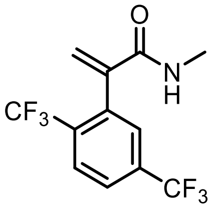

Following General Procedure D at 80 °C using **1n** (72.3 mg, 0.20 mmol), the purification by column chromatography on silica (5 – 20% EtOAc in heptane) afforded compound **2n** in 49% yield (29.5 mg) as an off-white solid (containing trace amounts of inseparable sulfonamide 1,4-adduct).

^{1}H NMR (400 MHz, CDCl$_3$): δ 7.87 (d, *J* = 8.3 Hz, 1H), 7.77 (d, *J* = 8.3 Hz, 1H), 7.64 (s, 1H), 6.45 (s, 1H), 5.58 (s, 1H), 5.37 (bs, 1H), 2.86 (d, *J* = 4.9 Hz, 3H) ppm.

^{13}C NMR (100 MHz, CDCl$_3$): δ 165.9, 140.7, 137.4, 134.2 (q, *J* = 33.6 Hz), 132.6 (q, *J* = 30.4 Hz), 129.0 (q, *J* = 3.6 Hz), 127.4 (q, *J* = 5.0 Hz), 126.4, 125.8 (q, *J* = 3.6 Hz), 123.24 (q, *J* = 274.4 Hz), 123.15 (q, *J* = 273.1 Hz), 27.0 ppm

^{19}F NMR (377 MHz, CDCl$_3$): δ -59.0, -63.2 ppm.

IR (neat) ν_{max}: 3317, 1661, 1614, 1536, 1314, 1178, 1129, 1084, 1040, 909, 842, 736 cm^{-1}.

HRMS (ESI$^+$): exact mass calculated for $[M+Na]^+$ ($C_{12}H_9F_6NNaO^+$) requires *m/z* 320.0481, found *m/z* 320.0478.

2o: 2-(4-Cyano-2-(trifluoromethyl)phenyl)-*N*-methylacrylamide

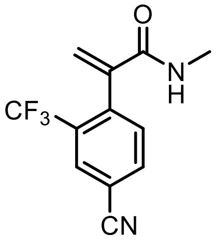

Following General Procedure D at 80 °C using **1o** (33.5 mg, 105 µmol), the purification by column chromatography on silica (20 – 100% EtOAc in heptane) afforded compound **2o** in 42% yield (11.2 mg) as a white solid.

^{1}H NMR (700 MHz, CDCl$_3$): δ 8.01 (s, 1H), 7.87 (dd, *J* = 7.9, 1.2 Hz, 1H), 7.54 (d, *J* = 7.9 Hz, 1H), 6.39 (s, 1H), 5.57 (s, 1H), 5.41 (bs, 1H), 2.86 (d, *J* = 4.9 Hz, 3H) ppm.

^{13}C NMR (100 MHz, CDCl$_3$): δ 165.7, 141.1, 140.8, 135.2, 133.2, 130.7 (q, *J* = 31.4 Hz), 130.3 (q, *J* = 5.3 Hz), 125.9, 124.2 (q, *J* = 274.7 Hz), 117.2, 113.2, 27.0 ppm

^{19}F NMR (659 MHz, CDCl$_3$): δ -59.0 ppm.

IR (neat) ν_{max}: 3081, 2942, 2236, 1664, 1610, 1537, 1315, 1173, 1132 cm^{-1}.

HRMS (ESI$^+$): exact mass calculated for $[M+Na]^+$ ($C_{12}H_9F_3N_2NaO^+$) requires *m/z* 277.0559, found *m/z* 277.0559.

2p: 2-(*N*-Acryloyl-*N*-methylsulfamoyl)pyridine 1-oxide

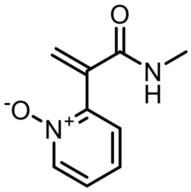

Following General Procedure D at 20-25 °C using **1p** (43.0 mg, 0.177 mmol), the purification by column chromatography on silica (0 – 10% MeOH in DCM) afforded compound **2p** in 44% yield (14.1 mg) as a white solid.

^{1}H NMR (600 MHz, CDCl$_3$): δ 8.29 (br s, 1H), 8.26 (d, *J* = 6.3 Hz, 1H), 7.39 – 7.35 (m, 2H), 7.35-7.30 (m, 1H), 6.36 (s, 1H), 5.70 (s, 1H), 2.86 (d, *J* = 4.8 Hz, 3H).

^{13}C NMR (151 MHz, CDCl$_3$): δ 167.1, 148.9, 140.9, 140.1, 128.6, 128.4, 127.6, 125.9, 26.6.

IR (neat) ν_{max}: 3271, 3078, 2925, 1651, 1615, 1553, 1281, 947, 851, 699, 416 cm^{-1}.

HRMS (ESI$^+$): exact mass calculated for [M+Na]$^+$ ($C_9H_{10}N_2NaO_2^+$) requires *m/z* 201.0635, found *m/z* 201.0634.

2q: (*Z*)- and (*E*)-*N*-Methyl-2-(4-nitrophenyl)but-2-enamide

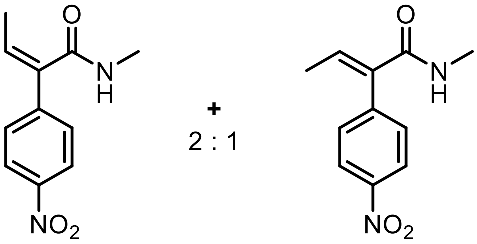

Following General Procedure D at 80 °C using **1q** (56.9 mg, 0.20 mmol), the purification by column chromatography on silica (5 – 60% EtOAc in heptane) afforded compound**(*Z*)-2q** and **(*E*)-2q** as a mixture of 2:1 in 79% combined yield (34.6 mg) as a white solid.

(*Z*): ^{1}H NMR (400 MHz, CDCl$_3$): δ 8.17 (d, *J* = 8.8 Hz, 2H), 7.52 (d, *J* = 8.8 Hz, 2H), 6.30 (q, *J* = 7.1 Hz, 1H), 5.63 (bs, 1H), 2.97 (d, *J* = 4.9 Hz, 3H), 2.01 (d, *J* = 7.1 Hz, 3H) ppm.

(*E*): ^{1}H NMR (400 MHz, CDCl$_3$): δ 8.29 (d, *J* = 8.6 Hz, 2H), 7.40 (d, *J* = 8.6 Hz, 2H), 7.12 (q, *J* = 7.3 Hz, 1H), 5.19 (bs, 1H), 2.83 (d, *J* = 4.9 Hz, 3H), 1.66 (d, *J* = 7.3 Hz, 3H) ppm.

(*Z*): ^{13}C NMR (100 MHz, CDCl$_3$): δ 168.6, 147.2, 143.8, 137.9, 131.8, 127.1 (2C), 124.1 (2C), 26.5, 16.1 ppm.

(*E*): ^{13}C NMR (100 MHz, CDCl$_3$): δ 166.4, 147.8, 142.9, 137.4, 135.5, 131.8 (2C), 124.2 (2C), 27.0, 15.2 ppm.

IR (neat) ν_{max} [mixture of isomers]: 3283, 1661, 1649, 1514, 1344, 854, 735 cm^{-1}.

HRMS (ESI$^+$): exact mass calculated for [M+Na]$^+$ ($C_{11}H_{12}N_2NaO_3^+$) requires *m/z* 243.0740, found *m/z* 243.0742.

2r: (*E*)-N-Methyl-2-(4-nitrophenyl)oct-3-enamide

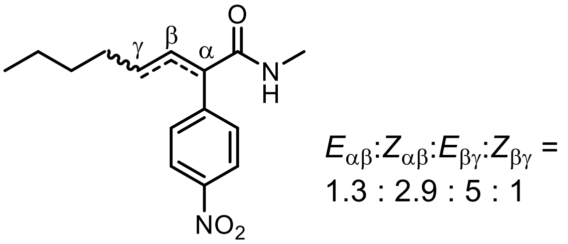

Following General Procedure D at 80 °C using **1r** (68.1 mg, 0.20 mmol), the purification by column chromatography on silica (20 – 40% EtOAc in heptane) afforded compound **2r** as a mixture of isomers in 91% combined yield (50.4 mg) as a colourless oil. The major isomer ((*E*)-N-methyl-2-(4-nitrophenyl)oct-3-enamide) is reported herein with the spectra partially containing the minor isomers.

^{1}H NMR (600 MHz, $CDCl_3$) [major isomer]: δ 8.19 – 8.14 (m, 2H), 7.47 – 7.42 (m, 2H), 5.90 – 5.59 (m, 3H), 4.20 (d, *J* = 8.2 Hz, 1H), 2.83 (d, *J* = 4.8 Hz, 3H), 2.13 – 2.03 (m, *J* = 14.3, 7.1 Hz, 1H), 1.39 – 1.24 (m, 4H), 0.88 (t, *J* = 7.2 Hz, 3H) ppm.

^{13}C NMR (150 MHz, $CDCl_3$) [major isomer]: δ 171.6, 147.2, 147.1, 136.4, 129.3, 126.9 (2C), 124.0 (2C), 56.4, 32.3, 31.2, 26.8, 22.4, 14.0 ppm.

IR (neat) ν_{max} [isomeric mixture]: 3287, 2957, 2928, 2855, 1646, 1518, 1345, 854, 738 cm^{-1}.

HRMS (ESI$^+$): exact mass calculated for $[M+Na]^+$ ($C_{15}H_{19}N_2NaO_3^+$) requires *m/z* 299.1366, found *m/z* 299.1362.

2s and *iso*-2s: *N*,3-Dimethyl-2-(4-nitrophenyl)but-3-enamide

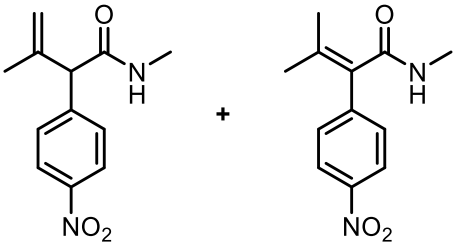

2s **iso-2s**

Following General Procedure D at 80 °C using **1s** (59.7 mg, 0.20 mmol), the purification by column chromatography on silica (10 – 50% EtOAc in heptane), afforded compound **2s** in 55% yield (25.9 mg) as a white solid and compound ***iso*-2s** in 27% yield (12.5 mg) as a white solid.

2s ^{1}H NMR (700 MHz, $CDCl_3$): δ 8.18 (dt, *J* = 8.8, 2.0 Hz, 2H), 7.48 (dt, *J* = 8.8, 2.0 Hz, 2H), 5.83 (bs, 1H), 5.11 (s, 1H), 4.87 (s, 1H), 4.26 (s, 1H), 2.86 (d, *J* = 4.9 Hz, 3H), 1.77 (s, 3H) ppm.

***iso*-2s ^{1}H NMR (700 MHz, $CDCl_3$)**: δ 8.29 – 8.15 (m, 2H), 7.49 – 7.39 (m, 2H), 5.37 (s, 1H), 2.83 (d, *J* = 4.9 Hz, 3H), 2.07 (s, 3H), 1.70 (s, 3H) ppm.

2s ^{13}C NMR (175 MHz, $CDCl_3$): δ 170.5, 147.3, 145.4, 143.1, 130.1 (2C), 123.8 (2C), 116.2, 60.5, 26.8, 22.1 ppm.

***iso*-2s ^{13}C NMR (175 MHz, $CDCl_3$):** δ 169.4, 147.1, 145.1, 140.8, 132.1, 130.3 (2C), 123.9 (2C), 26.6, 22.7, 22.4 ppm.

2s IR (neat) ν_{max}: 3311, 1681, 1518, 1375, 908, 867, 743 cm^{-1}.

***iso*-2s IR (neat) ν_{max}:** 3285, 1662, 1514, 1344, 1106, 851, 749, 703 cm^{-1}.

2s HRMS (ESI$^+$): exact mass calculated for [M+Na]$^+$ ($C_{12}H_{14}N_2NaO_3^+$) requires *m/z* 257.0897, found *m/z* 257.0896.

***iso*-2s HRMS (ESI$^+$):** exact mass calculated for [M+Na]$^+$ ($C_{12}H_{14}N_2NaO_3^+$) requires *m/z* 257.0897, found *m/z* 257.0899.

2t: *N*,4-Dimethyl-2-(4-nitrophenyl)pent-3-enamide

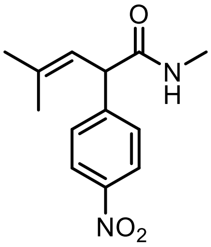

Following General Procedure D at 80 °C using **1t** (62.5 mg, 0.20 mmol), the purification by column chromatography on silica (0 – 40% EtOAc in heptane) afforded compound **2t** in 81% yield (40.3 mg) as an off-white solid.

^{1}H NMR (400 MHz, CDCl$_3$): δ 8.21 – 8.12 (m, 2H), 7.49 – 7.40 (m, 2H), 5.86 (bs, 1H), 5.57 – 5.50 (m, 1H), 4.44 (d, *J* = 9.3 Hz, 1H), 2.83 (d, *J* = 4.9 Hz, 3H), 1.80 (d, *J* = 1.0 Hz, 3H), 1.70 (d, *J* = 1.2 Hz, 3H) ppm.

^{13}C NMR (100 MHz, CDCl$_3$): δ 171.9, 148.0, 147.0, 137.8 , 129.1 (2C), 124.0 (2C), 121.7, 52.2, 26.8, 26.0, 18.4 ppm.

IR (neat) ν_{max}: 3305, 2937, 1681, 1515, 1345, 852, 741 cm^{-1}.

HRMS (ESI$^+$): exact mass calculated for [M+Na]$^+$ ($C_{13}H_{16}N_2NaO_3^+$) requires *m/z* 271.1053, found *m/z* 271.1060.

2u: *N*-methyl-2-(4-nitrophenyl)hexadienamide [isomeric mixture]

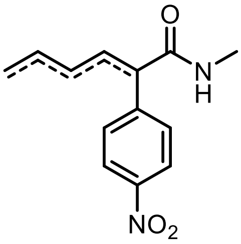

Following General Procedure D at 80 °C using **1u** (62.1 mg, 0.20 mmol), the purification by column chromatography on silica (0 – 50% EtOAc in heptane) afforded compound **2u** as a mixture of isomers in 73% combined yield (35.8 mg) as an orange oil.

^{1}H NMR (400 MHz, CDCl$_3$) [isomeric mixture]: δ 8.26 – 8.17 (m, 1.2H), 8.13 – 8.07 (m, 0.7H), 7.74 (d, *J* = 11.8 Hz, 0.1H), 7.56 – 7.45 (m, 0.7H), 7.40 – 7.31 (m, 1.8H), 6.99 (d, *J* = 11.8 Hz, 0.1H), 6.64 (d, *J* = 1.1 Hz, 0.3H), 6.49 – 6.30 (m, 0.4H), 6.22 – 6.00 (m, 0.9H), 5.90 – 5.67 (m, 1.1H), 5.23 (bs, 0.6H), 2.96 – 2.88 (m, 1.1H), 2.81 – 2.73 (m, 1.9H), 1.90 – 1.79 (m, 1.4H), 1.74 – 1.67 (m, 1.6H) ppm.

^{13}C NMR (100 MHz, CDCl$_3$) [isomeric mixture]: δ 168.5, 166.7, 147.7, 147.0, 143.7, 143.1, 140.6, 138.8, 138.57, 136.8, 134.3, 133.6, 133.0, 132.6, 131.5, 130.6, 128.1, 127.1, 127.1, 127.0, 124.7, 124.2, 124.1, 124.1, 27.1, 27.0, 26.6, 19.0, 18.9, 14.3 ppm.

IR (neat) ν_{max} [isomeric mixture]: 3419, 2937, 1655, 1628, 1513, 1343, 856, 735 cm^{-1}.

HRMS (ESI$^+$): exact mass calculated for $[M+Na]^+$ ($C_{13}H_{14}N_2NaO_3^+$) requires *m/z* 269,0897, found *m/z* 269.0901.

SI4: 2-(4-Nitrophenyl)-*N*-phenylacrylamide

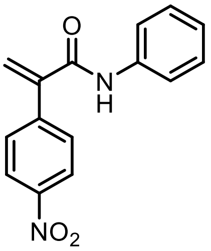

Following General Procedure D at 80 °C using **SI2** (66.5 mg, 0.20 mmol), the purification by column chromatography on silica (50 – 80% EtOAc in heptane) afforded compound **SI4** in 28% yield (14.9 mg) as a white solid.

^{1}H NMR (400 MHz, CDCl$_3$): δ 8.27 (d, *J* = 8.9 Hz, 2H), 7.68 (d, *J* = 8.8 Hz, 2H), 7.55 (d, *J* = 7.6 Hz, 2H), 7.44 – 7.31 (m, 3H), 7.17 (t, *J* = 7.4 Hz, 1H), 6.24 (s, 1H), 5.93 (s, 1H) ppm.

^{13}C NMR (100 MHz, CDCl$_3$): δ 164.9, 148.1, 144.3, 142.8, 137.4, 129.3 (2C), 129.0 (2C), 125.3, 124.2 (2C), 123.7, 120.2 (2C) ppm.

IR (neat) ν_{max}: 3286, 1651, 1595, 1514, 1340, 1247, 760, 690 cm^{-1}.

HRMS (ESI$^+$): exact mass calculated for $[M+Na]^+$ ($C_{15}H_{12}N_2NaO_3^+$) requires m/z 291.0740, found m/z 291.0739.

SI5: 2-(4-Cyanophenyl)-*N*-methylacrylamide

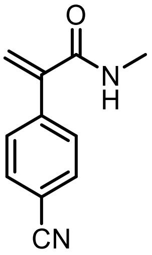

To an oven-dried 8 mL microwave vial containing a magnetic stir bar was added **SI2** (50.1 mg, 0.20 mmol). The compound was dissolved in dry MeCN (0.05 M) and DABCO (1.0 equiv.) was added. The vial was seal and the mixture stirred at 140 °C under microwave radiation for 5 h. After cooling to ambient temperature, it was diluted with DCM (20 mL) and the solution transfered to a separatory funnel. The vial was washed with saturated, aqueous $NaHCO_3$ solution (10 mL) and transfered to the same separatory funnel. The phases were separated and the aqueous phase was extracted with DCM (2 x 10 mL). The combined organic phases were dried over $MgSO_4$, filtered and the solvent was removed under reduced pressure. The mixture was purified via column chromatography on silica (50 – 80% EtOAc in heptane) afforded compound **SI4** in 33% yield (12.3 mg) as a white solid.

^{1}H NMR (400 MHz, CDCl$_3$): δ 7.67 (d, *J* = 7.5 Hz, 2H), 7.53 (d, *J* = 7.5 Hz, 2H), 6.08 (s, 1H), 5.73 (s, 1H), 5.70 (bs, 1H), 2.93 (d, *J* = 4.8 Hz, 3H) ppm.

^{13}C NMR (100 MHz, CDCl$_3$): δ 167.5, 144.0, 141.5, 132.6 (2C), 128.8 (2C), 122.8, 118.6, 112.4, 26.9 ppm.

IR (neat) ν_{max}: 3293, 2227, 1642, 1601, 1551, 1406, 845, 744, 545 cm^{-1}.

HRMS (ESI$^+$): exact mass calculated for $[M+Na]^+$ ($C_{11}H_{10}N_2NaO^+$) requires *m/z* 209.0685, found *m/z* 209.0680.

Characterisation of byproducts

1-rc: *N*1,*N*5-Dimethyl-2-methylene-*N*1,*N*5-bis((4-nitrophenyl)sulfonyl)pentanediamide

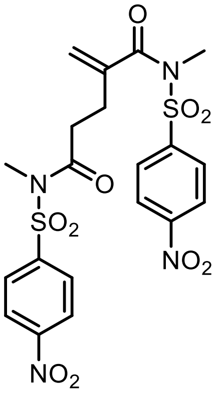

1-rc was isolated during the optimisation of the reaction conditions on standard substrate **1a** as a colourless solid.

^{1}H NMR (600 MHz, $CDCl_3$): δ 8.44 – 8.32 (m, 4H), 8.11 (d, *J* = 8.8 Hz, 4H), 5.45 (s, 1H), 5.22 (s, 1H), 3.30 (d, *J* = 26.5 Hz, 6H), 2.83 (t, *J* = 6.9 Hz, 2H), 2.62 (t, *J* = 6.8 Hz, 2H) ppm.

^{13}C NMR (151 MHz, $CDCl_3$): δ 171.9, 171.5, 150.8 (2C), 144.5, 144.2, 142.3, 129.8 (2C), 129.4 (2C), 124.6 (2C), 124.4 (2C), 122.2, 35.1, 35.0, 33.4, 28.0. ppm.

IR (neat) v_{max} 3106, 2956, 2925, 2854, 1695, 1529, 1349, 1170, 742.

HRMS (ESI$^+$): exact mass calculated for $[M+Na]^+$ ($C_{20}H_{20}N_4NaO_{10}S_2^+$) requires *m/z* 563.5014, found *m/z* 563.5015.

1-sa: *N*-Methyl-3-((*N*-methyl-4-nitrophenyl)sulfonamido)-*N*-((4-nitrophenyl)sulfonyl)-propanamide

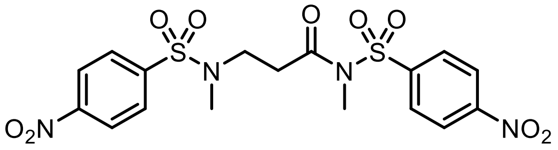

1-sa was isolated during the optimisation of the reaction conditions on standard substrate **1a** as a colourless solid.

^{1}H NMR (400 MHz, $CDCl_3$): δ 8.42 (dt, *J* = 9.3, 2.3 Hz, 2H), 8.38 (dt, *J* = 9.3, 2.3 Hz, 2H), 8.16 (dt, *J* = 9.3, 2.3 Hz, 2H), 7.94 (dt, *J* = 9.3, 2.3 Hz, 2H), 3.38 (s, 3H), 3.33 – 3.28 (m, 2H), 3.03 (t, *J* = 6.8 Hz, 2H), 2.81 (s, 3H) ppm.

^{13}C NMR (100 MHz, $CDCl_3$): δ 170.9, 150.9, 150.4, 144.2, 143.0, 129.4 (2C), 128.7 (2C), 124.69 (2C), 124.66 (2C), 46.4, 37.0, 36.9, 33.4 ppm.

IR (neat) v_{max} 3109, 2929, 1703, 1529, 1349, 1164, 742.

HRMS (ESI$^+$): exact mass calculated for $[M+Na]^+$ ($C_{17}H_{18}N_4NaO_9S_2^+$) requires *m/z* 509.0407, found *m/z* 509.0403.

Characterisation of cyclisation reaction products

3: 1-Methyl-3-(4-nitrophenyl)-5,6-diphenylpyridin-2(1*H*)-one

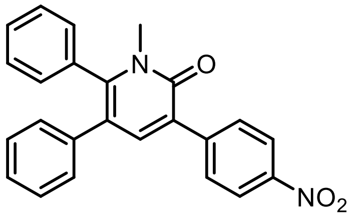

Following procedure reported by Ackermann *et al.*[15] using **2a** (103 mg, 0.50 mmol), diphenyl acetylene (44.6 mg, 0.25 mmol), $Cu(OAc)_2{\cdot}H_2O$ (49.9 mg, 0.25 mmol) and dichloro(*p*-cymene)ruthenium(II) dimer (7.7 mg, 12.5 µmol), the purification by column chromatography on silica (10 – 40% EtOAc in heptane) afforded compound **3** in 91% yield (87.3 mg) as a yellow solid.

^{1}H NMR (400 MHz, $CDCl_3$): δ 8.32 – 8.22 (m, 2H), 8.06 – 7.98 (m, 2H), 7.72 (s, 1H), 7.40 – 7.31 (m, 3H), 7.23 – 7.10 (m, 5H), 7.04 – 6.96 (m, 2H), 3.44 (s, 3H) ppm.

^{13}C NMR (100 MHz, $CDCl_3$): 161.3, 148.1, 147.1, 143.7, 141.0, 138.1, 134.1, 129.73 (2C), 129.67 (2C), 129.5 (2C), 129.3, 128.9 (2C), 128.2 (2C), 127.1, 126.9, 123.5 (2C), 120.9, 35.6 ppm.

IR (neat) ν_{max}: 1646, 1536, 1512, 1343, 910, 854, 735, 701 cm^{-1}.

HRMS (ESI$^+$): exact mass calculated for $[M+Na]^+$ ($C_{24}H_{18}N_2NaO_3^+$) requires *m/z* 405.1210, found *m/z* 405.1209.

4: 1-(2-Methoxypyrrolidin-1-yl)-2-(4-nitrophenyl)prop-2-en-1-one

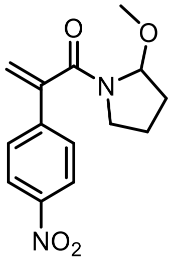

To an oven dried 4 mL vial containing a magnetic stir bar were added **2f** (30.8 mg, 0.10 mmol), dry MeOH (1 mL) and *para*-toluenesulfonic acid (23 µL, 0.15 mmol). The vial was capped and the suspension stirred at 21 °C for 20 h. The mixture was cooled to 0 °C, washed with a saturated aqueous $NaHCO_3$ solution and diluted with DCM (10 mL). The layers were separated and the aqueous phase was extracted with DCM (2 x 10 mL). The combined organic layers were dried over Na_2SO_4, filtered and the solvent was removed under reduced pressure. Purification by column chromatography on silica (10 – 100% EtOAc in heptane), afforded compound **4** in 43% yield (11.8 mg) as a white solid. The starting material **2f** was recovered in 54% yield (16.7 mg).

^{1}H NMR (600 MHz, $CDCl_3$) [two rotamers (0.6:0.4 ratio)]: δ 8.21 (m, 2.0H), 7.63 (m, 2.0H), 5.91 (app d, *J* = 17.9 Hz, 1.0H), 5.67 (app d, *J* = 21.0 Hz, 1.0H), 5.61 (d, *J* = 4.5 Hz, 0.4H), 4.84 (d, *J* = 4.4 Hz, 0.6H), 3.83 – 3.68 (m, 0.6H), 3.60 – 3.52 (m, 0.6H), 3.50 (s, 1.2H), 3.39 – 3.32 (m, 0.4H), 3.26 – 3.20 (m, 0.4H), 3.04 (s, 1.8H), 2.11 – 2.01 (m, 1.6H), 1.98 (m, 1.0H), 1.87 – 1.79 (m, 0.8H), 1.79 – 1.71 (m, 0.6H) ppm.

[15] L. Ackermann, A. V. Lygin, N. Hofmann, *Org. Lett.* **2011**, *13*, 3278–3281.

^{13}C NMR (151 MHz, $CDCl_3$) [two rotamers (0.6:0.4 ratio)]: δ 169.6 (0.4 C), 169.2 (0.6 C), 147.9 (0.4 C), 147.7 (0.6 C), 144.3 (0.4 C), 143.7 (0.6 C), 142.6 (0.6 C), 141.9 (0.4 C), 127.1 (1.2 C), 127.0 (0.8 C), 124.3 (0.8 C), 124.0 (1.2 C), 118.7 (1C), 90.2 (0.6 C), 87.5 (0.4 C), 57.1 (0.4 C), 54.5 (0.6 C), 47.6 (0.4 C), 45.3 (0.6 C), 31.7 (0.4 C), 30.9 (0.6 C), 23.0 (0.4 C), 21.2 (0.6 C) ppm.

IR (neat) ν_{max}: 3105, 3079, 2980, 2936, 2891, 2829, 1644, 1595, 1517, 1431, 1401, 1342, 1178, 1080, 1071, 934, 859 cm^{-1}.

HRMS (ESI$^+$): exact mass calculated for $[M+H]^+$ ($C_{14}H_{17}N_2O_4^+$) requires *m/z* 277.1183, found *m/z* 277.1182.

5: (*Z*)-*N*,5,5-Trimethyl-3-(4-nitrophenyl)furan-2(5*H*)-imine

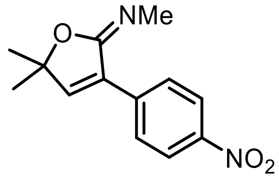

To an oven dried 4 mL vial containing a magnetic stir bar were added **2t** (24.8 mg, 0.10 mmol), dry MeCN (1 mL), K_2CO_3 (55.3 mg, 0.40 mmol) and I_2 (50.8 mg, 0.20 mmol). The vial was capped and the suspension stirred rapidly at 20 –25 °C for 96 h. The mixture was diluted with DCM (10 mL) and washed with a saturated, aqueous $Na_2S_2O_3$ solution. The layers were separated and the aqueous layer was extracted with DCM (2 x 10 mL). The combined organic layers were dried over $MgSO_4$, filtered and the solvent was removed under reduced pressure. Purification by column chromatography on silica (0 – 50% EtOAc in heptane) afforded compound **5** in 67% yield (16.6 mg) as a white solid.

^{1}H NMR (700 MHz, $CDCl_3$): δ 8.23 – 8.20 (m, 2H), 8.06 – 8.02 (m, 2H), 7.10 (s, 1H), 3.14 (s, 3H), 1.52 (s, 6H) ppm.

^{13}C NMR (175 MHz, $CDCl_3$): 161.0, 148.2, 147.7, 137.9, 131.7, 128.9 (2C), 123.6 (2C), 86.1, 34.8, 26.7 (2C) ppm.

IR (neat) ν_{max}: 2978, 2932, 2869, 1682, 1598, 1514, 1349, 1112, 948, 845, 759, 691 cm^{-1}.

HRMS (ESI$^+$): exact mass calculated for $[M+H]^+$ ($C_{13}H_{15}N_2O_3^+$) requires *m/z* 247.1077, found *m/z* 247.1077.

6: Ethyl 6-(4-nitrophenyl)-5-oxooctahydroindolizine-8-carboxylate

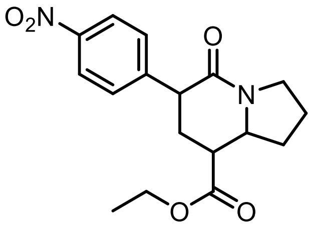

This procedure was inspired by Fukumoto *et al.*'s report.[16] To a flame-dried Schlenk flask containing a stir bar was added **2k** (33.2 mg, 0.10 mmol) and 2 mL dry DCM. The solution was cooled to 0 °C and Et_3N (13.9 µL, 0.10 mmol) followed by TMSOTf (18.3 µL, 0.10 mmol) were added dropwise while stirring. The now deeply red coloured solution was stirred at 0 °C for 4 h, upon which saturated, aqueous NH_4Cl solution (10 mL) was added to quench the reaction. The aqueous phase was extracted

[16] M. Ihara, M. Tsuruta, K. Fukumoto, T. Kametani, *J. Chem. Soc., Chem. Commun.* **1985**, 1159–1161.

with DCM (2 x 20 mL) and the combined organic phases were dried over $MgSO_4$. After filtration, the solvent was removed under reduced pressure. Purification by column chromatography on silica (10 – 60% EtOAc in heptane) afforded compound **6** in 51% yield (17.0 mg) as a yellow oil as a mixture of isomers.

^{1}H NMR (600 MHz, $CDCl_3$) [isomeric mixture]: δ 8.21 – 8.15 (m, 2H), 7.46 – 7.34 (m, 2H), 4.22 – 4.10 (m, 2H), 3.98 – 3.50 (m, 4H), 2.61 – 2.01 (m, 5H), 1.94 – 1.81 (m, 1H), 1.65 – 1.53 (m, 1H), 1.29 – 1.21 (m, 3H) ppm.

^{13}C NMR (150 MHz, $CDCl_3$) [isomeric mixture]: δ 172.6, 172.3, 171.9, 168.9, 167.7, 167.6, 148.6, 148.3, 147.13, 147.08, 130.3, 129.5, 129.4, 124.1, 124.0, 123.6, 61.4, 61.2, 60.9, 60.3, 57.5, 48.5, 46.5, 46.3, 46.1, 46.0, 45.3, 42.8, 42.0, 34.9, 33.2, 32.9, 32.8, 31.6, 29.5, 23.1, 22.4, 22.4, 14.4, 14.3, 14.3 ppm.

IR (neat) ν_{max} [isomeric mixture]: 2979, 2884, 1729, 1641, 1518, 1346, 1189, 856, 735 cm^{-1}.

HRMS (ESI$^+$): exact mass calculated for $[M+Na]^+$ ($C_{17}H_{20}N_2NaO_5^+$) requires *m/z* 355.1264, found *m/z* 355.1266.

Further experimental reaction investigations

Brønsted base comparison

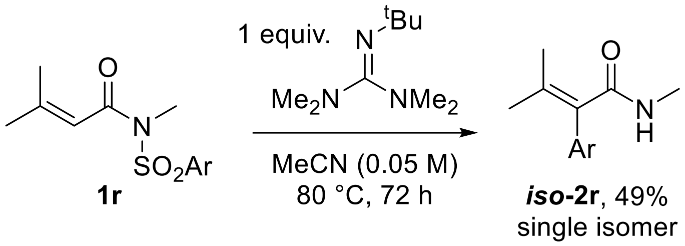

To an oven-dried 8 mL vial containing a magnetic stir bar was added **1r** (59.7 mg, 0.20 mmol). It was dissolved in 4 mL dry MeCN (0.05 M) and 2-*tert*-butyl-1,1,3,3-tetramethylguanidine (40.3 µL, 0.20 mmol) was added. The vial was capped and the mixture was stirred at 80 °C in an oil bath for 72 h, upon which it was diluted with DCM (20 mL) and the solution was transfered to a separatory funnel. The vial was washed with saturated aqueous $NaHCO_3$ solution (10 mL) and transfered to the same separatory funnel. The phases were separated and the aqueous phase was extracted with DCM (2 x 10 mL). The combined organic phases were dried over $MgSO_4$, filtered and the solvent was removed under reduced pressure. The mixture was purified via column chromatography to afford the pure product in 49% yield (22.9 mg) as a single isomer.

Observation of DABSO in the reaction mixture

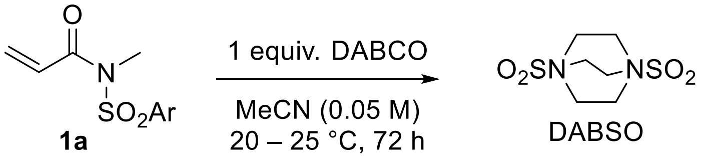

To an oven-dried 8 mL vial containing a magnetic stir bar was added **1a** (54.1 mg, 0.20 mmol). It was dissolved in 4 mL dry MeCN (0.05 M) and DABCO (22.4 mg, 0.20 mmol) was added. The vial was capped and the mixture was stirred at 80 °C in an oil bath for 72 h, upon which it was cooled to room temperature. The solution was removed using a syringe and the solid was washed with Et_2O. The residue was dissolved in CD_3OD and the ^1H NMR spectra was compared with commercial DABSO and DABCO and revealed DABSO to be present in the reaction precipitate.

^1H NMR (400 MHz, CD_3OD): δ 3.21 (s, 12H) ppm.

DABSO spectra comparison

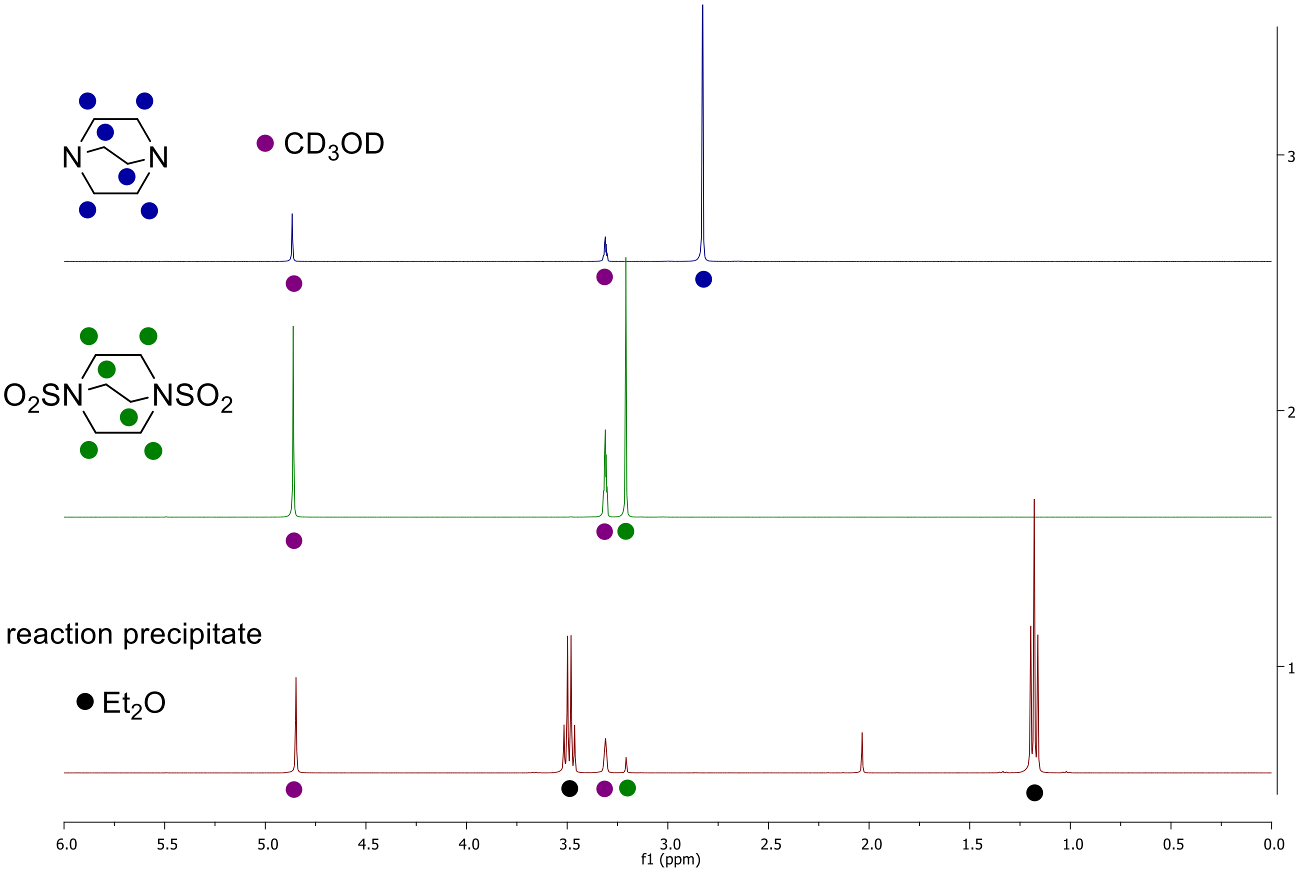

Deuterium NMR investigation

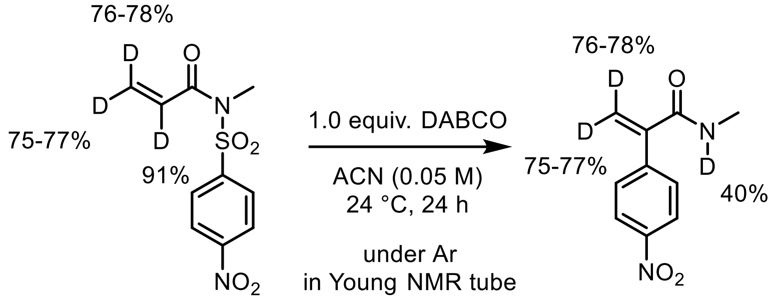

Key observation:

- 40% N–D (see **^{2}H NMR** spectrum below) and 60% N–H (see **^{1}H NMR** spectrum below) were observed after 24 h reaction time.
- An additional deuterium signal was observed at 4.1-4.3 ppm. We presume that it originates from inconsequential byproducts of the reaction.

Conclusion:

This experiment supports the proposed mechanism, consisting of a formal hydrogen/aryl exchange, as the N–D bond can only be formed through transfer from the α-position (β-deuterium integration remains constant; no other deuterium source present). The reduced deuterium incorporation at nitrogen in the final product suggests that this transposed proton is involved in H/D exchange during the reaction.

^{2}H NMR (700 MHz, MeCN)

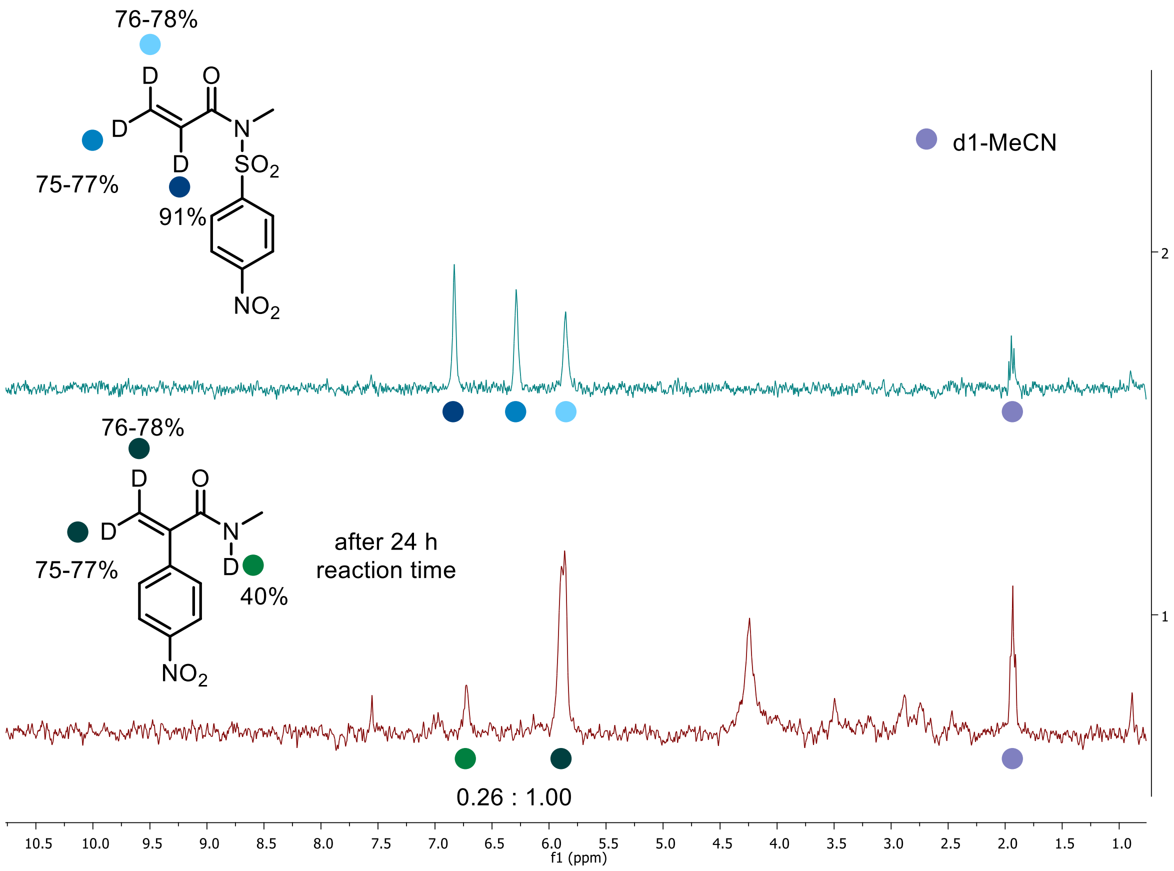

^{1}H NMR (400 MHz, MeCN)

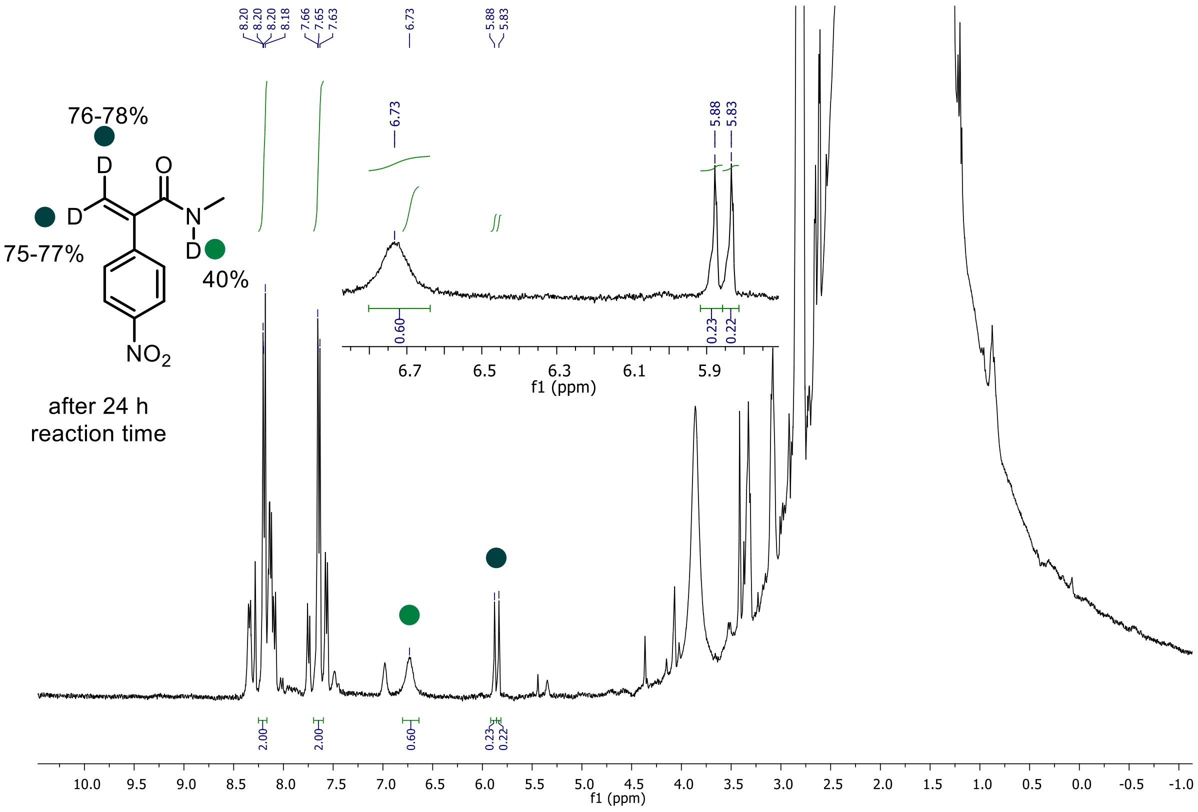

SI6: *N*-methyl-*N*-((4-nitrophenyl)sulfonyl)propiolamide

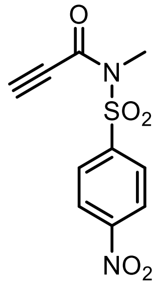

This procedure was inspired by Wu *et al.*[17] In a 100 mL round bottom flask, pivaloyl chloride (482 mg, 4.00 mmol, 2.0 equiv.) was added dropwise to a suspension of propiolic acid (280 mg, 4.00 mmol, 2.0 equiv.) and K_2CO_3 (1.67 g, 12.00 mmol, 6.0 equiv.) in 20 mL dry THF at 25 °C. The reaction mixture was stirred for 3 h, upon which the salts were filtered and rinsed with THF. The filtrate was concentrated under reduced pressure, to yield the crude pivalic propiolic anhydride as a yellow oil. In a separate flame dried 25 mL Schlenk flask, *n*-Butyllithium (0.88 mL, 2.5 mol/L in hexane, 2.20 mmol, 1.1 equiv.) was added to a solution of *N*-Methyl-*N*-((4-nitrophenyl)sulfonyl) amide (432 mg, 2.00 mmol, 1.0 equiv.) in 10 mL dry THF at – 78 °C. The reaction mixture was stirred for 1 h at 0 °C, before cooling to -78 °C and dropwise addition of freshly prepared pivalic propiolic anhydride. The cooling bath was removed, and the reaction mixture was stirred for 20 h at 25 °C. A saturated aqueous solution of NH_4Cl (25 mL) and EtOAc (25 mL) were added to the mixture. The phases were separated, and the aqueous layer was washed with EtOAc (2 x 25 ml). The combined organic phase was washed with brine (25 mL), dried over Na_2SO_4, filtered and the solvent was removed under reduced pressure. Purification by column chromatography on silica (10 – 50% EtOAc in heptane) afforded compound **SI6** as a-white solid (47 mg, 9%).

^{1}H NMR (600 MHz, $CDCl_3$): δ 8.40 (d, *J* = 8.7 Hz, 2H), 8.21 (d, *J* = 8.7 Hz, 2H), 3.56 (s, 3H), 3.37 (s, 1H) ppm.

^{13}C NMR (151 MHz, $CDCl_3$): δ 151.8, 151.1, 143.7, 130.1 (2C), 124.4 (2C), 83.0, 34.2, 31.1 ppm.

IR (neat) ν_{max}: 3268, 3109, 2960, 2921, 2860, 2111, 1670, 1607, 1532, 1368, 1182, 743 cm^{-1}.

HRMS (ESI$^+$): exact mass calculated for $[M+Na]^+$ ($C_{10}H_8N_2NaO_5S^+$) requires *m/z* 291.0047, found *m/z* 291.0046.

1a-*d*3: *N*-methyl-*N*-((4-nitrophenyl)sulfonyl)acrylamide-d3

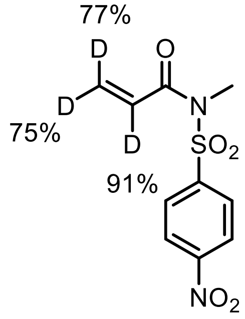

[17] J. Yan, H. W. Cheo, W. K. Teo, X. Shi, H. Wu, S. Binte Idres, L.-W. Deng, J. Wu* *J. Am. Chem. Soc.* **2020**, *142*, 11357–11362.

This procedure was inspired by Bonnesen *et al.*[18] A 12 mL oven dried vial was charged with **SI6** (45 mg, 0.17 mmol, 1.0 equiv.), Lindlar catalyst (18 mg, 5% palladium, 8 µmol, 0.05 equiv.) and quinoline (9 mg, 0.07 mmol, 0.41 equiv,) and a stirring bar. The vial was sealed with a septum cap, evacuated under reduced pressure and back filled with Ar three times, before addition of 1.7 ml MeOD. A ballon, filled with D_2, was connected to the reaction flask via syringe and needle. The reaction mixture was stirred for 16 h at 25 °C. The mixture was filtered over a membrane syringe filter, rinsed with MeOD (3 x 2 mL) and the filtrate was concentrated under reduced pressure. Purification by column chromatography on silica (10 - 30% EtOAc in heptane) afforded compound **SI7** as colourless solid (6 mg, 13 %).

^{1}H NMR (400 MHz, CDCl$_3$): δ 8.39 (d, *J* = 8.9 Hz, 2H), 8.11 (d, *J* = 9.0 Hz, 2H), 6.90 (app. s, 0.09H), 6.41 (app. s, 0.23H), 5.87 (app. s, 0.25H), 3.38 (s, 3H) ppm.

^{13}C NMR (151 MHz, CDCl$_3$): δ 165.9, 150.8, 144.6, 132.8 – 132.5 (m), 129.2 (2C), 127.6 (m), 124.6 (2C), 33.49 ppm.

IR (neat) ν_{max}: 3108, 29956, 2919, 2850, 1685, 1607, 1530, 1362, 1169, 741 cm^{-1}.

HRMS (ESI$^+$): exact mass calculated for $[M+Na]^+$ ($C_{10}H_7D_3N_2NaO_5S^+$) requires *m/z* 296.0391, found *m/z* 296.0382.

[18] J. Yang, K. Hong, P. V. Bonnesen, *J. Label. Compd. Rad.* **2011**, *54*, 743-748.

Computations

The conformational space of all molecules has been initially searched using meta-dynamics simulations based on tight-binding quantum chemical calculations as implemented in CREST.[19,20]

The structures located with CREST have then been subjected to PBE0-D3(BJ)/def2-SVP[21,22,23,24,25] geometry optimization. The nature of all stationary points (minima and transition states) was verified through the computation of the vibrational frequencies. The thermal corrections to the Gibbs free energies were combined with the single point energies calculated at the PBE0-D3(BJ)/def2-TZVP level of theory to yield Gibbs free energies ("G_{298}") at 298.15 K. All energies are reported in kcal mol^{-1}.

The DFT calculations have been performed with the Gaussian 16 program package.[26]

The polarizable continuum model (PCM) with SMD parameters[27,28] was applied to consider solvent effects for both geometries and energies. SMD parameters of acetonitrile are available in the used software package.

---

[19] P. Pracht, F. Bohle, S. Grimme, *Phys. Chem. Chem. Phys.* **2020**, *22*, 7169–7192.
[20] S. Grimme, *J. Chem. Theory Comput.* **2019**, *15*, 2847–2862.
[21] J. P. Perdew, K. Burke, M. Ernzerhof, *Phys. Rev. Lett.* **1996**, *77*, 3865–3868.
[22] J. P. Perdew, K. Burke, M. Ernzerhof, *Phys. Rev. Lett.* **1997**, *78*, 1396.
[23] C. Adamo, V. Barone, *J. Chem. Phys.* **1999**, *110*, 6158–6170.
[24] S. Grimme, J. Antony, S. Ehrlich, H. Krieg, *J. Chem. Phys.* **2010**, *132*, 154104.
[25] S. Grimme, S. Ehrlich, L. Goerigk, *J. Comput. Chem.* **2011**, *32*, 1456–1465.
[26] M. J. Frisch, *et al.*, *Gaussian 16, Revision C.01*, Gaussian, Inc., Wallingford CT, **2019**.
[27] E. Cancès, B. Mennucci, J. Tomasi, *J. Chem. Phys.* **1997**, *107*, 3032–3041.
[28] A. V. Marenich, C. J. Cramer, D. G. Truhlar, *J. Phys. Chem. B* **2009**, *113*, 6378–6396.

Overall mechanism

The calculations were performed applying the described computational approach (*vide supra*) starting from the isolated reagents: DABCO and the starting substrate *N*-sulfonylacrylamide **1a**.

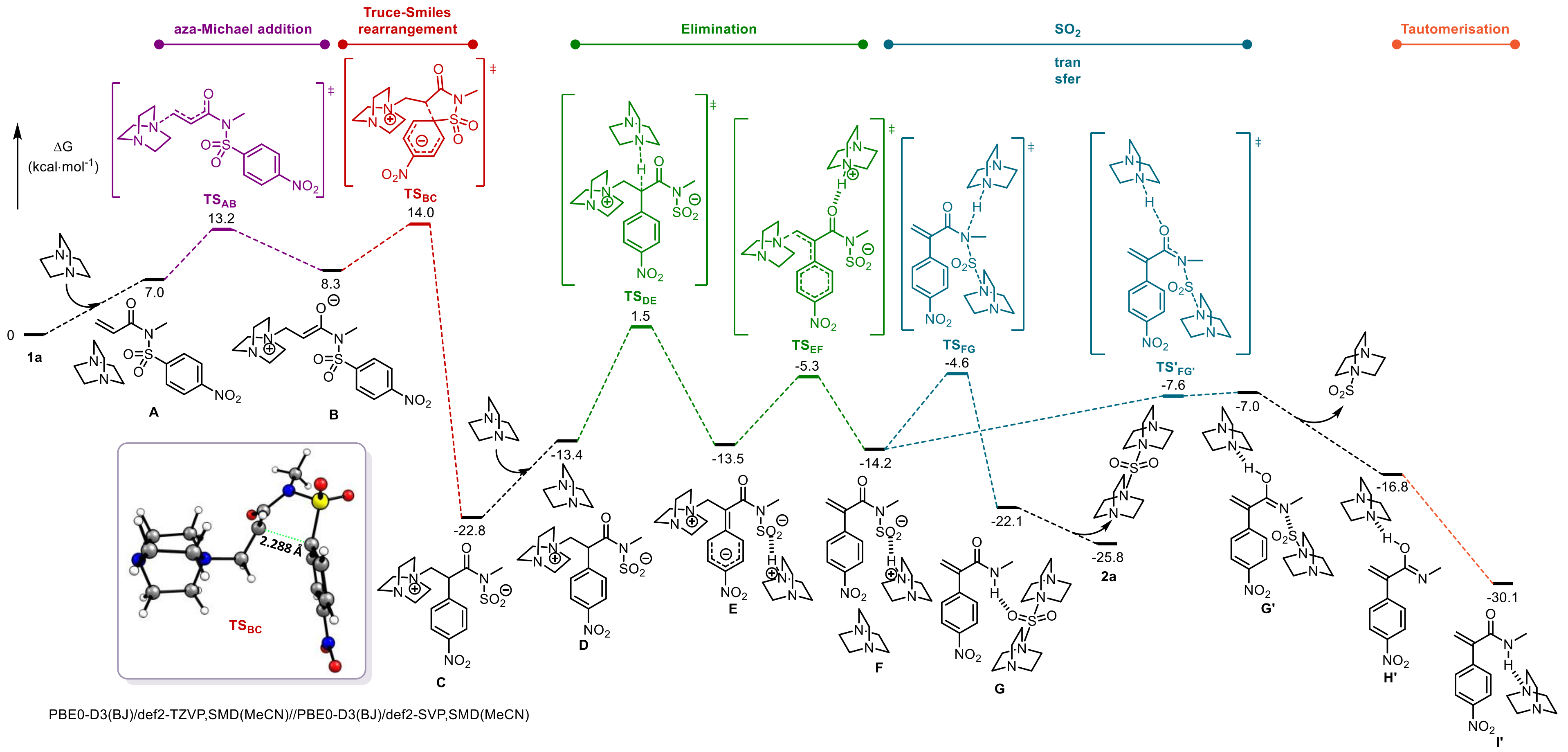

Computed scan for the spontaneous *N*-SO_2 bond dissociation

All the attempts to promote the N-S bond cleavage from intermediate **C** failed to provide a favourable pathway for the SO_2 release. As shown in one obtained energy scan, the stepwise elongation of the N–S bond resulted only in the destabilization of the system (energy increase), proving this step to be highly unfavourable.

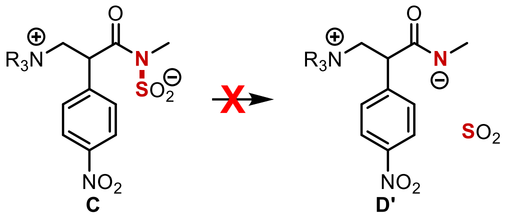

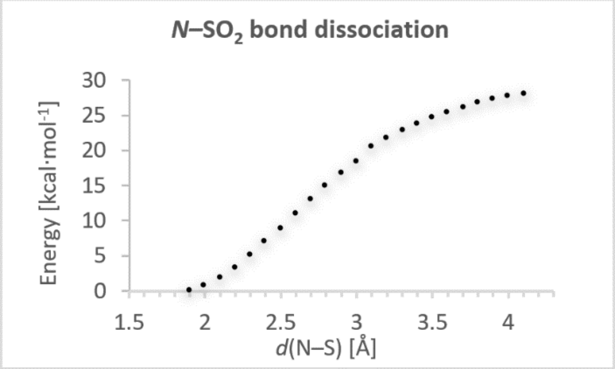

Computed scan for the direct DABCO-mediated SO_2 transfer

All the attempts to capture SO_2 from intermediate **C** mediated by DABCO failed to provide a favourable pathway. As shown in one obtained energy scan, the stepwise decrease of the DABCO SO_2 N–S distance resulted only in the destabilization of the system (energy increase), suggesting this pathway to be unfavourable.

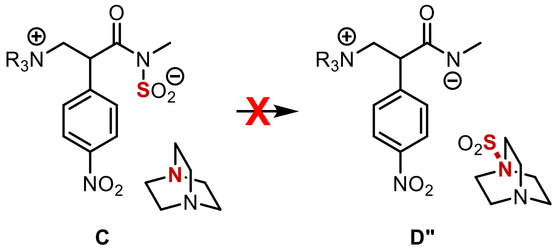

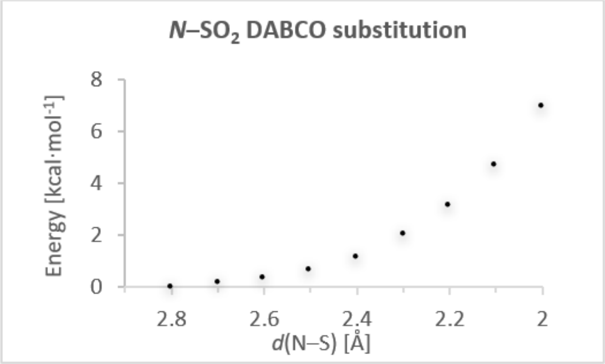

MO representation of intermediate E

Representation of the HOMO orbital of intermediate **E**, showing significant delocalization of the negative charge in the aromatic ring.

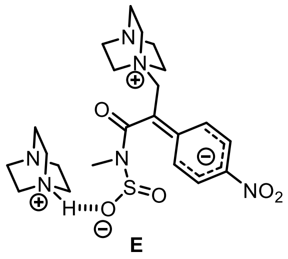

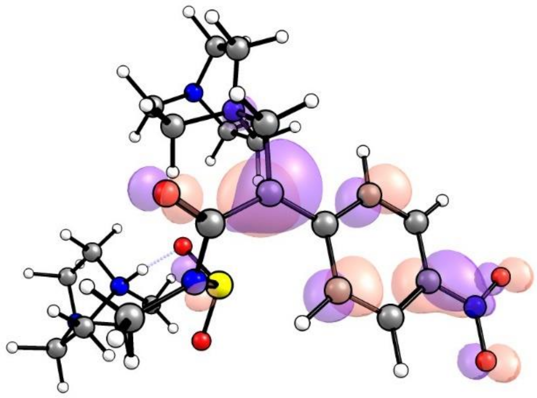

List of coordinates (xyz)

28

1a

C	2.717052000	-0.019748000	-0.184574000
C	2.267968000	0.151746000	1.119513000
C	0.948565000	0.542787000	1.322171000
C	0.131973000	0.746095000	0.213200000
C	0.590779000	0.584779000	-1.095013000
C	1.905840000	0.188481000	-1.297265000
H	2.303683000	0.041812000	-2.301256000
H	-0.066769000	0.754982000	-1.950405000
S	-1.540118000	1.298249000	0.467049000
O	-1.854780000	1.196740000	1.884727000
O	-1.710286000	2.572407000	-0.214825000
N	-2.465169000	0.173832000	-0.406569000
C	-2.431642000	-1.208294000	-0.188070000
O	-2.970325000	-1.936277000	-0.997027000
C	-1.737999000	-1.724395000	1.018157000
C	-1.417122000	-3.019161000	1.090915000
H	-0.915751000	-3.426658000	1.972651000
H	-1.648150000	-3.701076000	0.266953000
H	-1.515723000	-1.044851000	1.841509000
C	-3.176020000	0.628963000	-1.595815000
H	-3.222714000	1.721504000	-1.589279000
H	-2.669812000	0.284677000	-2.509802000
H	-4.194889000	0.221177000	-1.577497000
H	0.558046000	0.680503000	2.331664000
H	2.941072000	-0.021737000	1.958896000
N	4.108210000	-0.441397000	-0.398368000
O	4.474234000	-0.613844000	-1.540021000
O	4.806772000	-0.591710000	0.579594000

48

A

C	-0.742339000	-1.301188000	-0.662794000
C	-0.377248000	-1.568198000	0.652354000
C	0.665020000	-0.841593000	1.217180000
C	1.315420000	0.115815000	0.442262000
C	0.954891000	0.371676000	-0.882558000
C	-0.082643000	-0.356544000	-1.446268000
H	-0.395688000	-0.191179000	-2.476607000
H	1.478277000	1.125902000	-1.474682000
S	2.582250000	1.105343000	1.188380000
O	2.174350000	2.502036000	1.131849000
O	2.942092000	0.520358000	2.472813000
N	3.891110000	0.962394000	0.111676000
C	4.468524000	-0.254733000	-0.260973000
O	5.270452000	-0.262626000	-1.173523000
C	4.074437000	-1.485702000	0.469399000
C	4.351333000	-2.682126000	-0.056282000
H	4.081063000	-3.603424000	0.466371000
H	4.854357000	-2.766116000	-1.024414000
H	3.579928000	-1.399474000	1.437548000
C	4.324699000	2.142995000	-0.625094000
H	3.862017000	3.029944000	-0.183232000
H	5.417044000	2.228418000	-0.554757000
H	4.040745000	2.066308000	-1.685021000
H	0.974383000	-1.021372000	2.248068000
H	-0.909061000	-2.329072000	1.222334000
N	-1.833878000	-2.072417000	-1.260754000
O	-2.094909000	-1.872520000	-2.428591000
O	-2.388043000	-2.900746000	-0.567934000
C	-2.624640000	1.393634000	-0.146909000
N	-3.156279000	0.043265000	-0.239820000
C	-4.251312000	0.007985000	-1.195853000
H	-3.862694000	0.311247000	-2.180421000
H	-4.601023000	-1.033199000	-1.284418000
C	-5.382654000	0.958586000	-0.708263000
N	-4.983689000	1.595327000	0.541315000
C	-4.748169000	0.569625000	1.550314000
H	-4.469184000	1.063625000	2.494377000
H	-5.689333000	0.024573000	1.724274000
C	-3.624598000	-0.391266000	1.065848000
H	-2.769202000	-0.390975000	1.760579000
H	-3.983711000	-1.428561000	0.977252000
C	-3.755512000	2.351762000	0.327649000
H	-3.951711000	3.135628000	-0.420734000
H	-3.481880000	2.851723000	1.270122000
H	-5.589278000	1.743345000	-1.452970000
H	-6.319728000	0.405867000	-0.536649000
H	-1.776343000	1.387620000	0.556431000
H	-2.235236000	1.687532000	-1.134490000

48

TS_{AB}

C	-2.202771000	1.146716000	1.096938000
C	-1.297962000	0.899675000	2.125729000
C	0.002966000	1.367576000	1.990032000
C	0.353275000	2.058996000	0.830035000
C	-0.560491000	2.319920000	-0.186458000
C	-1.864243000	1.856643000	-0.051047000
H	-1.610826000	0.345734000	3.010512000
H	0.738097000	1.190084000	2.777319000
H	-0.243300000	2.860051000	-1.078068000
H	-2.609057000	2.027232000	-0.828130000
N	-3.564471000	0.616080000	1.219306000
O	-4.330901000	0.808456000	0.299909000
O	-3.845444000	0.007950000	2.229791000
S	2.049197000	2.575114000	0.654839000
O	2.517016000	2.995188000	1.973556000
O	2.165486000	3.501970000	-0.457755000
N	2.844379000	1.115643000	0.336526000
C	3.769593000	0.592694000	1.327873000
H	4.084375000	1.410850000	1.983796000
H	3.310360000	-0.198945000	1.940500000
H	4.659836000	0.195446000	0.822163000
C	2.354130000	0.357483000	-0.748333000
O	1.601032000	0.903725000	-1.548953000
C	2.749804000	-1.032747000	-0.806401000
H	3.383302000	-1.462220000	-0.030590000
C	2.204332000	-1.830554000	-1.777643000

H	1.700405000	-1.364017000	-2.627572000
H	2.565849000	-2.851149000	-1.922370000
C	0.532062000	-3.629800000	-0.138915000
C	-0.836146000	-4.182790000	0.343167000
H	1.162531000	-4.422509000	-0.569482000
H	1.096084000	-3.152067000	0.677488000
H	-0.904582000	-5.266892000	0.164790000
H	-0.972491000	-4.011761000	1.422090000
C	-0.403261000	-3.221349000	-2.300841000
H	-0.526694000	-2.449398000	-3.075651000
H	0.228970000	-4.021930000	-2.714470000
C	-1.772639000	-3.764143000	-1.803512000
H	-2.603863000	-3.270613000	-2.330069000
H	-1.855688000	-4.846268000	-1.987617000
C	-1.847364000	-2.088355000	-0.115806000
H	-2.687084000	-1.592646000	-0.627528000
H	-1.970301000	-1.922090000	0.965480000
C	-0.491841000	-1.525736000	-0.619285000
H	-0.624435000	-0.772384000	-1.407566000
H	0.090710000	-1.059891000	0.190764000
N	0.295762000	-2.624383000	-1.169078000
N	-1.920133000	-3.521673000	-0.373015000

48

B

C	5.028775000	-0.593060000	-0.156450000
C	4.414680000	0.636327000	0.071788000
C	3.100238000	0.650402000	0.518916000
C	2.440537000	-0.561523000	0.729741000
C	3.066804000	-1.787623000	0.507796000
C	4.381302000	-1.806054000	0.053312000
H	4.964856000	1.561401000	-0.100191000
H	2.590323000	1.595139000	0.711885000
H	2.534307000	-2.720180000	0.700983000
H	4.904248000	-2.744465000	-0.130927000
N	6.416235000	-0.609376000	-0.631101000
O	6.929095000	-1.687569000	-0.840936000
O	6.972104000	0.456430000	-0.787770000
S	0.734241000	-0.540705000	1.253605000
O	0.532026000	0.697586000	1.999936000
O	0.469909000	-1.825587000	1.898070000
N	-0.184177000	-0.469500000	-0.116890000
C	-0.140084000	-1.665803000	-0.946553000
H	-0.277397000	-2.555849000	-0.320025000
H	-0.972100000	-1.615144000	-1.662960000
H	0.800764000	-1.746646000	-1.516381000
C	-0.116531000	0.805111000	-0.914872000
O	0.771579000	0.854129000	-1.773955000
C	-1.099178000	1.736801000	-0.604887000
H	-1.060063000	2.665206000	-1.182505000
C	-2.082093000	1.624496000	0.478983000
H	-1.783928000	0.905002000	1.249159000
H	-2.285782000	2.590919000	0.965390000
C	-4.083578000	2.074631000	-0.948928000
C	-5.503350000	1.587493000	-1.308971000
H	-4.079759000	3.078223000	-0.503179000
H	-3.399460000	2.068252000	-1.806707000
H	-6.242558000	2.365860000	-1.071782000
H	-5.570001000	1.376208000	-2.385759000
C	-4.380475000	1.137954000	1.269619000
H	-3.903368000	0.474150000	2.002332000
H	-4.385318000	2.159152000	1.672427000
C	-5.787621000	0.656131000	0.853979000
H	-6.052697000	-0.258672000	1.402922000
H	-6.536799000	1.423516000	1.095301000
C	-4.873444000	-0.658924000	-0.896000000
H	-5.149385000	-1.580791000	-0.364354000
H	-4.923859000	-0.864094000	-1.974931000
C	-3.446535000	-0.227001000	-0.499387000
H	-3.004526000	-0.867444000	0.274297000
H	-2.756238000	-0.185453000	-1.350206000
N	-3.500461000	1.151244000	0.065861000
N	-5.837620000	0.380004000	-0.572139000

48

TS$_{BC}$

C	2.627866000	2.055332000	-0.238632000
C	2.608601000	1.300235000	0.946852000
C	2.202575000	-0.014489000	0.916192000
C	1.712361000	-0.578881000	-0.295378000
C	1.848476000	0.158155000	-1.499507000
C	2.264289000	1.472936000	-1.461683000
H	2.940061000	1.761320000	1.877860000
H	2.208924000	-0.606765000	1.834074000
H	1.575160000	-0.311292000	-2.446375000
H	2.328873000	2.064862000	-2.375291000
N	3.052443000	3.420867000	-0.202160000
O	3.067543000	4.055020000	-1.248143000
O	3.379555000	3.900824000	0.874492000
S	1.780776000	-2.375581000	-0.429148000
O	3.083384000	-2.881059000	0.006164000
O	1.321402000	-2.760677000	-1.763465000
N	0.658583000	-2.818881000	0.743888000
C	1.039270000	-3.822636000	1.716544000
H	0.161729000	-4.006131000	2.348896000
H	1.317752000	-4.758393000	1.212964000
H	1.873035000	-3.496910000	2.357904000
C	-0.287017000	-1.809242000	1.063217000
O	-0.671559000	-1.677503000	2.223394000
C	-0.532094000	-0.948300000	-0.046783000
H	-0.559378000	-1.379193000	-1.048049000
C	-1.049481000	0.409596000	0.176229000
H	-0.835029000	0.740445000	1.202116000
H	-0.600612000	1.126101000	-0.532473000
C	-2.944093000	0.381019000	-1.420667000
C	-4.477049000	0.514640000	-1.530842000
H	-2.407408000	1.133181000	-2.013793000
H	-2.584810000	-0.615627000	-1.704009000
H	-4.732164000	1.220072000	-2.334350000
H	-4.928929000	-0.456514000	-1.778173000
C	-2.903632000	2.004126000	0.389555000
H	-2.656011000	2.105011000	1.454412000
H	-2.251825000	2.671216000	-0.189263000
C	-4.401262000	2.232426000	0.101830000
H	-4.894688000	2.639149000	0.995888000
H	-4.528629000	2.957848000	-0.714253000

C	-4.814805000	-0.008376000	0.757836000
H	-5.192185000	0.385478000	1.712359000
H	-5.384085000	-0.918253000	0.520004000
C	-3.314600000	-0.345729000	0.871468000
H	-2.915818000	-0.247105000	1.888069000
H	-3.077448000	-1.353760000	0.509916000
N	-2.547874000	0.602518000	0.004293000
N	-5.044437000	0.985701000	-0.278366000

48

C

C	4.180471000	0.903442000	-0.429162000
C	3.498738000	1.543371000	0.602900000
C	2.144703000	1.283113000	0.770671000
C	1.476393000	0.395376000	-0.084964000
C	2.191801000	-0.237981000	-1.108864000
C	3.547233000	0.010715000	-1.288850000
H	4.029606000	2.231830000	1.260579000
H	1.599319000	1.757841000	1.587850000
H	1.679531000	-0.939946000	-1.770630000
H	4.113508000	-0.475519000	-2.083278000
N	5.604634000	1.175759000	-0.614370000
O	6.138617000	1.952356000	0.150198000
O	6.177666000	0.611298000	-1.523107000
S	-0.627202000	-2.892549000	-0.114660000
O	-0.726565000	-4.248436000	0.493315000
O	-1.957594000	-2.371444000	-0.587050000
N	-0.393560000	-1.871425000	1.480822000
C	-0.606138000	-2.532661000	2.737993000
H	-0.732448000	-3.605782000	2.530479000
H	0.249960000	-2.390295000	3.417802000
H	-1.510007000	-2.157478000	3.248120000
C	-0.208211000	-0.546264000	1.466315000
O	-0.178629000	0.157370000	2.475586000
C	0.002035000	0.106609000	0.089510000
H	-0.289594000	-0.575350000	-0.715876000
C	-0.791075000	1.402415000	-0.035019000
H	-0.704931000	1.980174000	0.894837000
H	-0.401392000	2.003542000	-0.866493000
C	-2.852537000	2.631661000	-0.294062000
C	-4.343473000	2.541146000	-0.668381000
H	-2.692274000	3.027216000	0.717037000
H	-2.271421000	3.230969000	-1.005807000
H	-4.949476000	3.052385000	0.092832000
H	-4.525525000	3.033976000	-1.633855000
C	-2.948361000	0.419064000	0.741442000
H	-2.567987000	-0.604413000	0.622977000
H	-2.656519000	0.819227000	1.719591000
C	-4.469190000	0.477796000	0.486535000
H	-4.872195000	-0.544288000	0.449456000
H	-4.975083000	1.012197000	1.303097000
C	-4.039220000	0.510139000	-1.847353000
H	-4.335709000	-0.547556000	-1.887708000
H	-4.322461000	0.978281000	-2.800679000
C	-2.513315000	0.620002000	-1.641366000
H	-2.049217000	-0.373899000	-1.623711000
H	-2.023120000	1.258351000	-2.387503000
N	-2.258318000	1.250946000	-0.302397000
N	-4.762148000	1.154776000	-0.764900000

68

D

C	2.172313000	2.553146000	4.117064000
C	0.836665000	2.769081000	4.440077000
C	0.136300000	3.769347000	3.776571000
C	0.757621000	4.557190000	2.798119000
C	2.108839000	4.313267000	2.500330000
C	2.821313000	3.316207000	3.148367000
H	0.355144000	2.154031000	5.200477000
H	-0.912442000	3.918016000	4.034468000
H	2.615765000	4.920437000	1.746965000
H	3.869632000	3.127248000	2.916939000
N	2.910972000	1.503671000	4.809465000
O	2.327694000	0.862651000	5.660119000
O	4.071673000	1.325501000	4.500078000
S	-0.703467000	3.231774000	0.334910000
O	-2.125944000	3.622405000	0.042252000
O	-0.159308000	2.292430000	-0.688067000
N	0.223108000	4.799526000	-0.184978000
C	0.526604000	4.918712000	-1.583750000
H	-0.079245000	5.703841000	-2.068772000
H	1.588711000	5.163846000	-1.744689000
H	0.310500000	3.946958000	-2.053303000
C	0.489468000	5.828442000	0.629676000
O	1.031816000	6.868183000	0.242373000
C	0.077333000	5.717368000	2.103455000
H	0.526432000	6.611698000	2.580737000
C	-1.422982000	5.751933000	2.371935000
H	-1.933799000	4.850831000	2.006945000
H	-1.576470000	5.832473000	3.455527000
C	-2.292748000	6.840537000	0.288666000
C	-3.333858000	7.878364000	-0.169603000
H	-1.301024000	7.095182000	-0.097926000
H	-2.529215000	5.801668000	0.017950000
H	-2.934095000	8.437644000	-1.027636000
H	-4.261556000	7.384360000	-0.492448000
C	-1.598309000	8.229367000	2.161546000
H	-1.625085000	8.289975000	3.257054000
H	-0.554882000	8.229360000	1.826352000
C	-2.425664000	9.336140000	1.474061000
H	-2.668860000	10.120663000	2.204619000
H	-1.842033000	9.798535000	0.665339000
C	-4.372015000	8.072034000	1.942482000
H	-4.523212000	8.738155000	2.803717000
H	-5.361454000	7.775224000	1.566399000
C	-3.581620000	6.821741000	2.362355000
H	-3.476974000	6.737026000	3.450806000
H	-4.015370000	5.891058000	1.974727000
N	-2.190121000	6.903389000	1.791126000
N	-3.653824000	8.798739000	0.910450000
C	2.657525000	7.837550000	6.207478000
C	1.684016000	7.131875000	5.225495000
H	3.522599000	7.196080000	6.440028000
H	2.159324000	8.079086000	7.159998000
H	2.049281000	6.131635000	4.945681000
H	0.682733000	7.006049000	5.667652000

C	3.838871000	8.773301000	4.364988000
H	4.224235000	9.714322000	3.940861000
H	4.706369000	8.133685000	4.593403000
C	2.865469000	8.069855000	3.382069000
H	2.744673000	8.640851000	2.448283000
H	3.225242000	7.065692000	3.108290000
C	1.037435000	9.252255000	4.351048000
H	0.915793000	9.829434000	3.420710000
H	0.037792000	9.130131000	4.797270000
C	2.014411000	9.954067000	5.332442000
H	2.398016000	10.895858000	4.908523000
H	1.516479000	10.198409000	6.284400000
N	3.146964000	9.077914000	5.613804000
N	1.554090000	7.930435000	4.009331000

68

TS_{DE}

C	2.204208000	2.529443000	4.657770000
C	0.921053000	2.961610000	4.994046000
C	0.277930000	3.879497000	4.182139000
C	0.873162000	4.404323000	3.007331000
C	2.183078000	3.941859000	2.713251000
C	2.836270000	3.022855000	3.514225000
H	0.438665000	2.575569000	5.892453000
H	-0.716087000	4.202508000	4.490070000
H	2.688757000	4.317406000	1.826727000
H	3.843005000	2.683416000	3.267877000
N	2.880803000	1.570146000	5.496992000
O	2.304412000	1.165547000	6.492125000
O	3.999261000	1.208539000	5.174460000
S	-0.352293000	8.339434000	0.847000000
O	0.505308000	9.423153000	0.265041000
O	-1.672915000	8.244861000	0.131740000
N	0.515173000	6.855438000	0.187339000
C	0.836673000	6.951866000	-1.213291000
H	1.894559000	6.708409000	-1.397015000
H	0.223915000	6.266795000	-1.824210000
H	0.655283000	7.988676000	-1.530756000
C	0.732893000	5.666915000	0.810971000
O	1.302361000	4.759696000	0.194831000
C	0.245218000	5.492092000	2.230993000
H	0.850370000	6.560143000	2.969247000
C	-1.227527000	5.732010000	2.433726000
H	-1.506785000	5.626822000	3.488680000
H	-1.525579000	6.742796000	2.134240000
C	-2.069175000	3.393322000	1.991533000
C	-3.051169000	2.593409000	1.110147000
H	-1.023697000	3.127408000	1.798247000
H	-2.278780000	3.273514000	3.061556000
H	-2.505623000	2.042313000	0.330826000
H	-3.585064000	1.859199000	1.730343000
C	-2.106753000	5.020778000	0.192376000
H	-2.058722000	6.101137000	-0.006798000
H	-1.158809000	4.552630000	-0.097245000
C	-3.306963000	4.328562000	-0.480579000
H	-4.014404000	5.074251000	-0.871593000
H	-2.953652000	3.723870000	-1.328296000
C	-4.634687000	4.316696000	1.476099000
H	-5.450765000	4.895461000	1.020318000
H	-5.071272000	3.669620000	2.250464000
C	-3.602096000	5.276475000	2.094941000
H	-3.719736000	6.309932000	1.743481000
H	-3.627399000	5.270491000	3.191974000
N	-2.225505000	4.846427000	1.680521000
N	-4.009066000	3.477874000	0.467184000
C	2.919290000	7.788169000	5.691602000
C	2.202839000	6.741726000	4.803683000
H	4.003390000	7.600859000	5.711084000
H	2.552058000	7.735130000	6.727526000
H	2.908538000	6.045844000	4.333028000
H	1.468099000	6.152298000	5.367180000
C	3.182843000	9.218299000	3.814509000
H	3.027406000	10.244245000	3.447940000
H	4.266403000	9.026862000	3.822677000
C	2.450992000	8.202027000	2.904989000
H	1.904695000	8.698533000	2.091632000
H	3.139179000	7.471122000	2.456904000
C	0.498851000	8.370145000	4.326519000
H	-0.058452000	8.837677000	3.505456000
H	-0.204083000	7.764066000	4.915785000
C	1.253690000	9.405059000	5.194101000
H	1.087864000	10.423951000	4.813049000
H	0.897497000	9.374940000	6.234763000
N	2.684425000	9.130823000	5.180904000
N	1.481640000	7.449507000	3.725730000

68

E

C	-2.141051000	4.517767000	-0.463801000
C	-0.745897000	4.653138000	-0.354831000
C	0.087534000	3.649808000	-0.791478000
C	-0.403033000	2.425134000	-1.366752000
C	-1.838511000	2.343408000	-1.470121000
C	-2.671288000	3.345714000	-1.038238000
H	-0.336350000	5.562498000	0.087512000
H	1.160931000	3.802584000	-0.676762000
H	-2.283516000	1.453090000	-1.914814000
H	-3.753437000	3.250744000	-1.139705000
N	-2.999711000	5.547065000	-0.007824000
O	-2.503658000	6.561032000	0.478991000
O	-4.214789000	5.394536000	-0.113814000
S	-1.139529000	-0.356077000	0.005733000
O	-2.395177000	-1.129055000	0.285635000
O	0.074113000	-1.178247000	0.492670000
N	-1.002125000	-0.546053000	-1.779739000
C	-1.420825000	-1.813873000	-2.319833000
H	-2.299688000	-2.152000000	-1.753662000
H	-1.697587000	-1.695852000	-3.377258000
H	-0.631814000	-2.584402000	-2.260779000
C	0.018578000	0.132295000	-2.415390000
O	0.558777000	-0.363483000	-3.407820000
C	0.464045000	1.411617000	-1.835189000
H	-0.231586000	-2.549878000	1.038752000
C	1.917640000	1.658325000	-2.002926000
H	2.270379000	1.237014000	-2.954673000
H	2.183845000	2.720908000	-1.967991000

C	4.233400000	1.435913000	-1.179044000
C	5.109422000	0.879130000	-0.037844000
H	4.523780000	1.052604000	-2.166371000
H	4.234988000	2.533072000	-1.212609000
H	6.005093000	0.398331000	-0.456568000
H	5.441361000	1.693905000	0.621622000
C	2.756391000	-0.493709000	-1.060533000
H	1.744144000	-0.782865000	-0.748952000
H	2.905441000	-0.751469000	-2.115403000
C	3.820300000	-1.103133000	-0.129051000
H	3.366774000	-1.905735000	0.471374000
H	4.643914000	-1.542463000	-0.710499000
C	3.270554000	0.586527000	1.434064000
H	2.651029000	-0.172586000	1.933237000
H	3.671319000	1.260150000	2.205129000
C	2.418115000	1.389254000	0.432976000
H	1.346987000	1.171481000	0.522691000
H	2.579456000	2.471388000	0.516314000
N	2.821817000	1.000705000	-0.952636000
N	4.367601000	-0.087379000	0.756156000
C	-1.824967000	-5.519166000	1.456279000
C	-1.410578000	-4.251647000	0.677430000
H	-1.513475000	-6.423783000	0.914289000
H	-2.917399000	-5.557876000	1.575826000
H	-0.857592000	-4.479273000	-0.243684000
H	-2.249423000	-3.589254000	0.427535000
C	0.241344000	-5.517953000	2.640481000
H	0.690646000	-5.547546000	3.643602000
H	0.553079000	-6.426006000	2.104523000
C	0.706152000	-4.254888000	1.877643000
H	1.365150000	-3.612577000	2.477126000
H	1.211448000	-4.489508000	0.931250000
C	-1.209704000	-3.064797000	2.791315000
H	-0.508327000	-2.456074000	3.377321000
H	-2.048711000	-2.428846000	2.480202000
C	-1.641359000	-4.353928000	3.522389000
H	-1.201455000	-4.391248000	4.529440000
H	-2.735056000	-4.388124000	3.631109000
N	-1.208591000	-5.527795000	2.776085000
N	-0.503647000	-3.467203000	1.549860000

68

TS$_{EF}$

C	1.710325000	3.195618000	5.171506000
C	0.340621000	3.470523000	5.205078000
C	-0.284056000	3.948308000	4.067246000
C	0.426820000	4.172661000	2.863372000
C	1.811283000	3.867741000	2.870138000
C	2.447517000	3.386587000	4.000502000
H	-0.216430000	3.317704000	6.129866000
H	-1.349186000	4.177013000	4.118174000
H	2.398524000	4.017965000	1.963635000
H	3.513107000	3.155717000	3.990481000
N	2.372164000	2.717373000	6.362593000
O	1.702896000	2.554104000	7.367979000
O	3.570043000	2.499390000	6.308787000
S	-0.691452000	3.621692000	-1.337879000
O	-1.952857000	4.313919000	-1.752066000
O	0.161237000	3.275751000	-2.513214000
N	0.330555000	5.103616000	-0.673046000
C	1.010338000	5.872659000	-1.678004000
H	0.753890000	6.942714000	-1.603120000
H	2.106229000	5.782897000	-1.598132000
H	0.702619000	5.488110000	-2.661925000
C	0.606706000	5.308101000	0.613286000
O	1.528469000	6.098345000	0.948039000
C	-0.229358000	4.646424000	1.652079000
H	1.750974000	6.809222000	2.311670000
C	-1.599113000	4.635645000	1.495879000
H	-2.035183000	4.789129000	0.507939000
H	-2.232319000	4.106051000	2.211765000
C	-1.908591000	7.482327000	1.066427000
C	-2.513425000	8.883448000	1.338829000
H	-0.819549000	7.470779000	1.215937000
H	-2.112636000	7.134522000	0.042872000
H	-1.722688000	9.614336000	1.568438000
H	-3.063305000	9.251070000	0.458980000
C	-2.170930000	6.900459000	3.370945000
H	-2.626862000	6.161560000	4.045873000
H	-1.079067000	6.840033000	3.486606000
C	-2.710183000	8.330836000	3.638092000
H	-3.388839000	8.336445000	4.504686000
H	-1.885764000	9.028230000	3.854206000
C	-4.516498000	7.893502000	2.150946000
H	-5.201583000	7.848503000	3.011346000
H	-5.081039000	8.293339000	1.294727000
C	-3.944199000	6.484618000	1.825695000
H	-4.352917000	5.712480000	2.495019000
H	-4.156383000	6.184932000	0.788083000
N	-2.496995000	6.524983000	2.000432000
N	-3.432664000	8.816695000	2.468571000
C	2.301005000	8.118092000	5.415303000
C	1.785218000	7.043058000	4.433003000
H	3.040631000	7.683359000	6.102868000
H	1.472202000	8.515221000	6.018832000
H	2.281211000	6.073657000	4.561835000
H	0.701351000	6.890511000	4.499811000
C	4.038439000	8.716254000	3.903086000
H	4.517963000	9.561047000	3.388165000
H	4.779050000	8.271615000	4.583198000
C	3.549577000	7.668135000	2.878945000
H	3.717782000	7.973415000	1.838122000
H	3.999722000	6.678511000	3.032040000
C	1.396027000	8.803978000	2.798948000
H	1.597021000	9.076702000	1.755000000
H	0.319388000	8.625584000	2.912292000
C	1.940179000	9.836489000	3.810262000
H	2.415821000	10.676514000	3.284171000
H	1.120212000	10.241234000	4.420926000
N	2.920826000	9.217506000	4.690028000
N	2.083143000	7.512658000	3.057073000

68

F

C	-3.382141000	-0.675881000	0.559321000
C	-3.639723000	-0.473923000	-0.796436000

C	-3.391874000	0.775639000	-1.342270000
C	-2.900294000	1.827179000	-0.548502000
C	-2.671607000	1.592243000	0.817544000
C	-2.909333000	0.345682000	1.377492000
H	-4.008636000	-1.295670000	-1.410213000
H	-3.552124000	0.931833000	-2.410721000
H	-2.291525000	2.393785000	1.453431000
H	-2.723445000	0.155900000	2.434556000
N	-3.584549000	-1.999343000	1.128734000
O	-4.079987000	-2.856305000	0.422272000
O	-3.237716000	-2.188058000	2.277951000
S	0.113504000	1.904313000	-1.016704000
O	1.393480000	2.268696000	-1.705948000
O	0.428466000	1.098613000	0.243820000
N	-0.362264000	3.480177000	-0.277156000
C	0.683554000	4.213663000	0.388902000
H	0.758231000	3.937853000	1.453851000
H	1.641127000	3.995245000	-0.107070000
H	0.476269000	5.289425000	0.314895000
C	-1.579822000	4.042929000	-0.486586000
O	-1.843331000	5.198797000	-0.181901000
C	-2.629213000	3.160404000	-1.127320000
H	1.894882000	0.729400000	0.427232000
C	-3.335089000	3.668936000	-2.148266000
H	-3.116715000	4.670787000	-2.527084000
H	-4.149012000	3.113981000	-2.622172000
C	1.648663000	-3.848774000	-0.368938000
C	0.703054000	-4.977396000	-0.859866000
H	2.258298000	-3.447126000	-1.194560000
H	2.342162000	-4.212168000	0.406539000
H	0.804757000	-5.140057000	-1.944906000
H	0.928133000	-5.932893000	-0.359512000
C	-0.056970000	-2.245115000	-0.820571000
H	-0.599716000	-1.387384000	-0.395226000
H	0.540403000	-1.861637000	-1.663492000
C	-1.011872000	-3.380285000	-1.275825000
H	-2.062140000	-3.130920000	-1.060670000
H	-0.930622000	-3.560440000	-2.359812000
C	-0.836270000	-4.414882000	0.862486000
H	-1.893998000	-4.198332000	1.079822000
H	-0.581918000	-5.356671000	1.374647000
C	0.085744000	-3.255424000	1.329351000
H	-0.502740000	-2.420647000	1.741798000
H	0.785479000	-3.587893000	2.112980000
N	0.859184000	-2.758243000	0.195084000
N	-0.682263000	-4.618505000	-0.574703000
C	4.549349000	-0.982234000	1.664757000
C	3.075536000	-0.517330000	1.643497000
H	4.610805000	-2.070243000	1.515416000
H	5.013158000	-0.749406000	2.634241000
H	2.365473000	-1.338941000	1.469011000
H	2.787005000	0.029634000	2.551044000
C	4.753451000	-0.684360000	-0.689438000
H	5.332447000	-0.173684000	-1.472655000
H	4.868264000	-1.767902000	-0.839042000
C	3.264313000	-0.286802000	-0.765275000
H	3.026815000	0.405252000	-1.583168000
H	2.596789000	-1.155175000	-0.828366000
C	3.780132000	1.608551000	0.672015000
H	3.598876000	2.252231000	-0.198500000
H	3.434317000	2.132040000	1.573297000
C	5.241115000	1.119866000	0.782670000
H	5.865254000	1.600523000	0.015436000
H	5.660591000	1.375988000	1.766369000
N	5.308640000	-0.324880000	0.608906000
N	2.923524000	0.412595000	0.498959000

68

TS$_{FG}$

C	2.258194000	-3.488775000	0.480648000
C	1.673639000	-3.667316000	-0.770807000
C	1.440622000	-2.551531000	-1.563566000
C	1.788204000	-1.263895000	-1.118405000
C	2.375410000	-1.120469000	0.150704000
C	2.616344000	-2.226802000	0.952377000
H	1.404589000	-4.668725000	-1.107024000
H	0.988521000	-2.667381000	-2.550480000
H	2.616351000	-0.123630000	0.523496000
H	3.059706000	-2.123701000	1.942890000
N	2.484961000	-4.653924000	1.329323000
O	2.147362000	-5.740744000	0.905827000
O	2.998488000	-4.476242000	2.415147000
S	-0.634958000	1.940592000	-0.891008000
O	-2.042766000	2.417869000	-0.996856000
O	-0.431199000	1.049780000	0.295553000
N	-0.828559000	0.373010000	-2.328565000
C	-1.974589000	0.458029000	-3.195748000
H	-2.724221000	1.100177000	-2.709329000
H	-1.721561000	0.888793000	-4.181349000
H	-2.427214000	-0.531513000	-3.385194000
C	0.284888000	-0.163880000	-2.847513000
O	0.371590000	-0.724704000	-3.943480000
C	1.508779000	-0.085993000	-1.959623000
H	-1.318524000	-0.736224000	-0.813196000
C	2.304987000	0.992127000	-1.992543000
H	2.061361000	1.854705000	-2.618660000
H	3.231005000	1.040735000	-1.411093000
C	1.373615000	3.662598000	0.993530000
C	1.790574000	4.858265000	1.892369000
H	1.384674000	2.706103000	1.537211000
H	2.036264000	3.556371000	0.119561000
H	2.043076000	4.520722000	2.910003000
H	2.674701000	5.373107000	1.484492000
C	-0.899687000	3.967667000	1.639866000
H	-1.917551000	4.087862000	1.238851000
H	-0.865248000	3.004828000	2.171786000
C	-0.478571000	5.156619000	2.544612000
H	-1.289753000	5.896667000	2.631986000
H	-0.230807000	4.814146000	3.561794000
C	0.372392000	6.304342000	0.642002000
H	-0.440861000	7.042601000	0.723901000
H	1.254207000	6.824995000	0.236512000
C	-0.041560000	5.113804000	-0.265743000
H	-1.066245000	5.222503000	-0.652758000
H	0.632519000	5.005676000	-1.130341000
N	0.017862000	3.886677000	0.513915000

N	0.694371000	5.818662000	1.980418000
C	-2.145001000	-2.649277000	1.838821000
C	-1.216686000	-1.721232000	1.029293000
H	-2.346375000	-2.217904000	2.829978000
H	-1.670928000	-3.630163000	1.988207000
H	-0.984716000	-0.773041000	1.524813000
H	-0.276487000	-2.207998000	0.747114000
C	-4.053942000	-1.550322000	0.947282000
H	-5.036153000	-1.708766000	0.479277000
H	-4.219977000	-1.087790000	1.930870000
C	-3.182122000	-0.636462000	0.061679000
H	-3.652268000	-0.411673000	-0.903512000
H	-2.889295000	0.310677000	0.529022000
C	-2.220714000	-2.604177000	-1.007887000
H	-2.674852000	-2.294111000	-1.957025000
H	-1.262621000	-3.095443000	-1.217062000
C	-3.165349000	-3.471618000	-0.145355000
H	-4.127661000	-3.614292000	-0.657518000
H	-2.722000000	-4.463711000	0.021843000
N	-3.409406000	-2.841352000	1.143570000
N	-1.921133000	-1.371269000	-0.234223000

68

G

C	-2.183071000	1.991226000	-1.421968000
C	-1.986804000	2.682223000	-0.228289000
C	-0.701156000	3.080428000	0.106077000
C	0.382671000	2.808197000	-0.747394000
C	0.142497000	2.138870000	-1.956796000
C	-1.135881000	1.717955000	-2.296748000
H	-1.324839000	1.164155000	-3.216006000
H	0.978246000	1.909636000	-2.620409000
C	1.752099000	3.226422000	-0.376140000
C	2.590918000	3.795115000	-1.254190000
H	3.591542000	4.094151000	-0.931045000
H	2.297767000	3.988524000	-2.289333000
C	2.253620000	3.012642000	1.033221000
O	3.144083000	3.707064000	1.510119000
N	1.683346000	1.995257000	1.706382000
C	2.115222000	1.662682000	3.037522000
H	3.181203000	1.379904000	3.067206000
H	1.979243000	2.513085000	3.724613000
H	1.516241000	0.817107000	3.398213000
H	1.057623000	1.326986000	1.241856000
H	-0.534083000	3.609782000	1.046053000
H	-2.832542000	2.885327000	0.428485000
N	-3.517458000	1.494409000	-1.736569000
O	-3.662521000	0.855242000	-2.759046000
O	-4.414285000	1.733510000	-0.952483000
S	0.200222000	-1.609125000	0.260584000
O	0.233884000	-0.298388000	0.980490000
O	-0.180281000	-1.490629000	-1.169692000
N	2.454124000	-1.551176000	-0.229888000
C	2.797492000	-2.775843000	-0.947264000
H	2.158922000	-2.829675000	-1.840884000
H	2.549371000	-3.631542000	-0.300693000
C	4.308551000	-2.732250000	-1.300384000
N	4.900394000	-1.490727000	-0.815448000
C	4.737995000	-1.414432000	0.631734000
H	5.203958000	-0.483439000	0.989706000
H	5.277013000	-2.256702000	1.091751000
C	3.231081000	-1.452289000	1.001879000
H	2.981986000	-2.318046000	1.634513000
H	2.911446000	-0.543490000	1.528672000
C	4.217164000	-0.361139000	-1.435749000
H	4.682961000	0.571905000	-1.082477000
H	4.363877000	-0.416186000	-2.525347000
C	2.708971000	-0.390323000	-1.077086000
H	2.062506000	-0.474938000	-1.962047000
H	2.401176000	0.504095000	-0.519881000
H	4.842555000	-3.579027000	-0.842768000
H	4.458101000	-2.791986000	-2.389283000
C	-2.775109000	-0.911775000	0.419760000
N	-1.961578000	-2.020977000	0.905874000
C	-2.406270000	-3.270743000	0.295478000
H	-1.750953000	-4.078649000	0.655693000
H	-2.266577000	-3.179070000	-0.791344000
C	-3.889856000	-3.507098000	0.686333000
N	-4.359232000	-2.426636000	1.545955000
C	-4.249495000	-1.161226000	0.828990000
H	-4.616730000	-0.353582000	1.480041000
H	-4.899988000	-1.195235000	-0.058213000
C	-3.531187000	-2.370966000	2.745182000
H	-3.913129000	-1.573444000	3.400694000
H	-3.630829000	-3.324676000	3.285708000
C	-2.050103000	-2.106162000	2.360224000
H	-1.385010000	-2.916014000	2.697373000
H	-1.671554000	-1.160563000	2.774582000
H	-4.006412000	-4.461185000	1.222729000
H	-4.528940000	-3.546182000	-0.209004000
H	-2.641820000	-0.866698000	-0.670627000
H	-2.365671000	0.012735000	0.850795000

25

2a

C	2.439898000	4.192692000	3.889254000
C	1.141876000	4.617768000	4.158185000
C	0.208154000	4.598135000	3.130632000
C	0.558888000	4.156893000	1.843904000
C	1.880030000	3.736684000	1.608053000
C	2.824032000	3.750447000	2.626328000
H	0.878214000	4.963836000	5.157713000
H	-0.810165000	4.942966000	3.321715000
H	2.170629000	3.382575000	0.616843000
H	3.847754000	3.418109000	2.453911000
N	3.430146000	4.213884000	4.964253000
O	3.070501000	4.591507000	6.059974000
O	4.558756000	3.851968000	4.703662000
N	0.864832000	5.489909000	-0.704479000
C	1.234947000	6.126809000	-1.939539000
H	2.323754000	6.272716000	-1.972578000
H	0.930299000	5.488395000	-2.779114000
H	0.747768000	7.109867000	-2.062342000
C	-0.177063000	4.637243000	-0.612675000
O	-0.873221000	4.331131000	-1.571786000
C	-0.461005000	4.099853000	0.771007000

H	1.341027000	5.779345000	0.142272000
C	-1.675431000	3.573883000	0.985822000
H	-2.400593000	3.540948000	0.168554000
H	-1.958831000	3.156634000	1.955481000

68

TS'$_{FG'}$

C	2.672042000	3.460892000	5.574644000
C	1.304761000	3.630458000	5.784564000
C	0.501292000	3.968694000	4.706813000
C	1.040872000	4.133258000	3.417923000
C	2.423421000	3.944761000	3.244220000
C	3.242830000	3.611421000	4.314693000
H	0.889640000	3.513612000	6.785612000
H	-0.563813000	4.135605000	4.876050000
H	2.871716000	4.069196000	2.257743000
H	4.315200000	3.468181000	4.181794000
N	3.528702000	3.124016000	6.707145000
O	3.006487000	2.990665000	7.795038000
O	4.718280000	2.994654000	6.501895000
S	-0.699401000	2.621962000	-0.570444000
O	-2.030996000	3.229114000	-0.817768000
O	0.084871000	2.419532000	-1.813559000
N	0.446806000	4.717648000	-0.094714000
C	1.019509000	5.331420000	-1.257005000
H	0.723920000	6.392379000	-1.352064000
H	2.124485000	5.310961000	-1.245148000
H	0.675425000	4.785590000	-2.149345000
C	0.810680000	5.125026000	1.070392000
O	1.701530000	6.058262000	1.244502000
C	0.178610000	4.481421000	2.268816000
H	1.793997000	6.616470000	2.238219000
C	-1.138018000	4.211180000	2.244840000
H	-1.734627000	4.458733000	1.364489000
H	-1.639847000	3.706637000	3.074617000
C	-1.008325000	-1.903587000	-0.409172000
C	-0.424692000	-0.465010000	-0.400062000
H	-0.761463000	-2.432797000	0.523902000
H	-0.595230000	-2.488839000	-1.244931000
H	0.098283000	-0.238291000	0.541815000
H	0.277307000	-0.289959000	-1.228245000
C	-3.023683000	-1.100309000	0.566667000
H	-4.119920000	-1.092385000	0.467133000
H	-2.776596000	-1.621436000	1.504220000
C	-2.459132000	0.345849000	0.571744000
H	-3.244527000	1.104834000	0.444366000
H	-1.914561000	0.574245000	1.501104000
C	-2.221285000	0.255110000	-1.807017000
H	-3.002572000	1.024040000	-1.898146000
H	-1.495655000	0.415444000	-2.618114000
C	-2.798722000	-1.184879000	-1.799351000
H	-3.894525000	-1.168778000	-1.902941000
H	-2.392466000	-1.775029000	-2.635075000
N	-2.459256000	-1.853102000	-0.547775000
N	-1.527588000	0.479938000	-0.543645000
C	1.736424000	8.523603000	5.419755000
C	1.391093000	7.332407000	4.493084000
H	2.197092000	8.166131000	6.353108000
H	0.830517000	9.087392000	5.689124000
H	1.746909000	6.380359000	4.906403000
H	0.310594000	7.242479000	4.310799000
C	3.868807000	8.698301000	4.380064000
H	4.581365000	9.401155000	3.922501000
H	4.333932000	8.299273000	5.294149000
C	3.518349000	7.554574000	3.397884000
H	3.990032000	7.692054000	2.414208000
H	3.816589000	6.569153000	3.783776000
C	1.622714000	8.814602000	2.608559000
H	2.089434000	8.903764000	1.617311000
H	0.533776000	8.759597000	2.465596000
C	2.032109000	9.966283000	3.556118000
H	2.740614000	10.647450000	3.060980000
H	1.153251000	10.557604000	3.854436000
N	2.664005000	9.428572000	4.753852000
N	2.058617000	7.533712000	3.196322000

68

G'

C	2.629141000	3.505092000	5.638233000
C	1.260593000	3.697666000	5.816318000
C	0.484469000	4.026735000	4.715902000
C	1.052717000	4.156482000	3.435807000
C	2.436031000	3.947122000	3.294338000
C	3.228599000	3.624544000	4.388160000
H	0.823311000	3.606161000	6.810502000
H	-0.581612000	4.211038000	4.858991000
H	2.906897000	4.041925000	2.314821000
H	4.301452000	3.464368000	4.280839000
N	3.457373000	3.180276000	6.795331000
O	2.911238000	3.079944000	7.874928000
O	4.648501000	3.027773000	6.617260000
S	-0.688957000	2.543263000	-0.597753000
O	-1.999518000	3.196940000	-0.836280000
O	0.095609000	2.341710000	-1.840002000
N	0.545472000	4.658957000	-0.101475000
C	1.146387000	5.249339000	-1.262712000
H	0.885487000	6.318405000	-1.364979000
H	2.249808000	5.193423000	-1.239602000
H	0.794877000	4.709467000	-2.155268000
C	0.872852000	5.089151000	1.059674000
O	1.768597000	6.035131000	1.228836000
C	0.216287000	4.483587000	2.261360000
H	1.814346000	6.519101000	2.177027000
C	-1.100123000	4.216148000	2.212827000
H	-1.676197000	4.445291000	1.314005000
H	-1.620709000	3.730837000	3.042374000
C	-1.078713000	-1.942325000	-0.451738000
C	-0.466700000	-0.515888000	-0.435047000
H	-0.846555000	-2.478196000	0.481134000
H	-0.671964000	-2.532340000	-1.287064000
H	0.053757000	-0.300956000	0.510784000
H	0.242752000	-0.351567000	-1.258823000
C	-3.083309000	-1.103124000	0.515146000
H	-4.178536000	-1.073986000	0.410232000
H	-2.850343000	-1.630086000	1.452882000
C	-2.493028000	0.332543000	0.527463000

H	-3.263096000	1.106076000	0.395650000
H	-1.949367000	0.549035000	1.459989000
C	-2.242376000	0.242288000	-1.852109000
H	-3.008061000	1.026321000	-1.945376000
H	-1.508129000	0.389763000	-2.657645000
C	-2.847202000	-1.186403000	-1.849452000
H	-3.941742000	-1.147990000	-1.958736000
H	-2.447847000	-1.781760000	-2.684610000
N	-2.527254000	-1.863431000	-0.597934000
N	-1.551739000	0.450691000	-0.583024000
C	1.919837000	8.501617000	5.503518000
C	1.570795000	7.285279000	4.609391000
H	2.519532000	8.187756000	6.371730000
H	1.007529000	8.984308000	5.886413000
H	2.037926000	6.363551000	4.980550000
H	0.486407000	7.113089000	4.548969000
C	3.894815000	8.864074000	4.229270000
H	4.479626000	9.625042000	3.690014000
H	4.499698000	8.522804000	5.083364000
C	3.536188000	7.679988000	3.297716000
H	3.892575000	7.841771000	2.269584000
H	3.961134000	6.730388000	3.655812000
C	1.470376000	8.762761000	2.720425000
H	1.821255000	8.893730000	1.686181000
H	0.380612000	8.614617000	2.688471000
C	1.868646000	9.954546000	3.624324000
H	2.448783000	10.700169000	3.059137000
H	0.976235000	10.460277000	4.023824000
N	2.679082000	9.485089000	4.741292000
N	2.073826000	7.529904000	3.251329000

45

H'

C	2.823409000	-0.189929000	1.067748000
C	2.729685000	-0.384787000	-0.308532000
C	1.640048000	-1.080637000	-0.811302000
C	0.645196000	-1.589609000	0.042626000
C	0.773690000	-1.370938000	1.425712000
C	1.857345000	-0.674961000	1.944383000
H	3.497537000	0.018662000	-0.968628000
H	1.551007000	-1.214262000	-1.890877000
H	0.012882000	-1.748462000	2.110913000
H	1.959816000	-0.504562000	3.016129000
N	3.959324000	0.554778000	1.603037000
O	4.786087000	0.971760000	0.817970000
O	4.017613000	0.717083000	2.804607000
N	-2.362204000	-3.497000000	0.506903000
C	-3.594702000	-3.454486000	1.253665000
H	-3.420278000	-3.232935000	2.323341000
H	-4.292351000	-2.677807000	0.889392000
H	-4.101974000	-4.430094000	1.199084000
C	-1.766775000	-2.381290000	0.319905000
O	-2.199914000	-1.235317000	0.817423000
C	-0.505473000	-2.352148000	-0.489086000
H	-1.824664000	-0.375299000	0.349775000
C	-0.454205000	-3.028525000	-1.648198000
H	-1.330111000	-3.579230000	-1.999224000
H	0.451806000	-3.069105000	-2.258284000
C	-2.868318000	3.086829000	-0.686602000
C	-2.896781000	1.675542000	-0.050182000
H	-3.026000000	3.865653000	0.075376000
H	-3.662422000	3.194425000	-1.441555000
H	-3.046383000	1.719056000	1.039042000
H	-3.695374000	1.048260000	-0.473458000
C	-0.519965000	3.206393000	-0.335698000
H	0.444147000	3.411257000	-0.826410000
H	-0.674177000	3.981646000	0.430654000
C	-0.526385000	1.791277000	0.295089000
H	0.418035000	1.261438000	0.111543000
H	-0.688658000	1.824242000	1.382719000
C	-1.399731000	0.895364000	-1.752242000
H	-0.452418000	0.363333000	-1.917979000
H	-2.208999000	0.277231000	-2.169071000
C	-1.376089000	2.316215000	-2.369203000
H	-0.411323000	2.513948000	-2.861440000
H	-2.166041000	2.430351000	-3.127533000
N	-1.580809000	3.318039000	-1.329773000
N	-1.612584000	1.003867000	-0.302852000

45

I'

C	0.710761000	-3.936515000	-0.061012000
C	0.986206000	-2.954831000	-1.009567000
C	1.181003000	-1.649182000	-0.581526000
C	1.094123000	-1.312673000	0.781007000
C	0.823109000	-2.331640000	1.709616000
C	0.628380000	-3.642620000	1.297798000
H	0.423401000	-4.437492000	2.015037000
H	0.780214000	-2.097270000	2.775051000
C	1.249713000	0.092885000	1.208973000
C	0.633933000	0.597055000	2.290453000
H	-0.035493000	-0.007046000	2.909088000
H	0.788209000	1.637293000	2.587412000
C	2.193439000	0.948202000	0.400489000
O	3.287132000	0.511436000	0.052649000
N	1.754907000	2.190884000	0.140948000
H	0.741749000	2.404843000	0.266035000
C	2.560716000	3.122237000	-0.601873000
H	2.256200000	4.149960000	-0.359063000
H	2.464477000	2.983551000	-1.693940000
H	3.620980000	2.994176000	-0.341929000
H	1.397995000	-0.875191000	-1.319667000
H	1.043508000	-3.219660000	-2.065379000
N	0.510129000	-5.313555000	-0.502910000
O	0.589650000	-5.546603000	-1.691673000
O	0.272924000	-6.152951000	0.341436000
C	-3.377959000	3.081806000	0.719795000
N	-3.421923000	3.143419000	-0.737318000
C	-2.932746000	1.883925000	-1.288457000
H	-2.993034000	1.935981000	-2.387019000
H	-3.601648000	1.073717000	-0.957885000
C	-1.476728000	1.632082000	-0.818383000
N	-1.041267000	2.741865000	0.031634000
C	-1.108414000	3.990895000	-0.729778000
H	-0.416984000	3.911352000	-1.583235000
H	-0.748526000	4.807138000	-0.084272000

C	-2.567581000	4.232570000	-1.197218000
H	-2.629159000	4.286674000	-2.295591000
H	-2.961232000	5.179838000	-0.796173000
C	-1.921912000	2.834097000	1.195907000
H	-1.559601000	3.650347000	1.840097000
H	-1.837082000	1.896644000	1.766496000
H	-1.393204000	0.701440000	-0.236306000
H	-0.779322000	1.554748000	-1.667471000
H	-4.047628000	2.274653000	1.056355000
H	-3.772349000	4.028423000	1.121782000

20

DABCO

C	-6.092076000	3.992044000	0.083156000
C	-6.402519000	5.451283000	-0.344766000
H	-6.357227000	3.275670000	-0.710811000
H	-6.658194000	3.708841000	0.984924000
H	-6.829122000	5.490100000	-1.359840000
H	-7.128589000	5.923675000	0.336064000
C	-3.902029000	4.190847000	-0.825746000
H	-2.831413000	4.057094000	-0.602671000
H	-4.165070000	3.474021000	-1.620037000
C	-4.212698000	5.649786000	-1.254675000
H	-3.302671000	6.270916000	-1.254402000
H	-4.637249000	5.688060000	-2.270636000
C	-4.614215000	6.231818000	1.017478000
H	-3.704349000	6.853134000	1.020036000
H	-5.338581000	6.705593000	1.699192000
C	-4.304330000	4.772740000	1.446262000
H	-3.234107000	4.638803000	1.671201000
H	-4.868719000	4.491096000	2.349600000
N	-4.667596000	3.854906000	0.370823000
N	-5.175058000	6.241410000	-0.330166000

23

DABCO·SO_2

S	-0.737956000	2.442178000	-0.618445000
O	-2.016099000	3.157504000	-0.828710000
O	0.082724000	2.279958000	-1.839034000
C	-1.074008000	-1.931587000	-0.448348000
C	-0.455273000	-0.508175000	-0.431822000
H	-0.842077000	-2.462840000	0.486430000
H	-0.663619000	-2.520166000	-1.282019000
H	0.063688000	-0.292296000	0.513690000
H	0.248320000	-0.343116000	-1.259495000
C	-3.080304000	-1.094675000	0.513700000
H	-4.174282000	-1.060609000	0.404352000
H	-2.850685000	-1.620036000	1.452371000
C	-2.491956000	0.341451000	0.536884000
H	-3.260385000	1.115507000	0.403965000
H	-1.945154000	0.550981000	1.468063000
C	-2.239405000	0.254857000	-1.853408000
H	-3.007325000	1.036717000	-1.940610000
H	-1.502845000	0.403698000	-2.655737000
C	-2.838586000	-1.176051000	-1.847886000
H	-3.931984000	-1.135791000	-1.960813000
H	-2.433839000	-1.765955000	-2.683397000
N	-2.520307000	-1.851195000	-0.597448000
N	-1.546715000	0.460378000	-0.577915000

43

$(DABCO)_2$-SO_2

S	0.195830000	-1.616819000	0.209876000
O	0.230799000	-0.282783000	0.865667000
O	-0.179670000	-1.564267000	-1.227796000
N	2.470521000	-1.572418000	-0.259617000
C	2.827457000	-2.744349000	-1.051583000
H	2.201091000	-2.741600000	-1.955482000
H	2.573595000	-3.642200000	-0.467016000
C	4.343716000	-2.673983000	-1.377985000
N	4.926083000	-1.473269000	-0.789119000
C	4.735282000	-1.504376000	0.656703000
H	5.201616000	-0.607472000	1.092618000
H	5.259349000	-2.383638000	1.061804000
C	3.220388000	-1.559065000	0.991642000
H	2.956260000	-2.465338000	1.558453000
H	2.886041000	-0.687543000	1.573189000
C	4.256192000	-0.297970000	-1.335757000
H	4.715669000	0.602409000	-0.899611000
H	4.429405000	-0.268006000	-2.422544000
C	2.738889000	-0.355551000	-1.017474000
H	2.117607000	-0.385275000	-1.924836000
H	2.401358000	0.496405000	-0.409007000
H	4.872525000	-3.553643000	-0.979932000
H	4.510571000	-2.648287000	-2.465874000
C	-2.821378000	-0.949803000	0.350400000
N	-1.989586000	-2.021512000	0.885849000
C	-2.429458000	-3.308165000	0.357600000
H	-1.752060000	-4.085971000	0.742908000
H	-2.319665000	-3.276404000	-0.736260000
C	-3.898215000	-3.545900000	0.801005000
N	-4.370148000	-2.414719000	1.591863000
C	-4.288889000	-1.200395000	0.787117000
H	-4.670964000	-0.357501000	1.383434000
H	-4.946015000	-1.314780000	-0.088724000
C	-3.525216000	-2.265344000	2.771446000
H	-3.903477000	-1.422420000	3.370088000
H	-3.611883000	-3.175692000	3.384321000
C	-2.052650000	-2.023678000	2.343234000
H	-1.381575000	-2.814293000	2.713267000
H	-1.663579000	-1.057693000	2.696723000
H	-3.982118000	-4.459760000	1.409015000
H	-4.557892000	-3.664133000	-0.072364000
H	-2.699160000	-0.948401000	-0.742780000
H	-2.423617000	0.002105000	0.732204000

26

DABSO

S	0.071437000	-1.634283000	0.310307000
O	0.189008000	-0.313356000	0.958731000
O	-0.166905000	-1.589561000	-1.145942000
C	-2.798693000	-0.926194000	0.335431000
N	-1.999734000	-2.019141000	0.892754000
C	-2.431956000	-3.303370000	0.337290000
H	-1.775373000	-4.089621000	0.734978000
H	-2.306074000	-3.260014000	-0.752607000

C	-3.906749000	-3.540630000	0.738952000	H	-1.470064000	-2.840997000	2.736931000
N	-4.370034000	-2.383764000	1.507740000	H	-1.715667000	-1.073037000	2.727830000
C	-4.281531000	-1.163721000	0.702632000	H	-4.021765000	-4.433110000	1.368078000
H	-4.694058000	-0.335884000	1.294594000	H	-4.551496000	-3.648428000	-0.144286000
H	-4.901595000	-1.298641000	-0.194402000	H	-2.640564000	-0.913738000	-0.751597000
C	-3.586629000	-2.240006000	2.736070000	H	-2.416824000	0.015067000	0.753836000
H	-3.992827000	-1.385827000	3.294789000	S	-6.441140000	-2.704721000	2.133820000
H	-3.731314000	-3.149059000	3.335723000	O	-6.579749000	-1.456079000	2.908918000
C	-2.103920000	-2.029543000	2.353336000	O	-6.187521000	-3.911718000	2.945081000

NMR spectra

0f: *N*-(4,4-Dimethoxybutyl)-4-nitrobenzenesulfonamide

^{1}H NMR (400 MHz, $CDCl_3$)

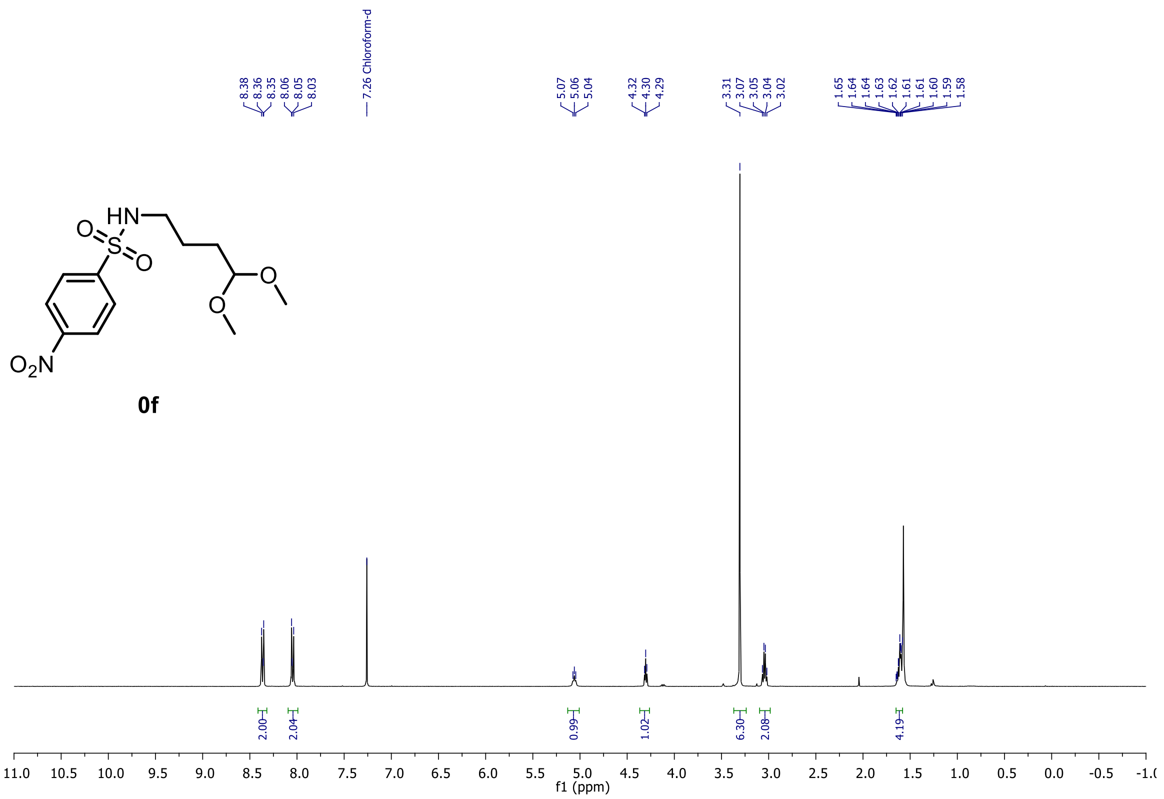

^{13}C NMR (150 MHz, $CDCl_3$)

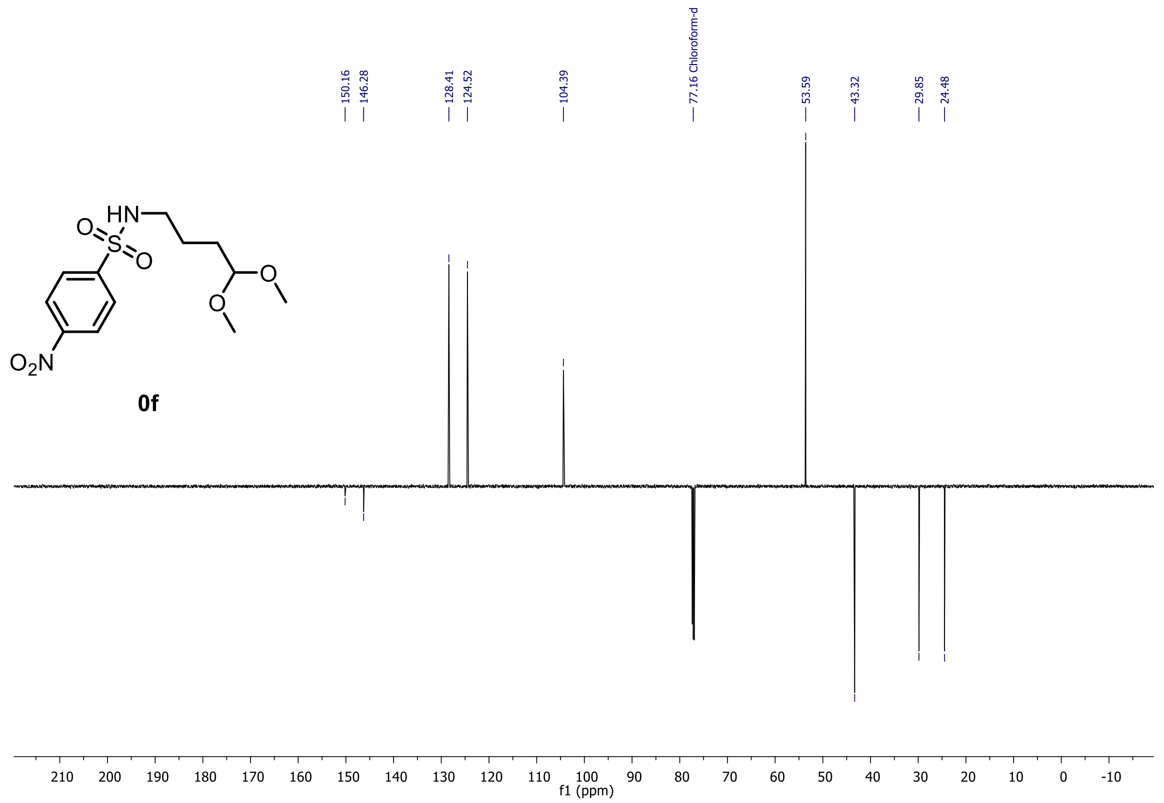

0j: *tert*-Butyl 3-(2-((4-nitrophenyl)sulfonamido)ethyl)-1*H*-indole-1-carboxylate

^{1}H NMR (400 MHz, $CDCl_3$)

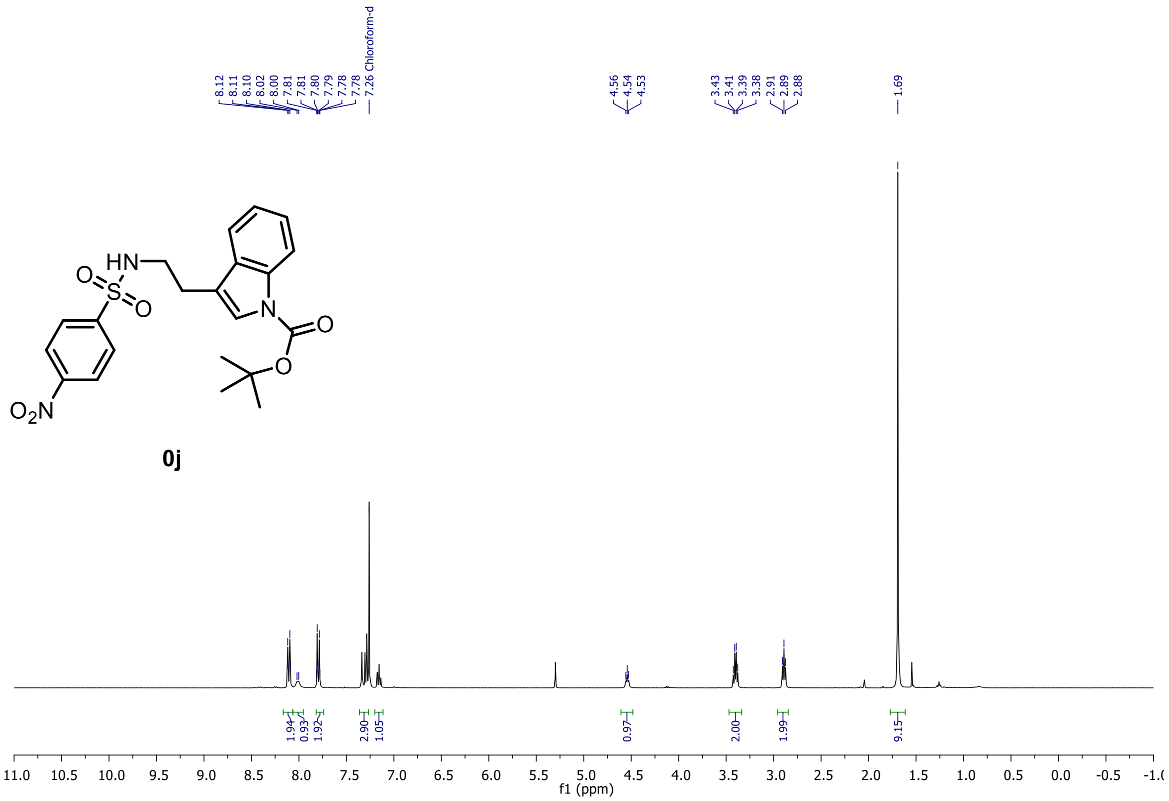

^{13}C NMR (100 MHz, $CDCl_3$)

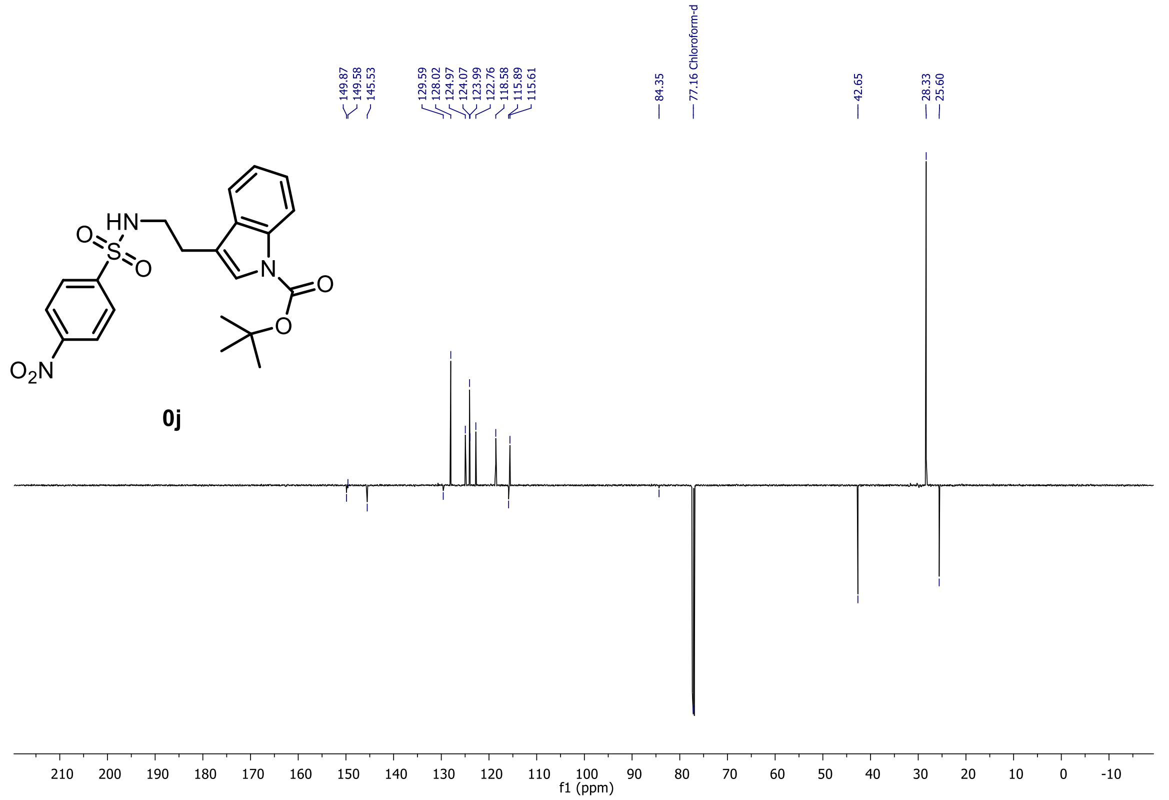

0k: Ethyl-(*E*)-6-((4-nitrophenyl)sulfonamido)hex-2-enoate

^{1}H NMR (400 MHz, $CDCl_3$)

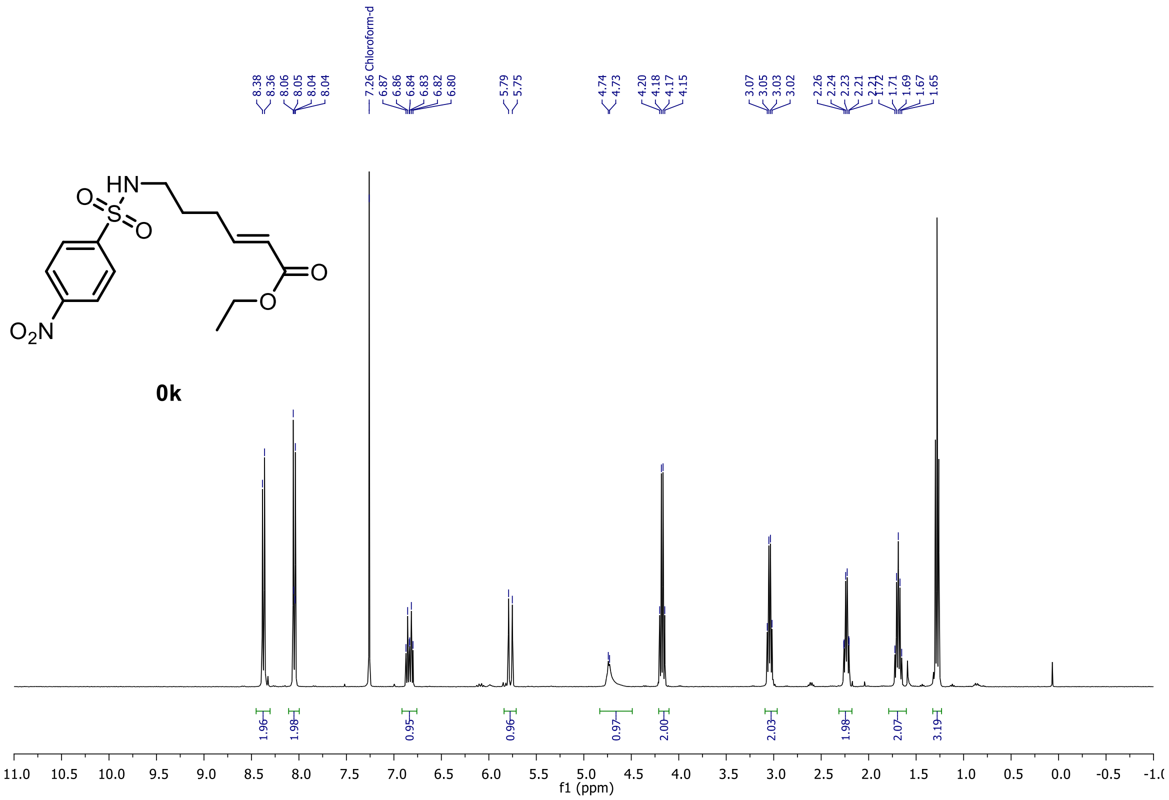

^{13}C NMR (100 MHz, $CDCl_3$)

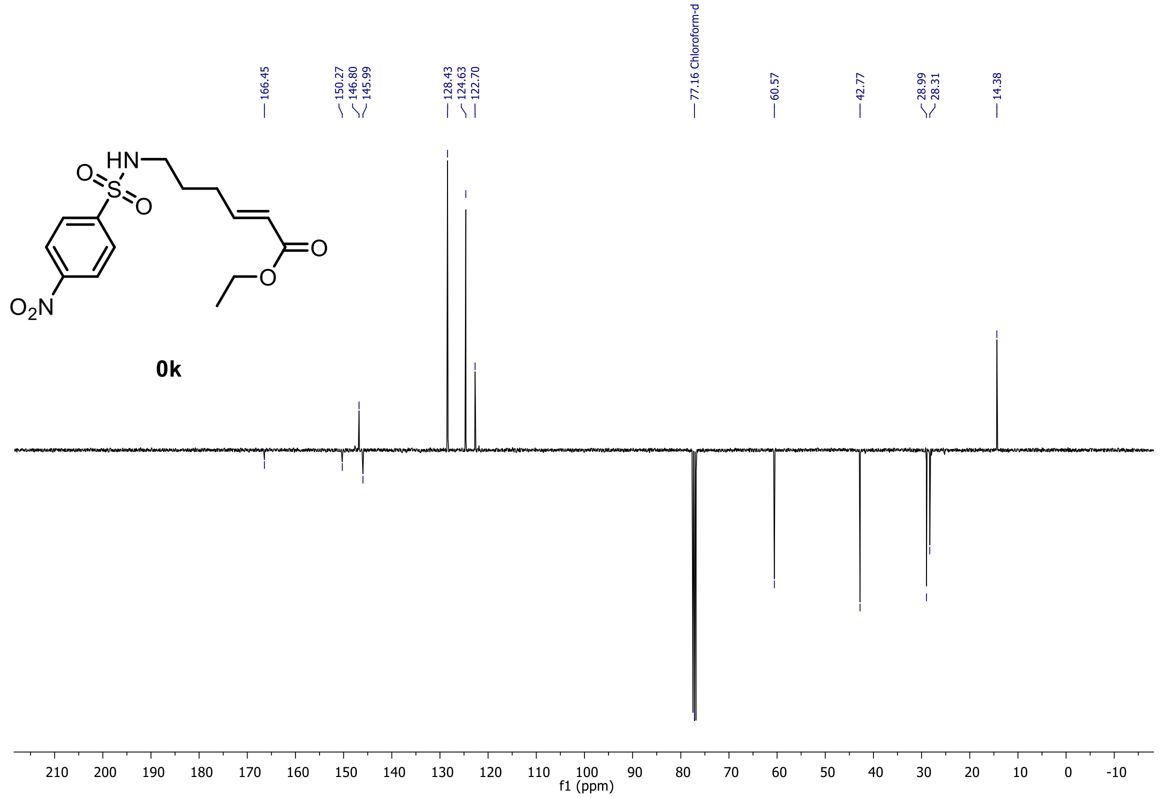

0n: *N*-Methyl-2,5-bis(trifluoromethyl)benzenesulfonamide

^{1}H NMR (400 MHz, $CDCl_3$)

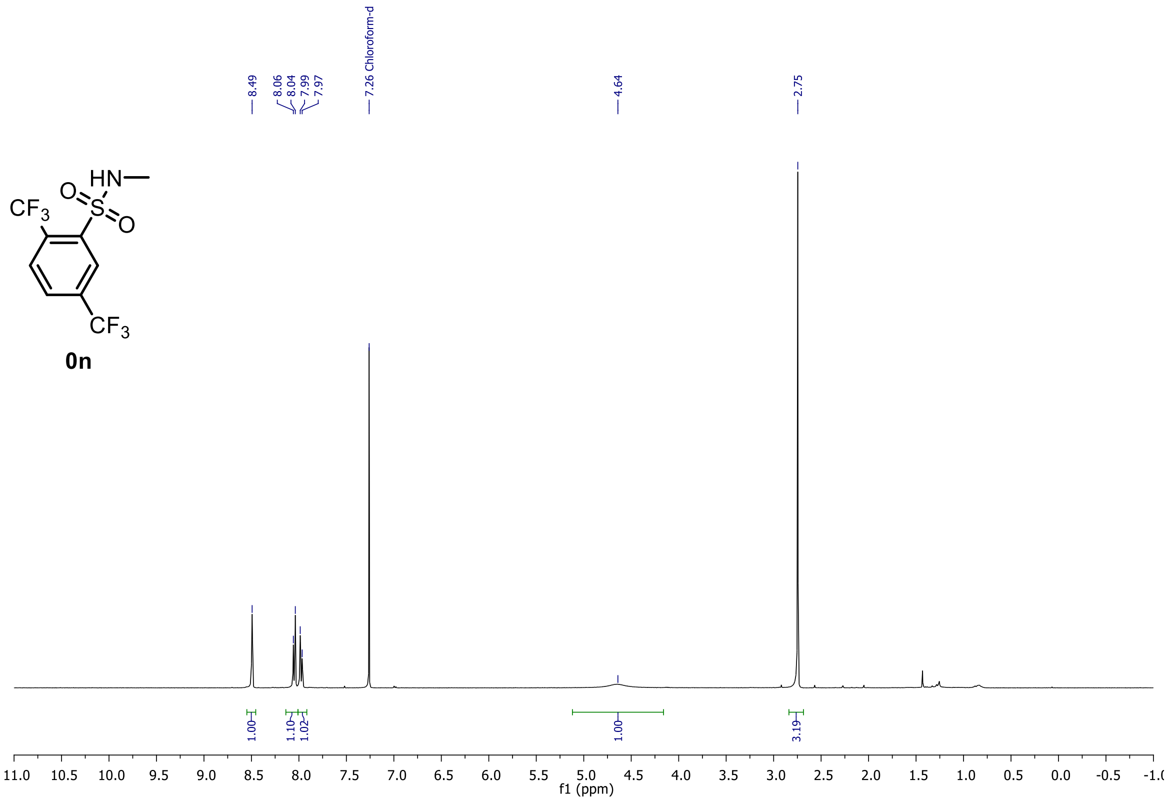

^{13}C NMR (100 MHz, $CDCl_3$)

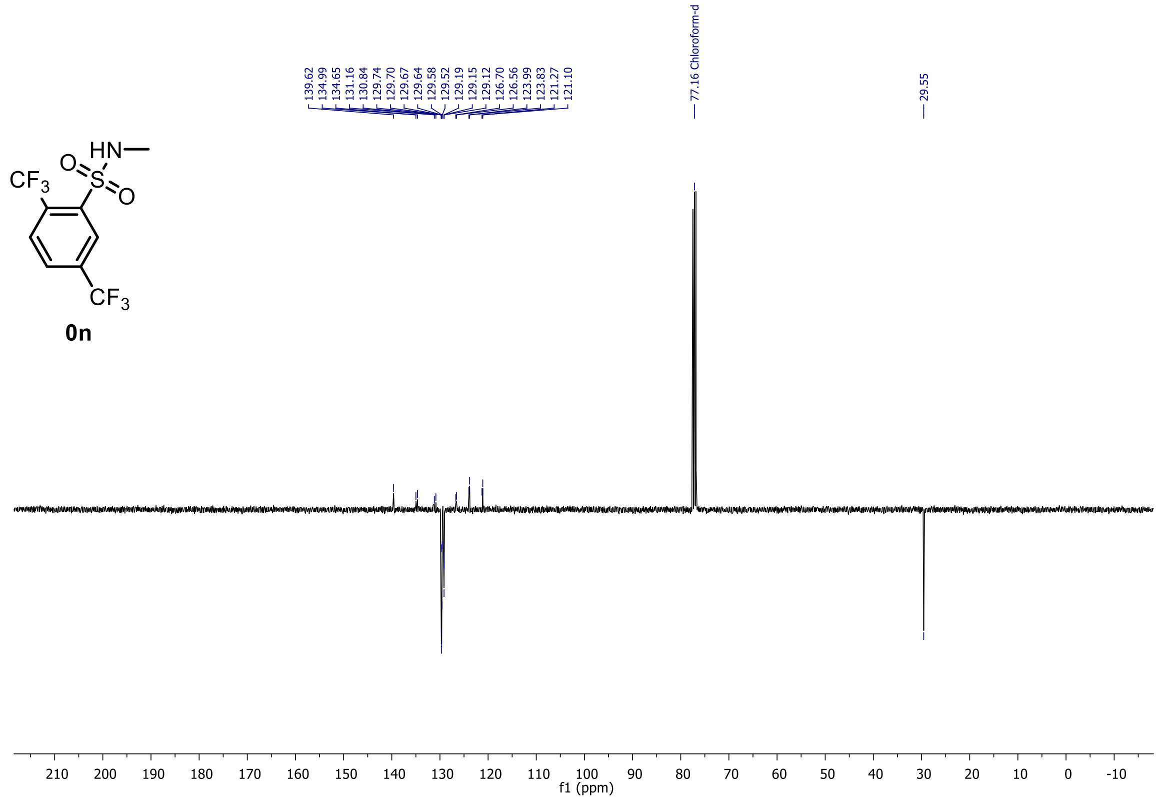

^{19}F NMR (377 MHz, $CDCl_3$)

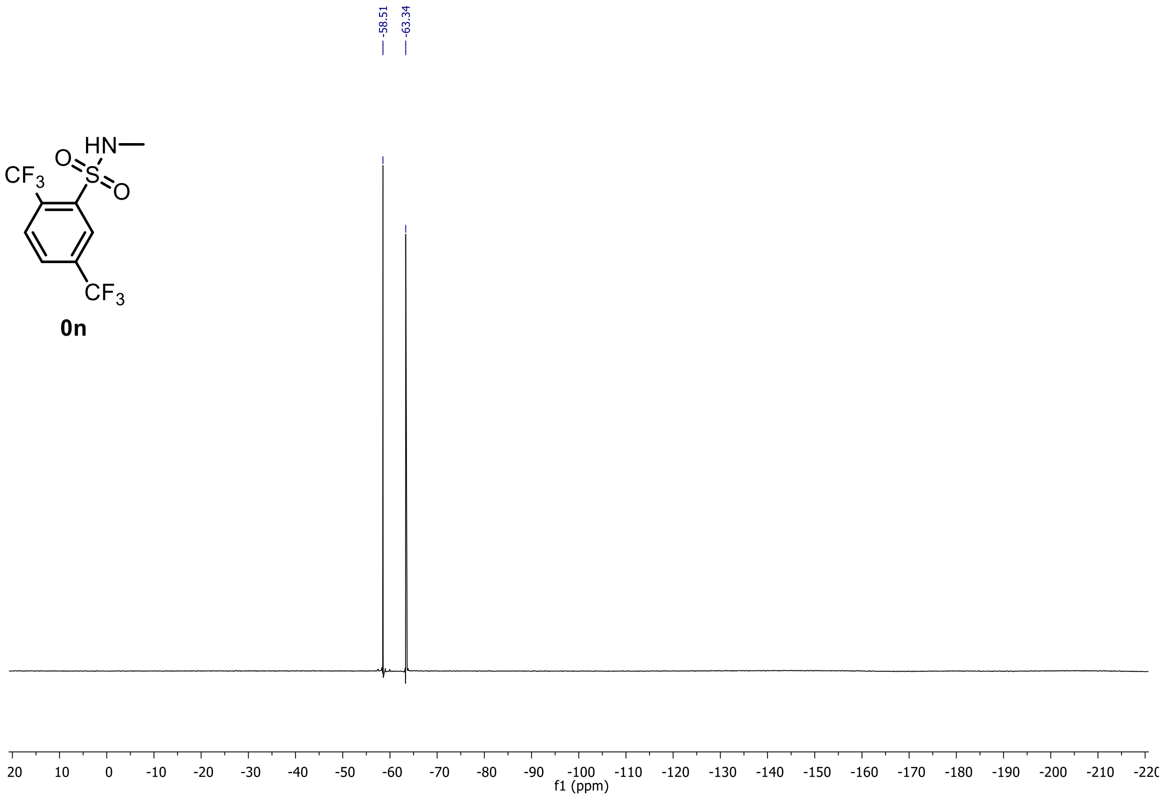

0o: *N*-4-Cyano-*N*-methyl-2-(trifluoromethyl)benzenesulfonamide

^{1}H NMR (400 MHz, $CDCl_3$)

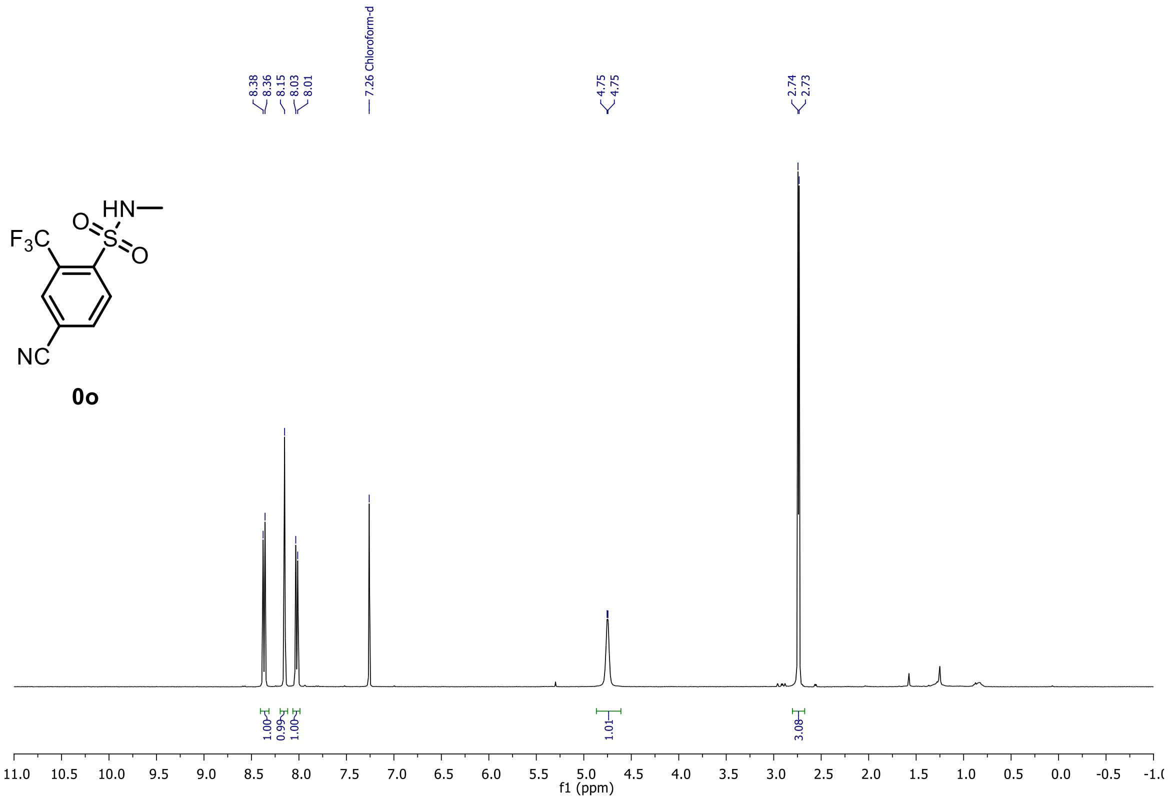

^{13}C NMR (100 MHz, $CDCl_3$)

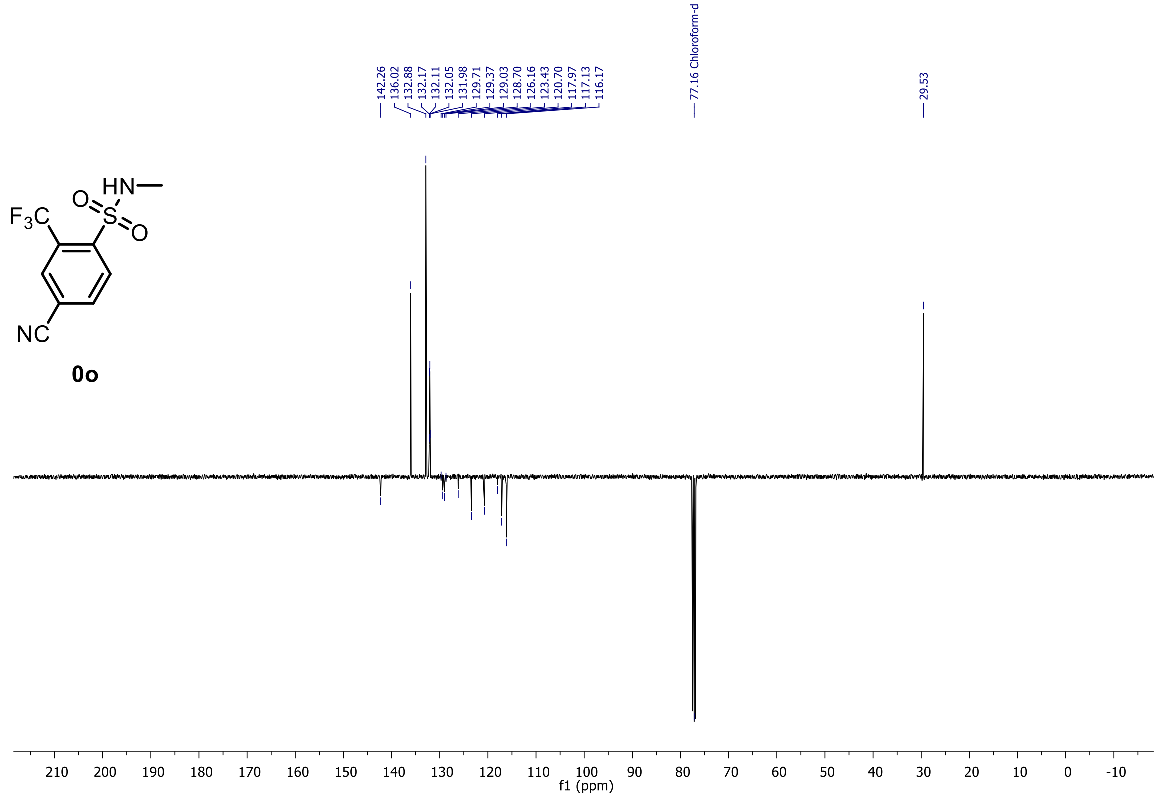

^{19}F NMR (377 MHz, $CDCl_3$)

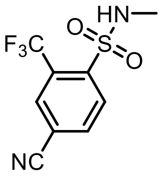

0o

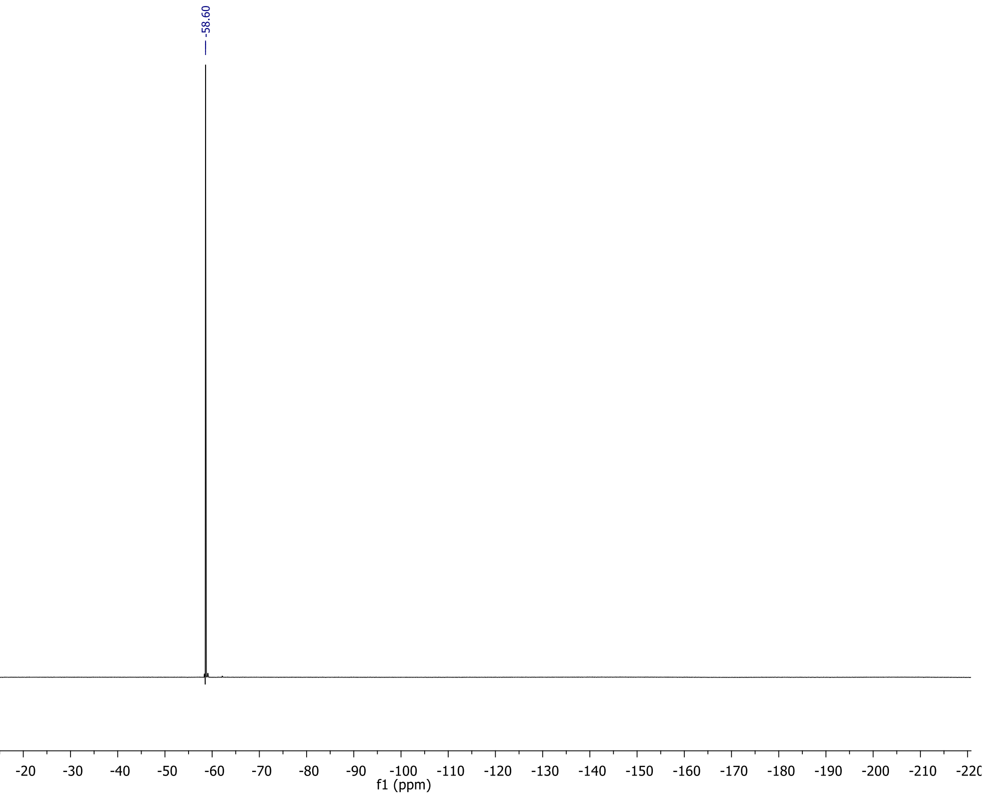

1a: *N*-Methyl-*N*-((4-nitrophenyl)sulfonyl)acrylamide

^{1}H NMR (400 MHz, $CDCl_3$)

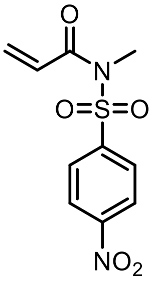

1a

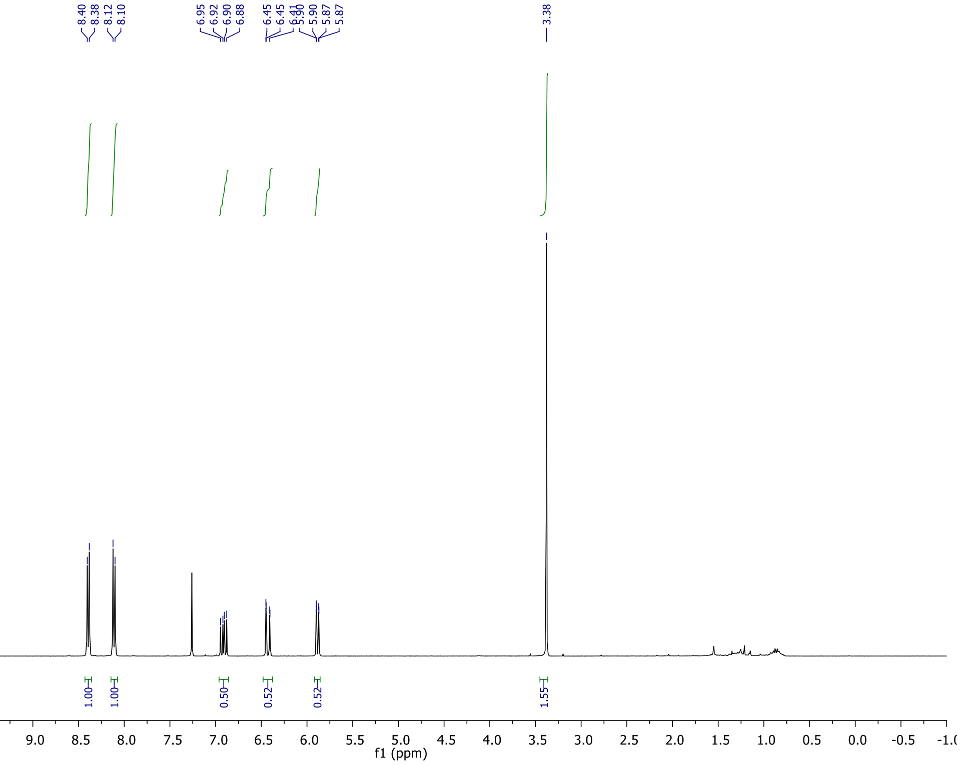

^{13}C NMR (100 MHz, $CDCl_3$)

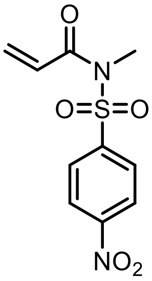

1a

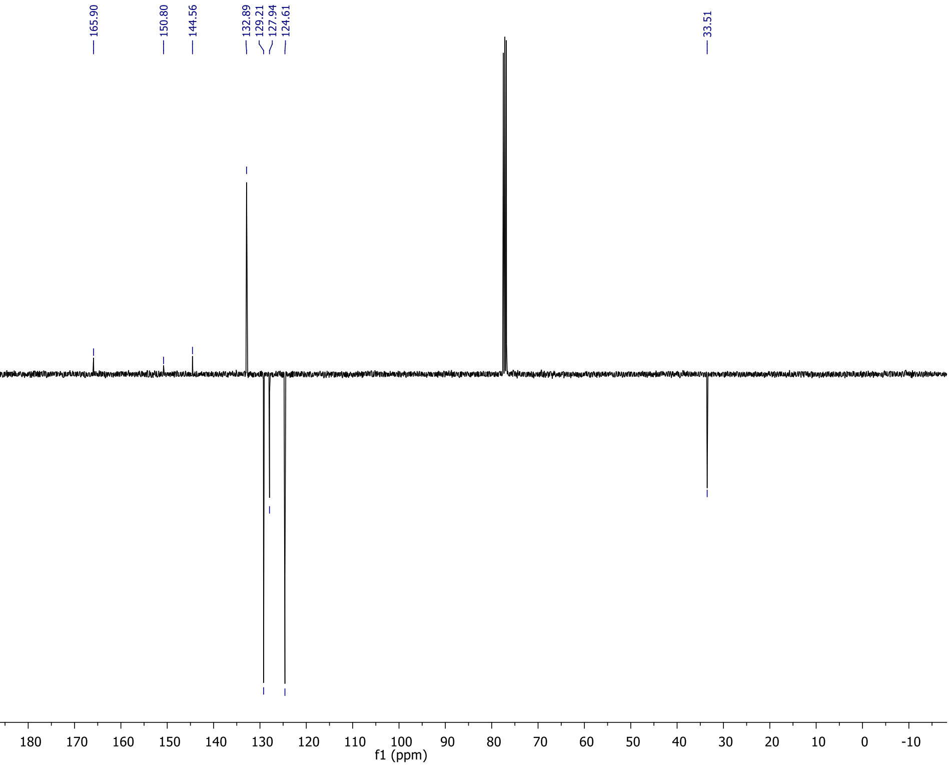

1b: *N*-Isopropyl-*N*-((4-nitrophenyl)sulfonyl)acrylamide

^{1}H NMR (400 MHz, $CDCl_3$)

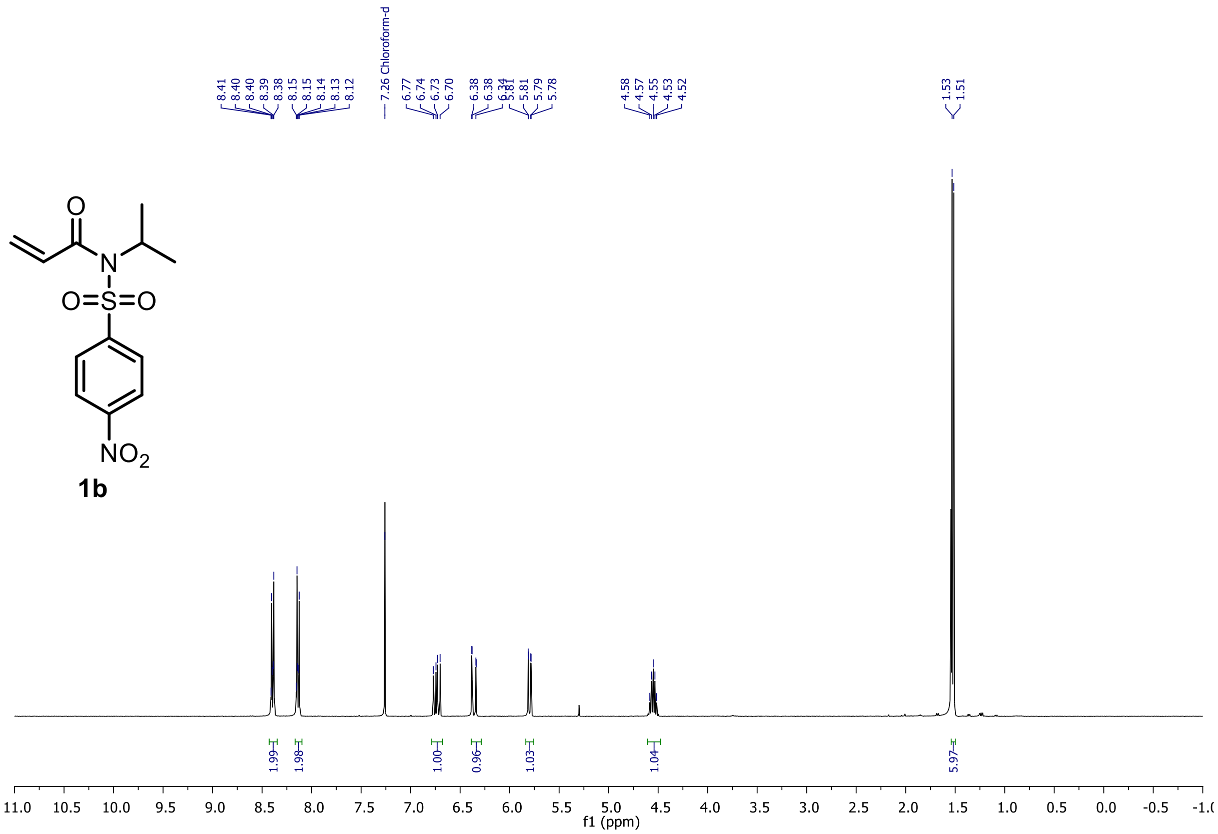

^{13}C NMR (100 MHz, $CDCl_3$)

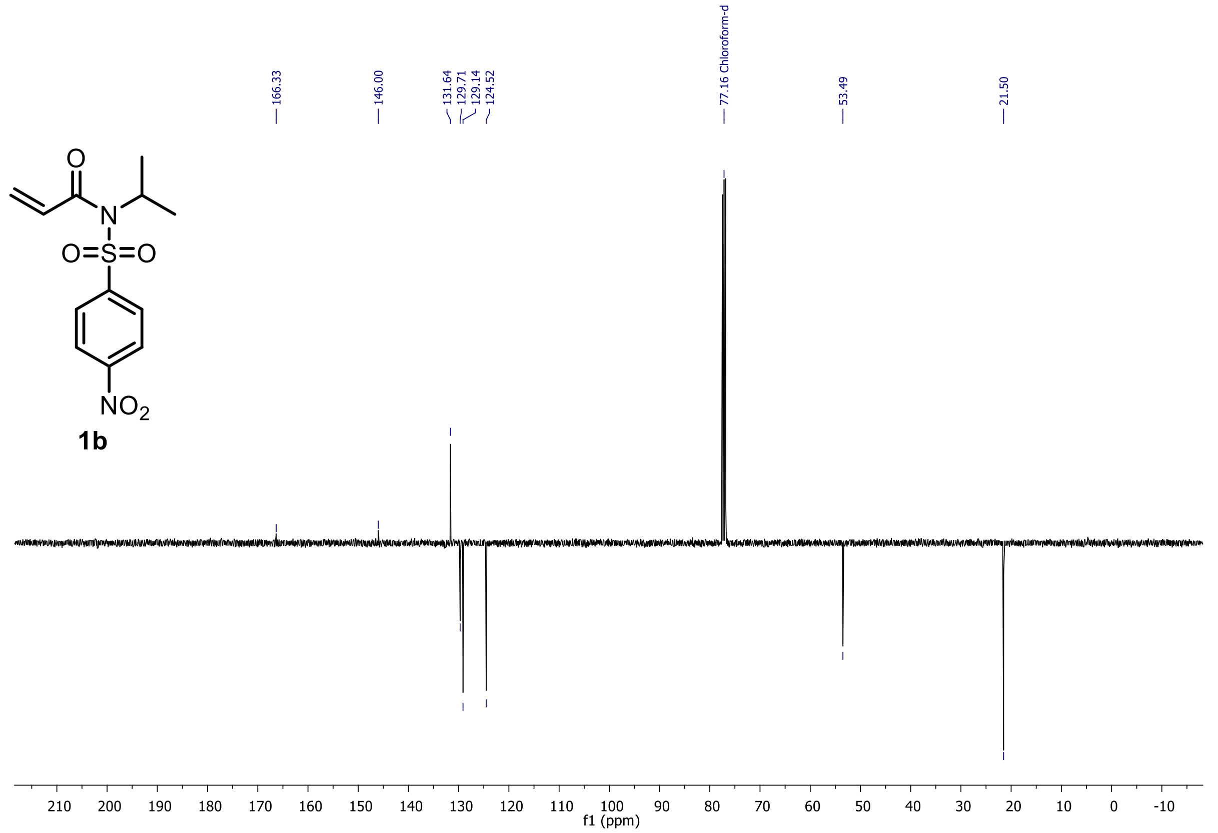

1c: *N*-(*tert*-Butyl)-*N*-((4-nitrophenyl)sulfonyl)acrylamide

^{1}H NMR (400 MHz, $CDCl_3$)

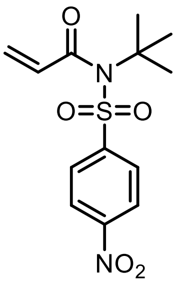

1c

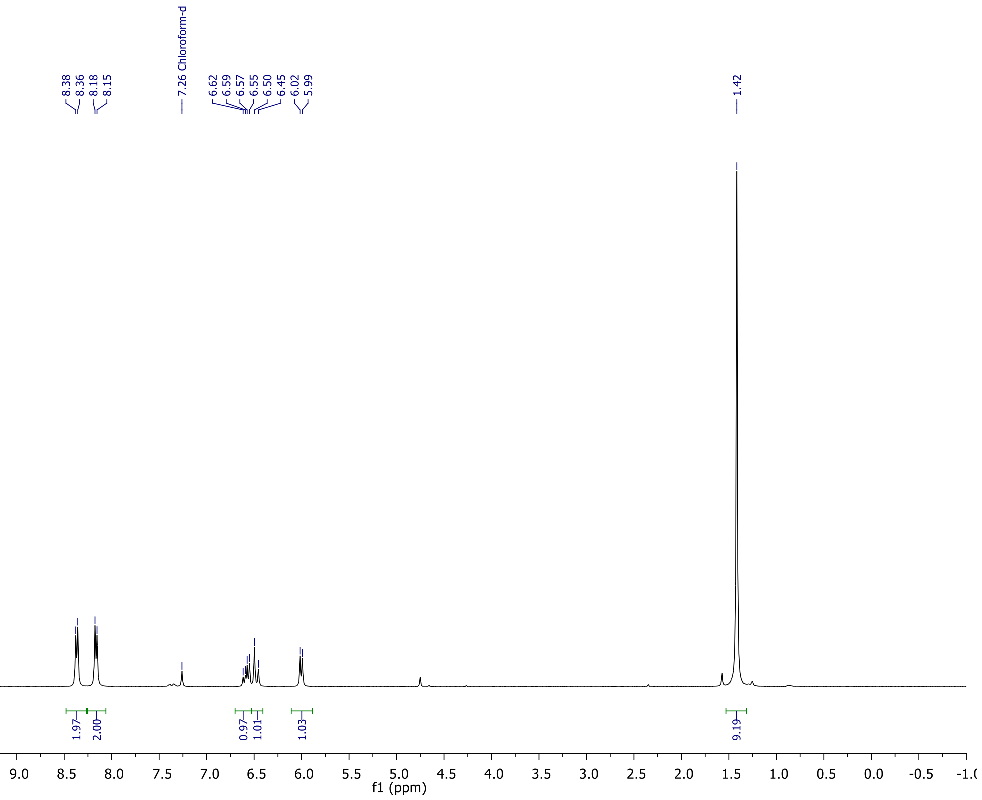

^{13}C NMR (100 MHz, $CDCl_3$)

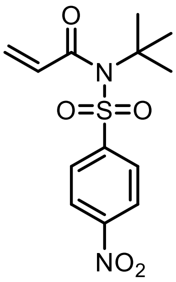

1c

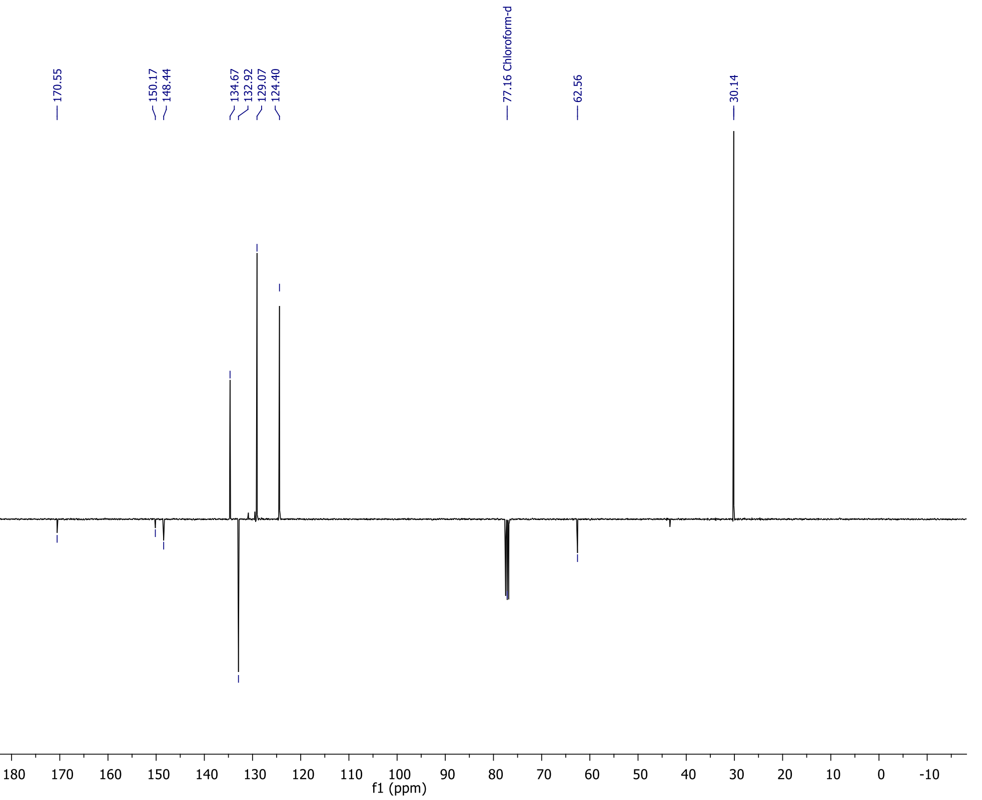

1d: *N*-Allyl-*N*-((4-nitrophenyl)sulfonyl)acrylamide

^{1}H NMR (400 MHz, CDCl$_3$)

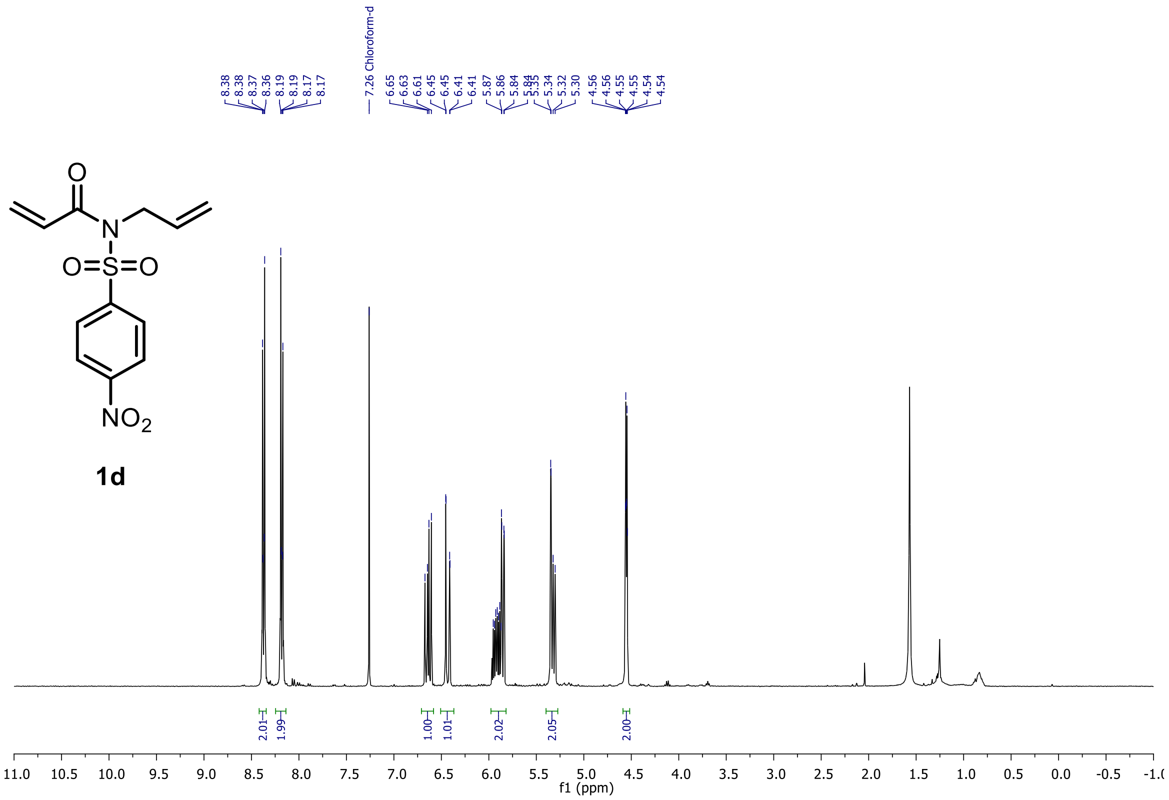

^{13}C NMR (100 MHz, CDCl$_3$)

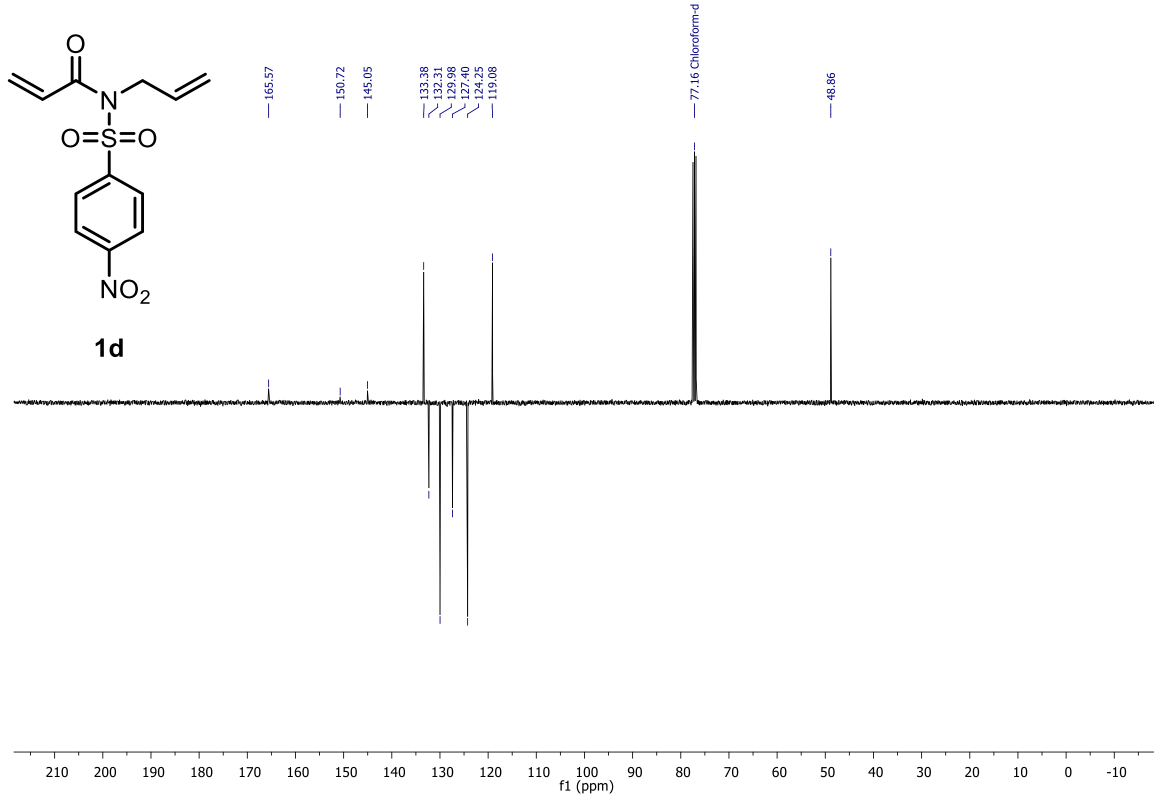

1e: *N*-Benzyl-*N*-((4-nitrophenyl)sulfonyl)acrylamide

^{1}H NMR (400 MHz, $CDCl_3$)

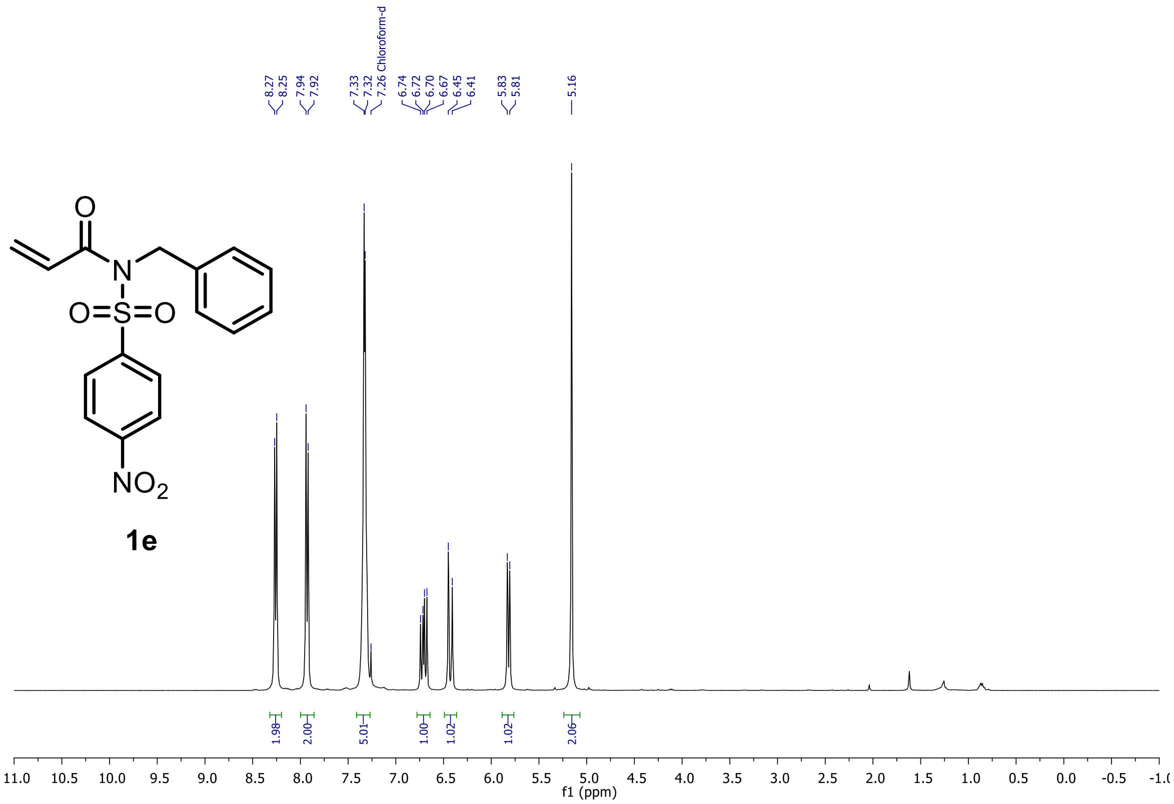

^{13}C NMR (100 MHz, $CDCl_3$)

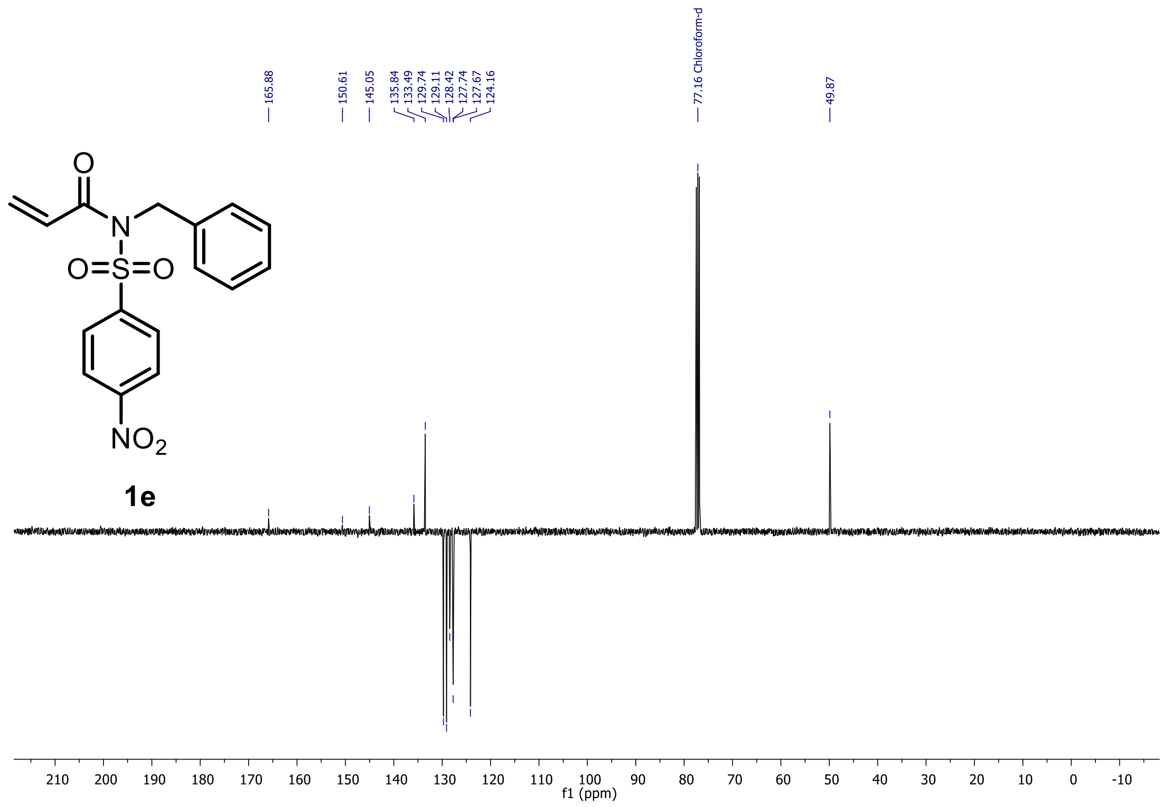

1f: *N*-(4,4-Dimethoxybutyl)-*N*-((4-nitrophenyl)sulfonyl)acrylamide

^{1}H NMR (400 MHz, $CDCl_3$)

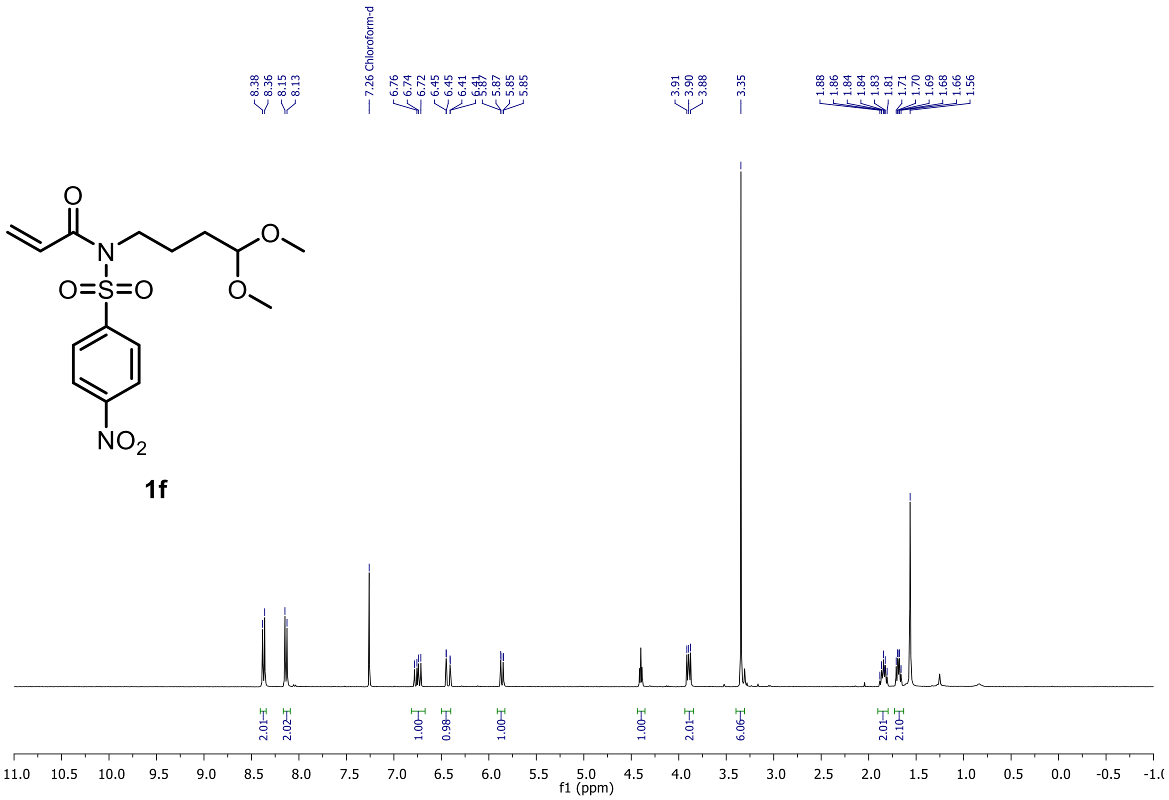

^{13}C NMR (100 MHz, $CDCl_3$)

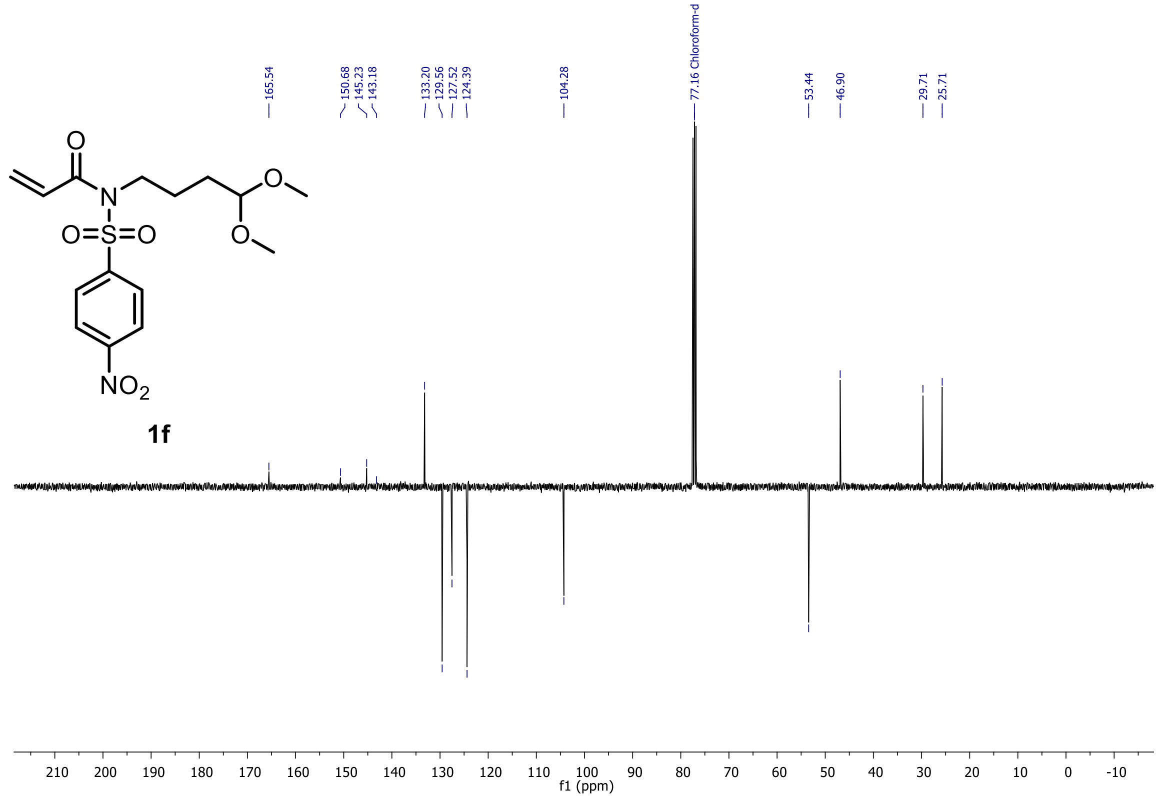

1g: Methyl-*N*-acryloyl-*N*-((4-nitrophenyl)sulfonyl)glycinate

^{1}H NMR (400 MHz, $CDCl_3$)

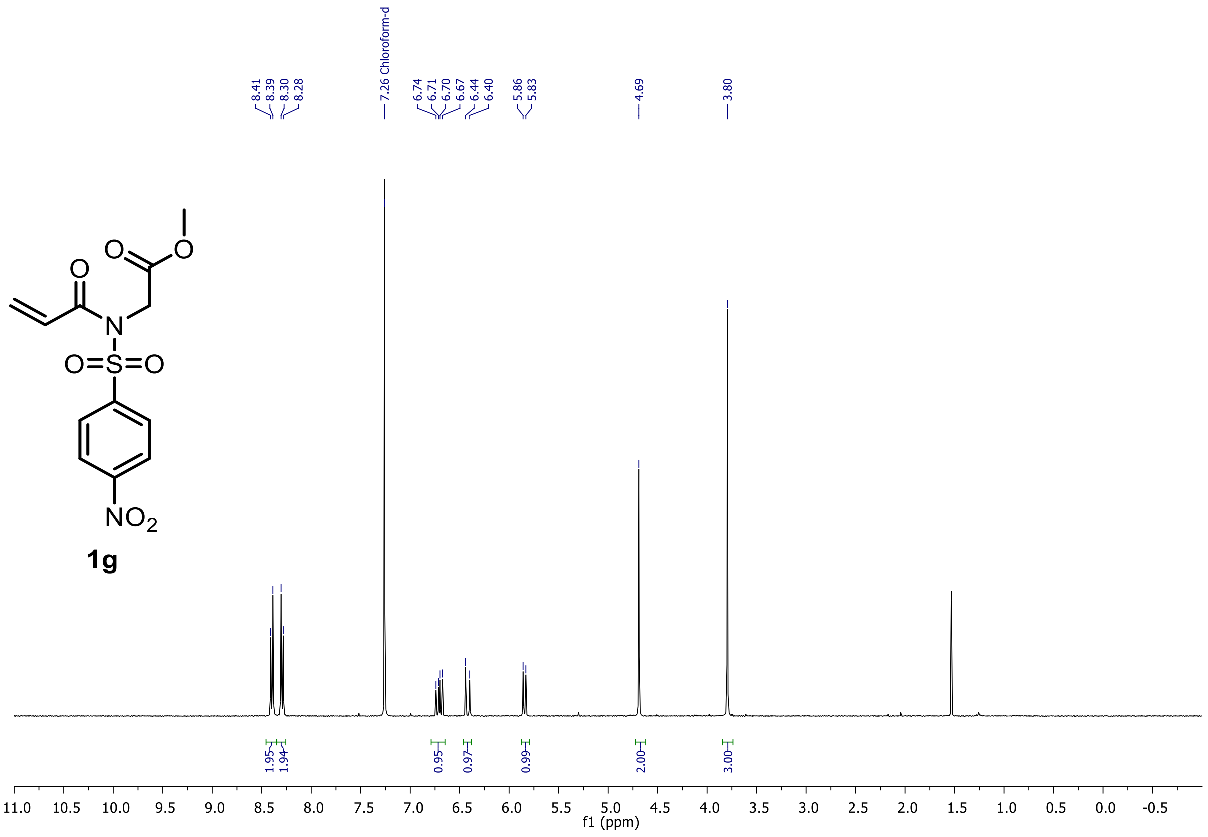

^{13}C NMR (100 MHz, $CDCl_3$)

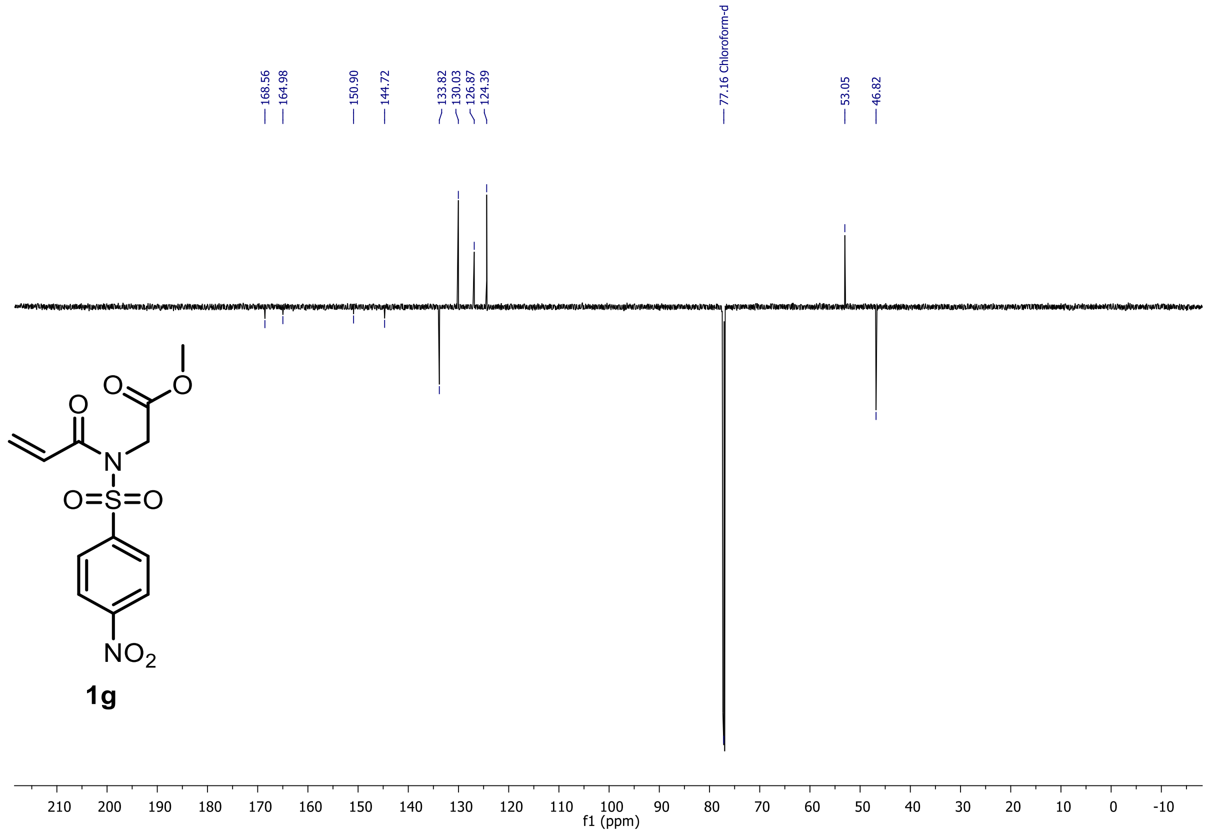

1h: Methyl-*N*-acryloyl-*N*-((4-nitrophenyl)sulfonyl)methioninate

^{1}H NMR (400 MHz, $CDCl_3$)

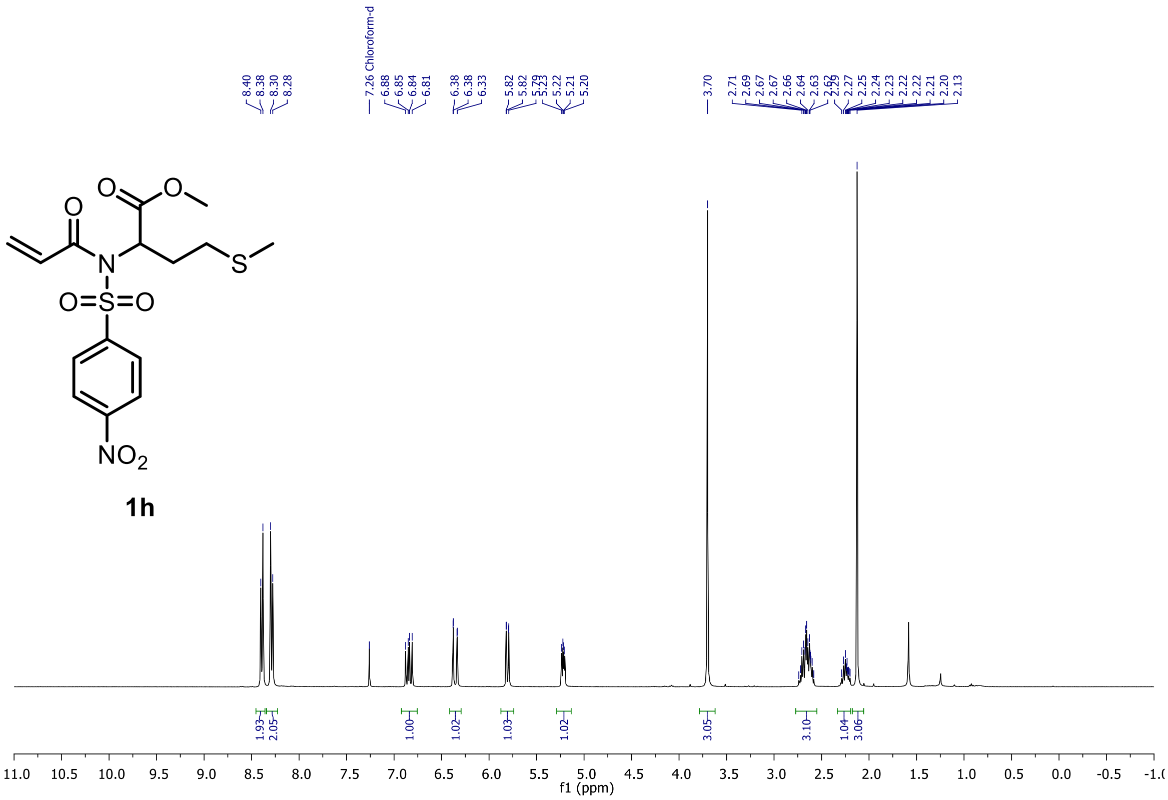

^{13}C NMR (100 MHz, $CDCl_3$)

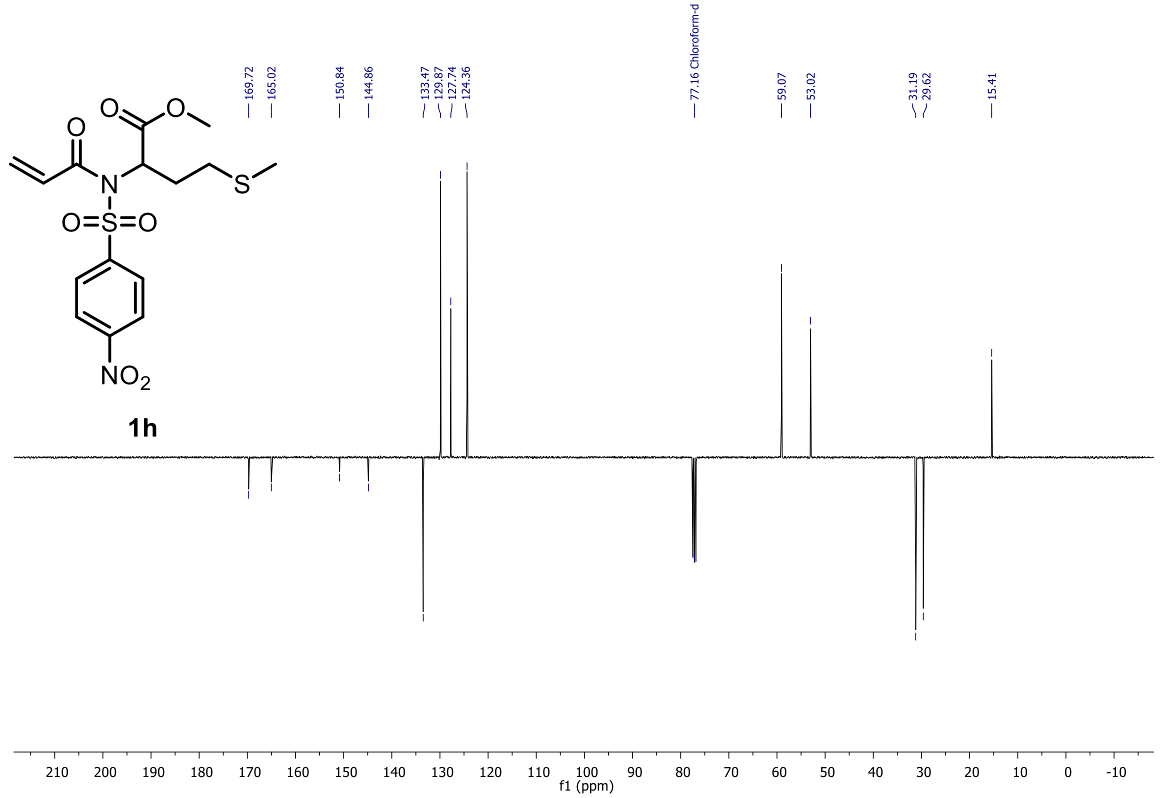

1i: *N*-(3,4-Dimethoxyphenethyl)-*N*-((4-nitrophenyl)sulfonyl)acrylamide

^{1}H NMR (400 MHz, CDCl$_3$)

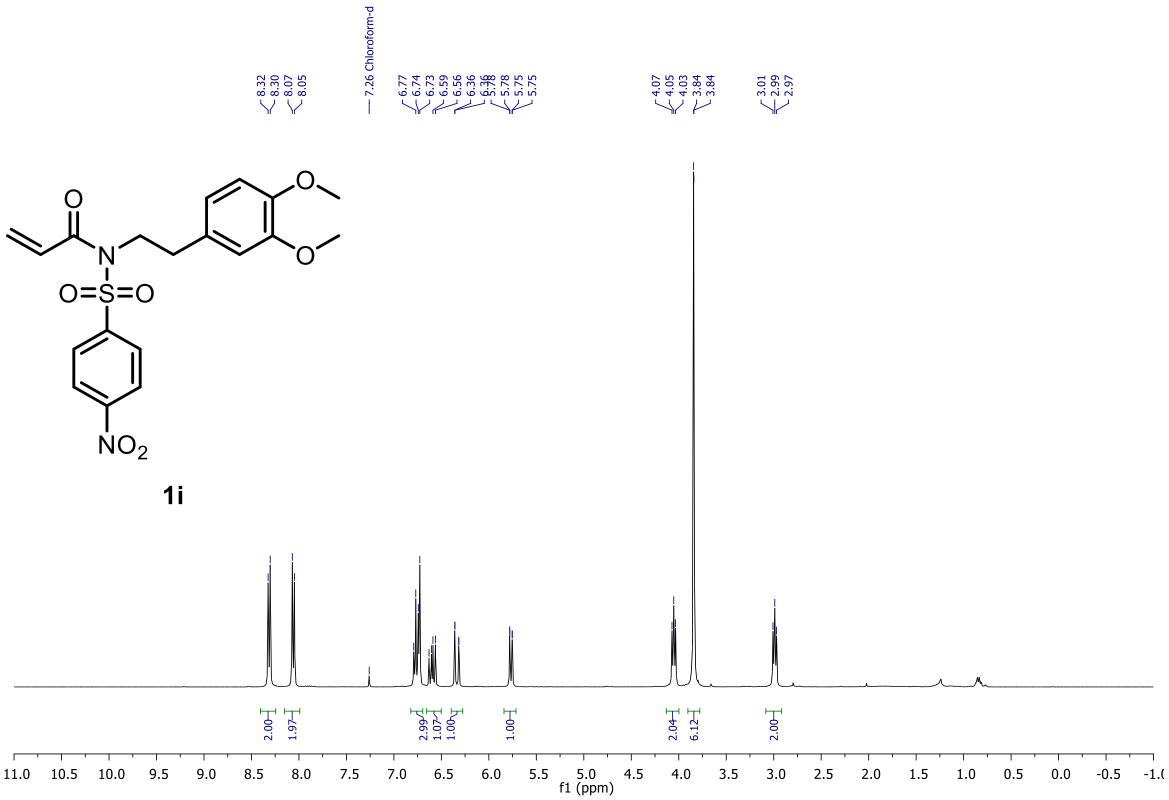

^{13}C NMR (100 MHz, CDCl$_3$)

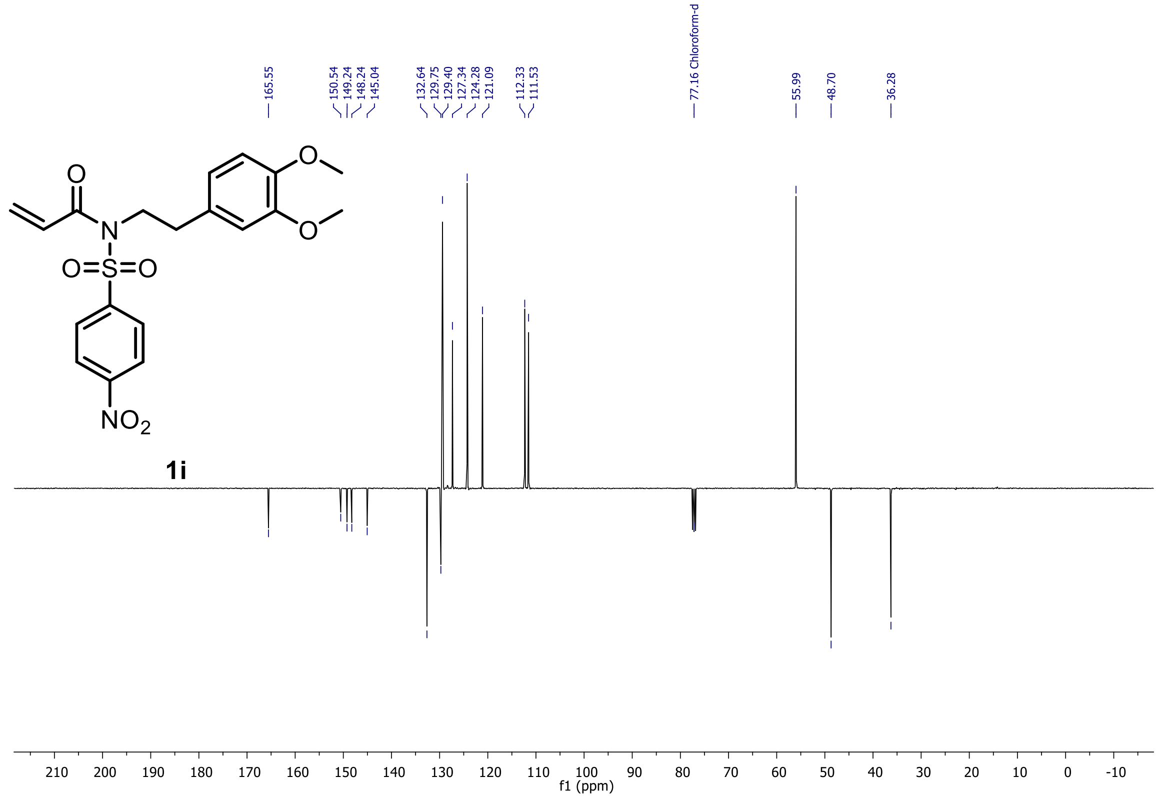

1j: *tert*-Butyl 3-(2-(*N*-((4-nitrophenyl)sulfonyl)acrylamido)ethyl)-1*H*-indole-1-carboxylate

^{1}H NMR (400 MHz, CDCl$_3$)

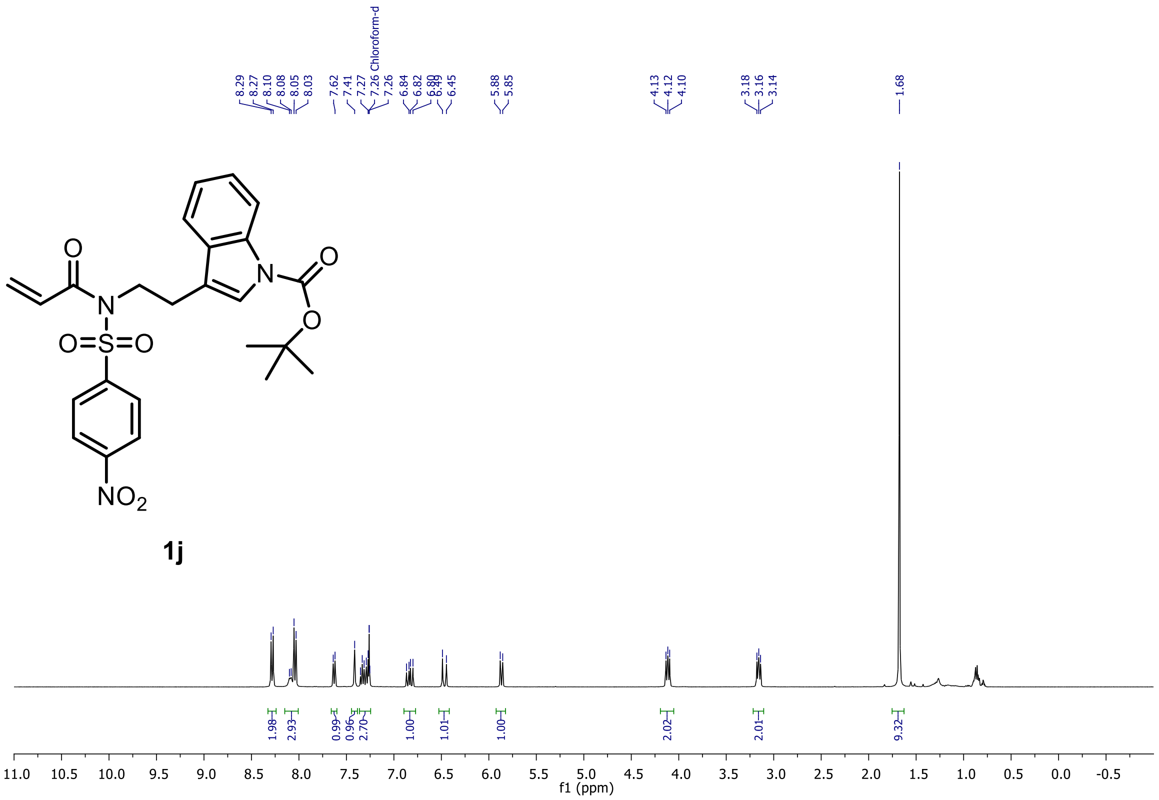

^{13}C NMR (100 MHz, CDCl$_3$)

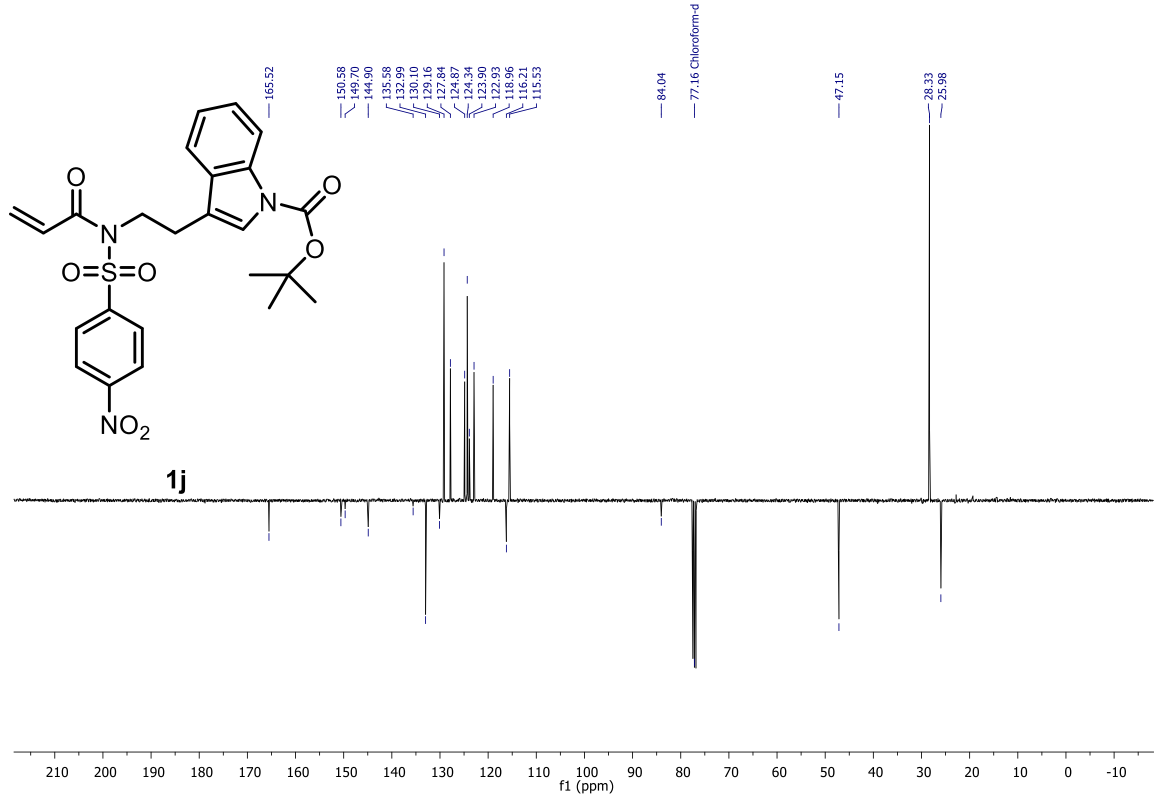

1k: Ethyl-(*E*)-6-(*N*-((4-nitrophenyl)sulfonyl)acrylamido)hex-2-enoate

^{1}H NMR (400 MHz, CDCl$_3$)

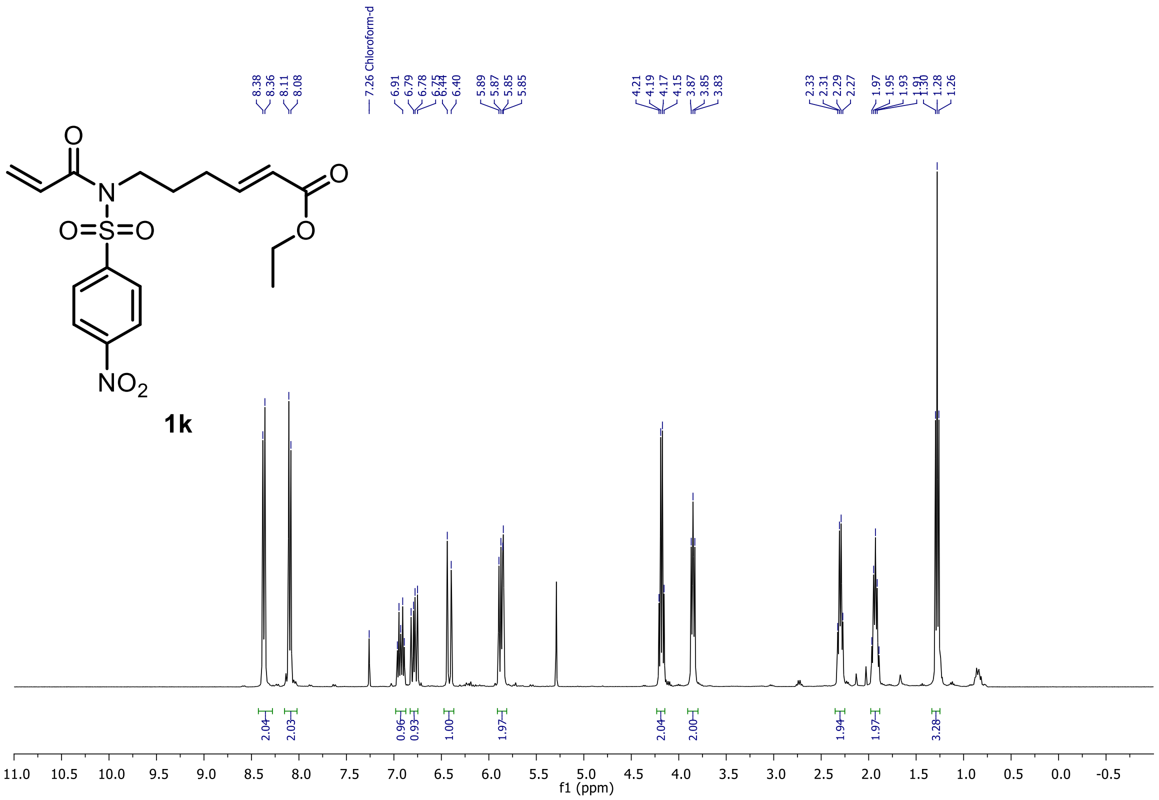

^{13}C NMR (100 MHz, CDCl$_3$)

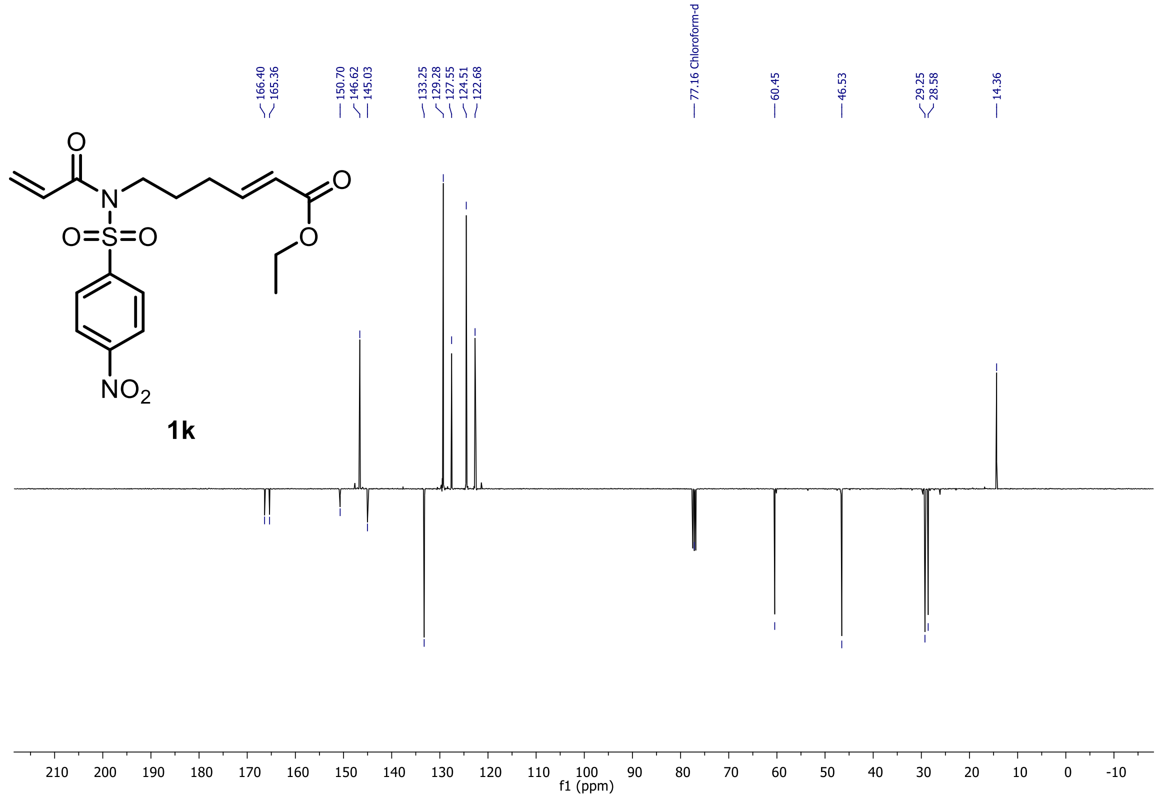

1l: *N*-Methyl-*N*-((2-nitrophenyl)sulfonyl)acrylamide

^{1}H NMR (400 MHz, $CDCl_3$)

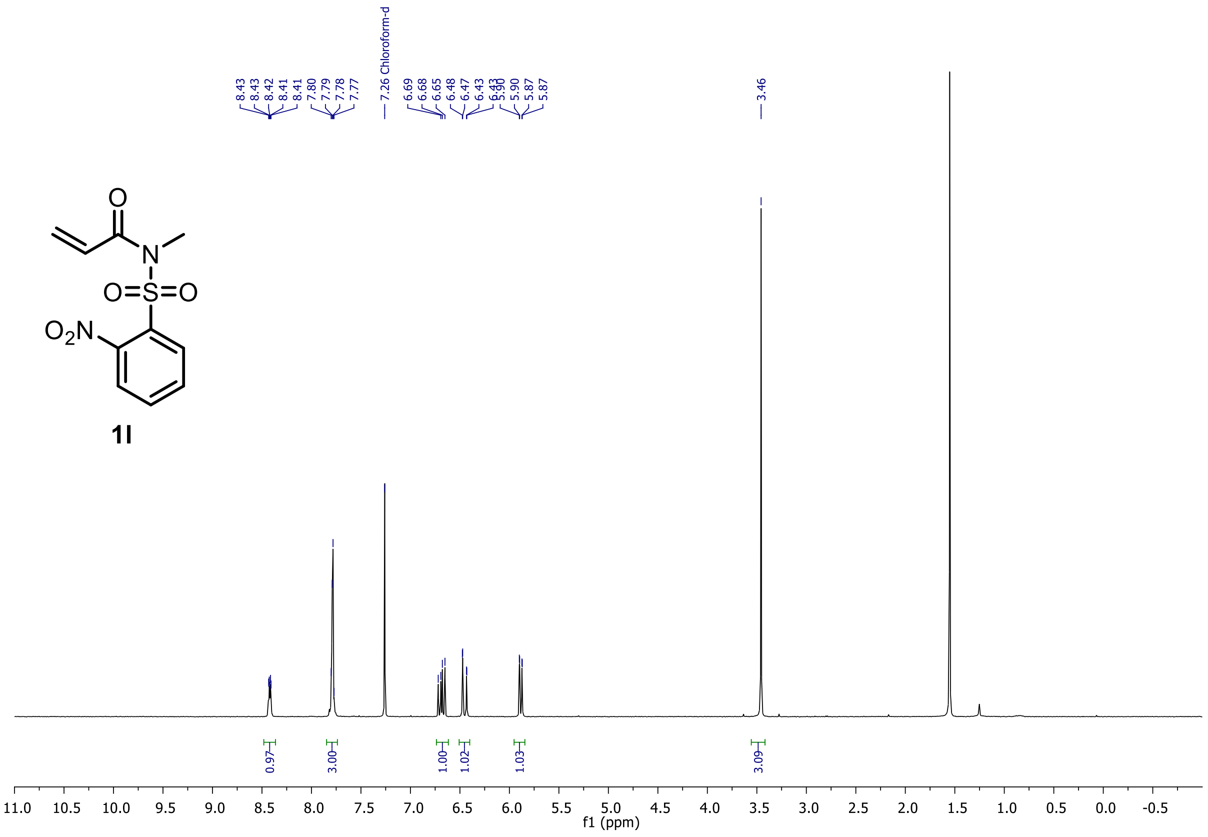

^{13}C NMR (100 MHz, $CDCl_3$)

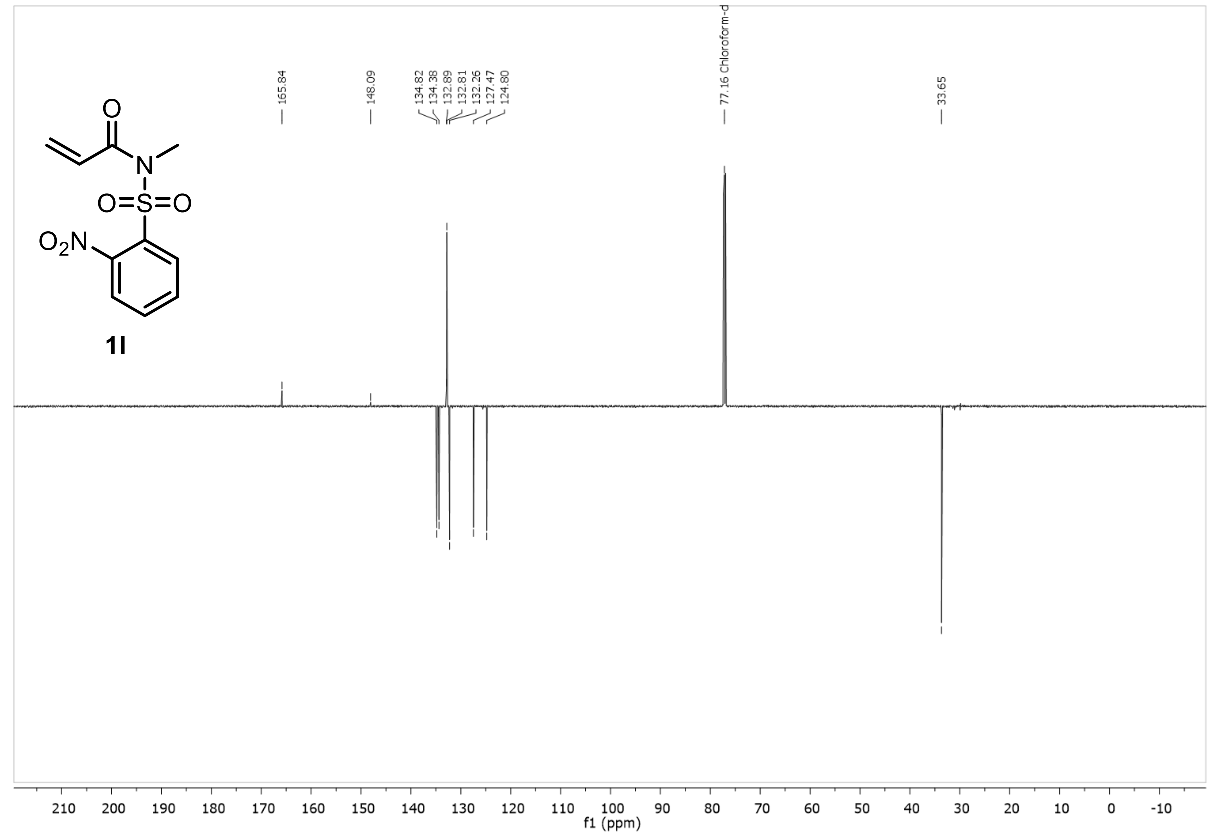

1m: *N*-((2-Methoxy-4-nitrophenyl)sulfonyl)-*N*-methylacrylamide

^{1}H NMR (400 MHz, $CDCl_3$)

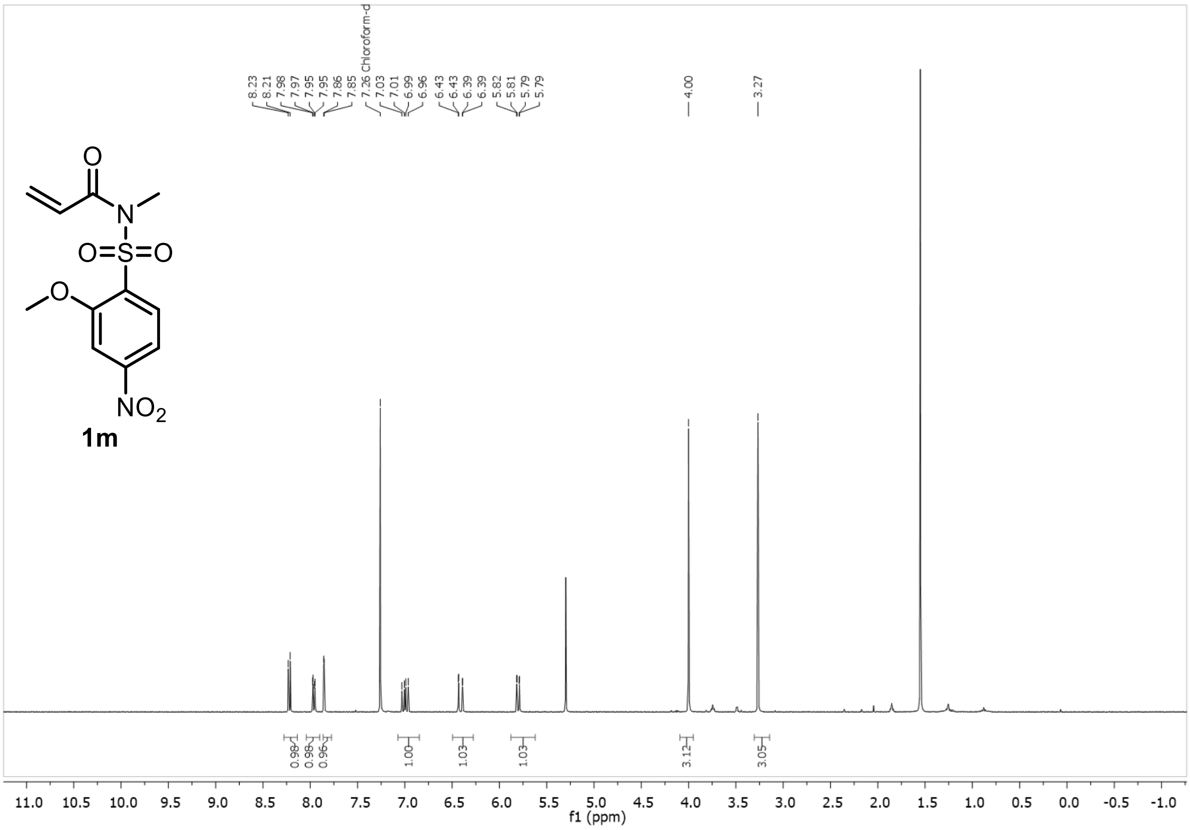

^{13}C NMR (100 MHz, $CDCl_3$)

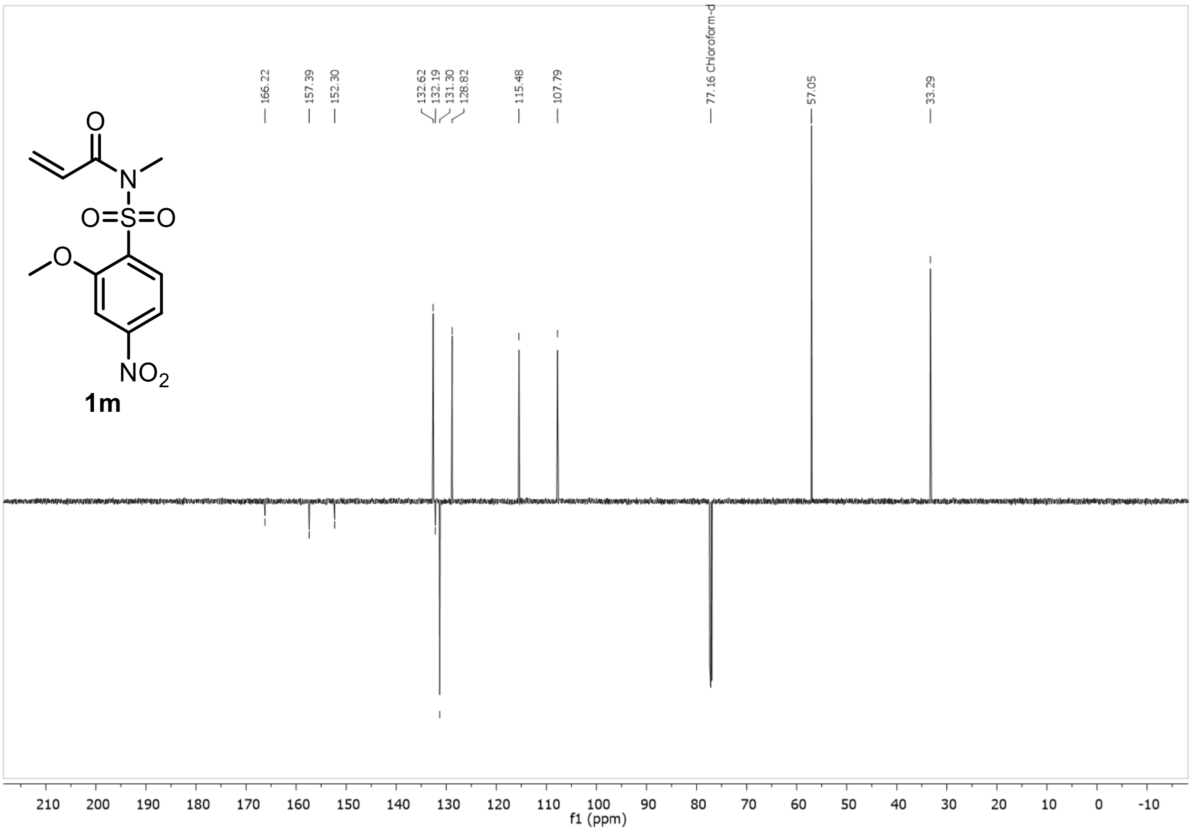

1n: *N*-((2,5-Bis(trifluoromethyl)phenyl)sulfonyl)-N-methylacrylamide

^{1}H NMR (400 MHz, $CDCl_3$)

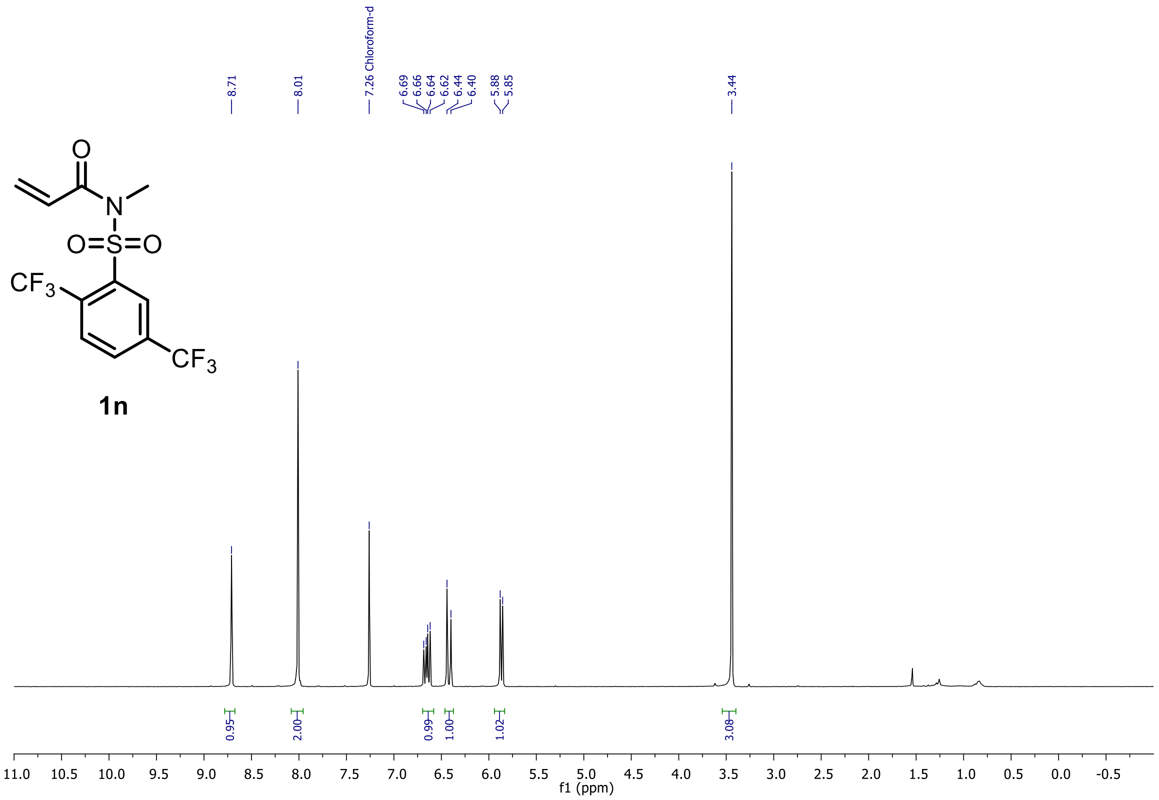

^{13}C NMR (176 MHz, $CDCl_3$)

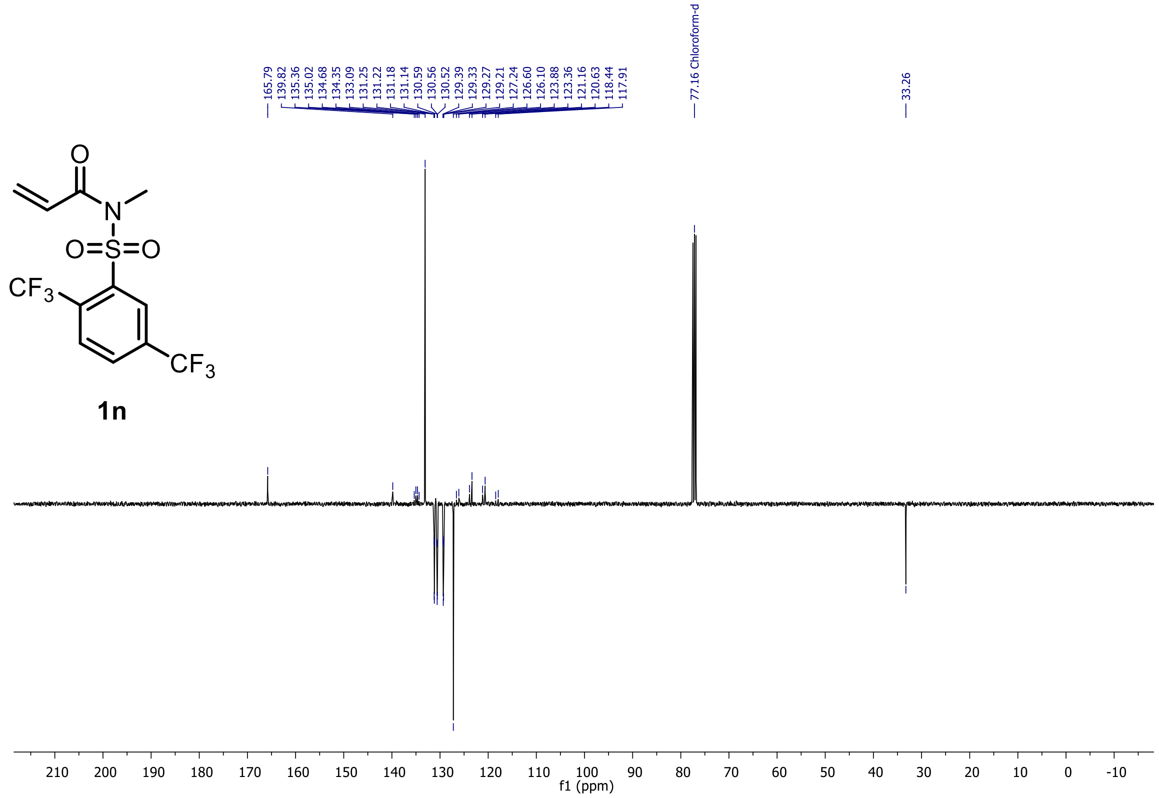

^{19}F NMR (377 MHz, CDCl$_3$)

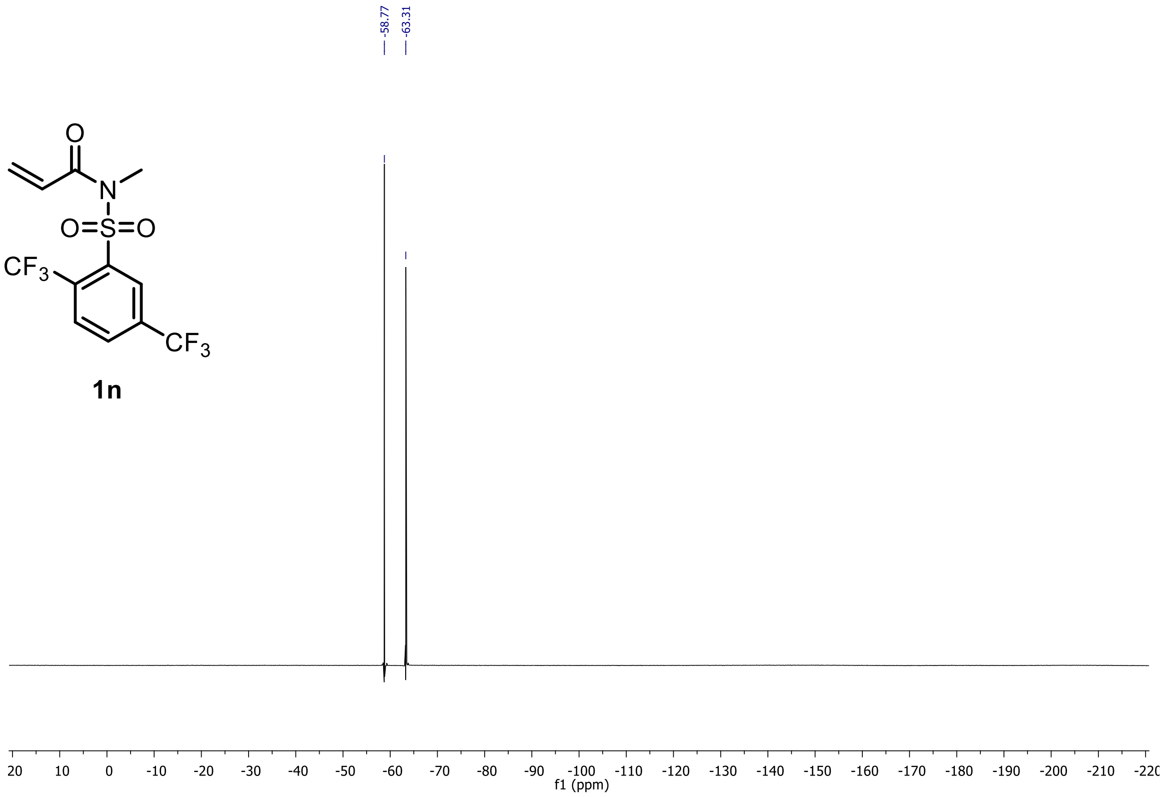

1n

1o: *N*-((4-Cyano-2-(trifluoromethyl)phenyl)sulfonyl)-*N*-methylacrylamide

^{1}H NMR (400 MHz, $CDCl_3$)

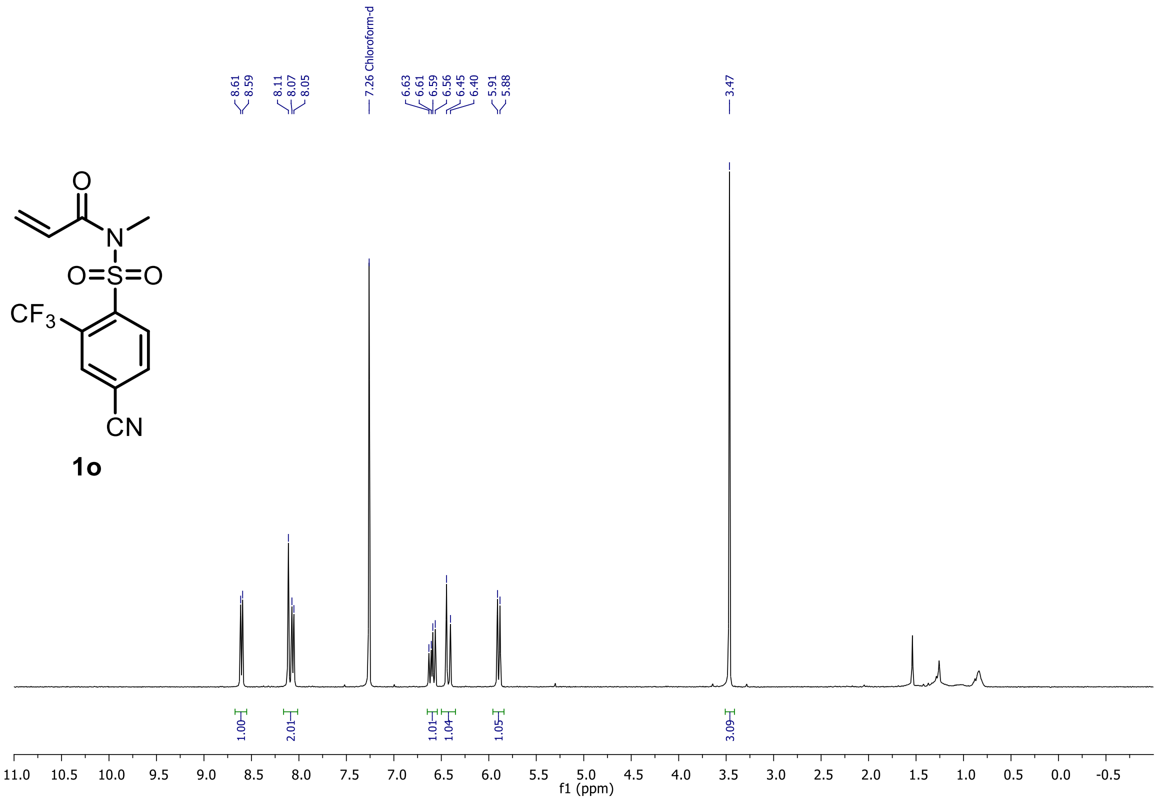

^{13}C NMR (176 MHz, $CDCl_3$)

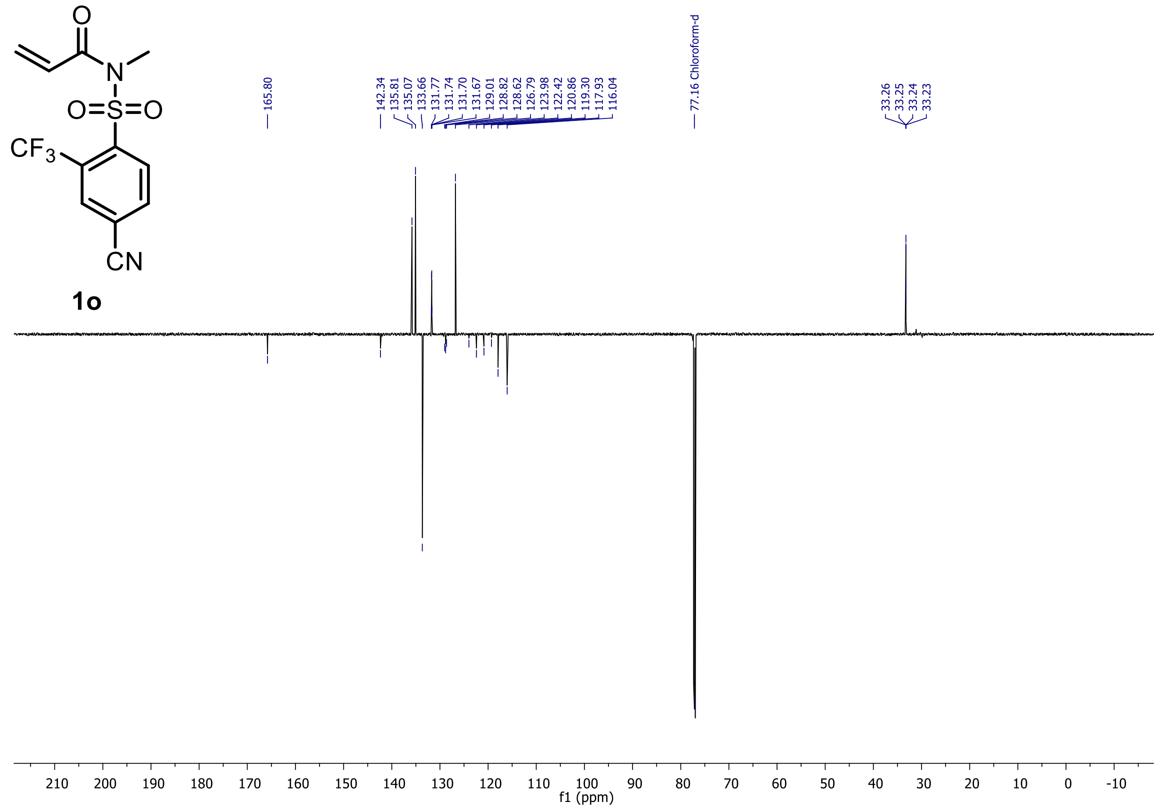

^{19}F NMR (659 MHz, $CDCl_3$)

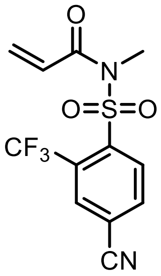

1o

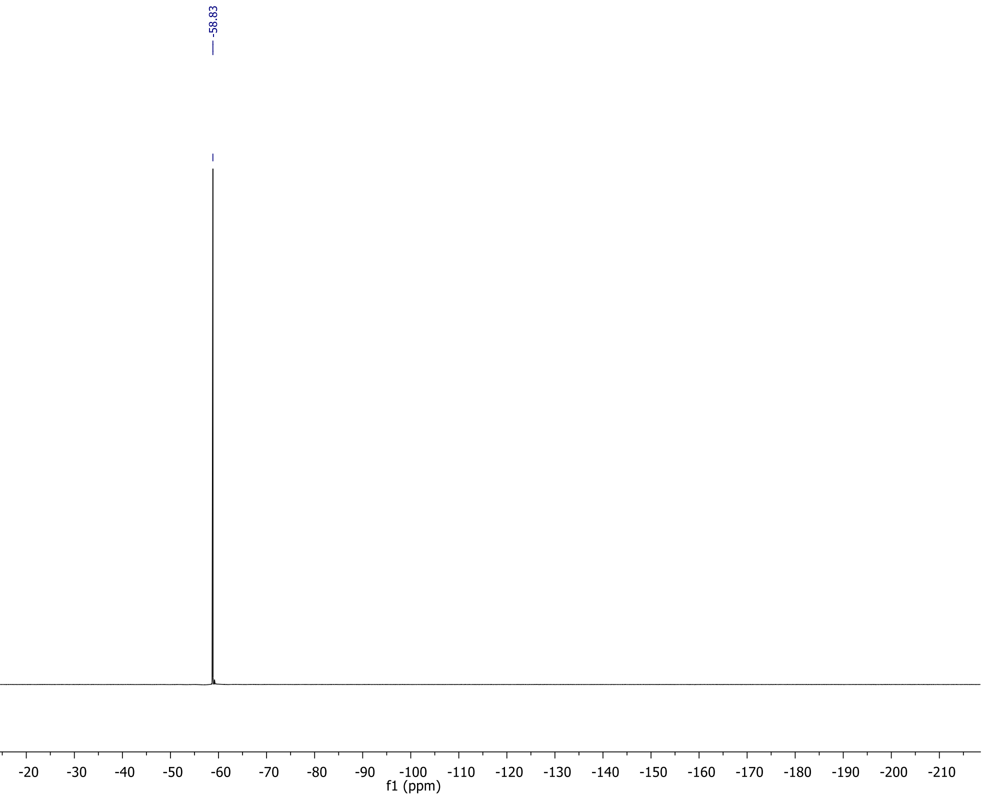

SI1: *N*-methyl-*N*-(pyridin-2-ylsulfonyl)acrylamide

^{1}H NMR (600 MHz, CDCl$_3$)

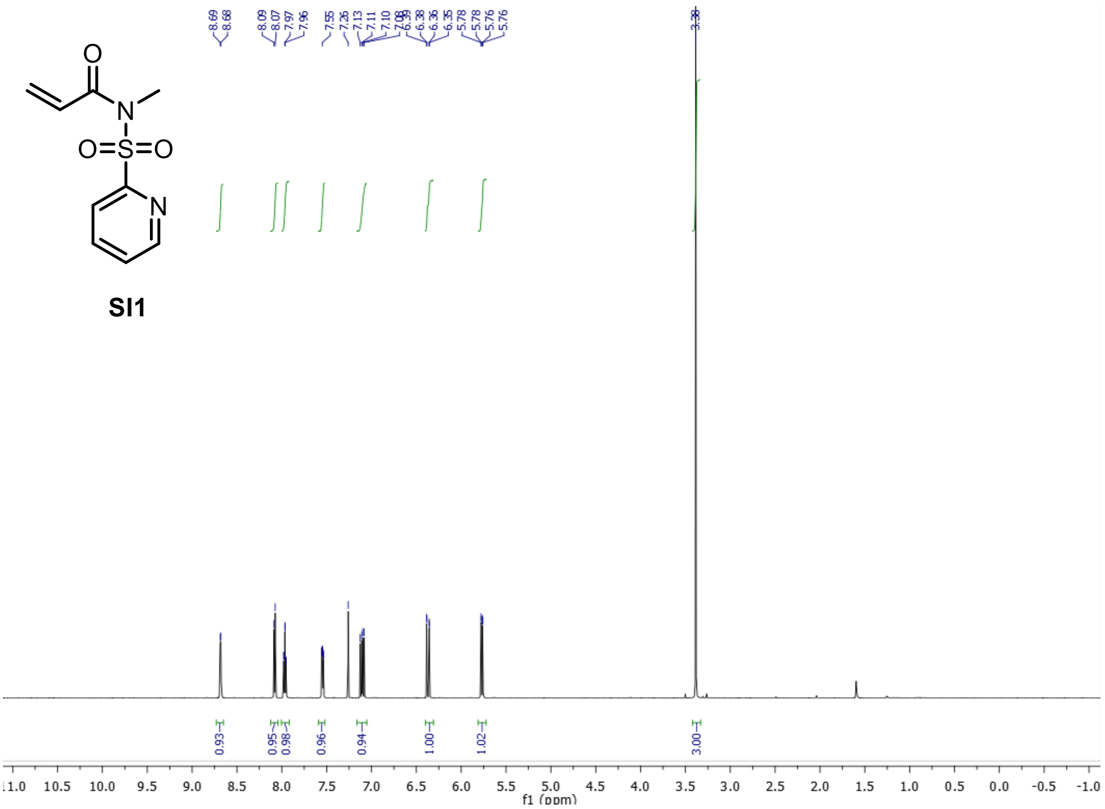

^{13}C NMR (151 MHz, CDCl$_3$)

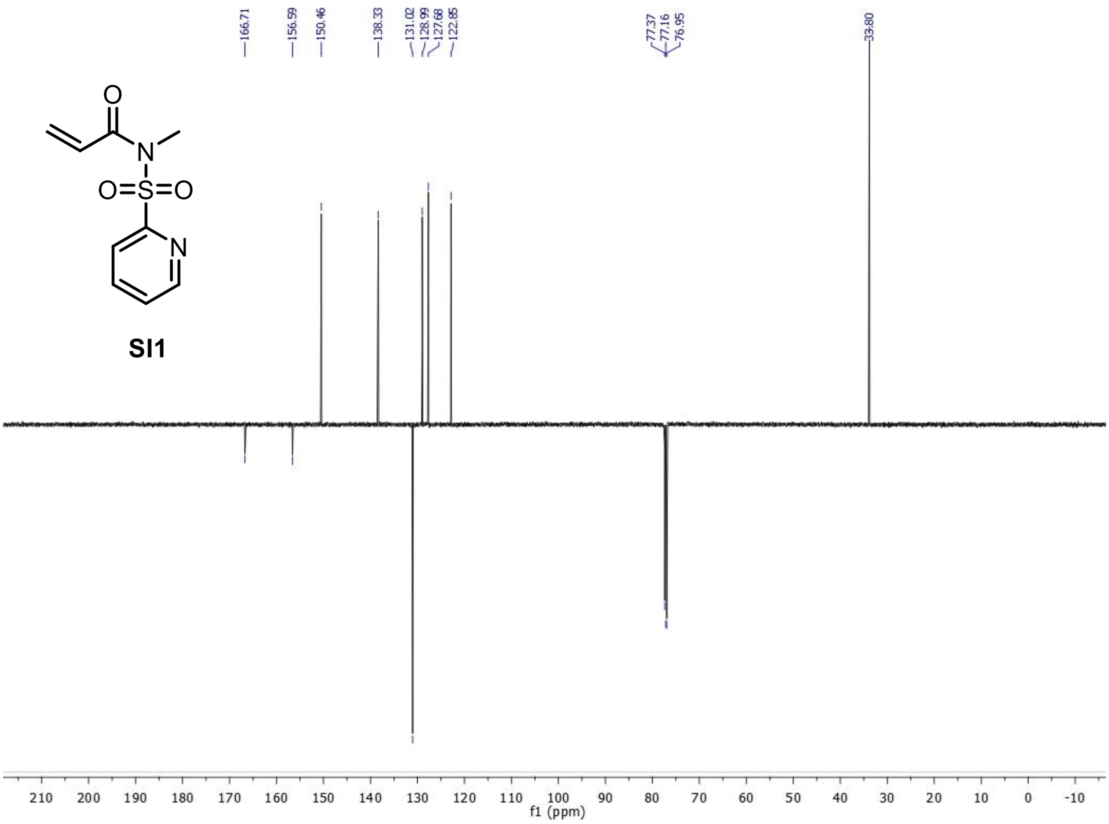

1p: 2-(*N*-acryloyl-*N*-methylsulfamoyl)pyridine 1-oxide

^{1}H NMR (600 MHz, $CDCl_3$)

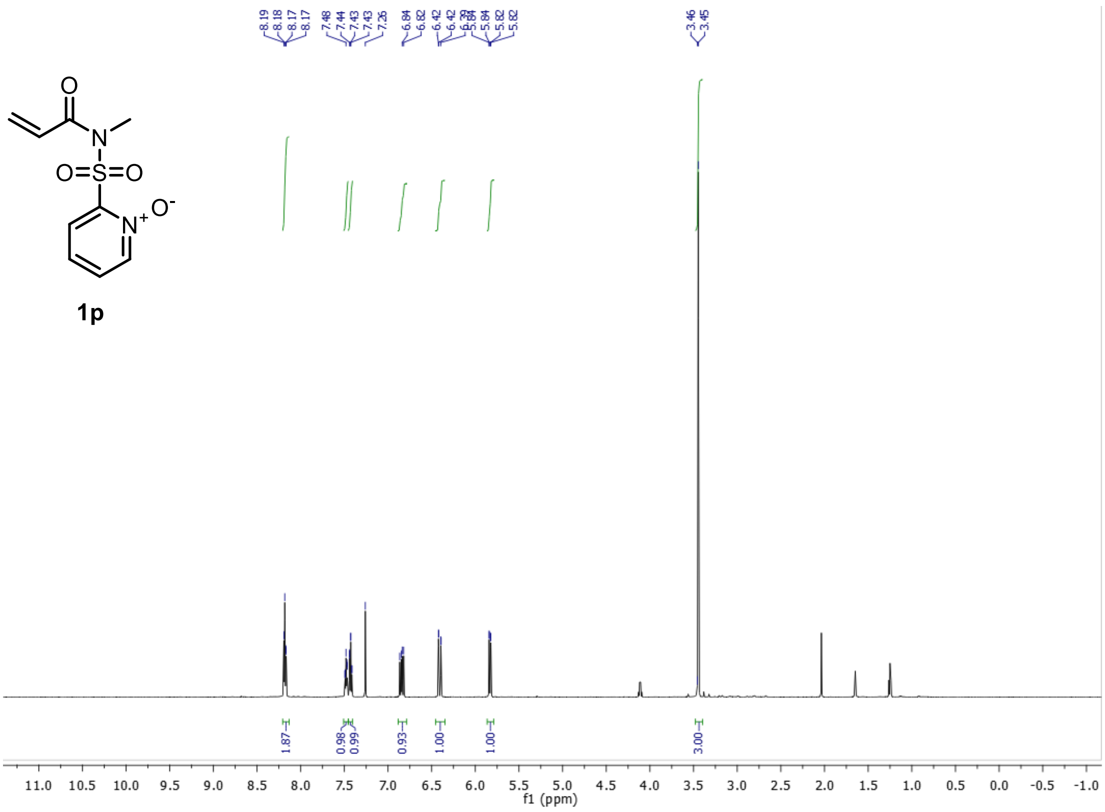

^{13}C NMR (151 MHz, $CDCl_3$)

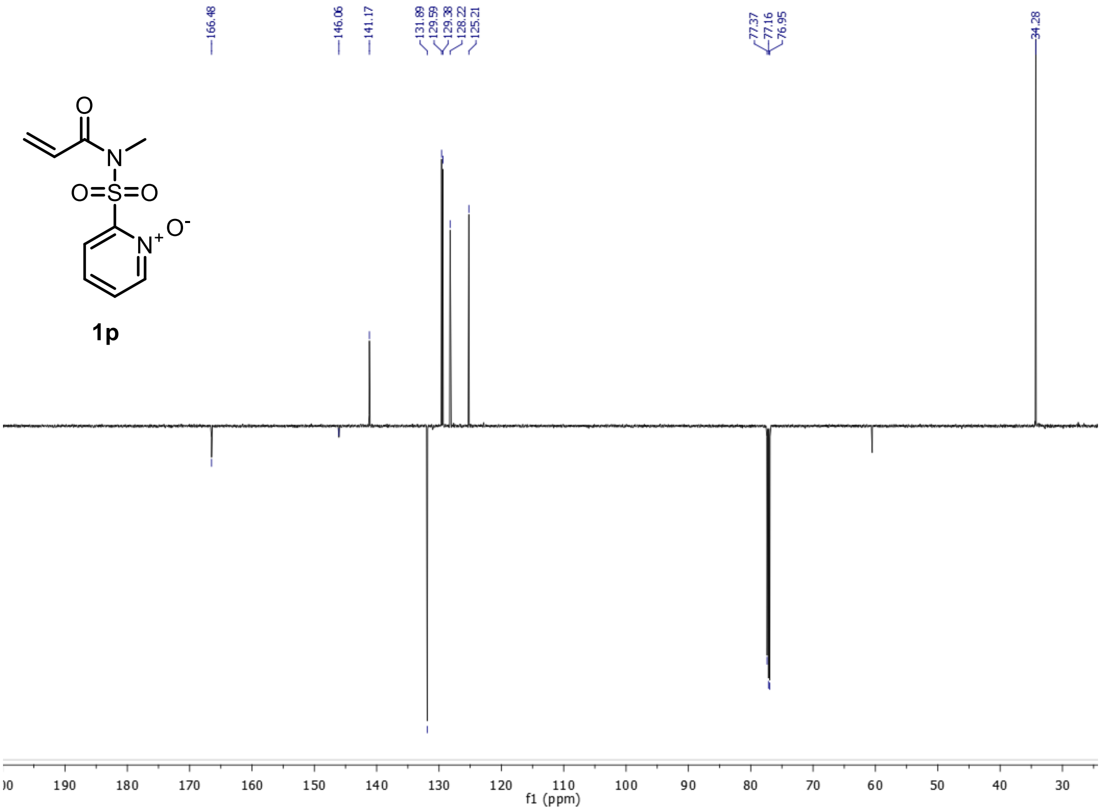

1q: (*E*)-*N*-Methyl-*N*-((4-nitrophenyl)sulfonyl)but-2-enamide

^{1}H NMR (400 MHz, $CDCl_3$)

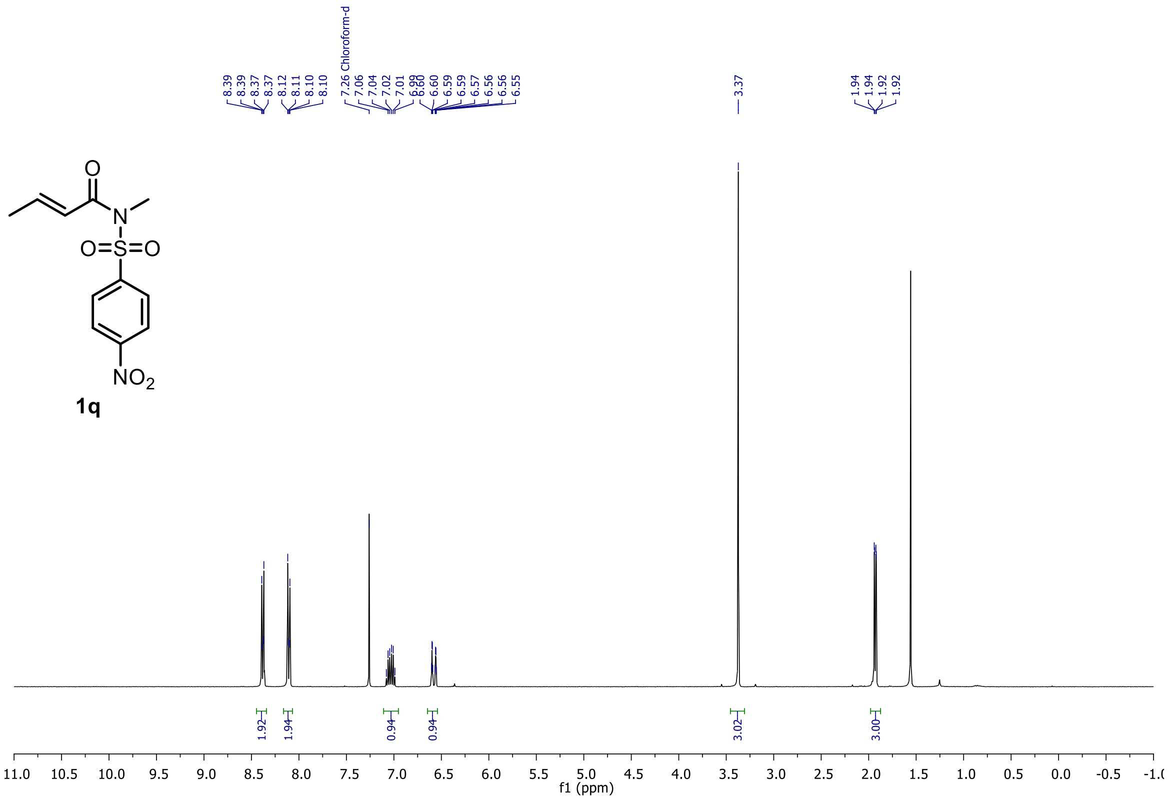

^{13}C NMR (100 MHz, $CDCl_3$)

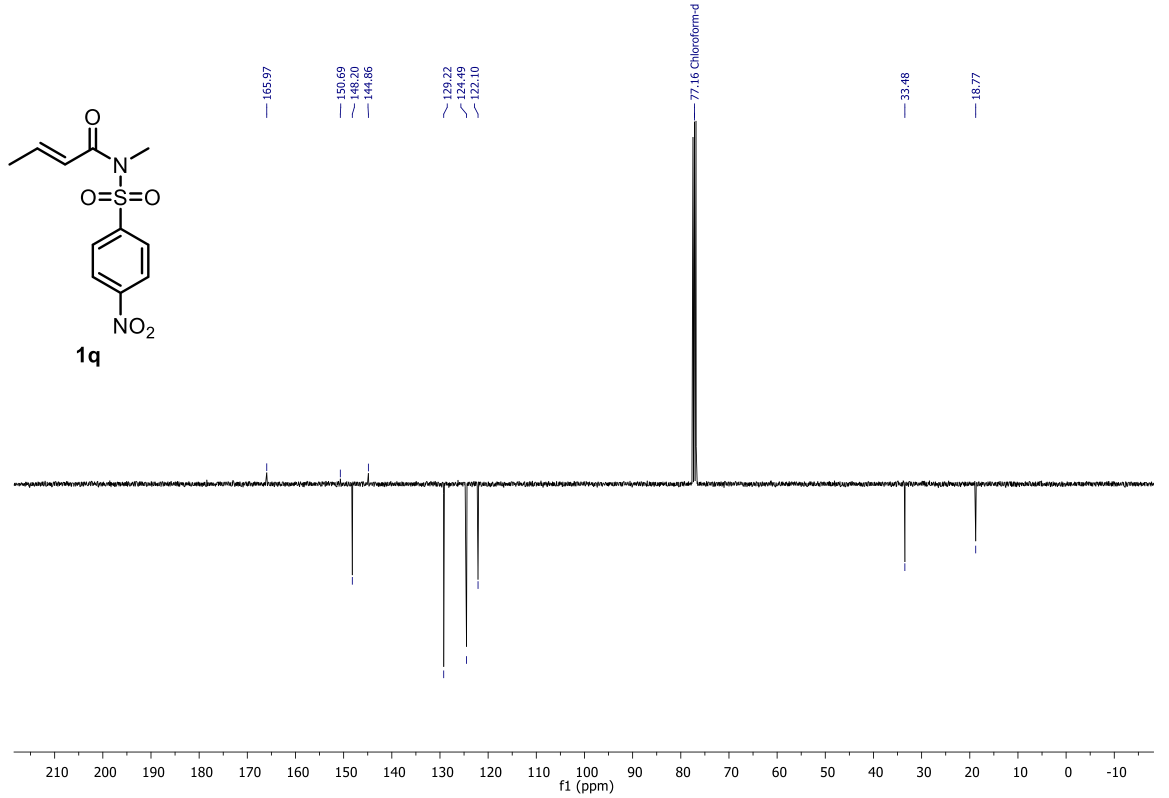

1r: (*E*)-*N*-Methyl-*N*-((4-nitrophenyl)sulfonyl)oct-2-enamide

^{1}H NMR (400 MHz, CDCl$_3$)

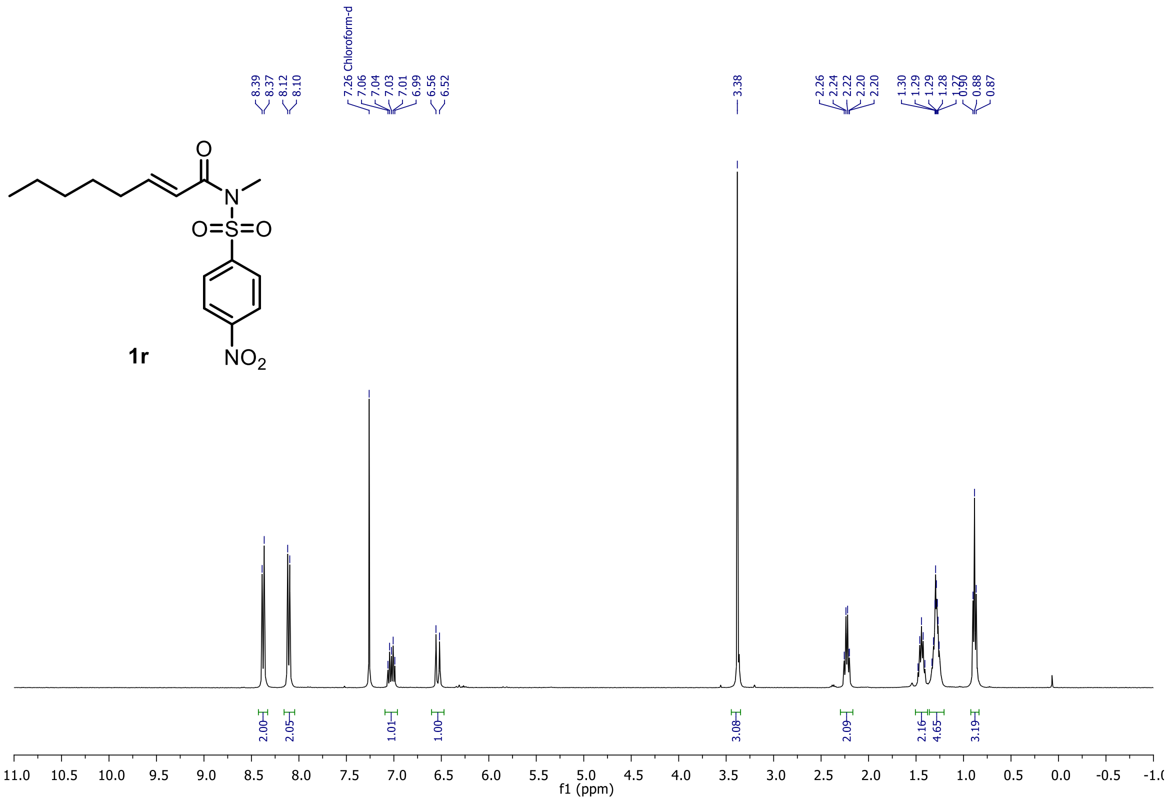

^{13}C NMR (100 MHz, CDCl$_3$)

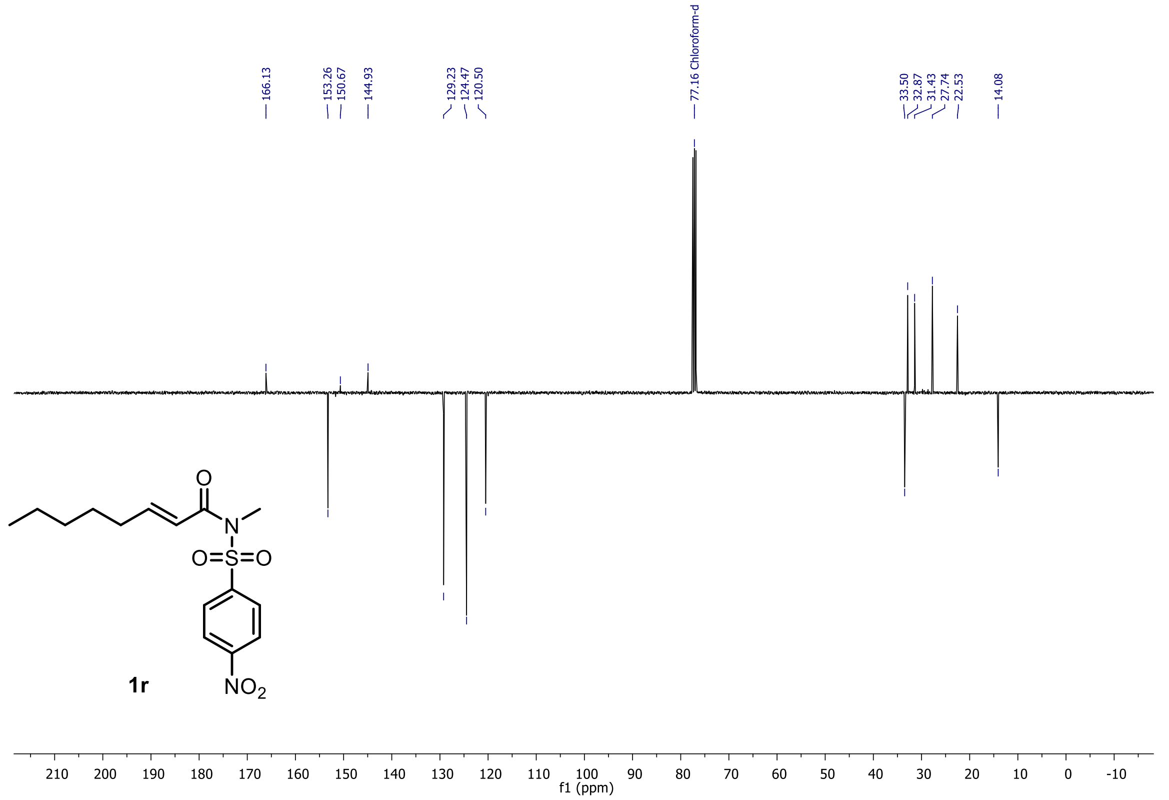

1s: *N*-3-Dimethyl-*N*-((4-nitrophenyl)sulfonyl)but-2-enamide

^{1}H NMR (400 MHz, $CDCl_3$)

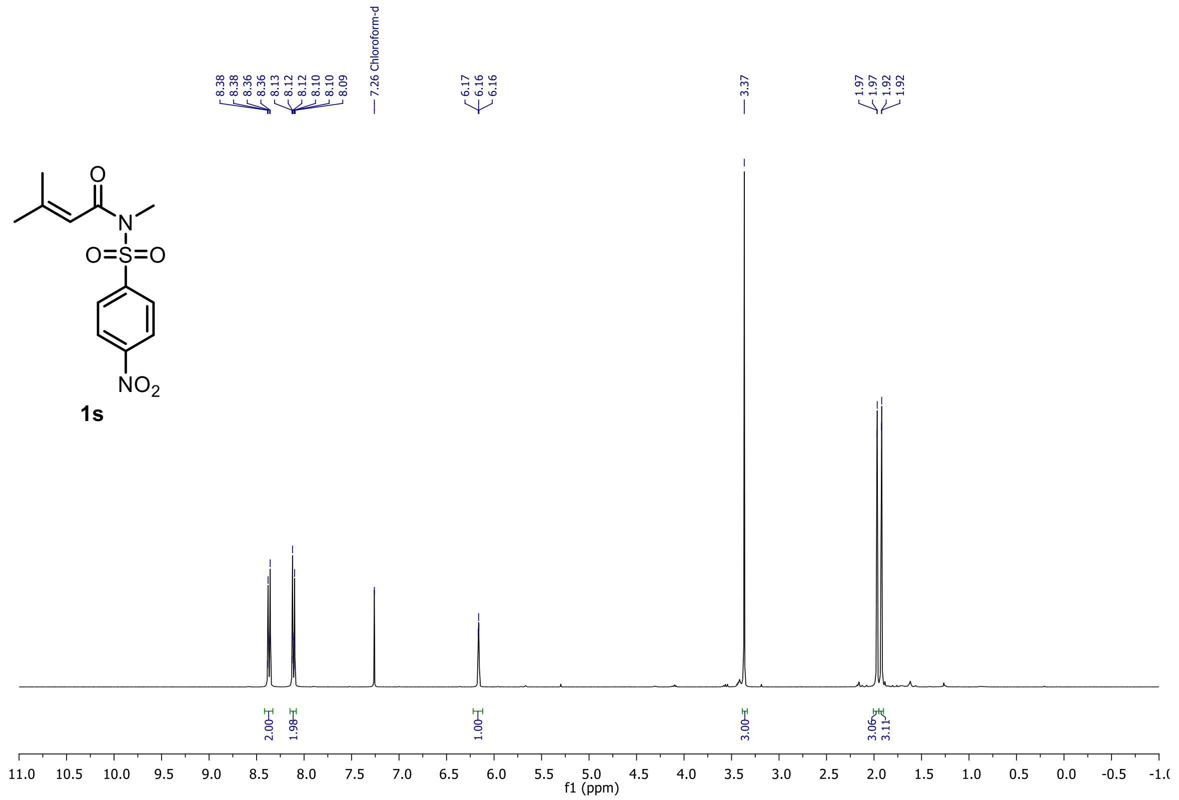

^{13}C NMR (100 MHz, $CDCl_3$)

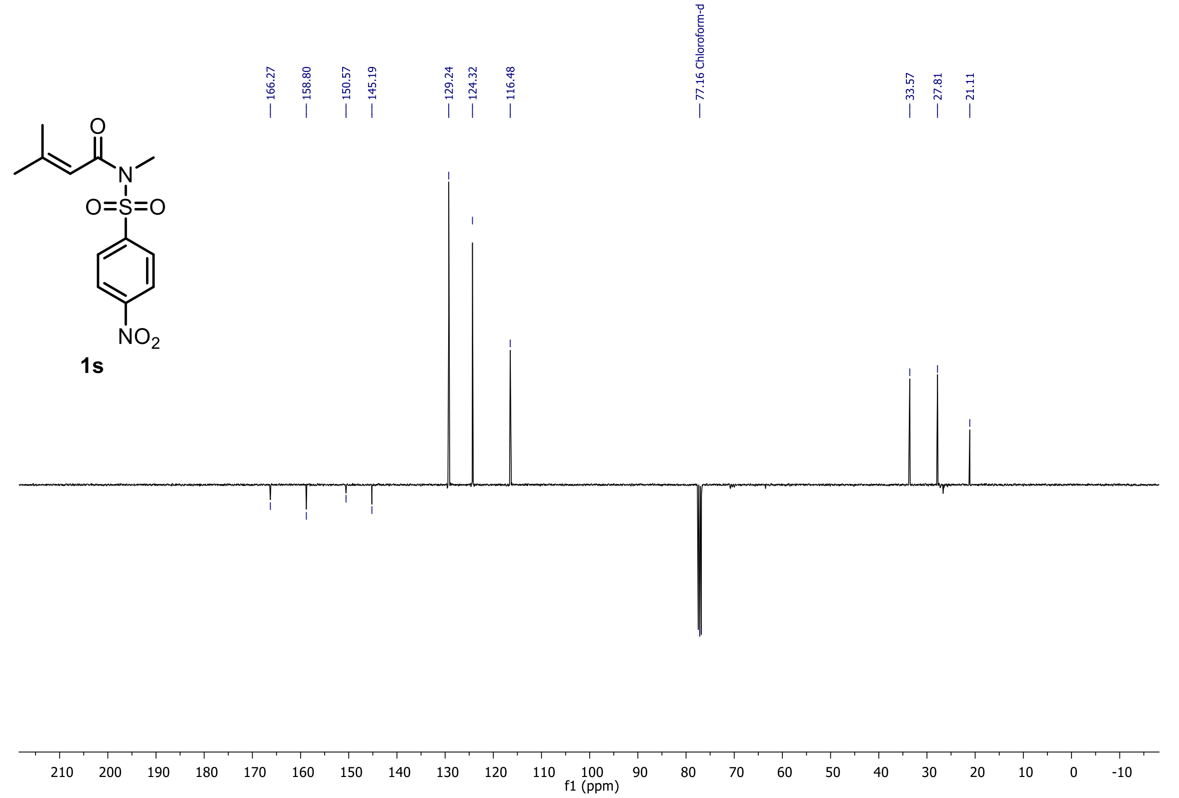

1t: (*E*)-*N*-4-Dimethyl-*N*-((4-nitrophenyl)sulfonyl)pent-2-enamide

^{1}H NMR (400 MHz, $CDCl_3$)

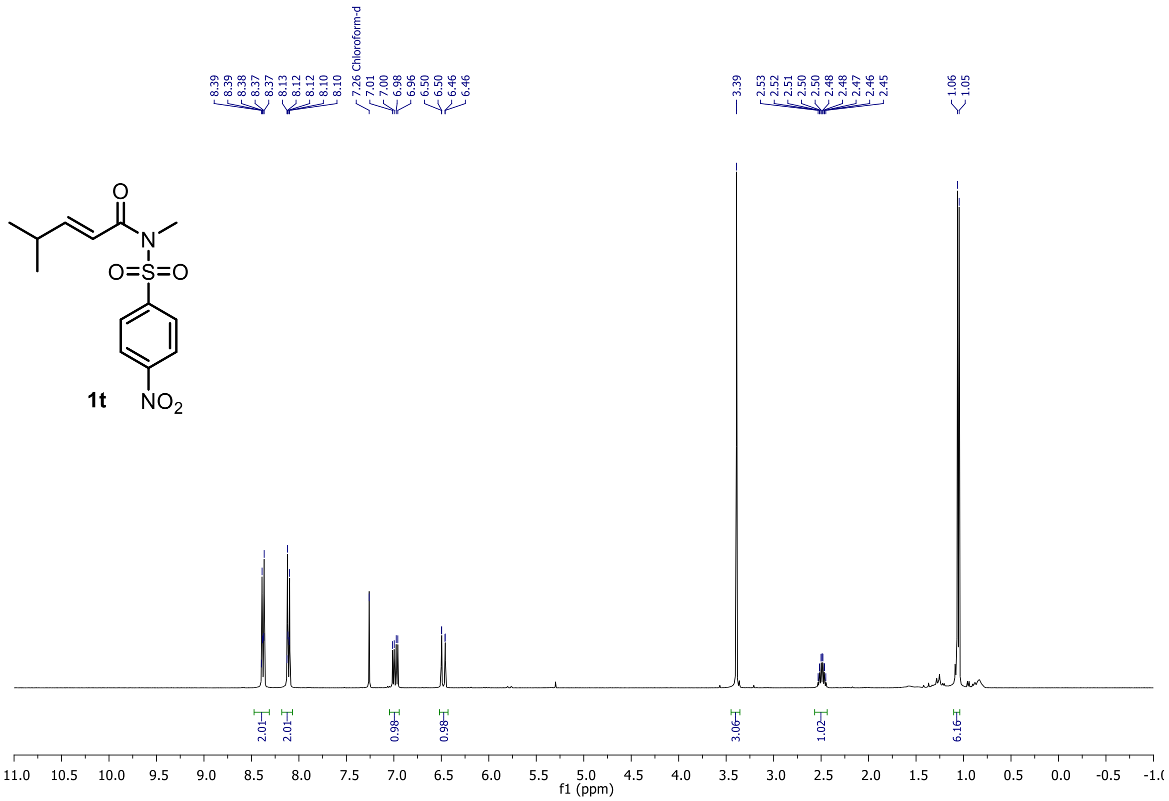

^{13}C NMR (100 MHz, $CDCl_3$)

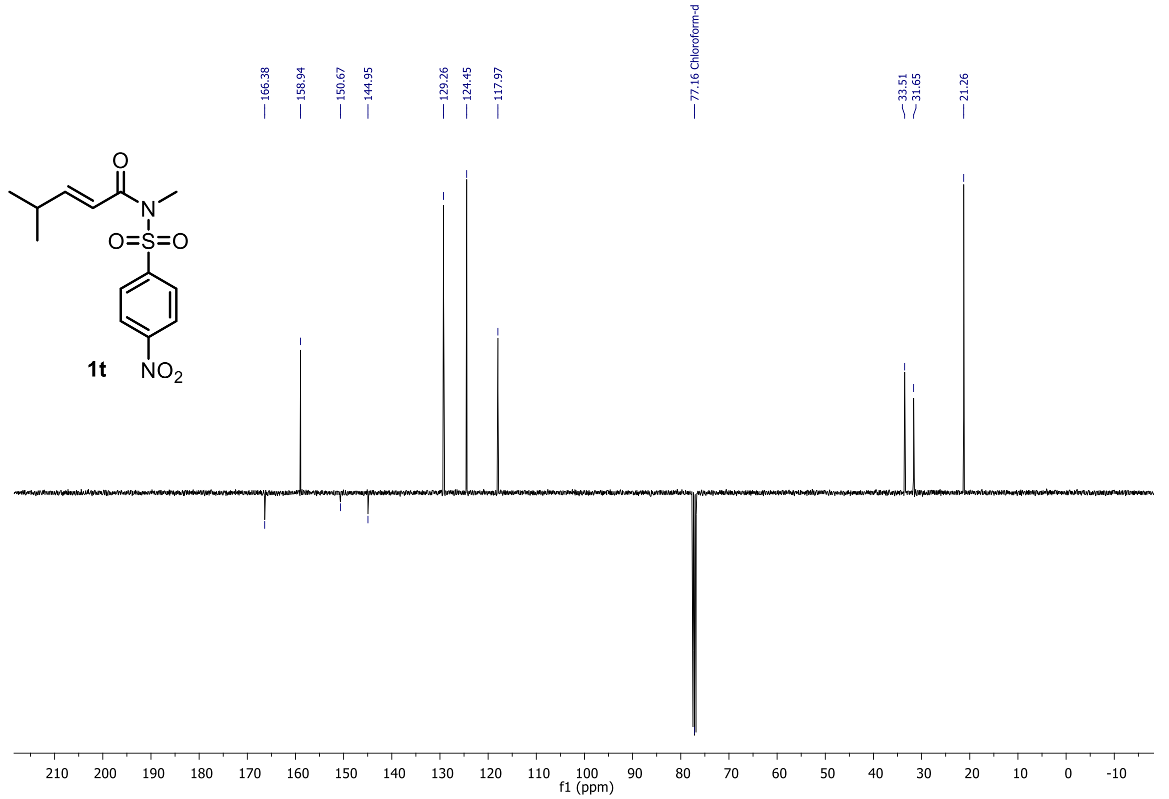

1u: (*2E,4E*)-*N*-Methyl-*N*-((4-nitrophenyl)sulfonyl)hexa-2,4-dienamide

^{1}H NMR (400 MHz, $CDCl_3$)

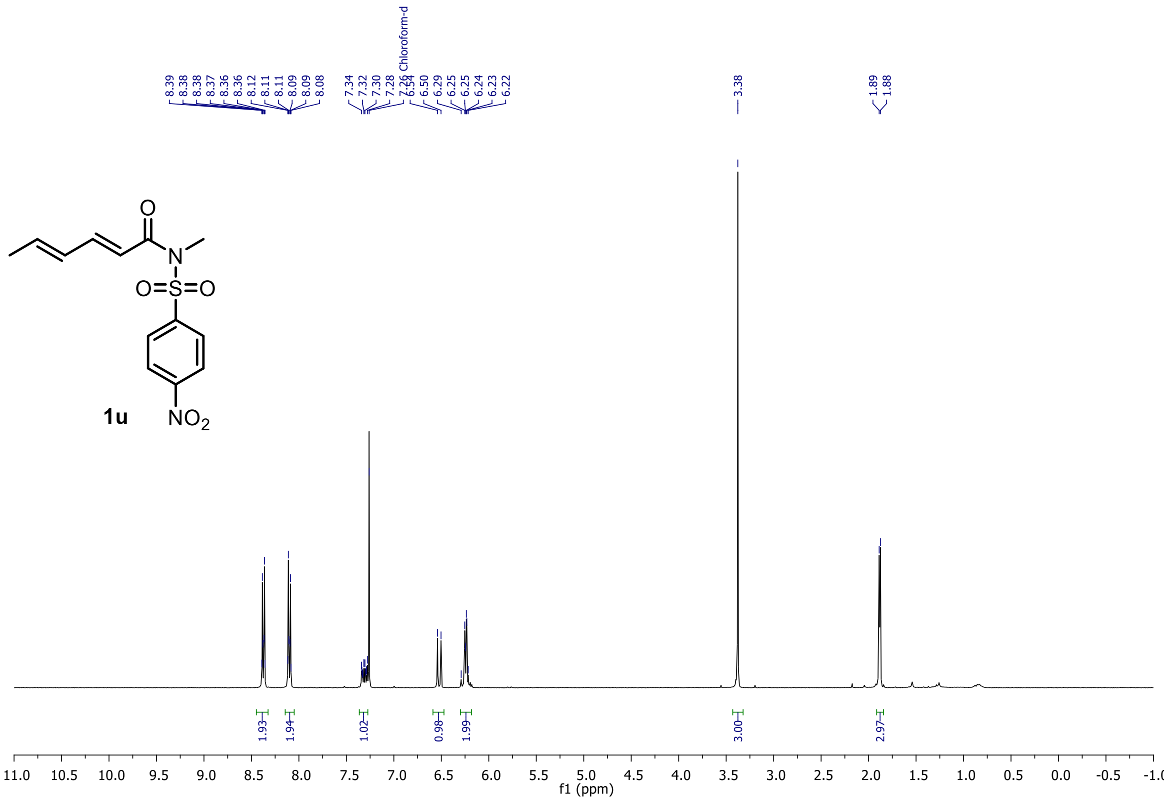

^{13}C NMR (100 MHz, $CDCl_3$)

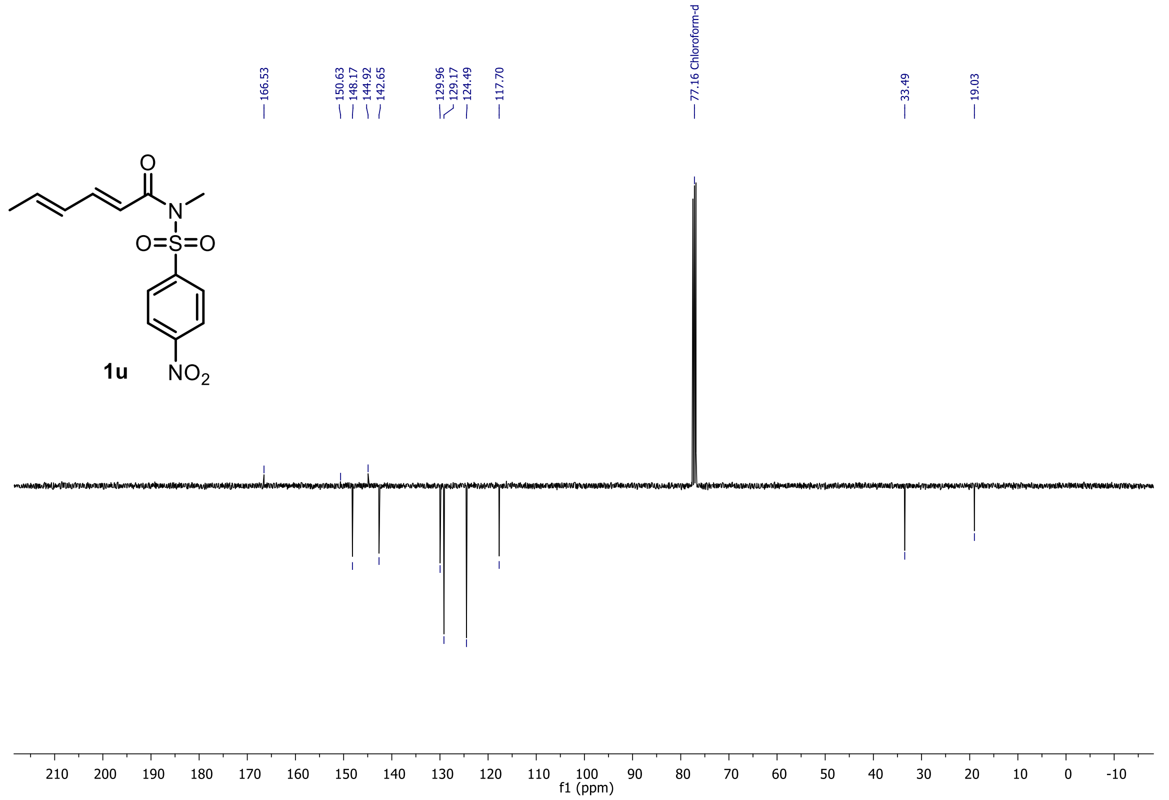

SI2: *N*-((4-Nitrophenyl)sulfonyl)-*N*-phenylacrylamide

^{1}H NMR (400 MHz, CDCl$_3$)

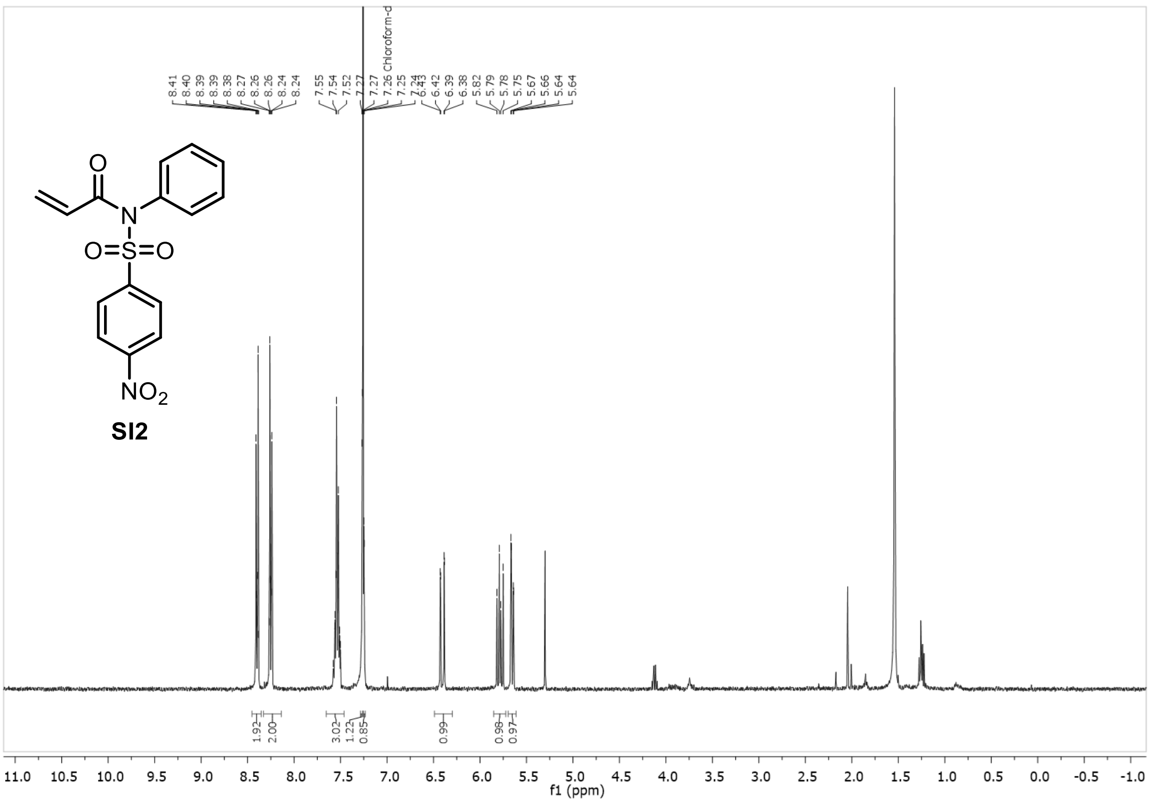

^{13}C NMR (100 MHz, CDCl$_3$)

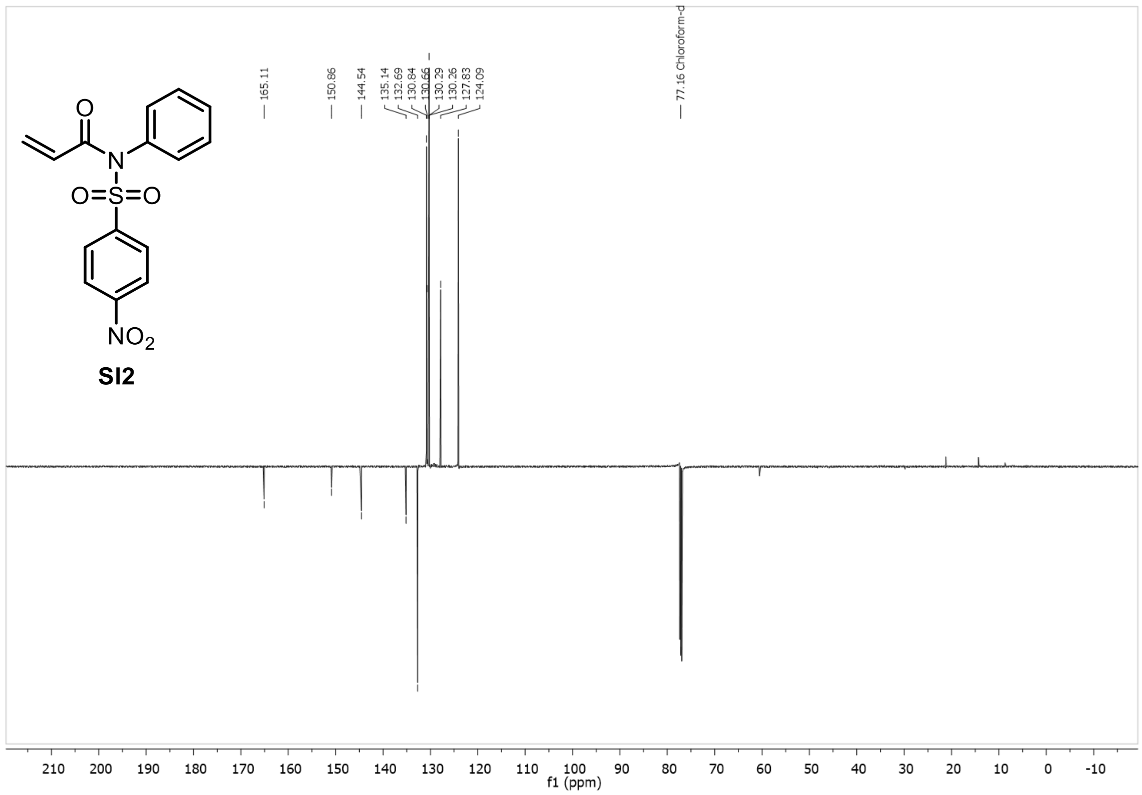

SI3: *N*-((4-Cyanophenyl)sulfonyl)-*N*-methylacrylamide

^{1}H NMR (400 MHz, $CDCl_3$)

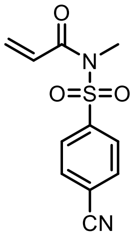

SI3

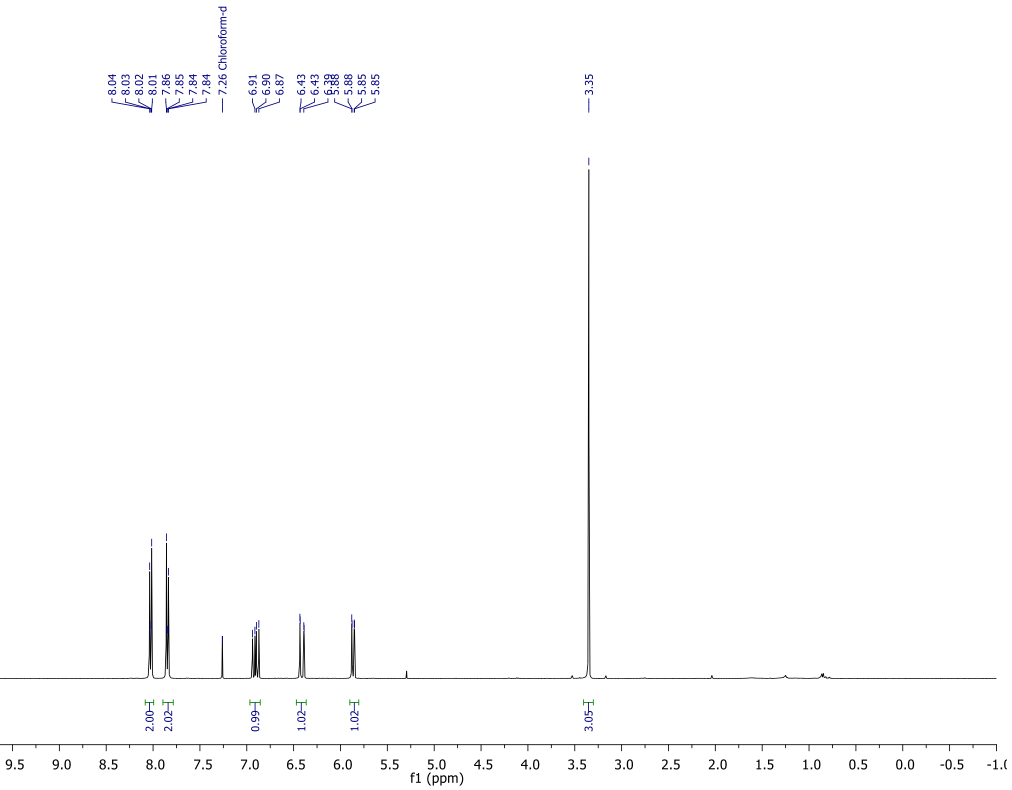

^{13}C NMR (100 MHz, $CDCl_3$)

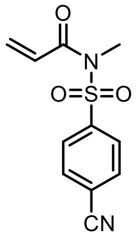

SI3

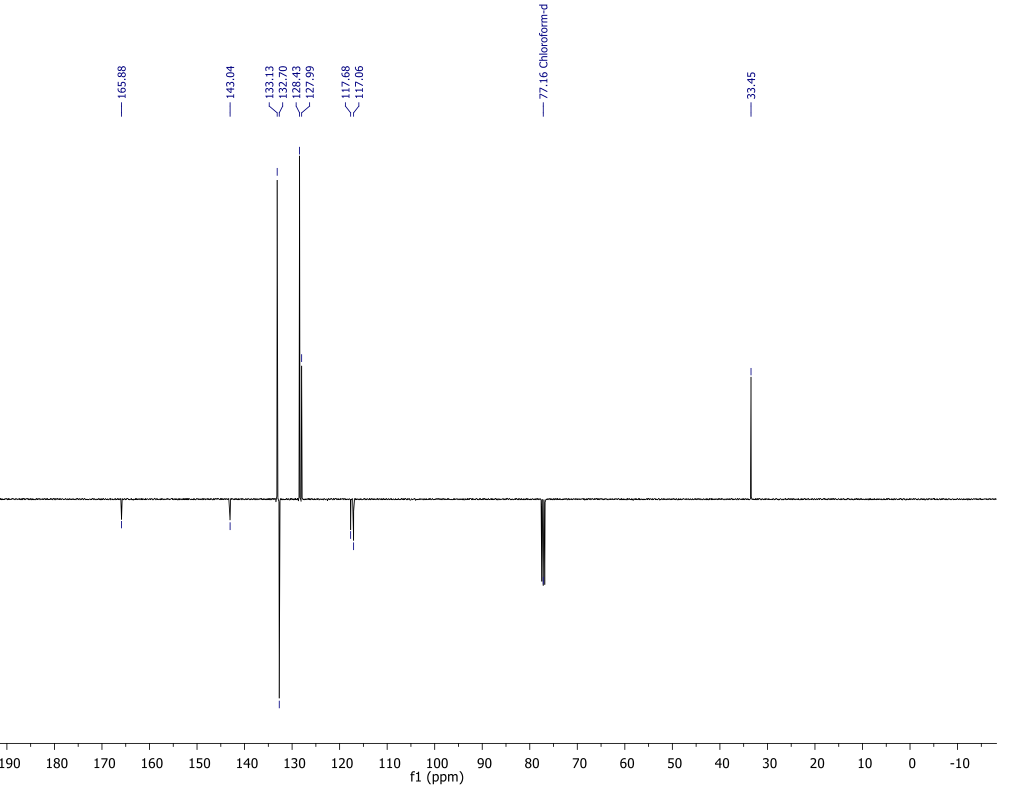

2a: *N*-Methyl-2-(4-nitrophenyl)acrylamide

^{1}H NMR (400 MHz, $CDCl_3$)

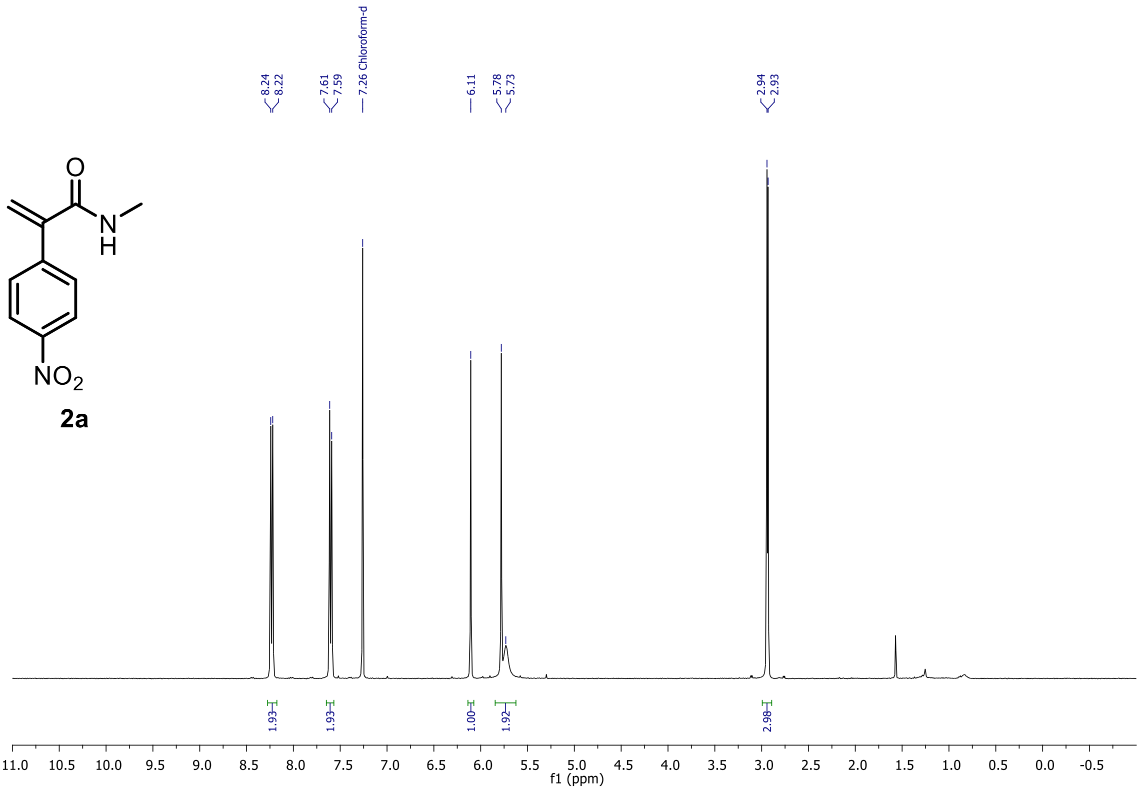

^{13}C NMR (150 MHz, $CDCl_3$)

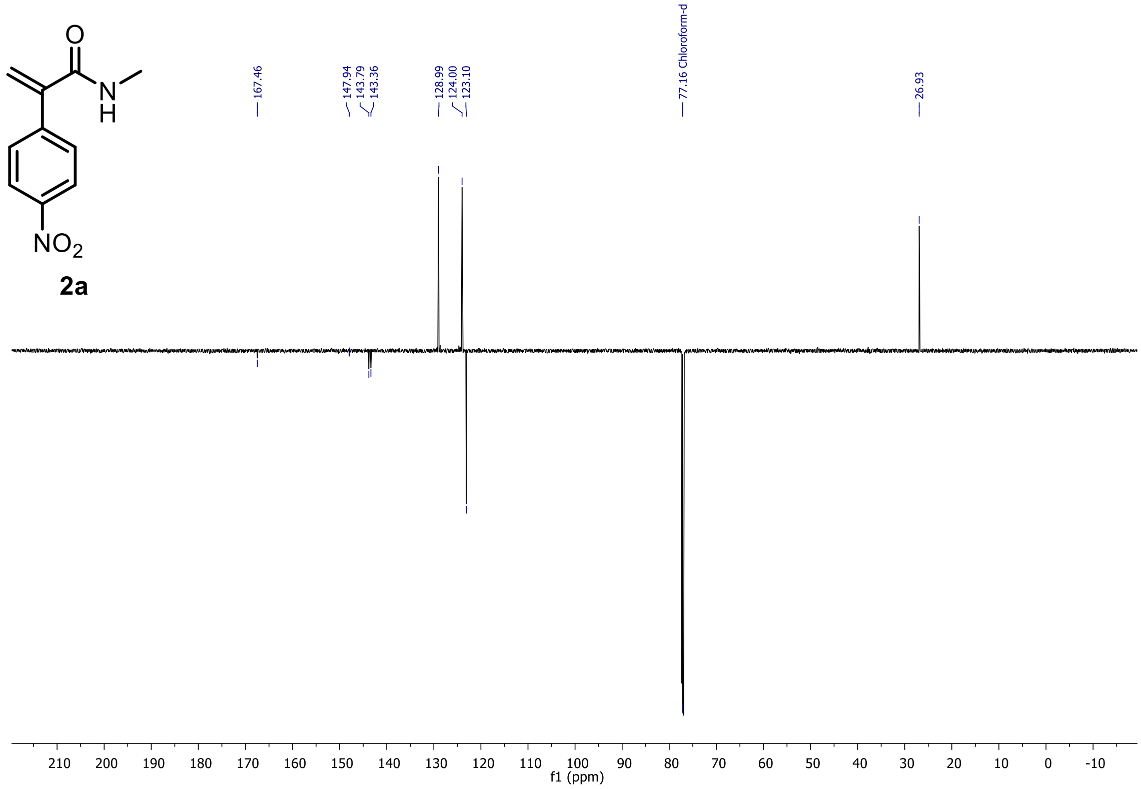

2b: *N*-Isopropyl-2-(4-nitrophenyl)acrylamide

^{1}H NMR (400 MHz, $CDCl_3$)

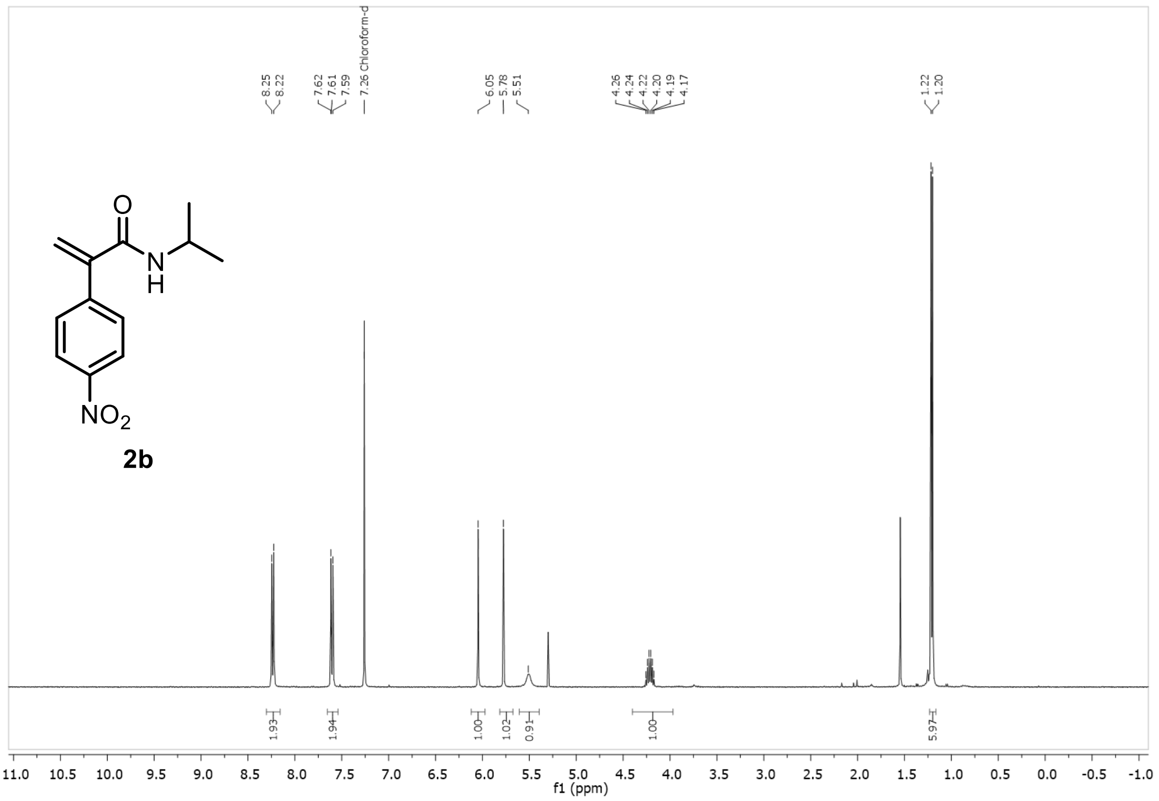

^{13}C NMR (100 MHz, $CDCl_3$)

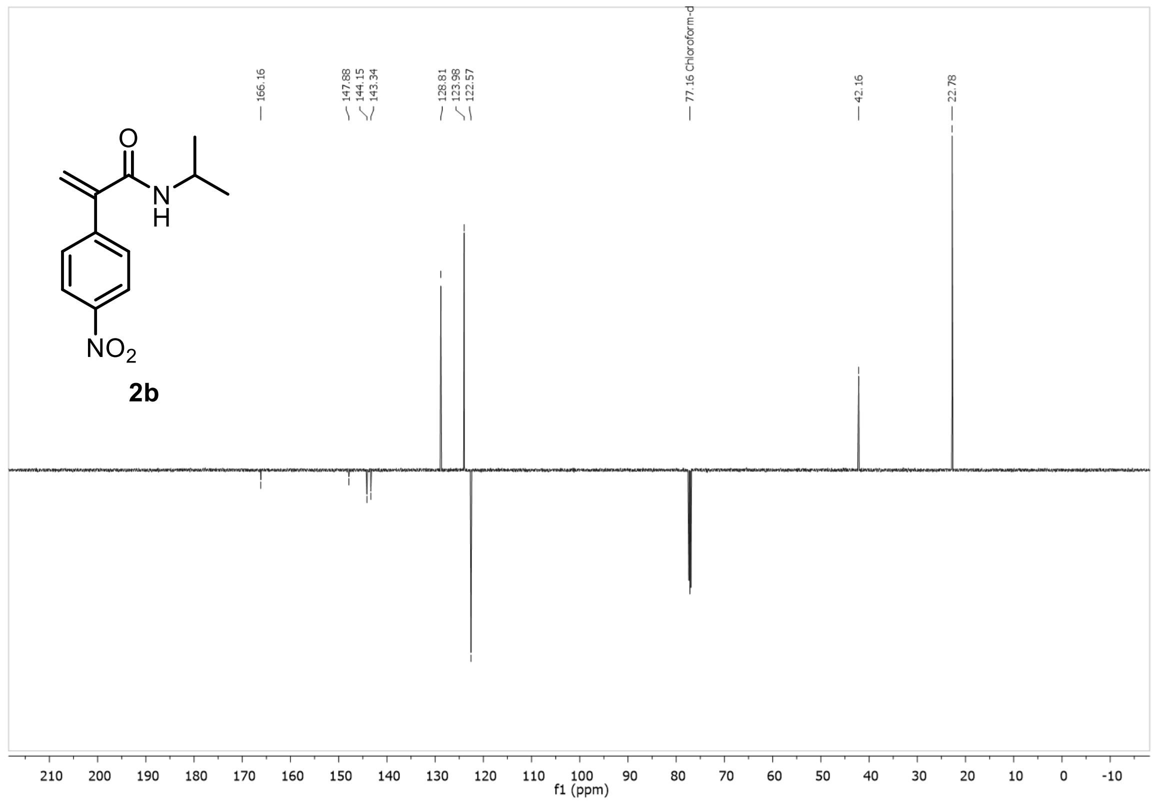

2c: *N*-(*tert*-Butyl)-2-(4-nitrophenyl)acrylamide

^{1}H NMR (400 MHz, $CDCl_3$)

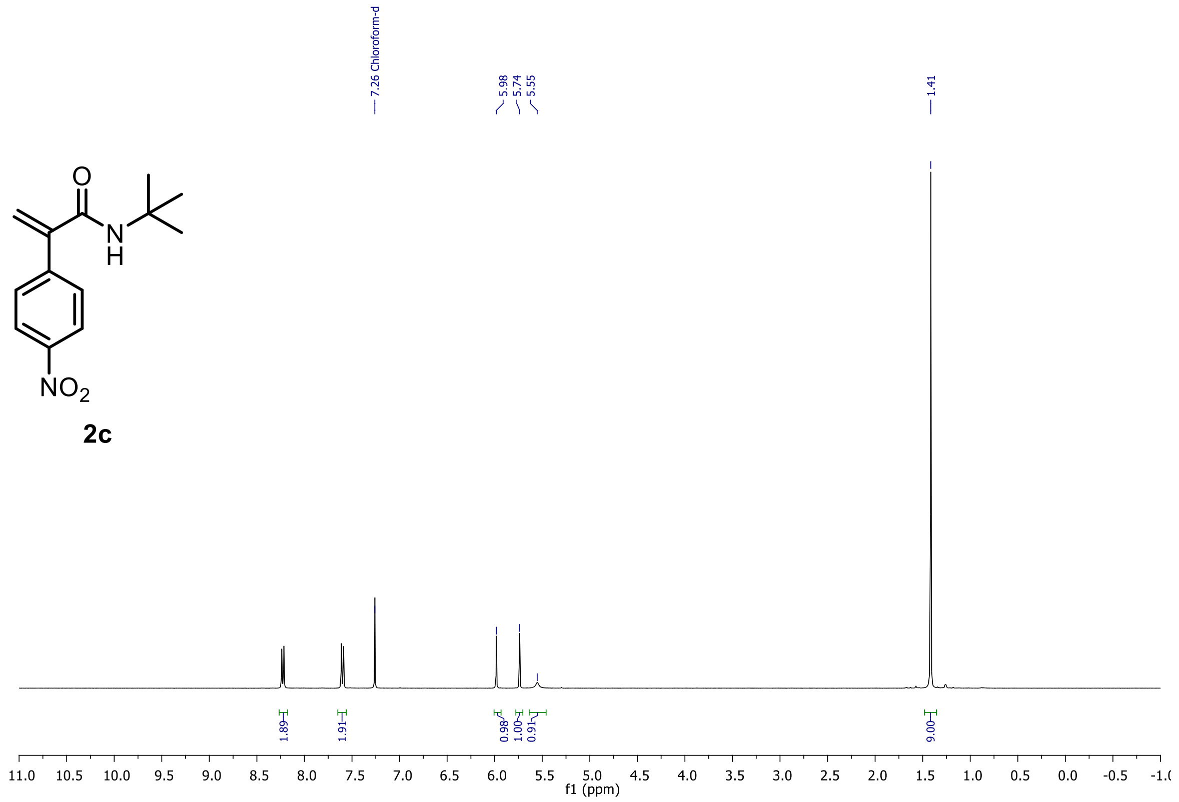

^{13}C NMR (100 MHz, $CDCl_3$)

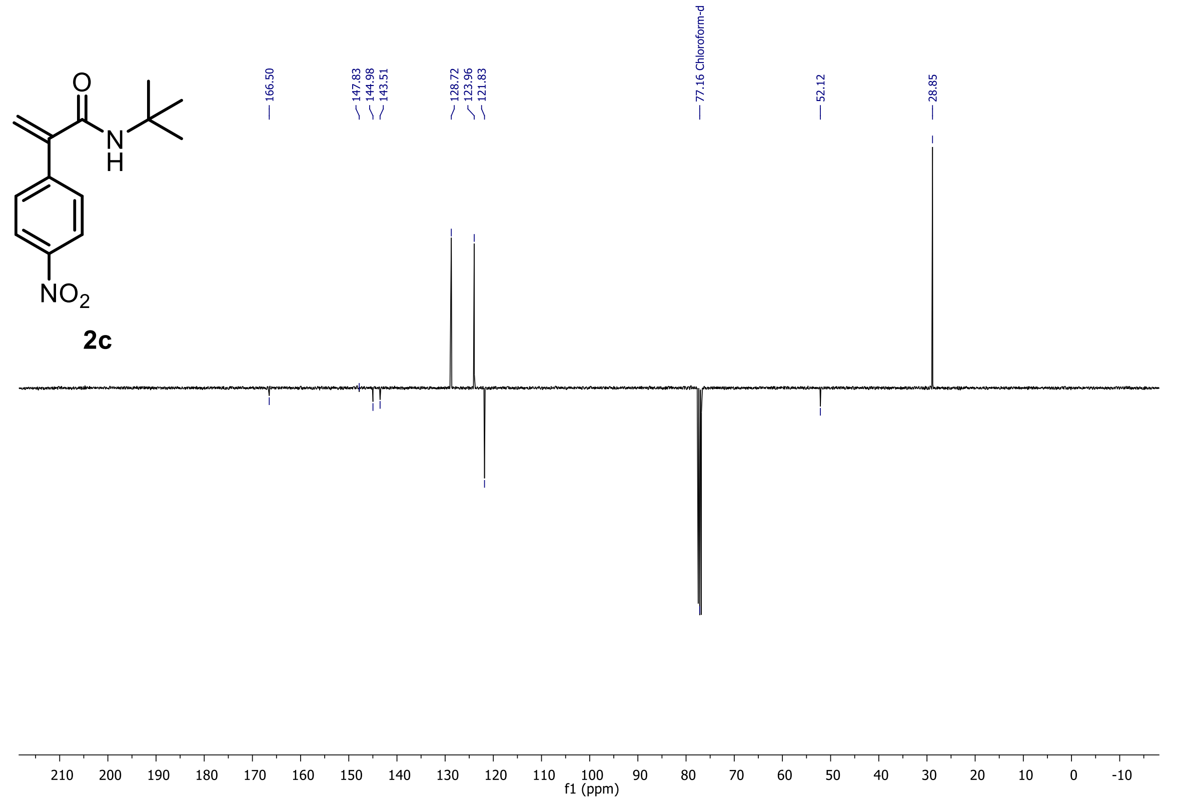

2d: *N*-Allyl-2-(4-nitrophenyl)acrylamide

^{1}H NMR (400 MHz, CDCl$_3$)

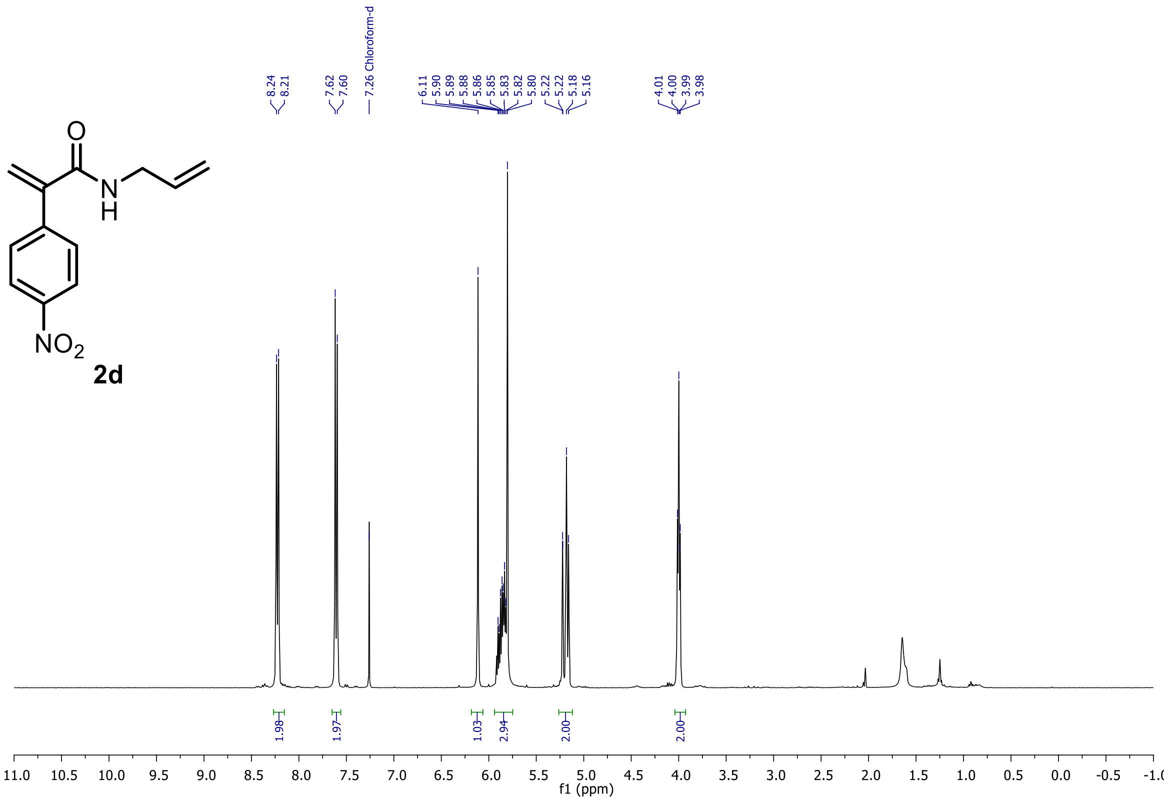

^{13}C NMR (100 MHz, CDCl$_3$)

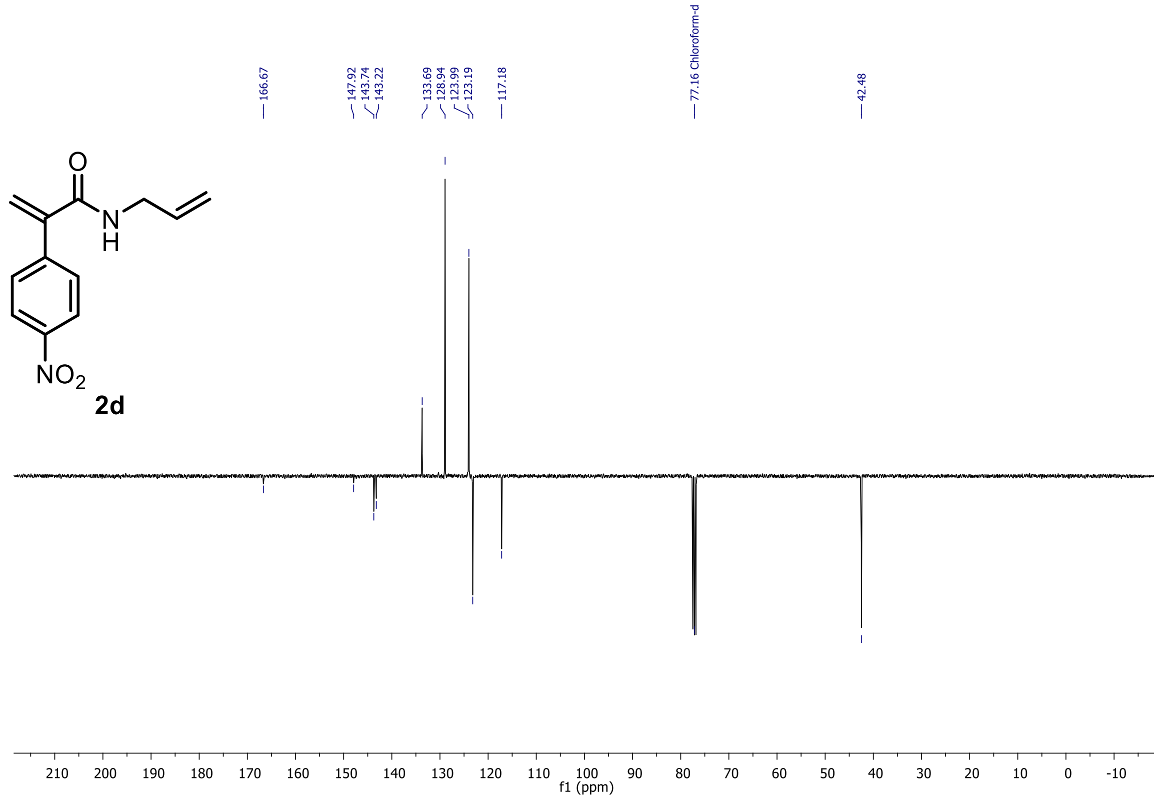

2e: *N*-Benzyl-2-(4-nitrophenyl)acrylamide

^{1}H NMR (400 MHz, CDCl$_3$)

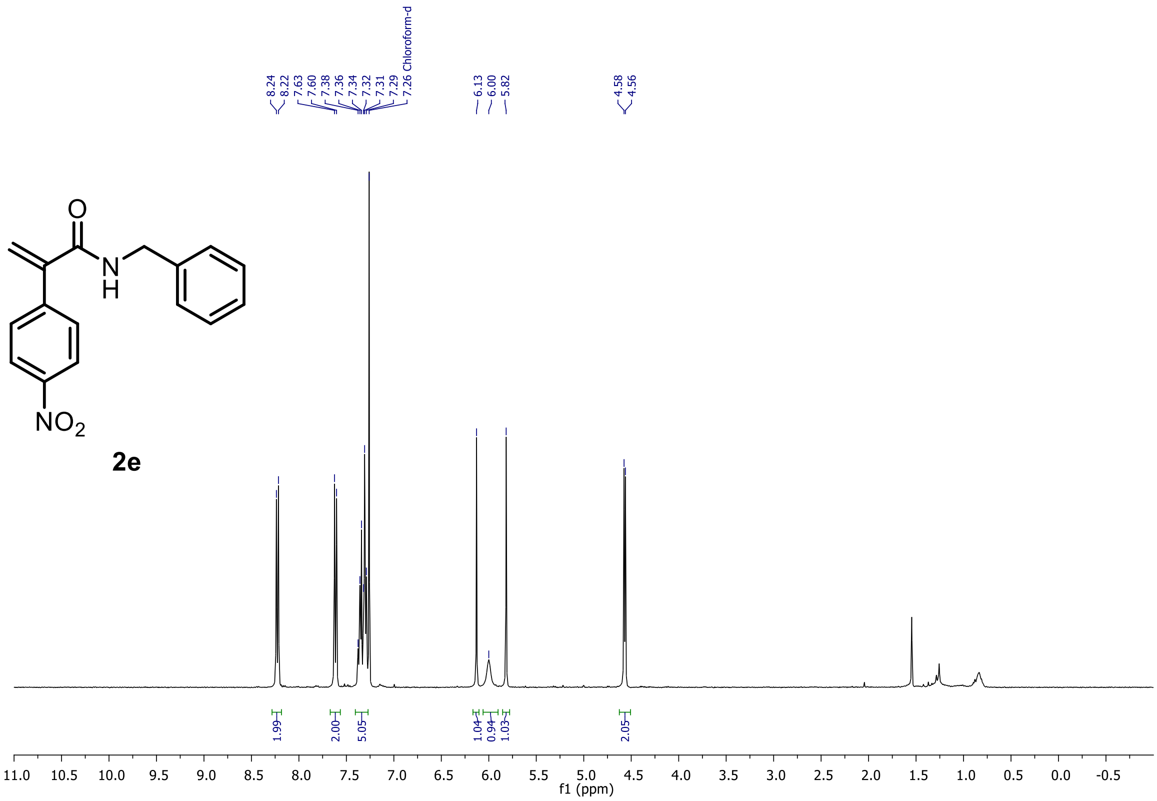

^{13}C NMR (100 MHz, CDCl$_3$)

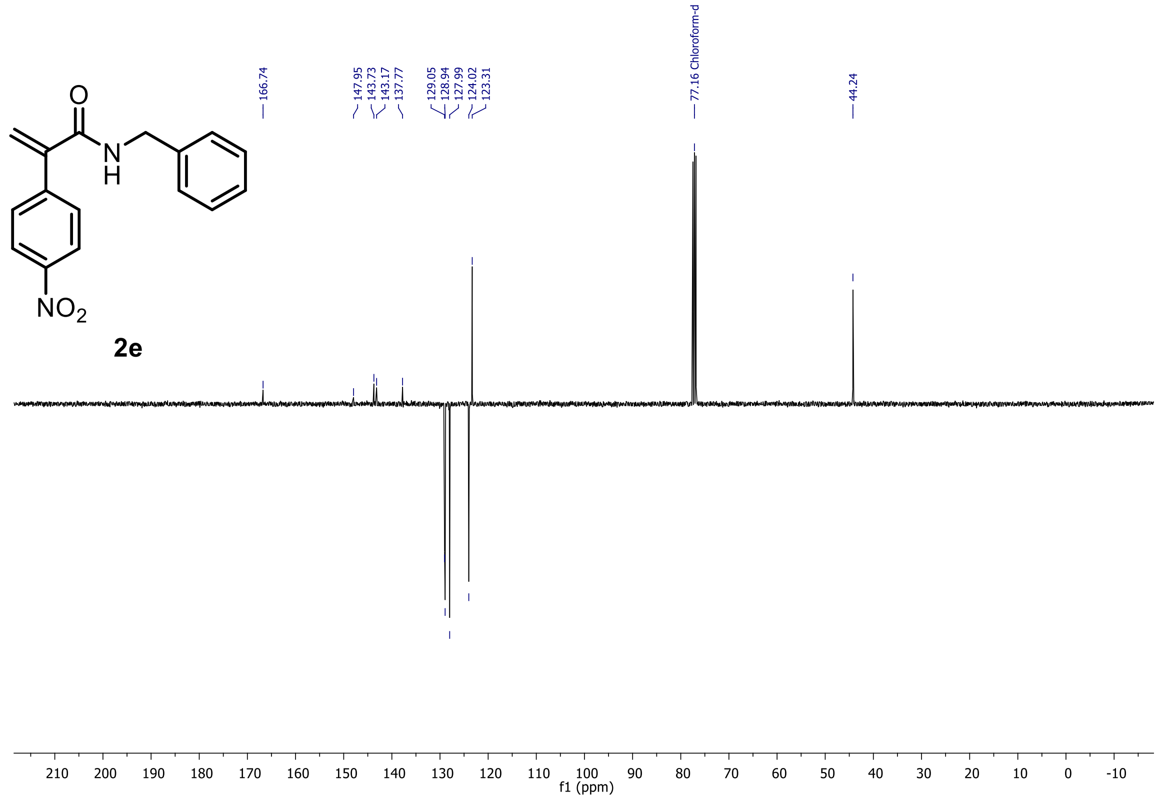

2f: *N*-(4,4-Dimethoxybutyl)-2-(4-nitrophenyl)acrylamide

^1H NMR (400 MHz, $CDCl_3$)

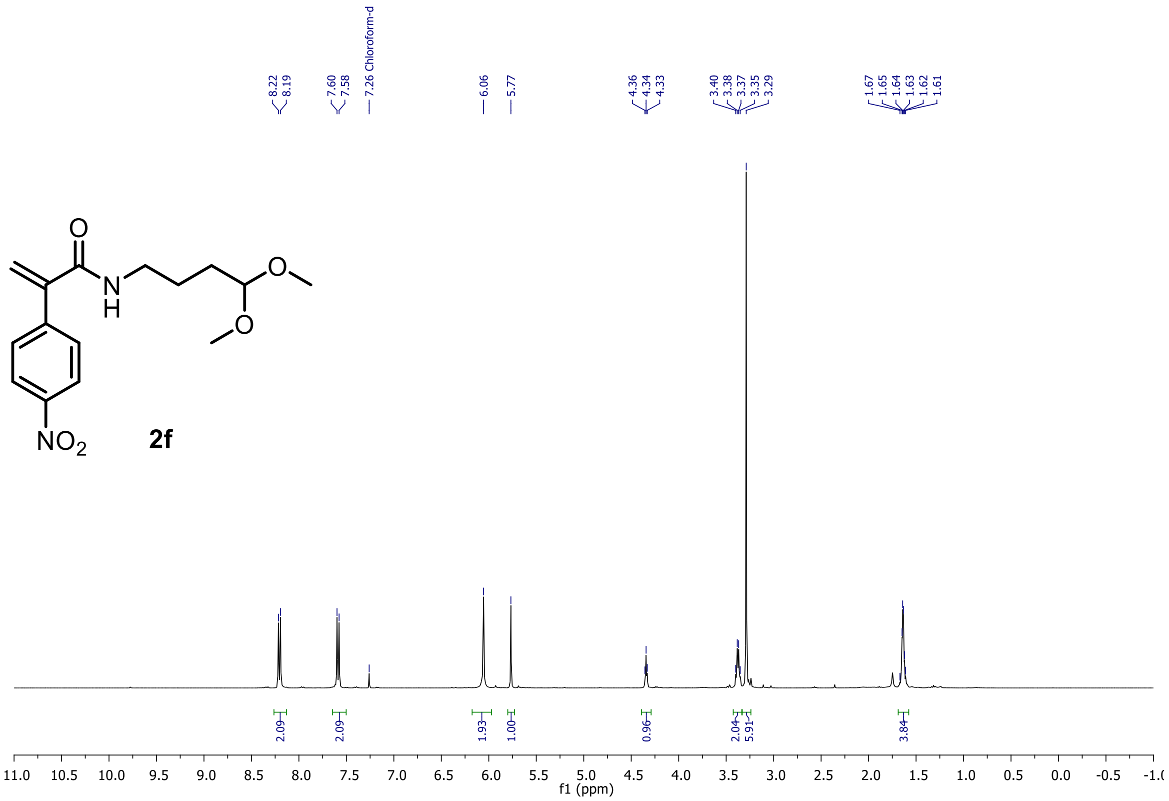

^{13}C NMR (100 MHz, $CDCl_3$)

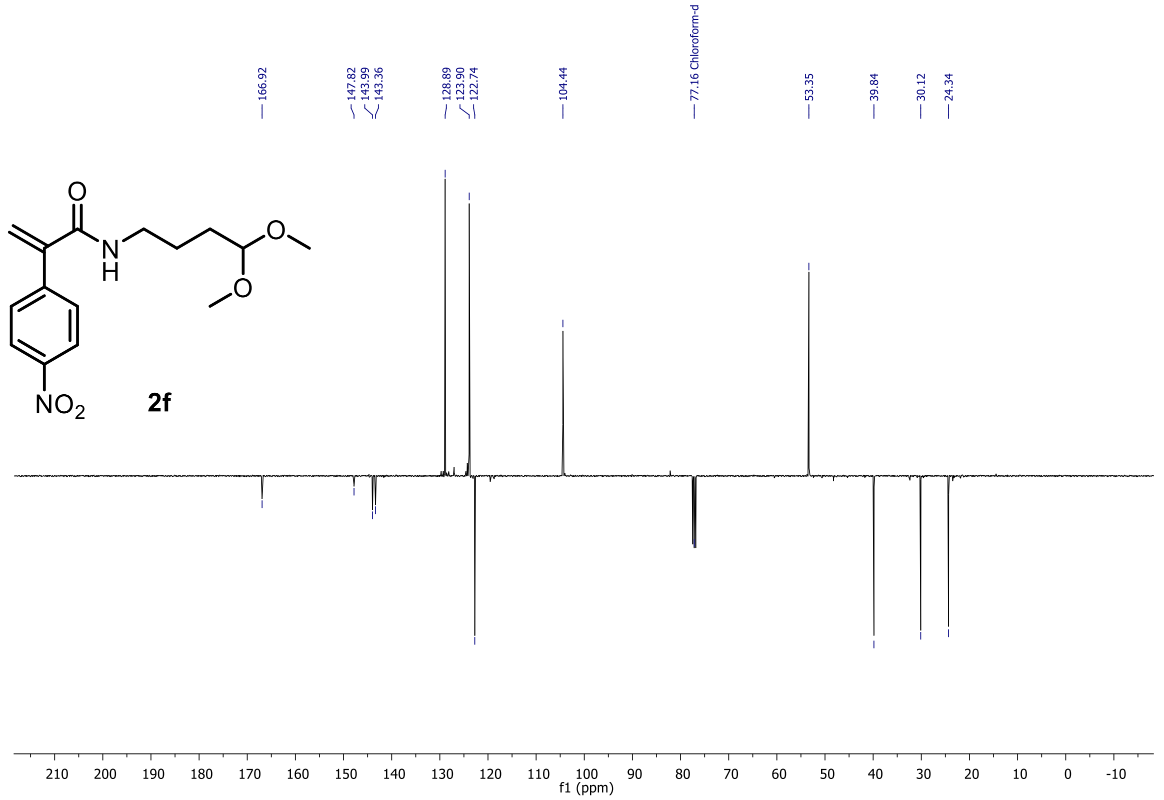

2g: Methyl-(2-(4-nitrophenyl)acryloyl)glycinate

^{1}H NMR (400 MHz, $CDCl_3$)

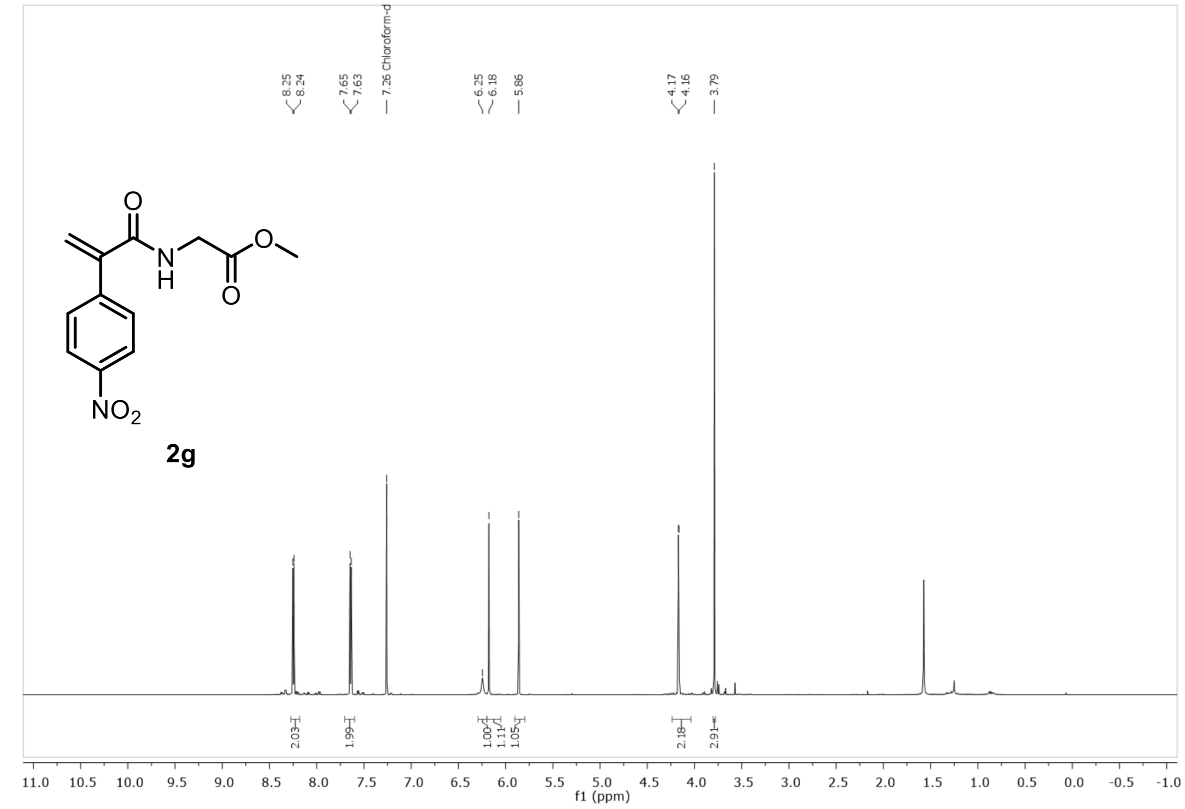

^{13}C NMR (100 MHz, $CDCl_3$)

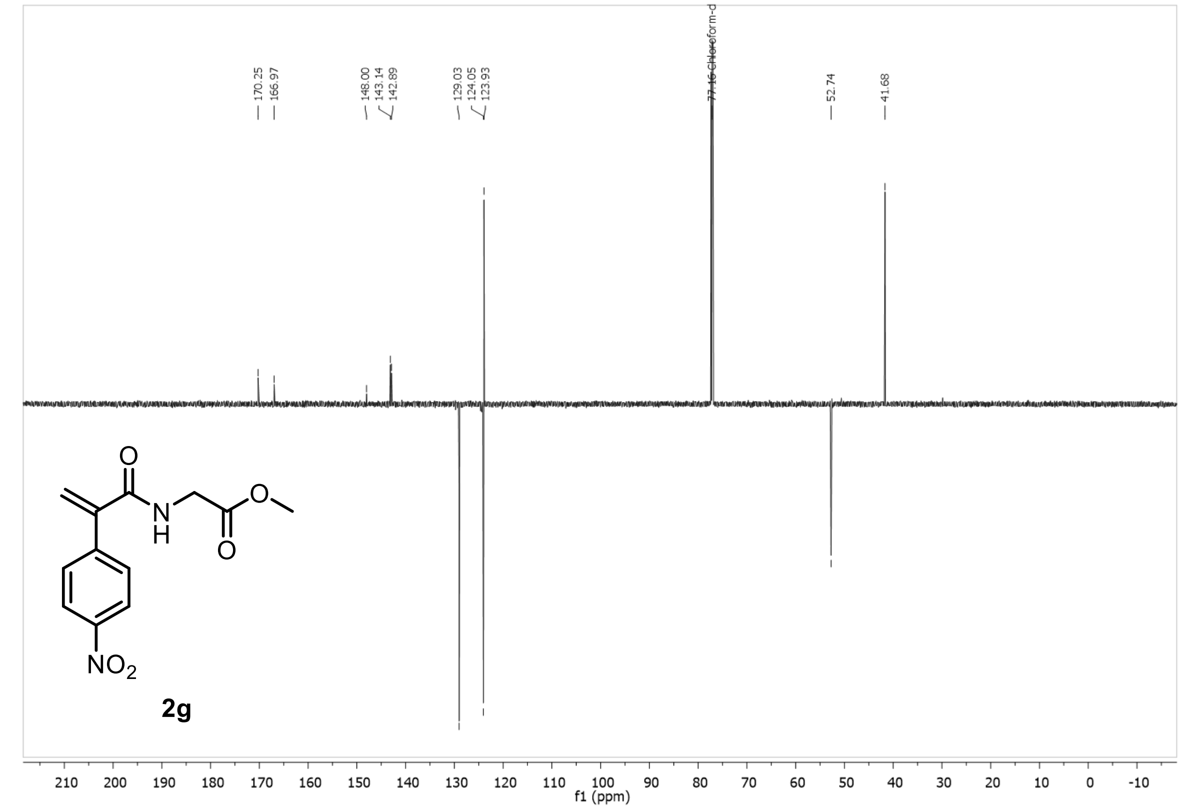

2h: Methyl-(2-(4-nitrophenyl)acryloyl)methioninate

^{1}H NMR (400 MHz, $CDCl_3$)

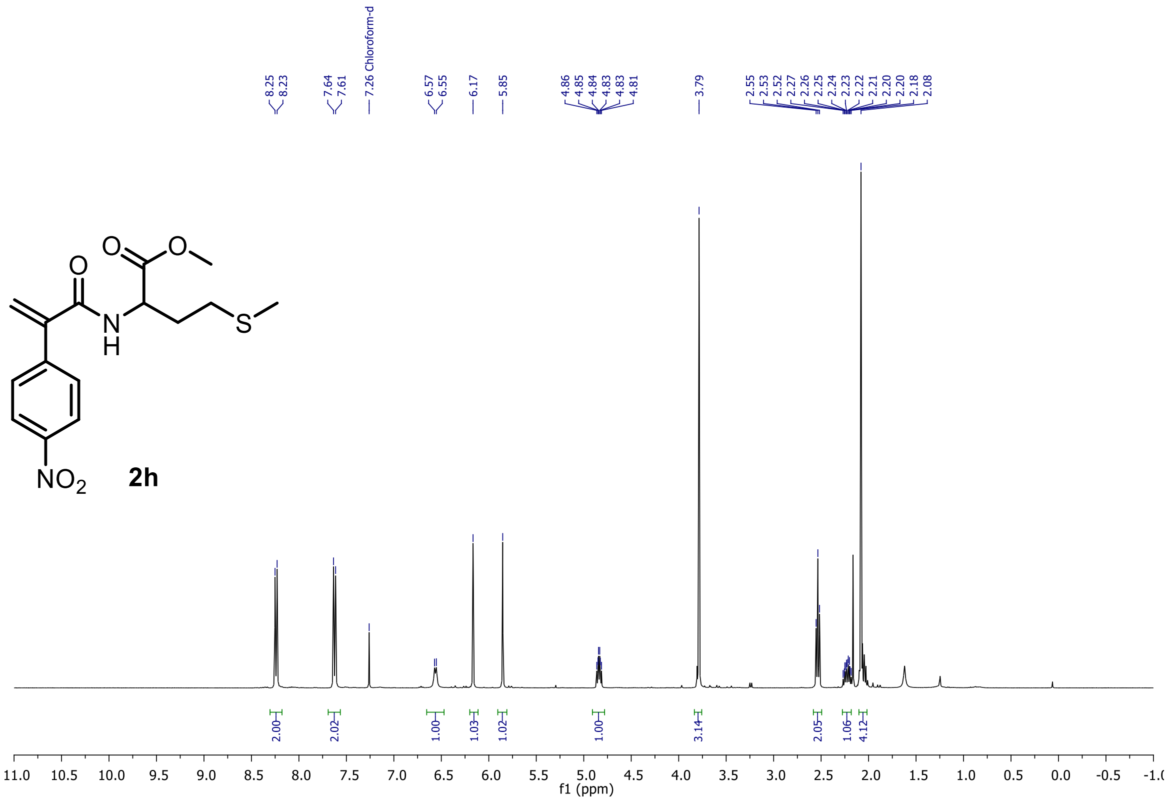

^{13}C NMR (100 MHz, $CDCl_3$)

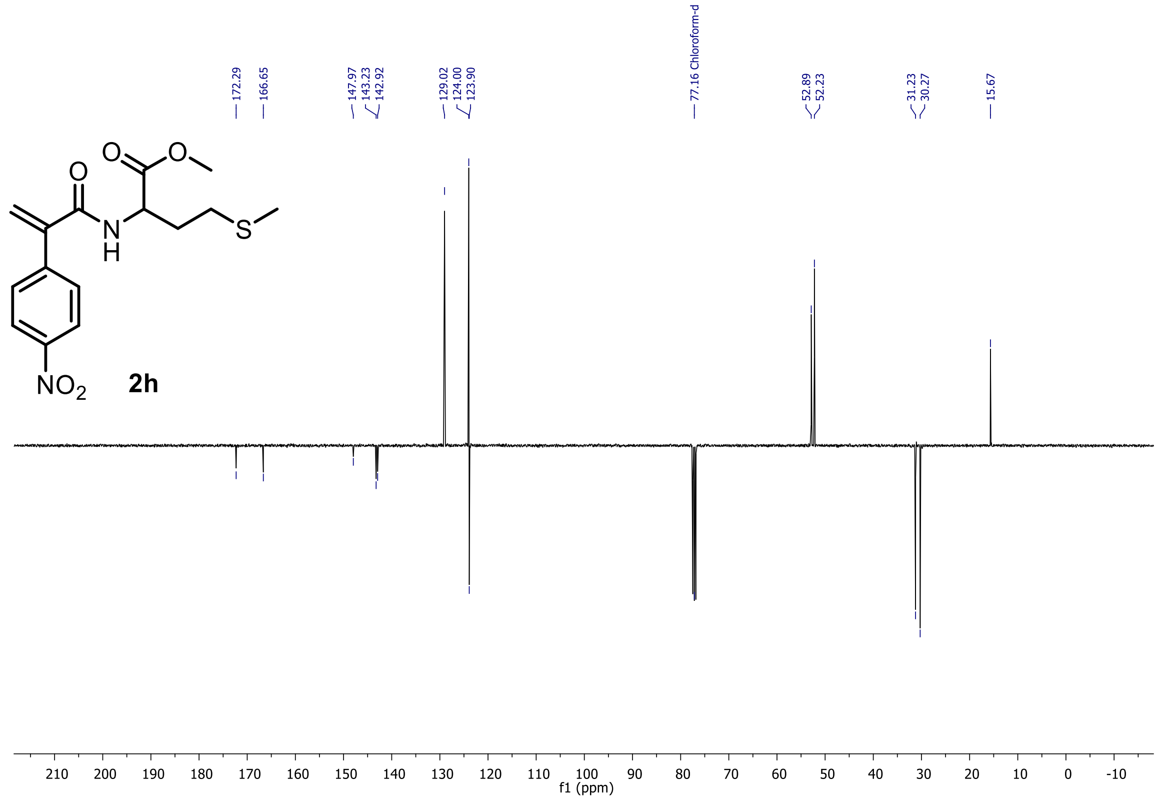

2i: *N*-(3,4-Dimethoxyphenethyl)-2-(4-nitrophenyl)acrylamide

^{1}H NMR (400 MHz, CDCl$_3$)

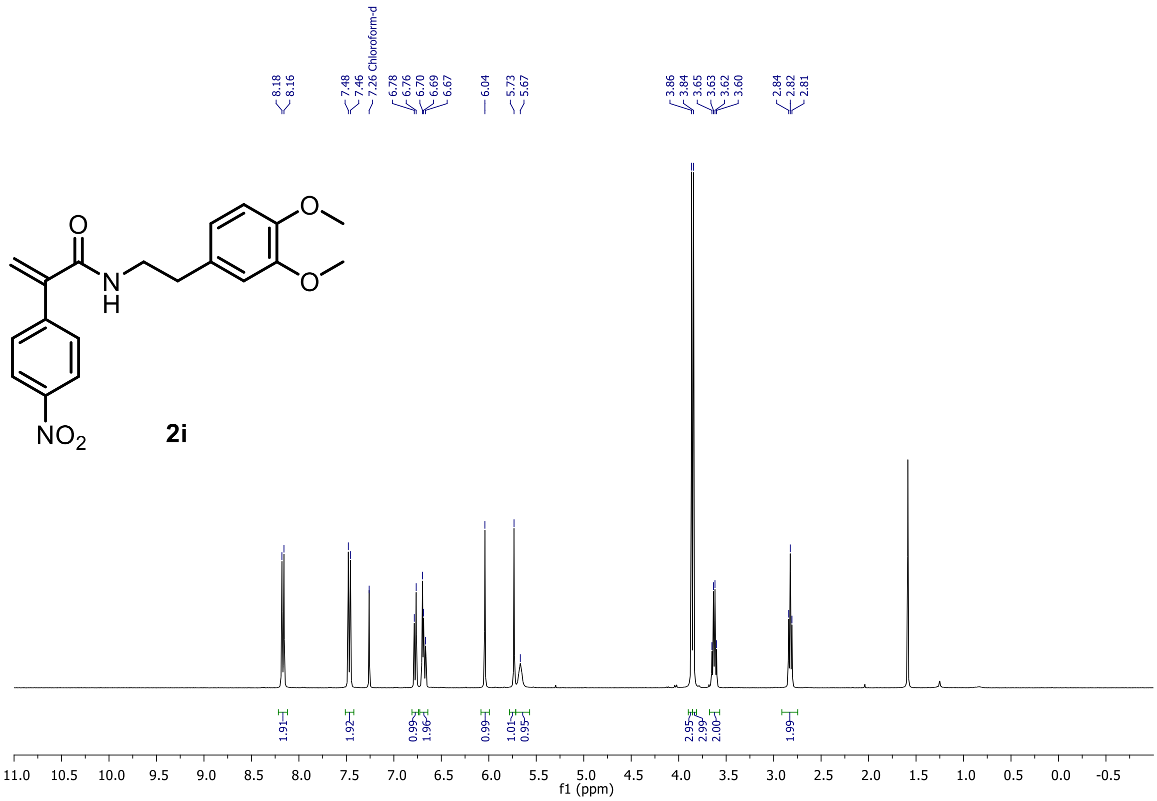

^{13}C NMR (100 MHz, CDCl$_3$)

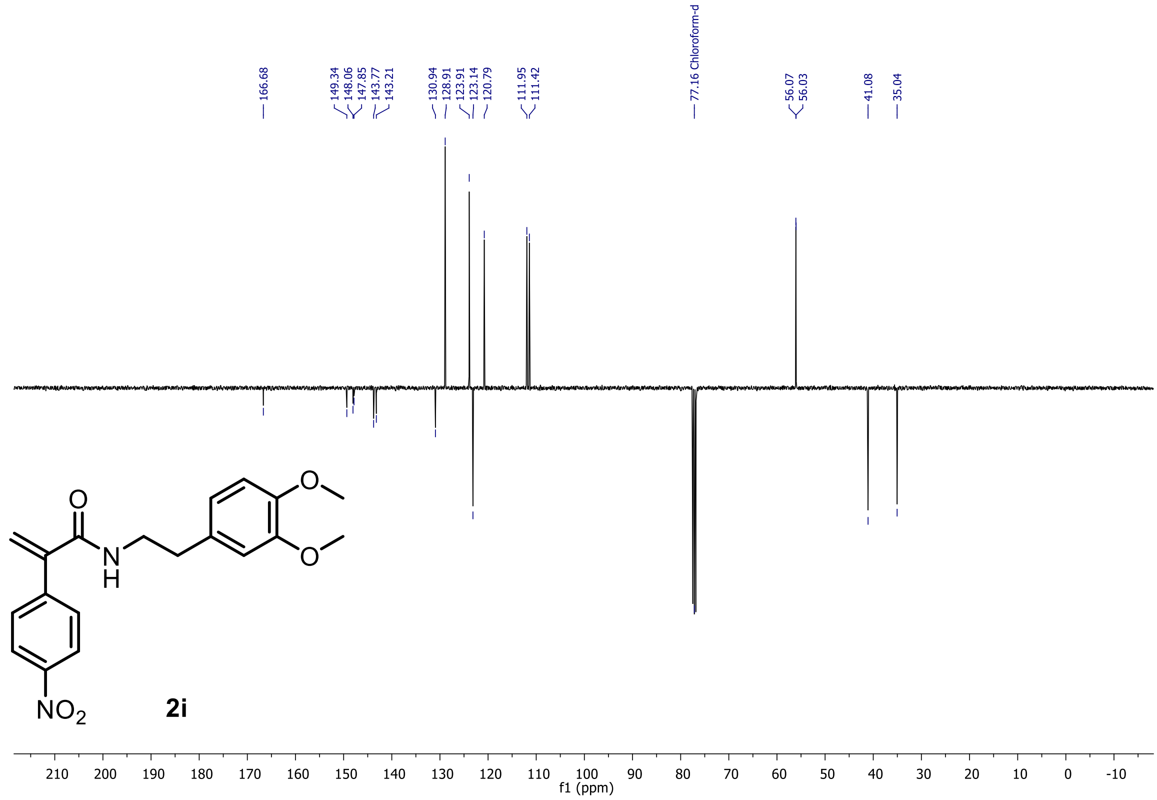

2j: *tert*-Butyl 3-(2-(2-(4-nitrophenyl)acrylamido)ethyl)-1H-indole-1-carboxylate

^{1}H NMR (400 MHz, $CDCl_3$)

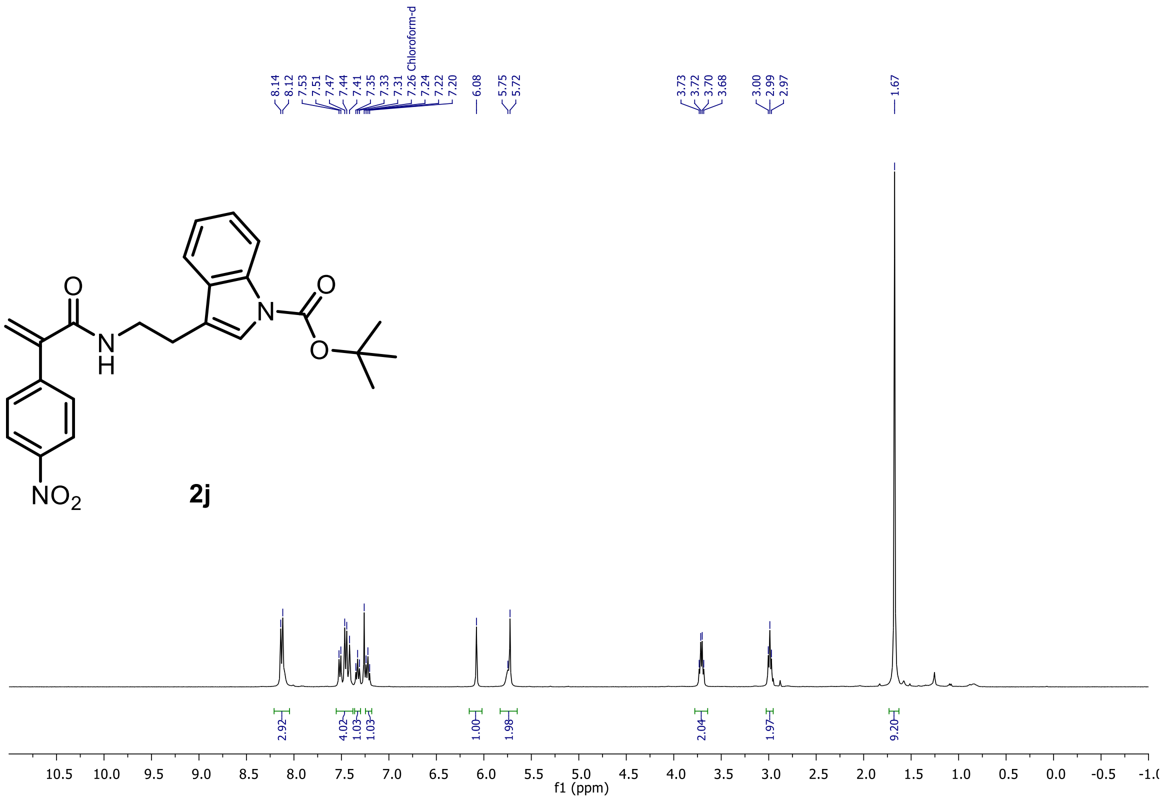

^{13}C NMR (100 MHz, $CDCl_3$)

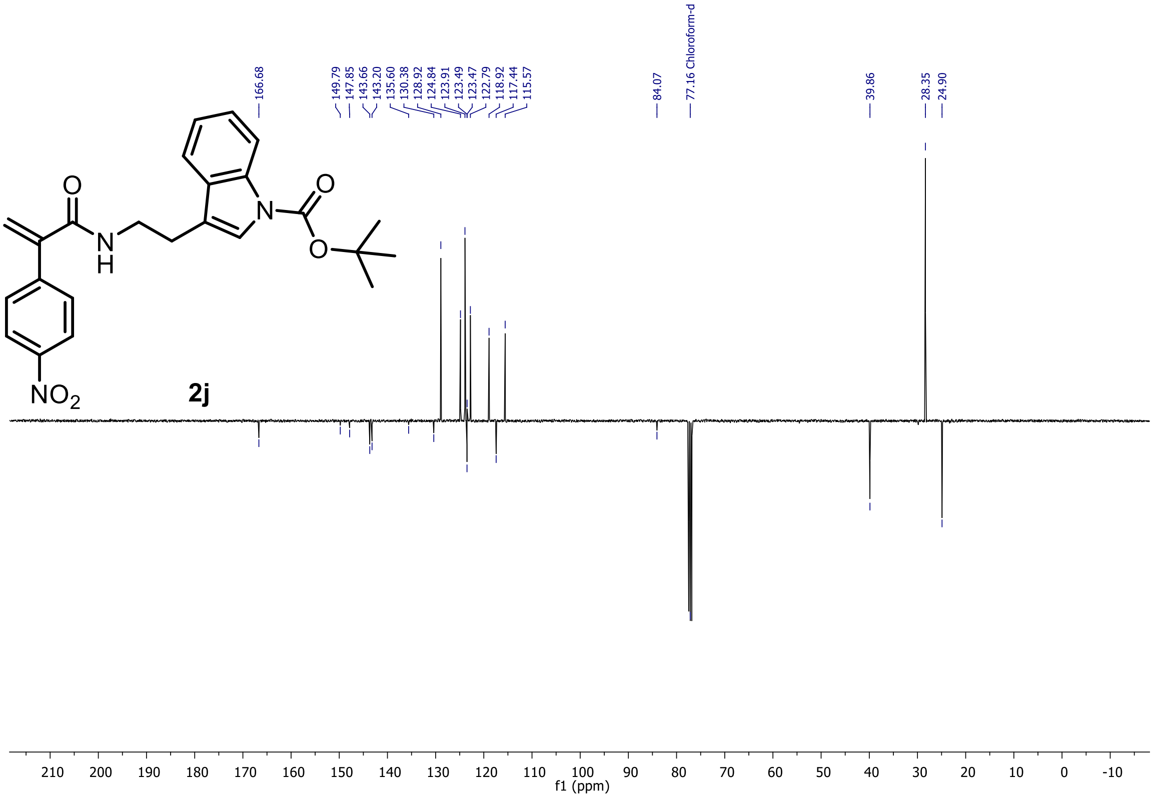

2k: Ethyl-(*E*)-6-(2-(4-nitrophenyl)acrylamido)hex-2-enoate

^{1}H NMR (600 MHz, $CDCl_3$)

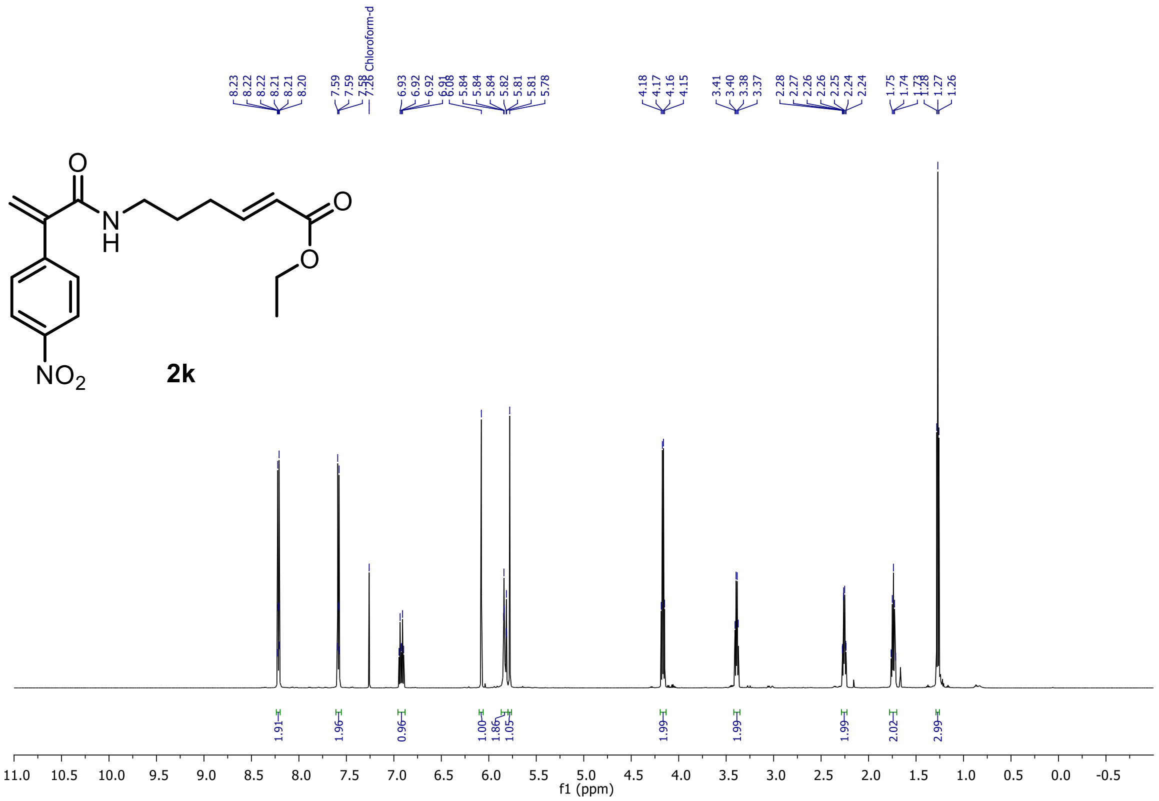

^{13}C NMR (150 MHz, $CDCl_3$)

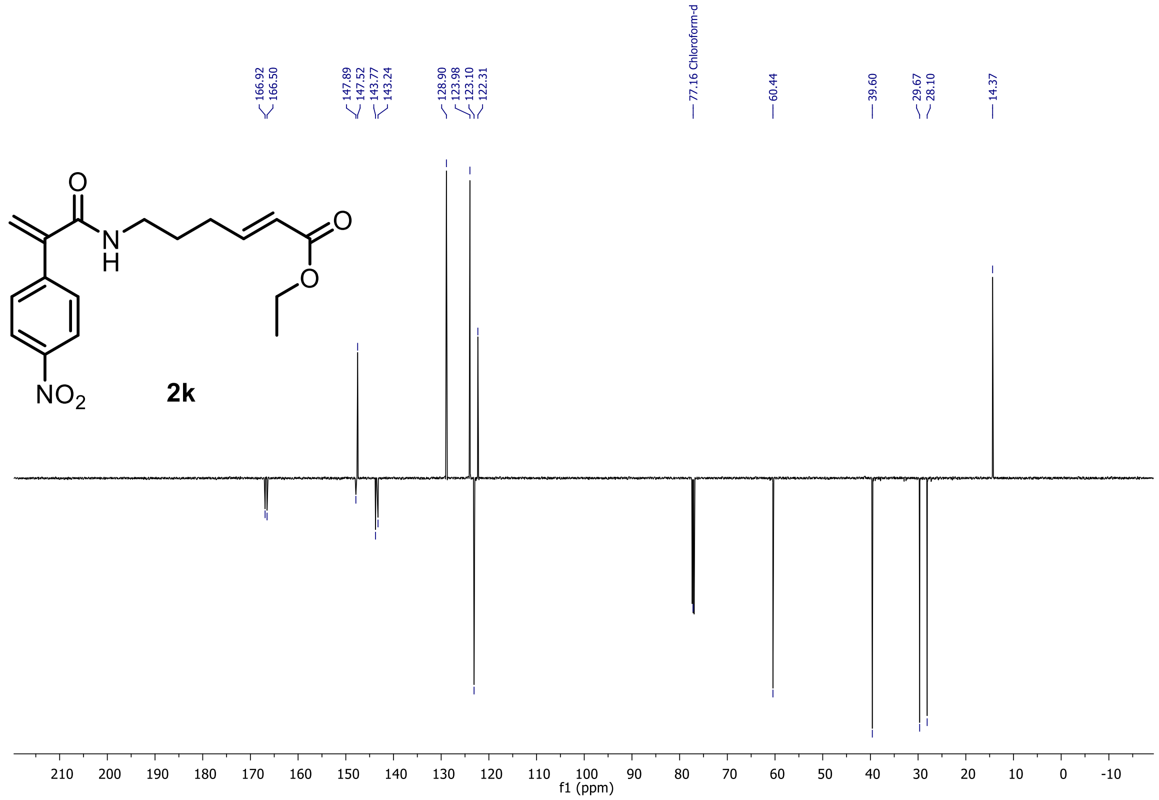

2l: *N*-Methyl-2-(2-nitrophenyl)acrylamide

^{1}H NMR (400 MHz, $CDCl_3$)

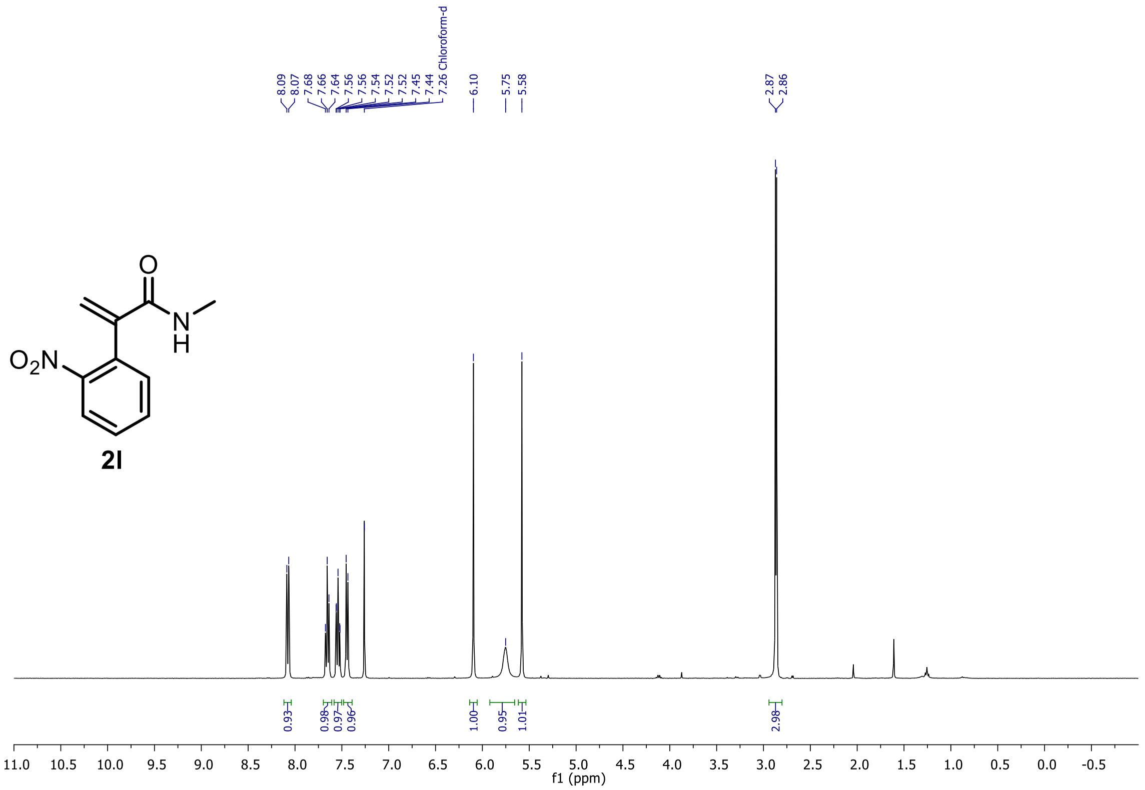

^{13}C NMR (100 MHz, $CDCl_3$)

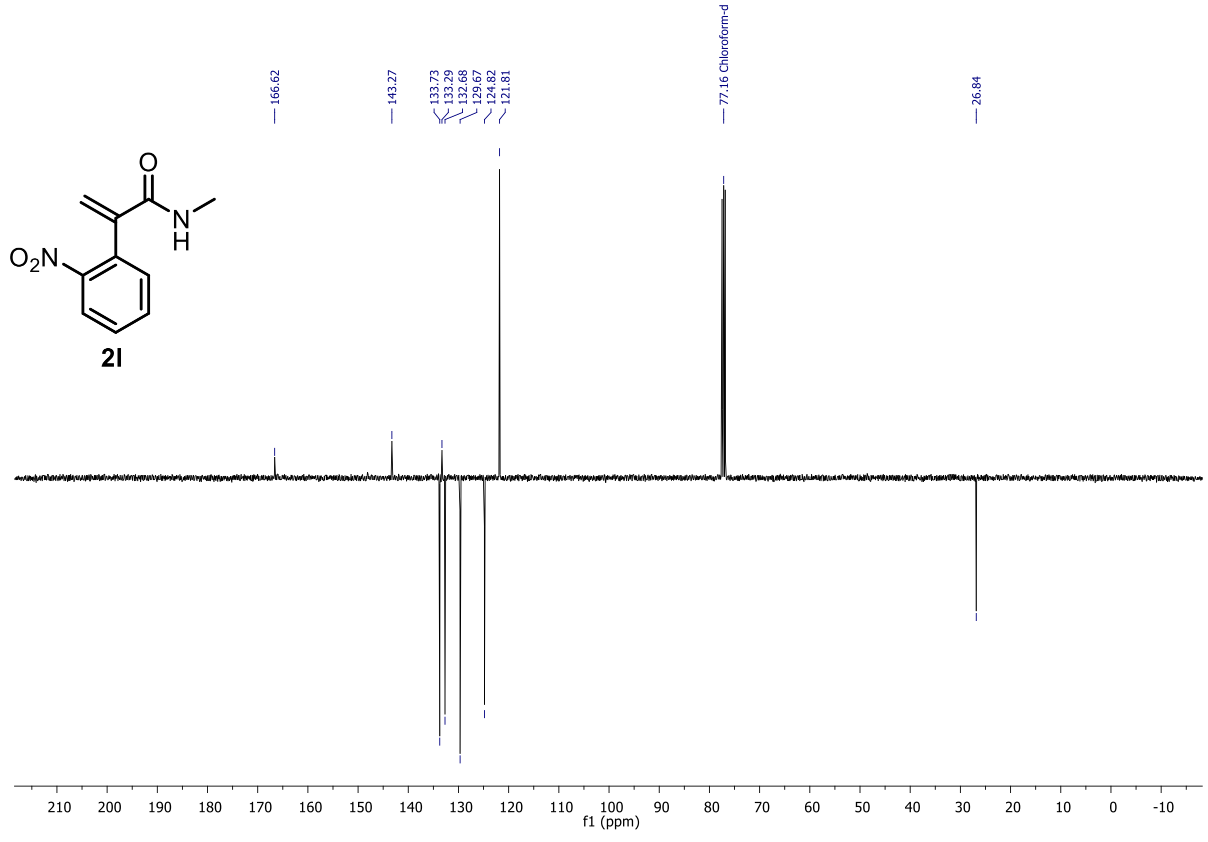

2m: 2-(2-Methoxy-4-nitrophenyl)-*N*-methylacrylamide

^{1}H NMR (400 MHz, $CDCl_3$)

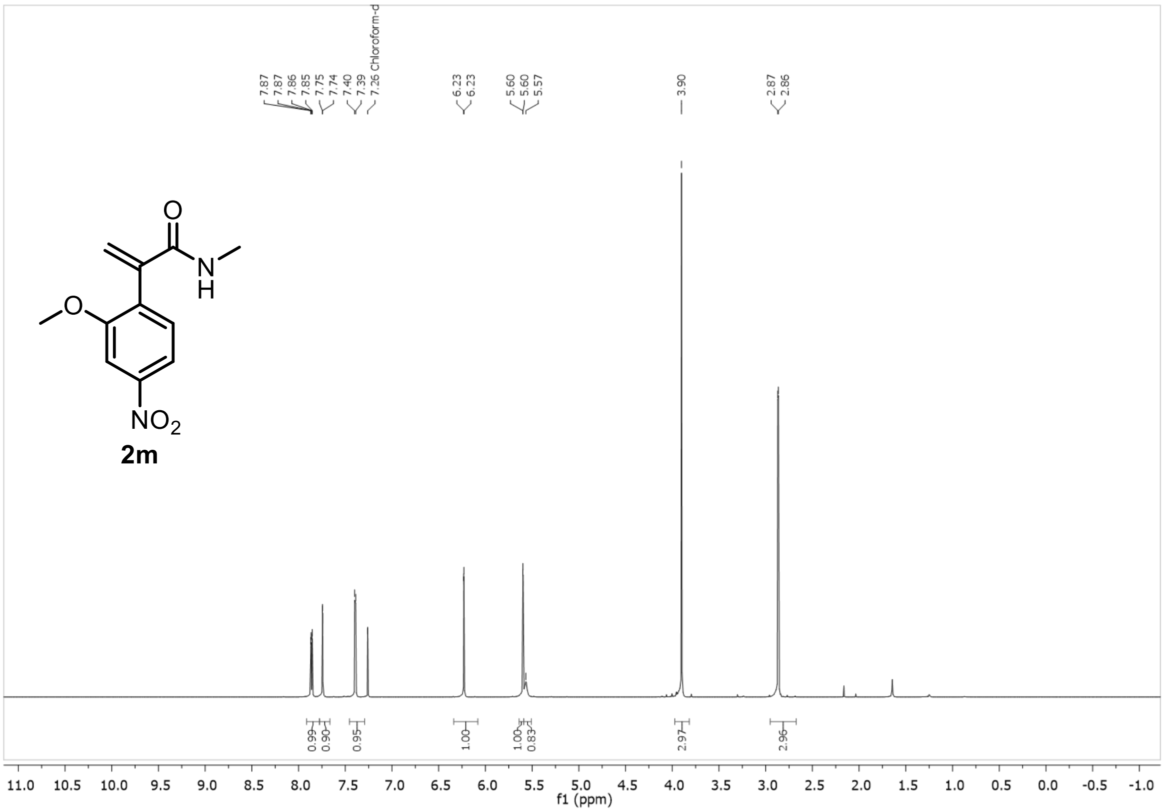

^{13}C NMR (100 MHz, $CDCl_3$)

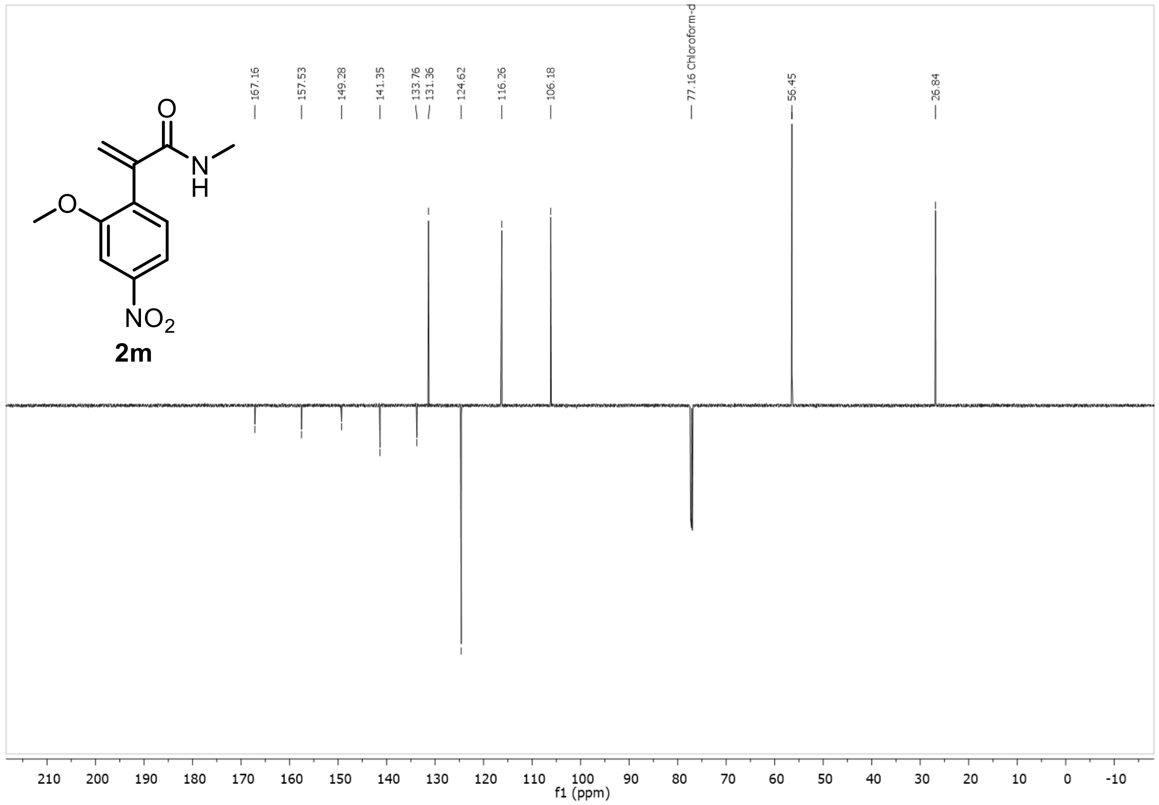

2n: 2-(2,5-Bis(trifluoromethyl)phenyl)-*N*-methylacrylamide

^{1}H NMR (400 MHz, CDCl$_3$)

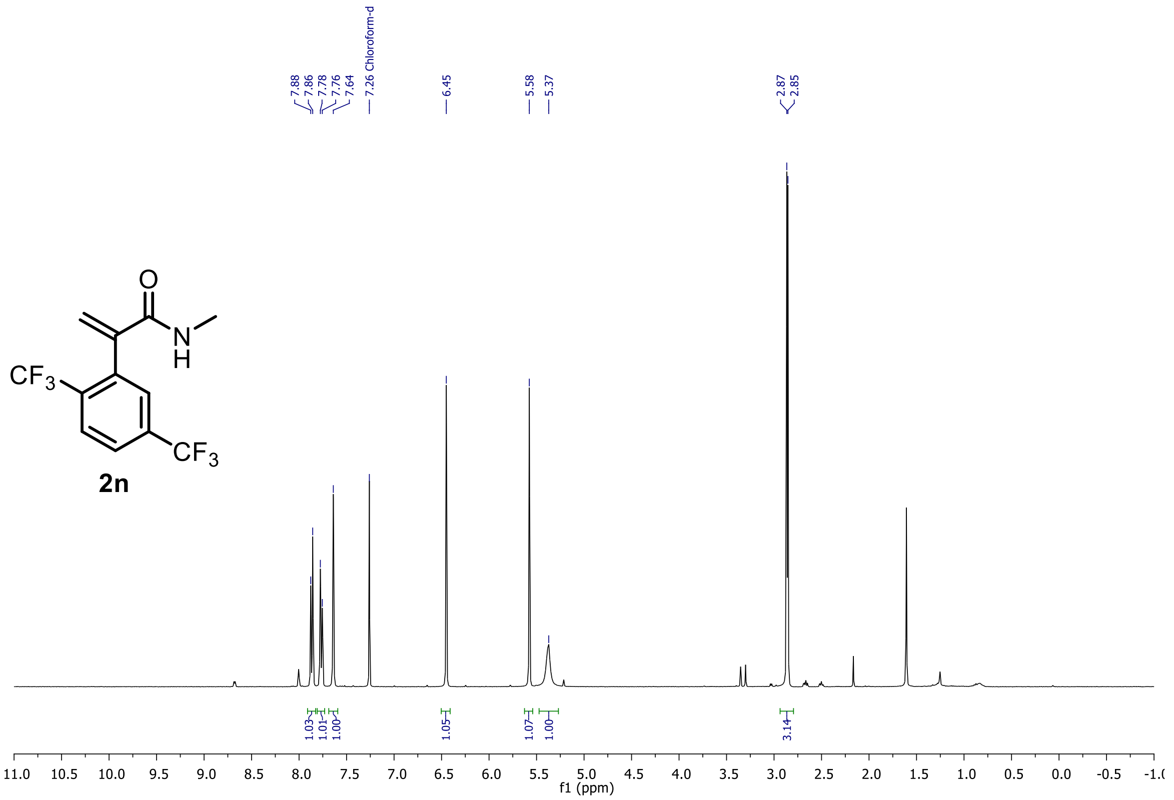

^{13}C NMR (100 MHz, CDCl$_3$)

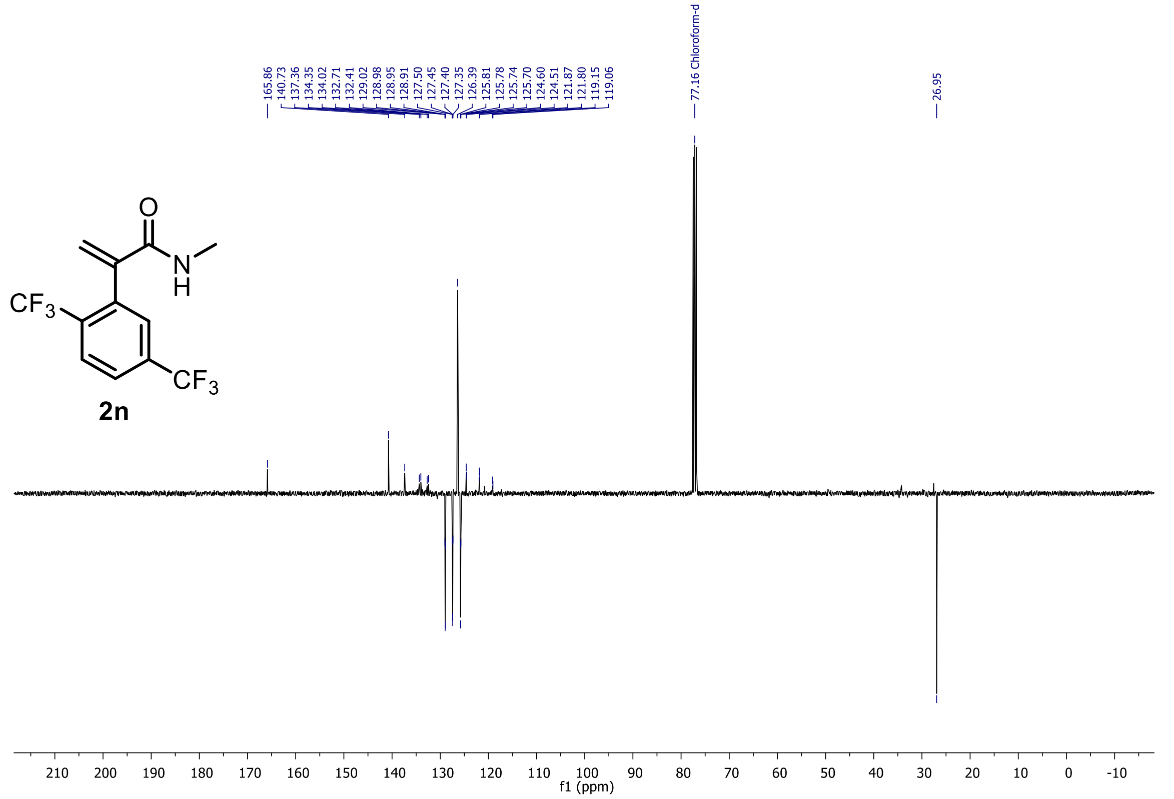

^{19}F NMR (377 MHz, $CDCl_3$)

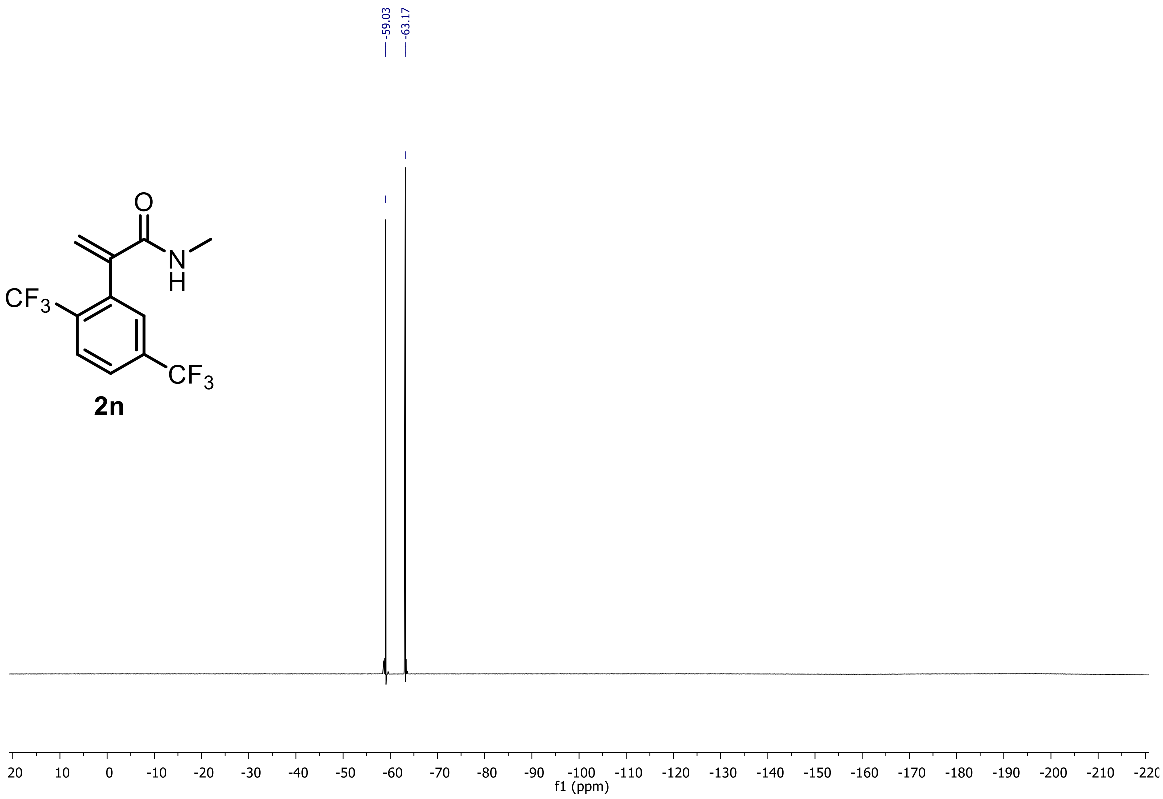

2o: 2-(4-Cyano-2-(trifluoromethyl)phenyl)-*N*-methylacrylamide

^{1}H NMR (700 MHz, $CDCl_3$)

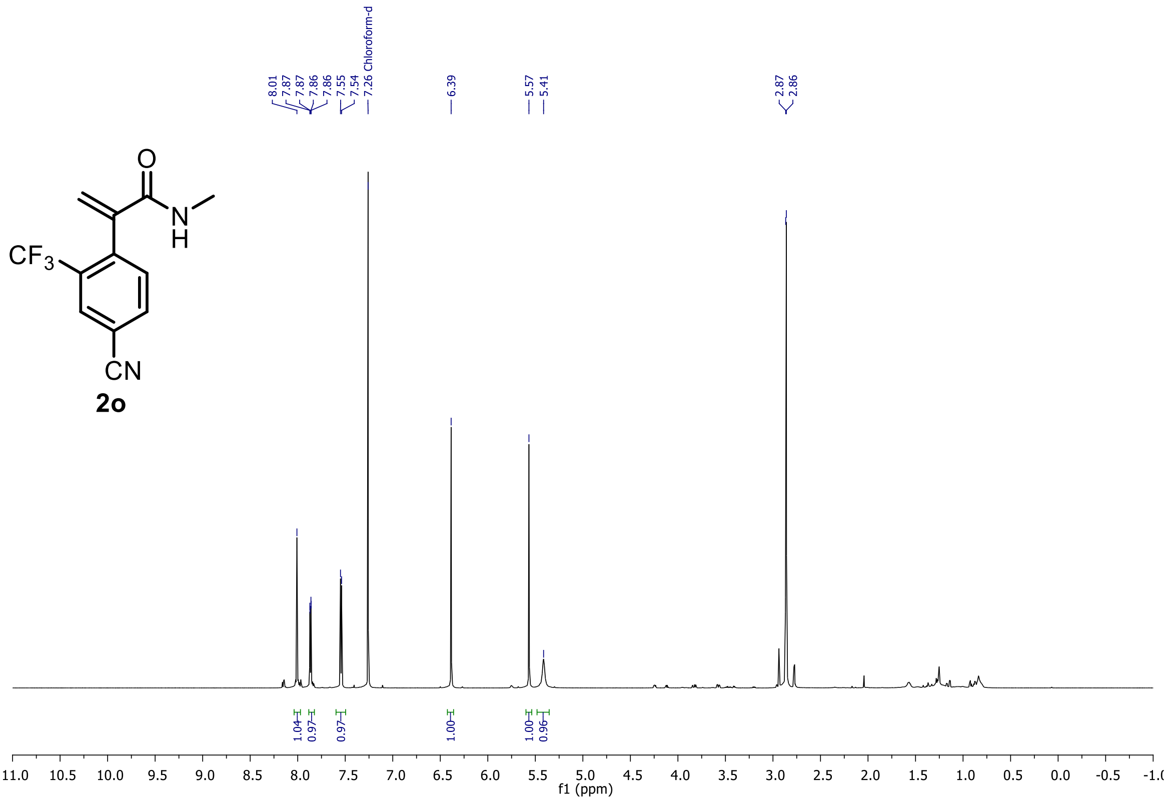

^{13}C NMR (100 MHz, $CDCl_3$)

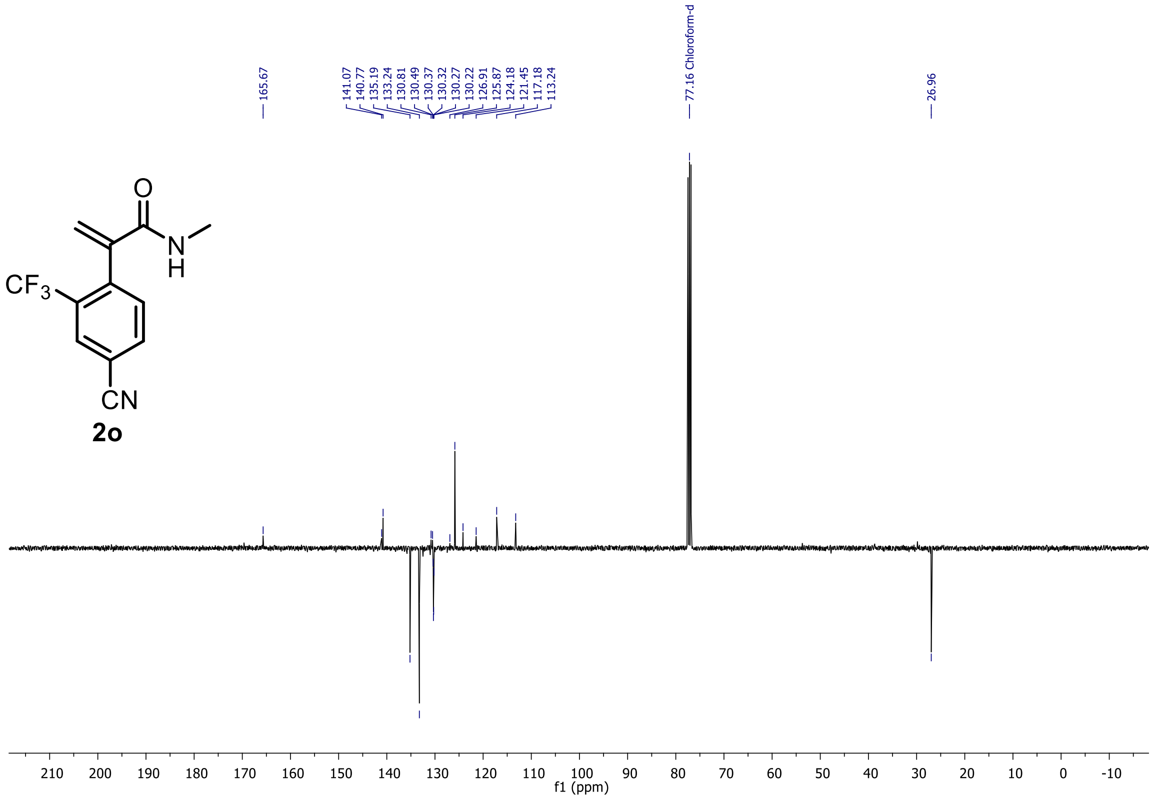

^{19}F NMR (659 MHz, $CDCl_3$)

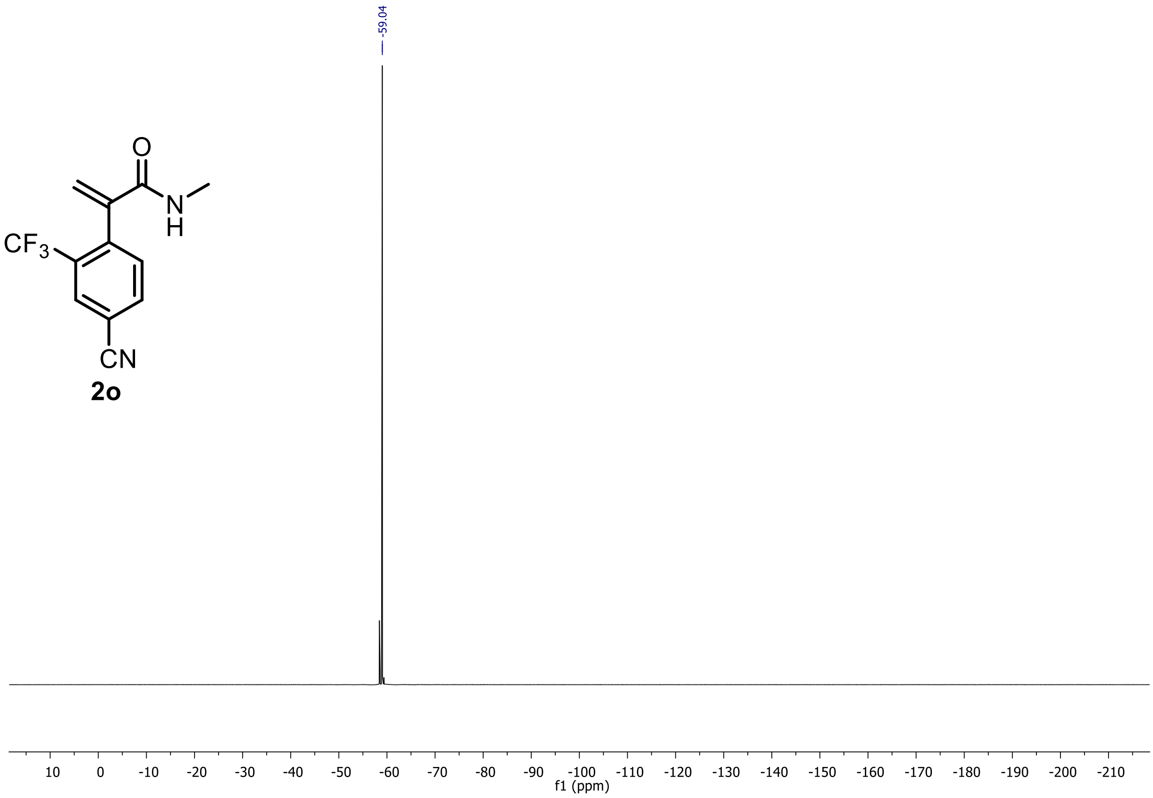

2p: 2-(3-(Methylamino)-3-oxoprop-1-en-2-yl)pyridine 1-oxide

^{1}H NMR (600 MHz, $CDCl_3$)

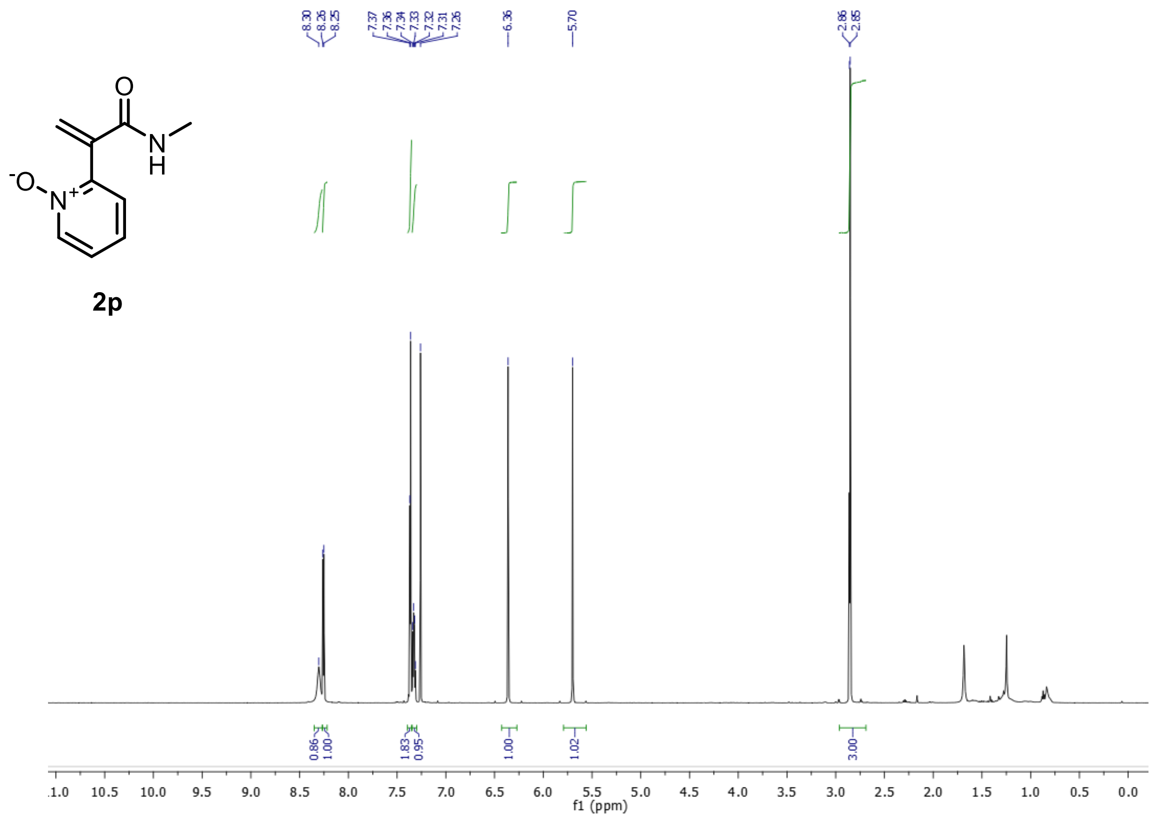

2p

^{13}C NMR (151 MHz, $CDCl_3$)

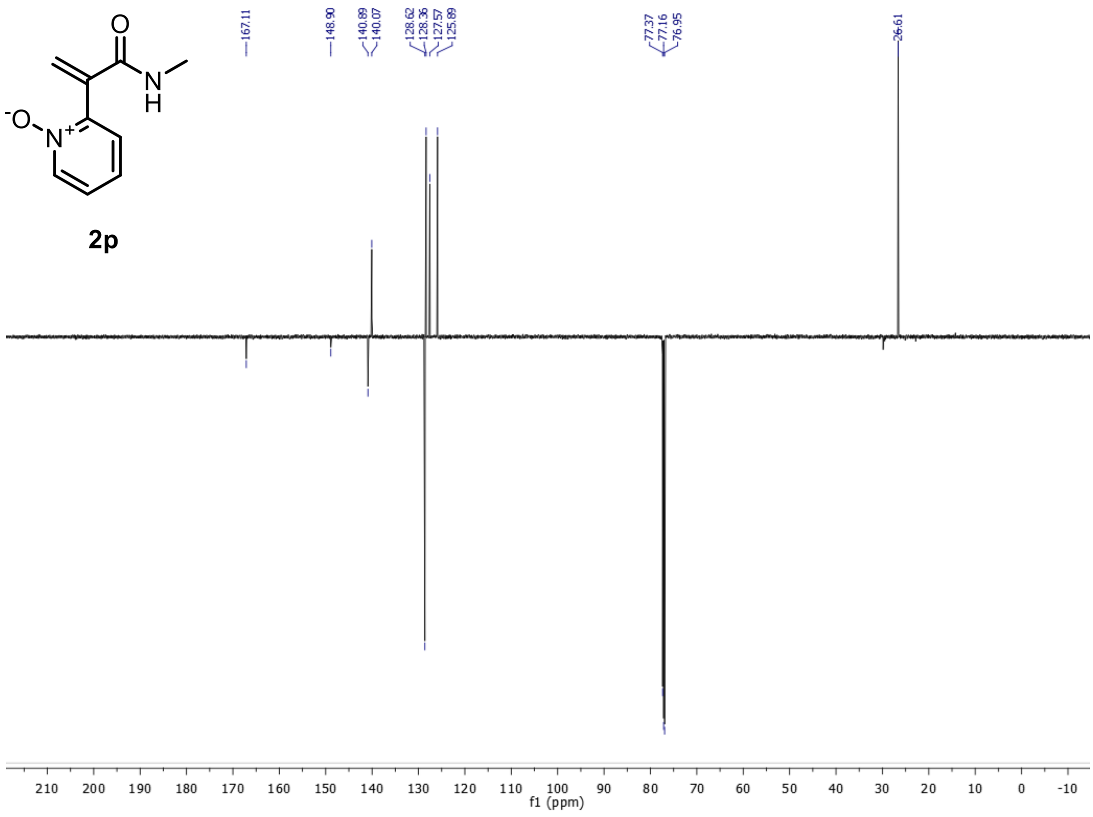

2p

2q: (*Z*)- and (*E*)-*N*-Methyl-2-(4-nitrophenyl)but-2-enamide

^{1}H NMR (400 MHz, $CDCl_3$)

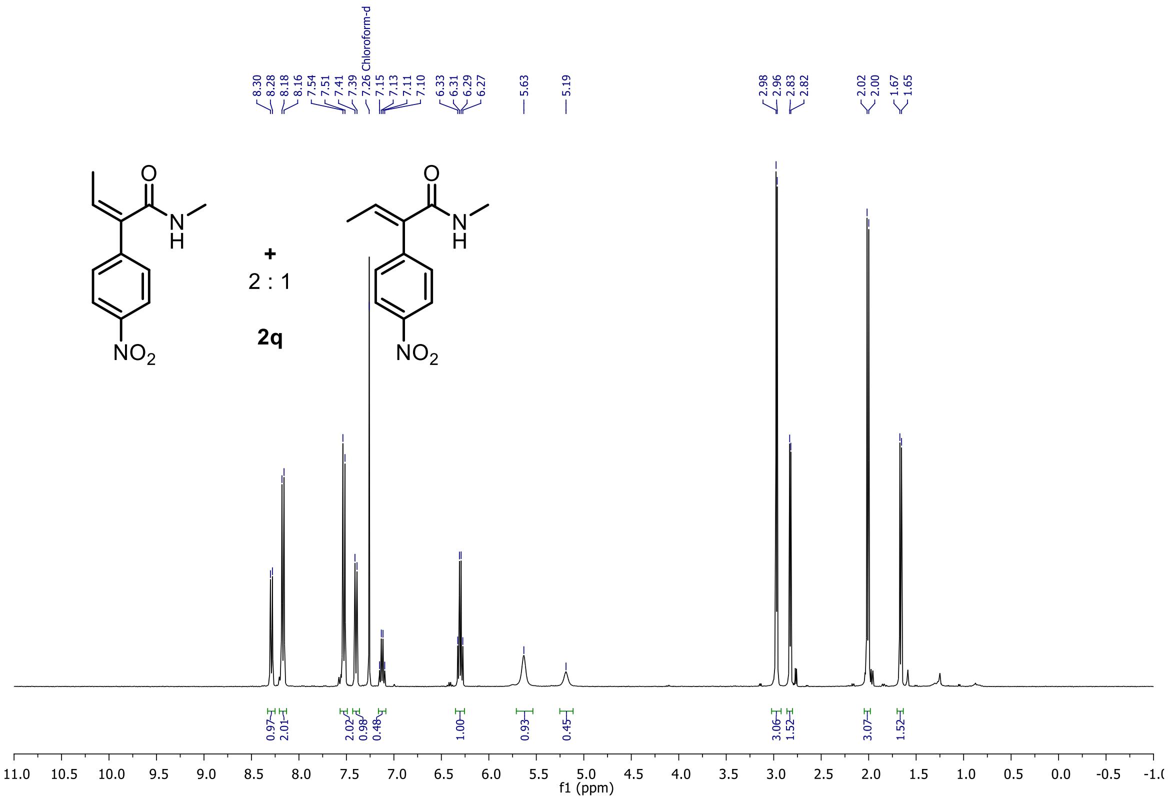

^{13}C NMR (100 MHz, $CDCl_3$)

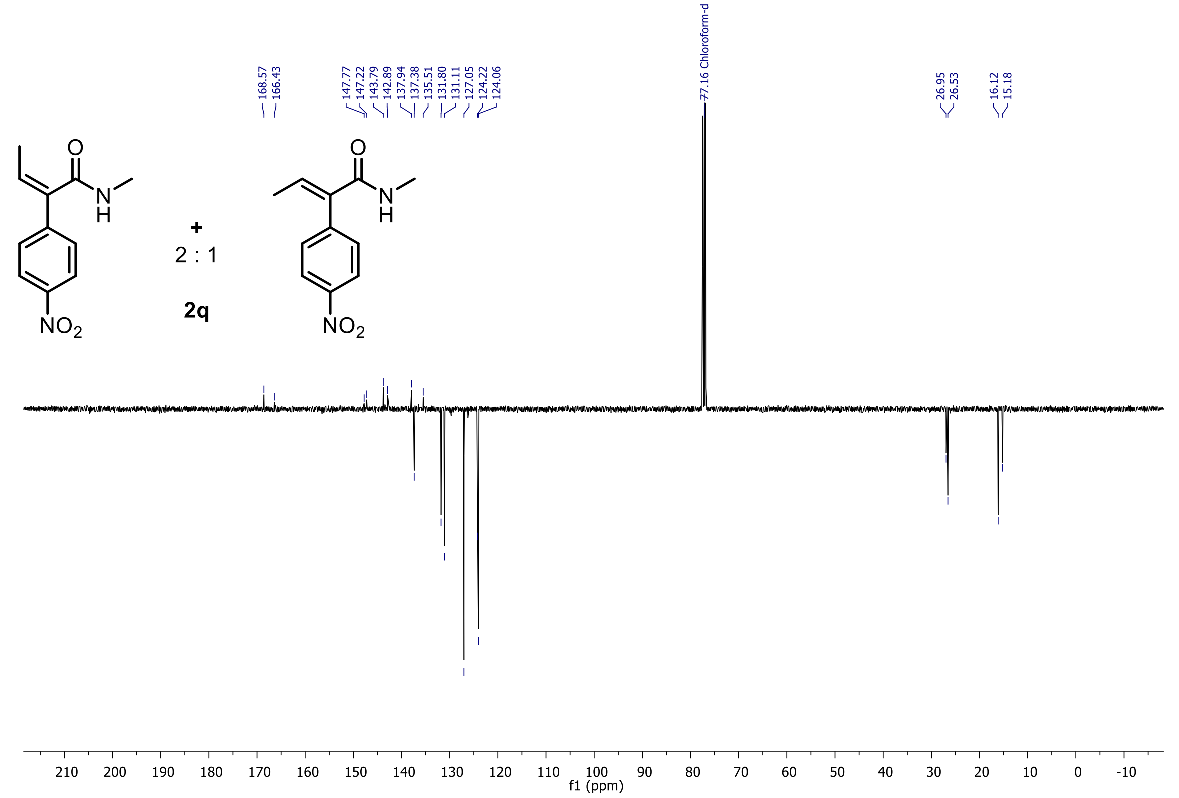

2r: (*E*)-*N*-Methyl-2-(4-nitrophenyl)oct-3-enamide

^{1}H NMR (600 MHz, $CDCl_3$)

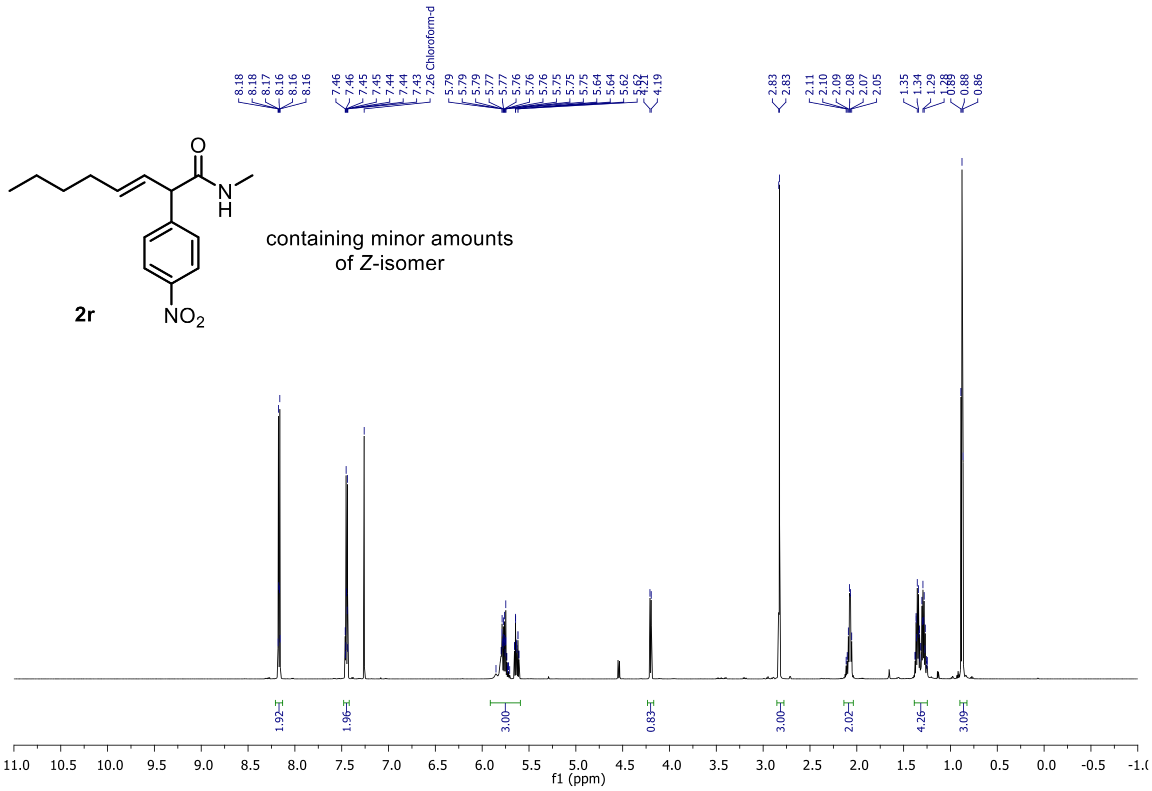

^{13}C NMR (150 MHz, $CDCl_3$)

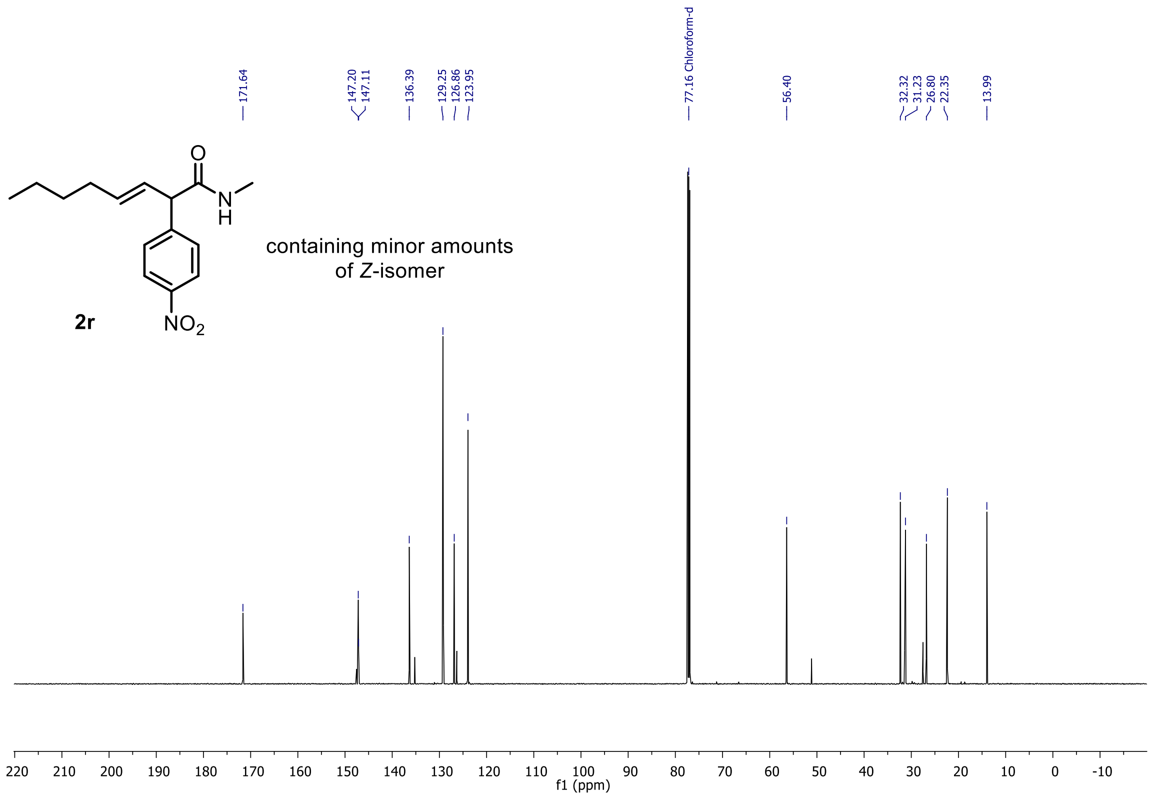

2s: *N*-3-Dimethyl-2-(4-nitrophenyl)but-3-enamide

^{1}H NMR (700 MHz, $CDCl_3$)

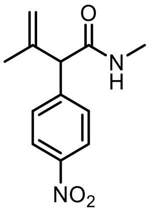

2s

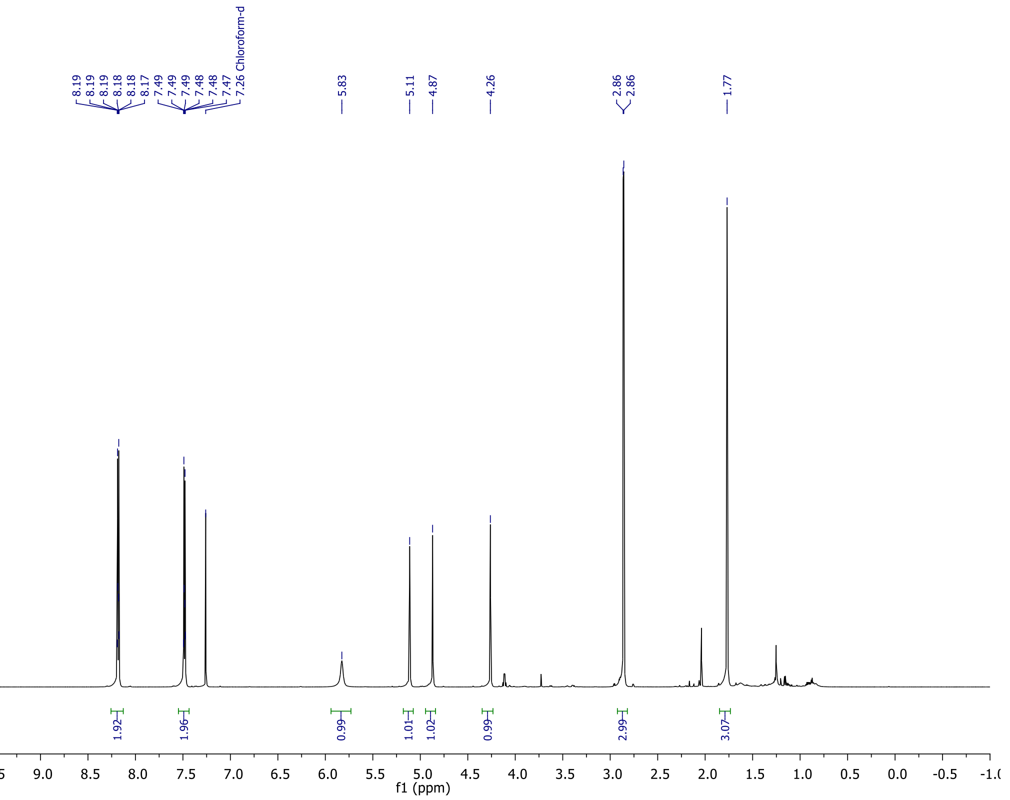

^{13}C NMR (175 MHz, $CDCl_3$)

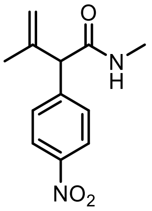

2s

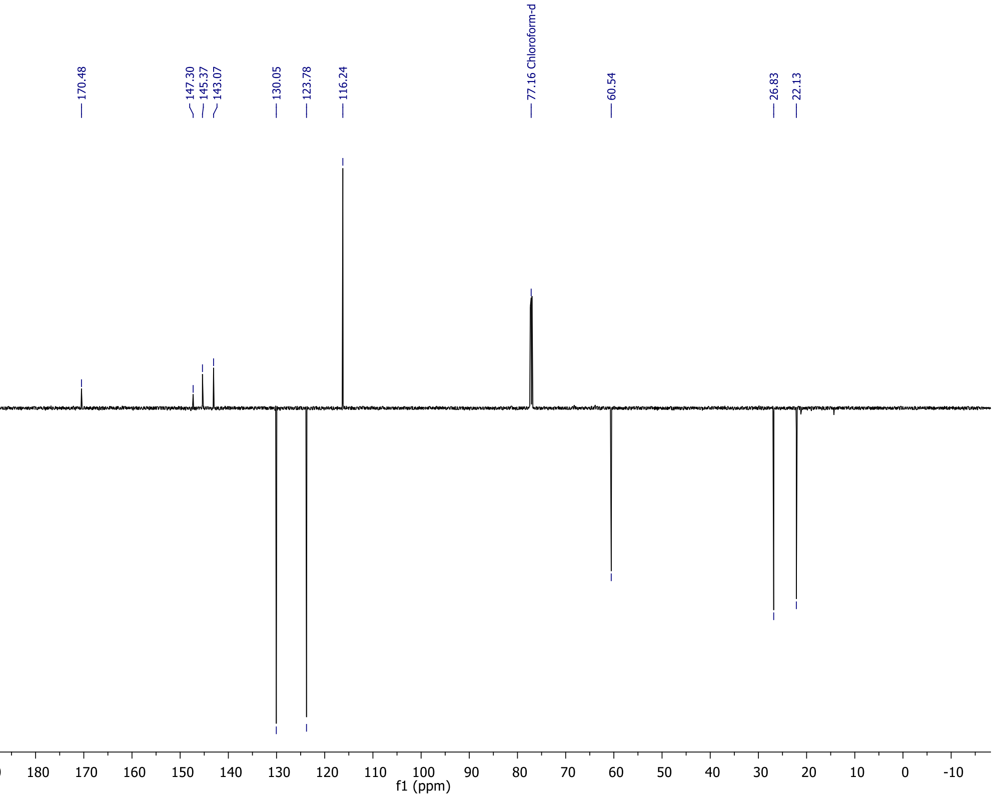

iso-2s: *N*-3-Dimethyl-2-(4-nitrophenyl)but-2-enamide

^{1}H NMR (700 MHz, CDCl$_3$)

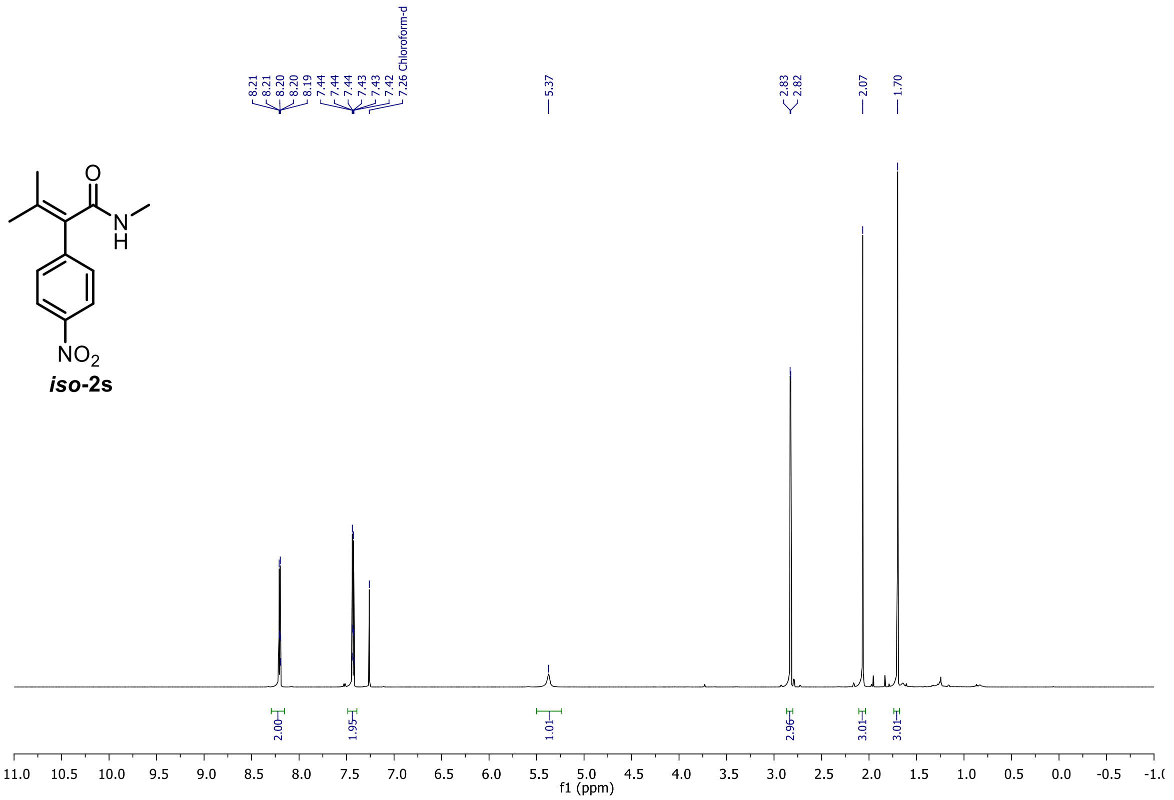

^{13}C NMR (175 MHz, CDCl$_3$)

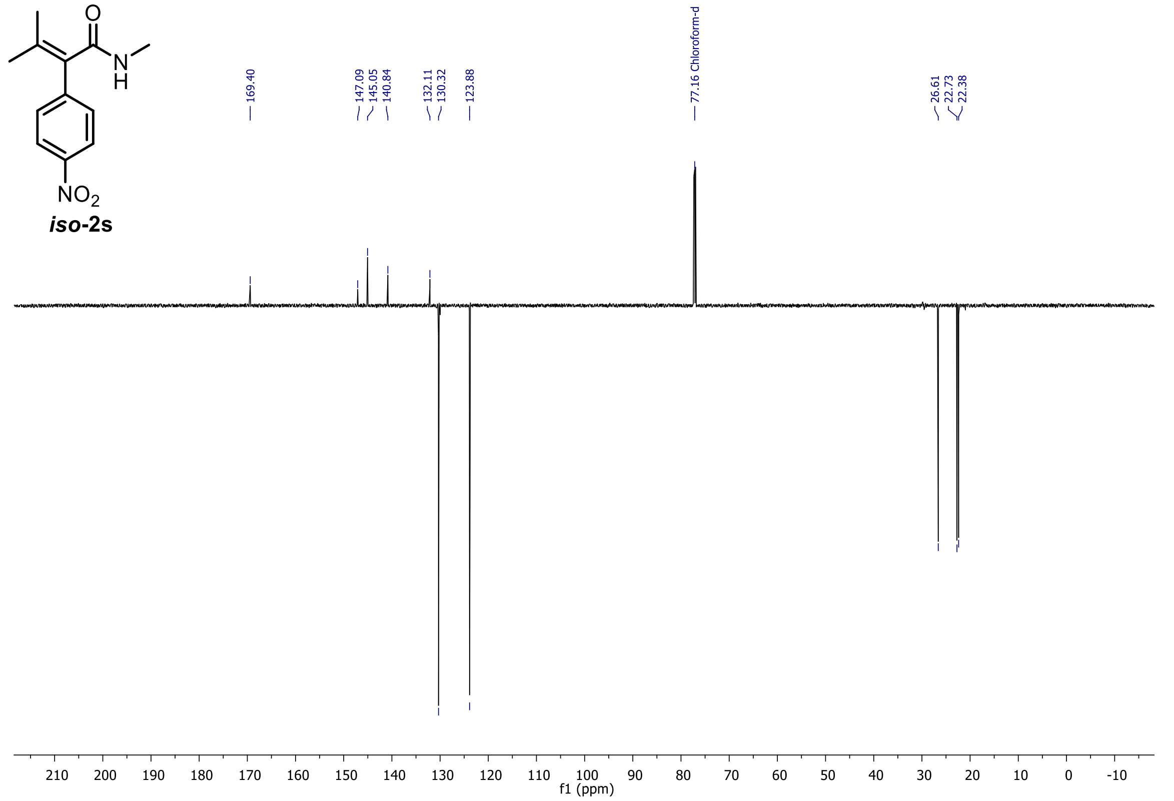

2t: *N*-4-Dimethyl-2-(4-nitrophenyl)pent-3-enamide

^{1}H NMR (400 MHz, $CDCl_3$)

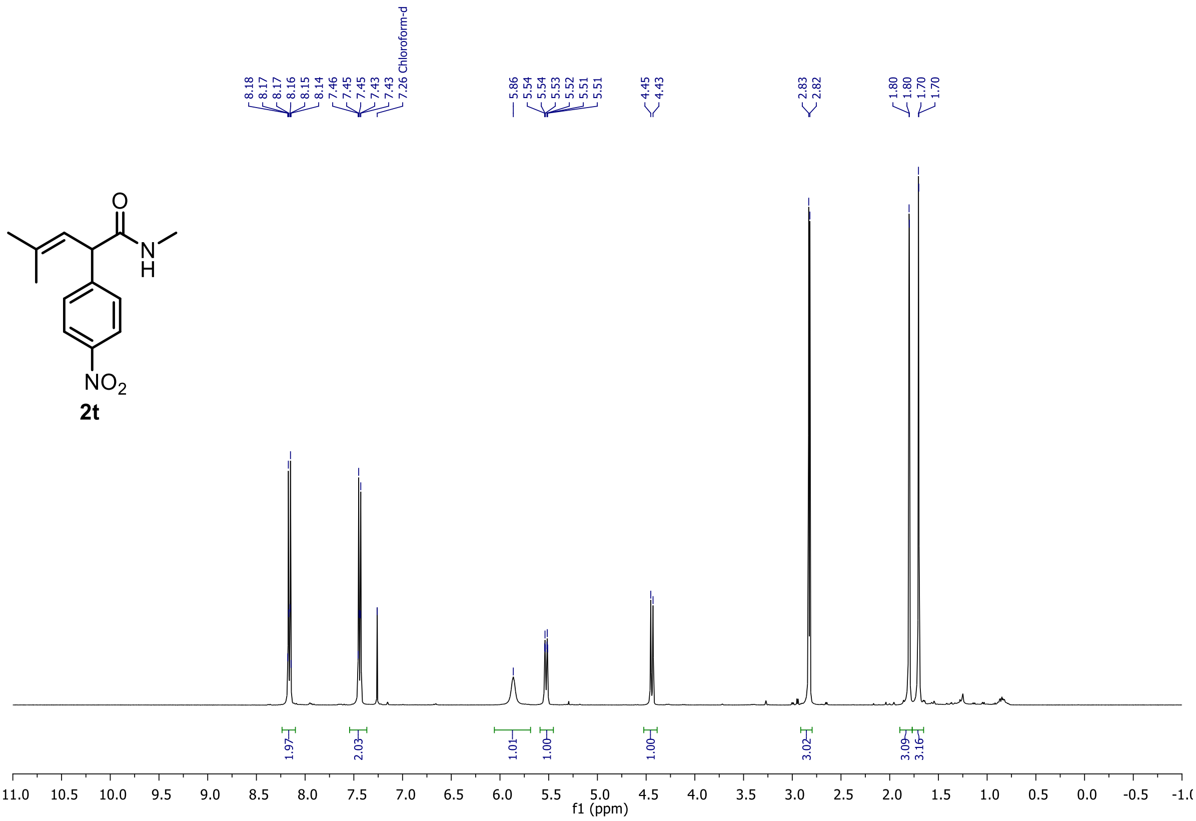

^{13}C NMR (100 MHz, $CDCl_3$)

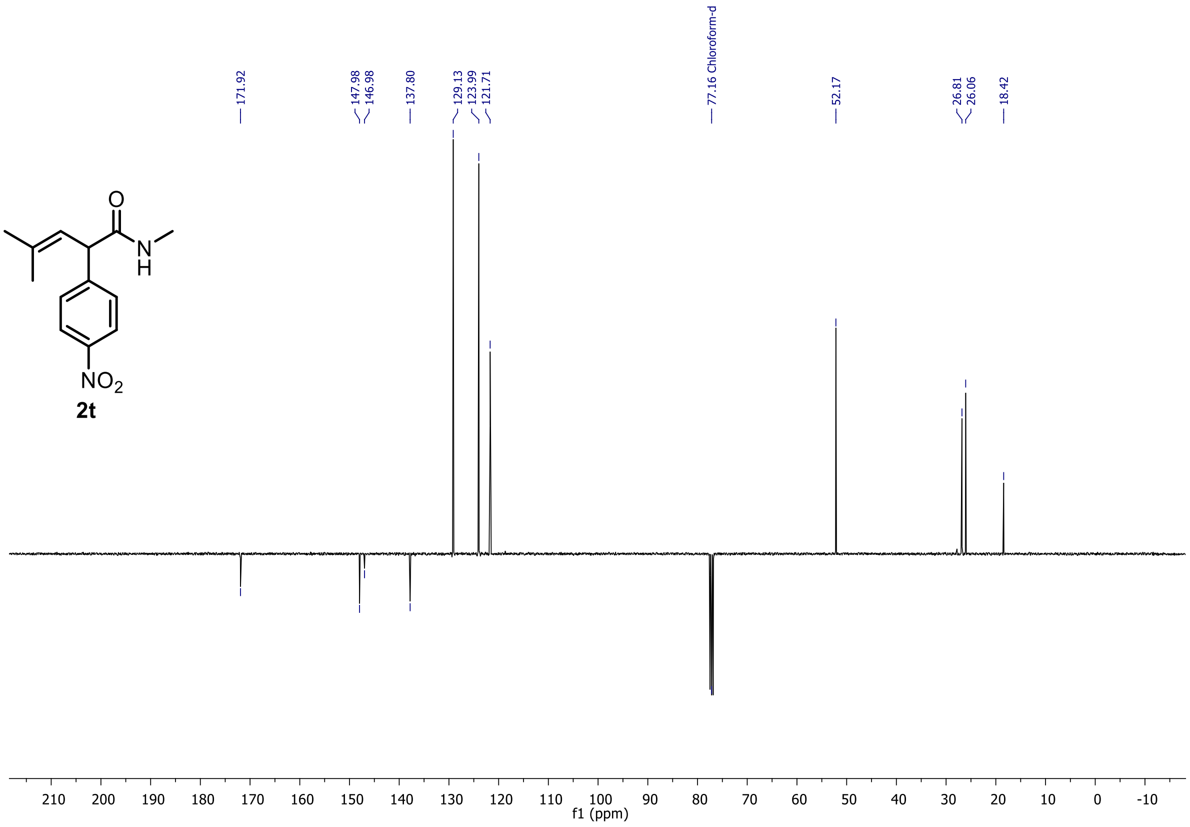

2u: *N*-Methyl-2-(4-nitrophenyl)hexadienamide [isomeric mixture]

^{1}H NMR (400 MHz, CDCl$_3$)

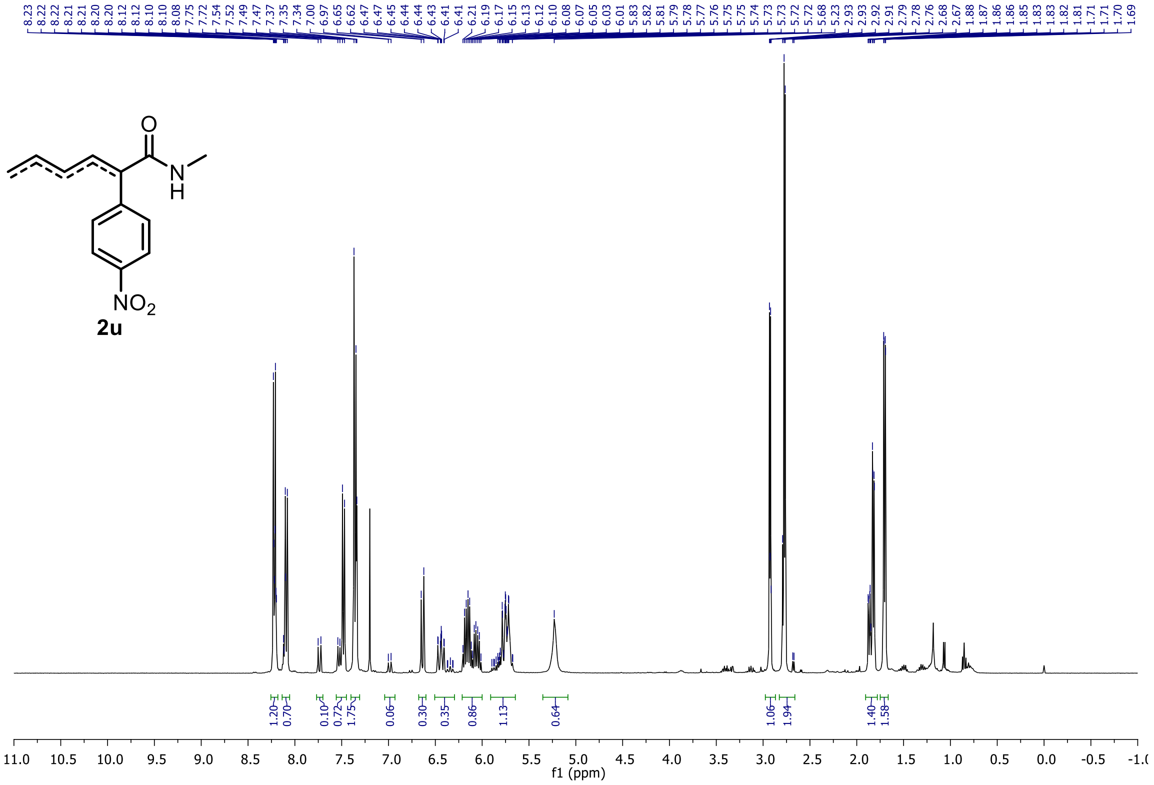

^{13}C NMR (100 MHz, CDCl$_3$)

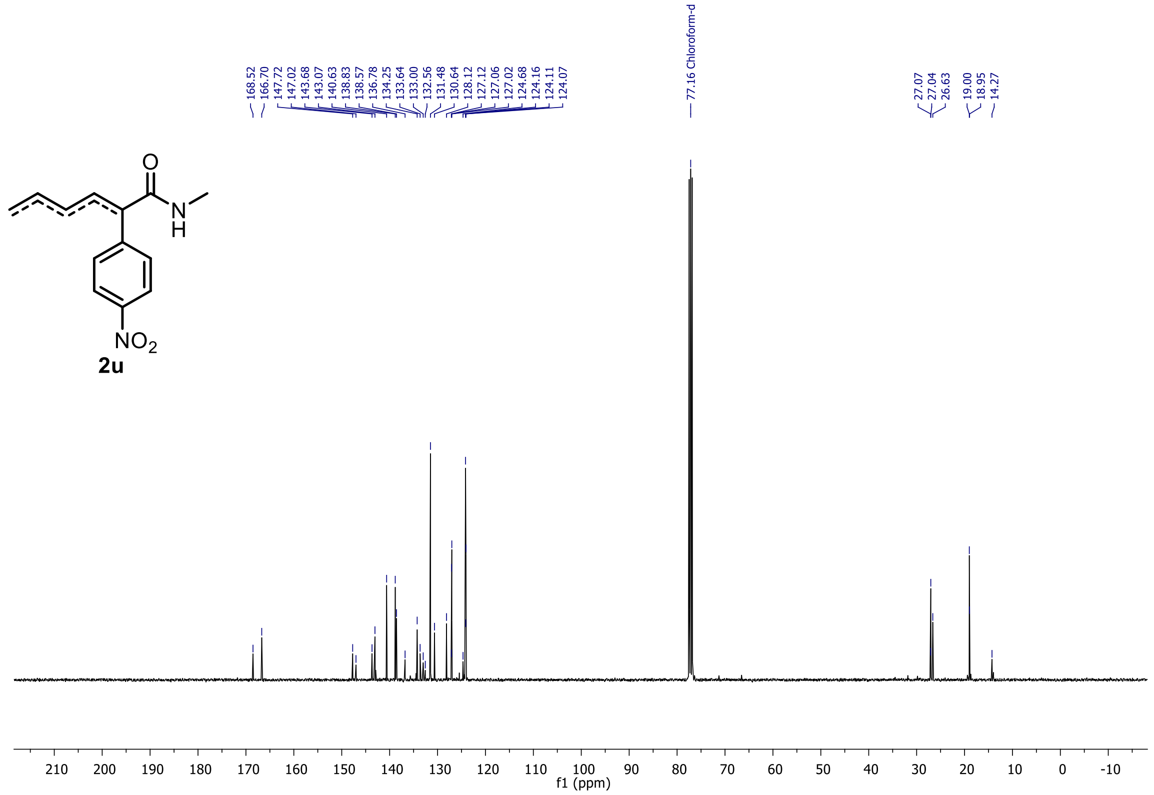

SI4: 2-(4-Nitrophenyl)-*N*-phenylacrylamide

^{1}H NMR (400 MHz, $CDCl_3$)

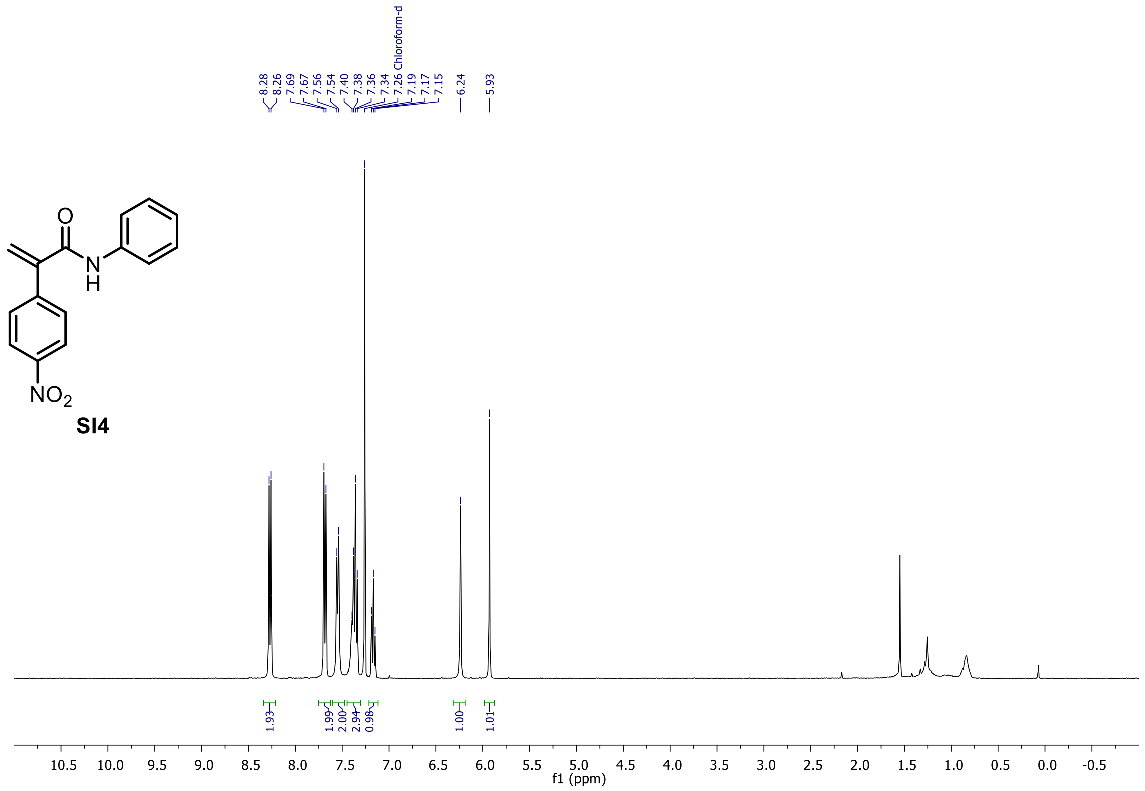

^{13}C NMR (100 MHz, $CDCl_3$)

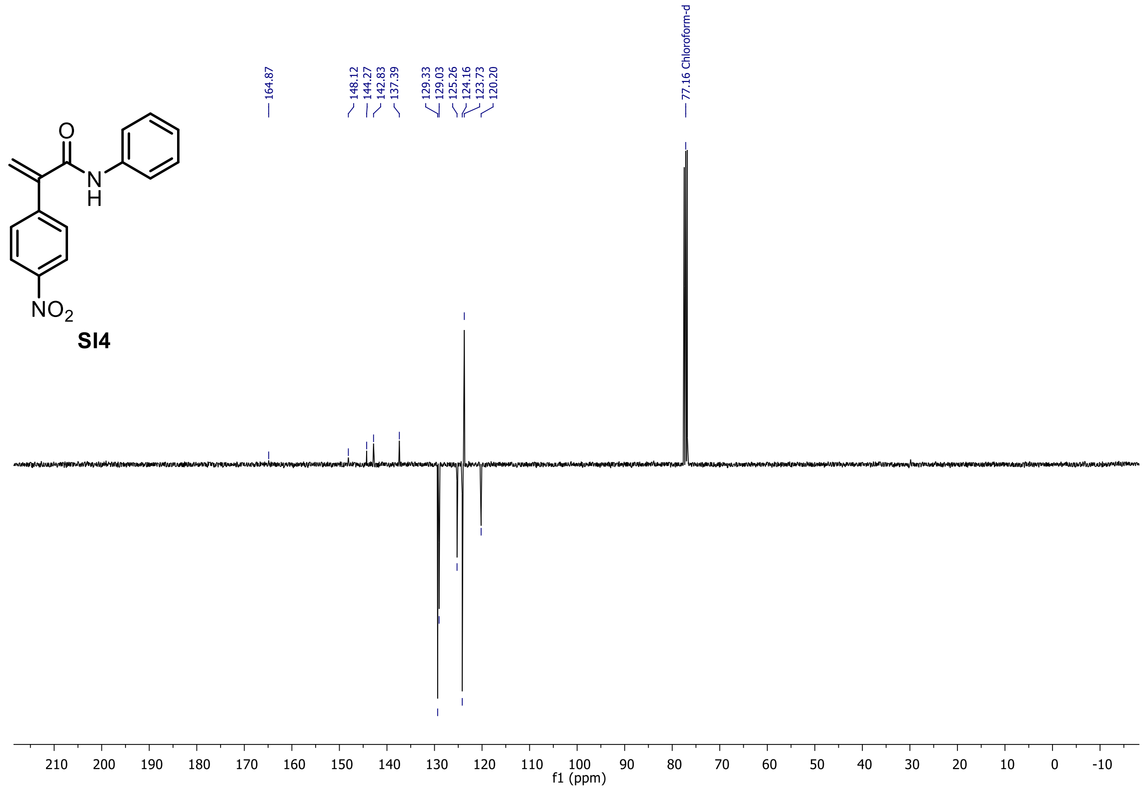

SI5: 2-(4-Cyanophenyl)-*N*-methylacrylamide

^{1}H NMR (400 MHz, $CDCl_3$)

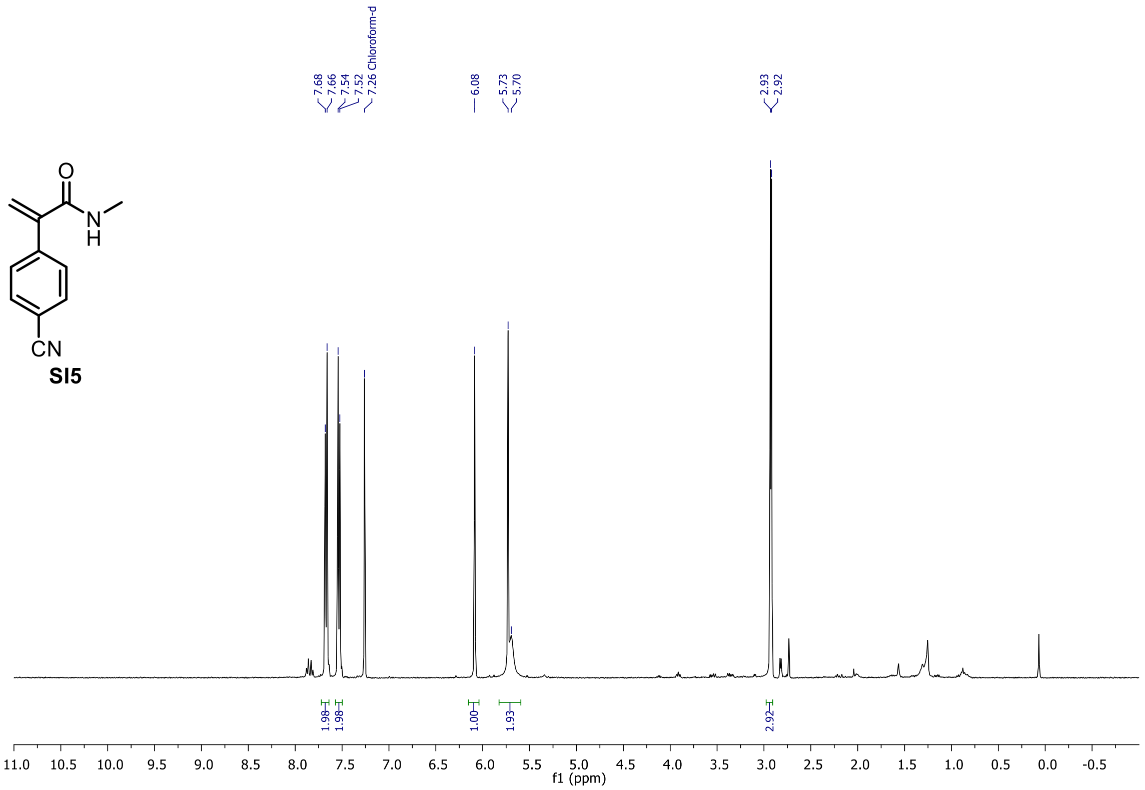

^{13}C NMR (100 MHz, $CDCl_3$)

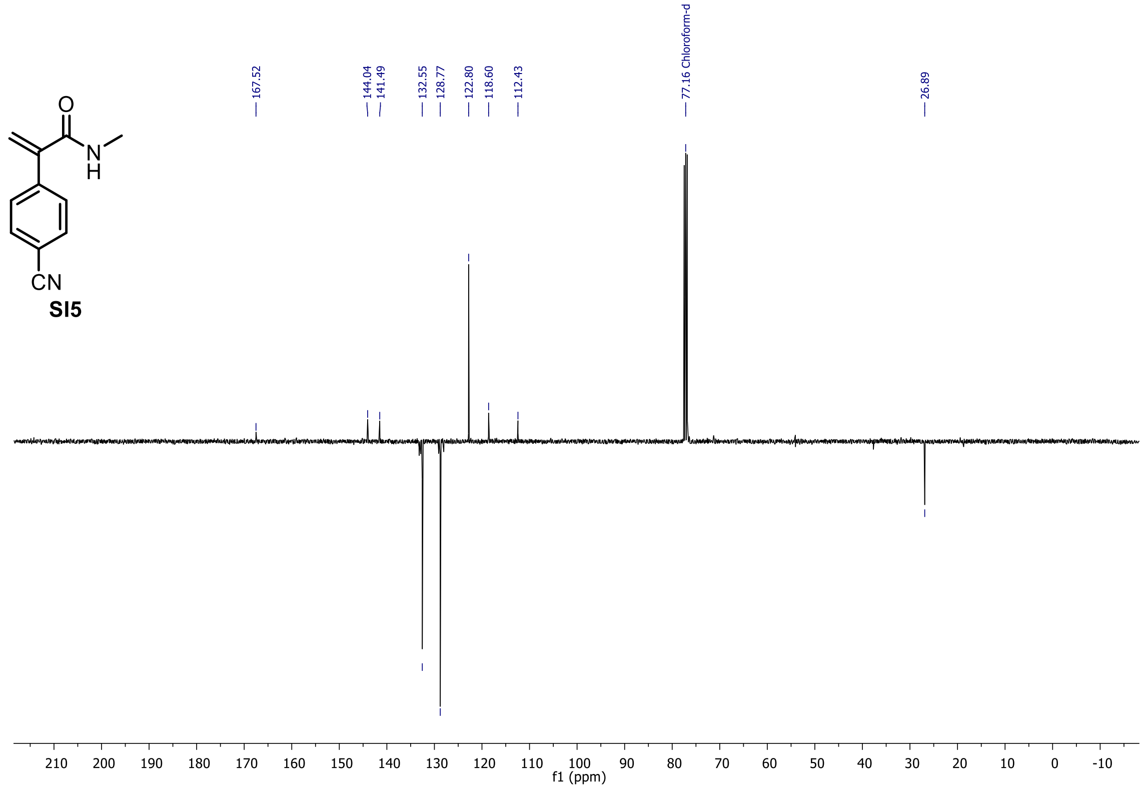

1-rc: *N*-Methyl-3-((*N*-methyl-4-nitrophenyl)sulfonamido)-*N*-((4-nitrophenyl)sulfonyl)-propanamide

^{1}H NMR (400 MHz, $CDCl_3$)

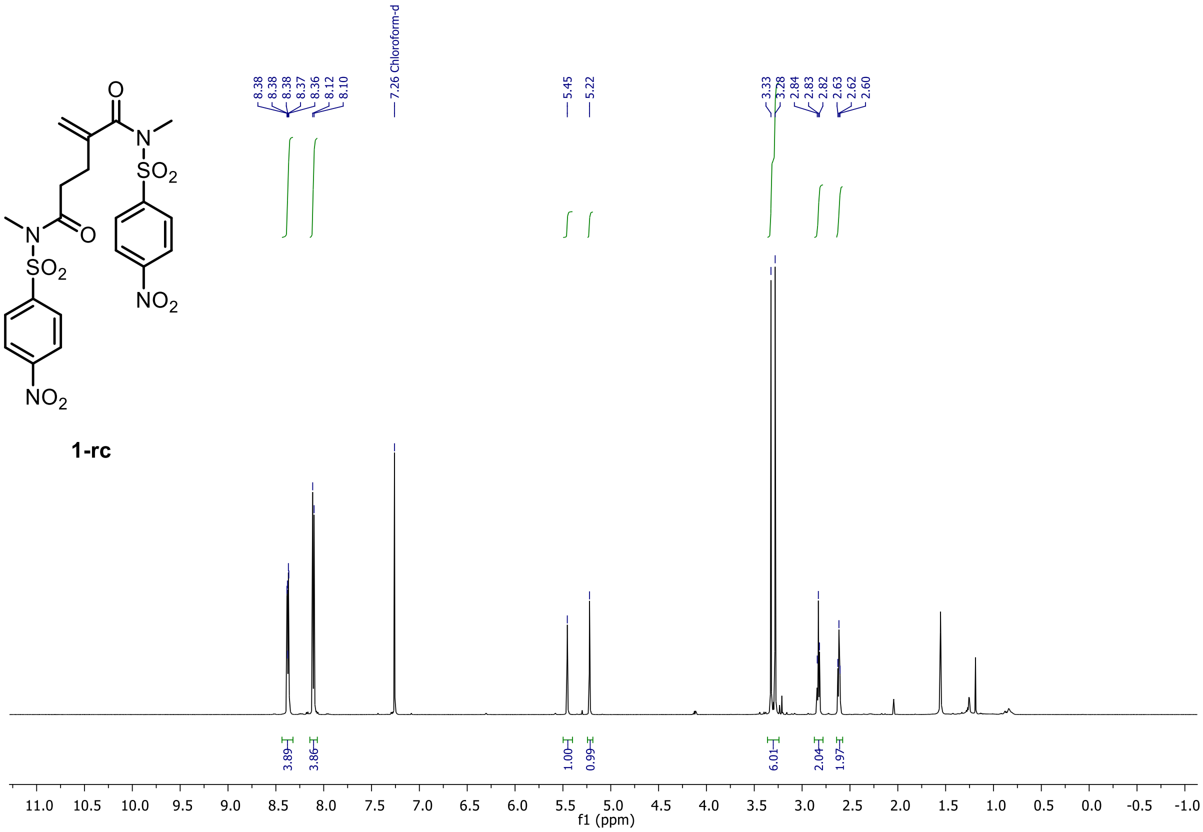

^{13}C NMR (100 MHz, $CDCl_3$)

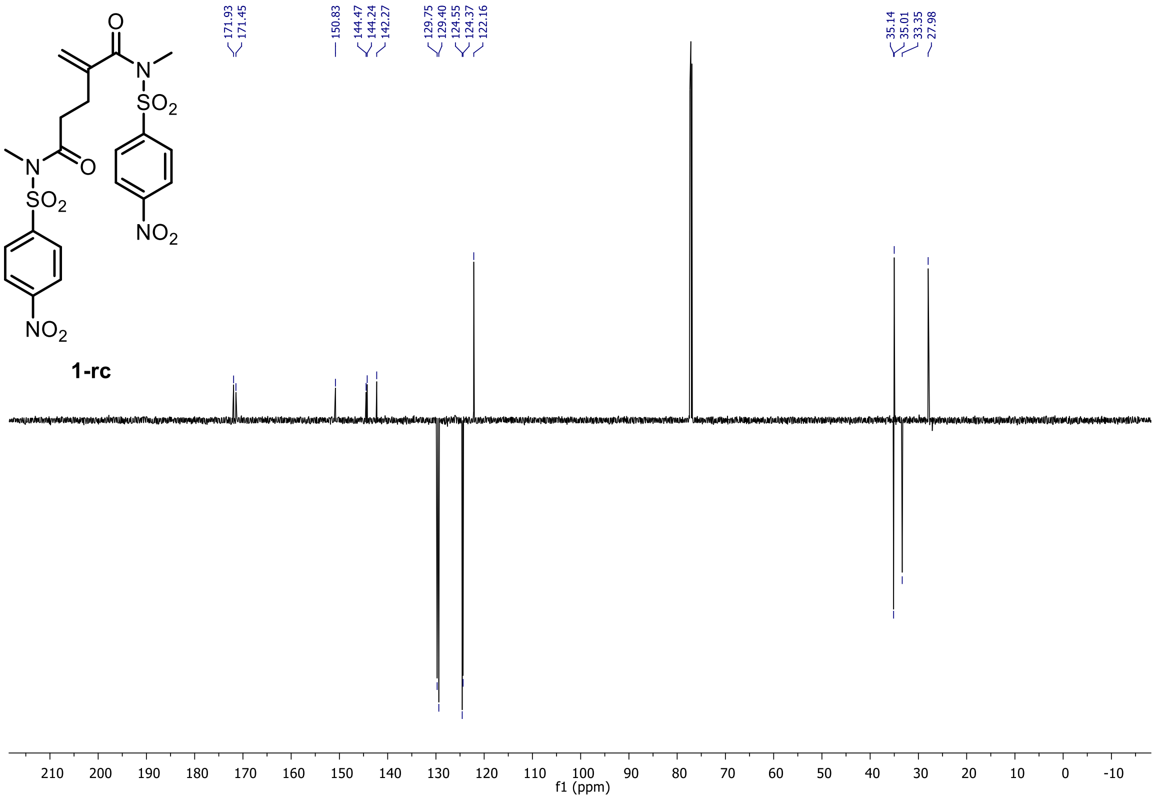

1-sa: *N*-Methyl-3-((*N*-methyl-4-nitrophenyl)sulfonamido)-*N*-((4-nitrophenyl)sulfonyl)-propanamide

^{1}H NMR (400 MHz, $CDCl_3$)

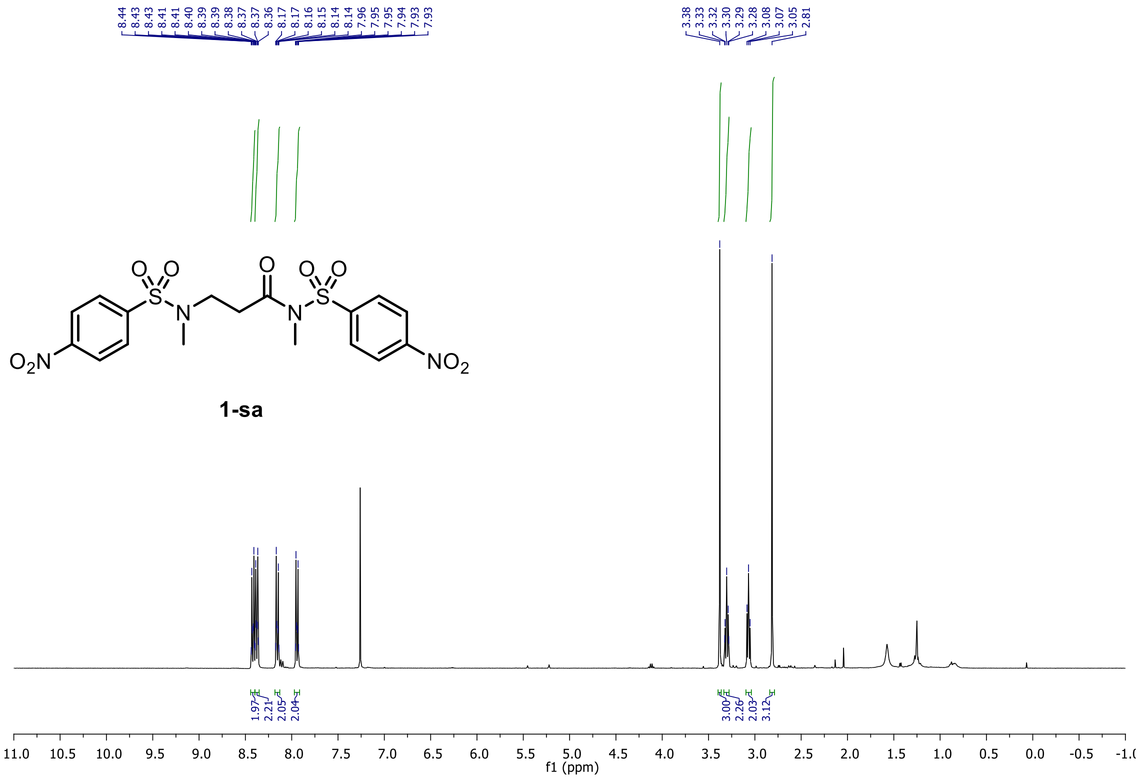

^{13}C NMR (100 MHz, $CDCl_3$)

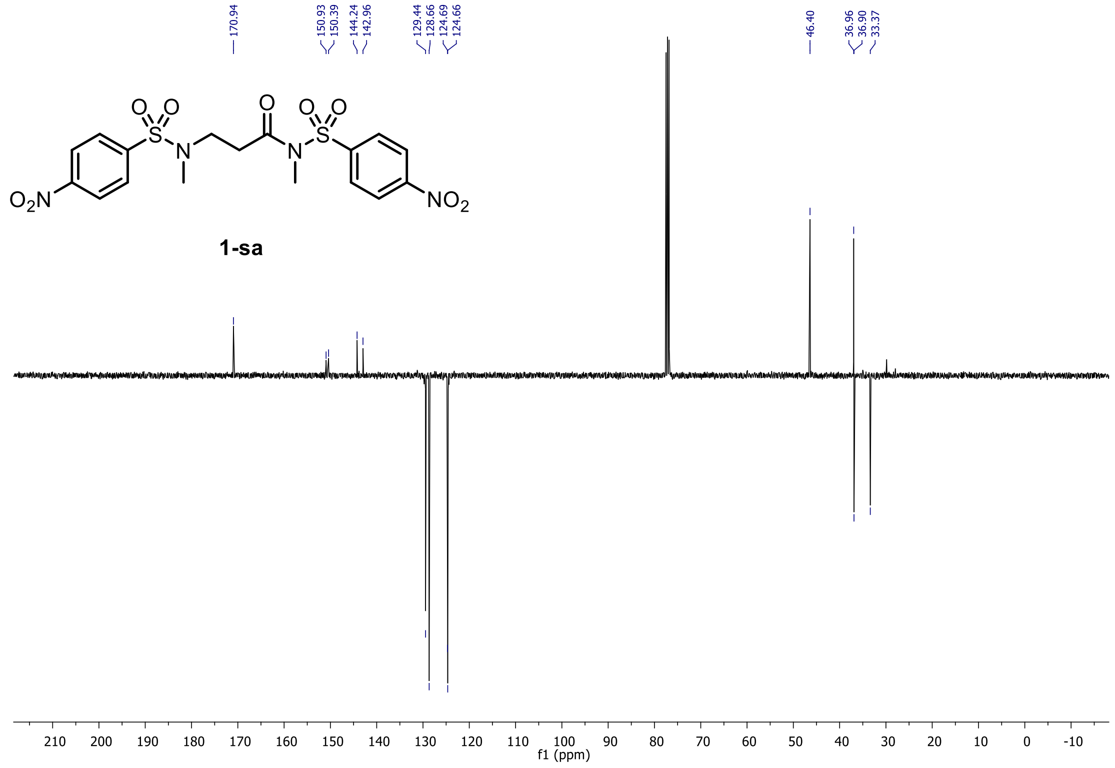

3: 1-Methyl-3-(4-nitrophenyl)-5,6-diphenylpyridin-2(1*H*)-one

^{1}H NMR (400 MHz, $CDCl_3$)

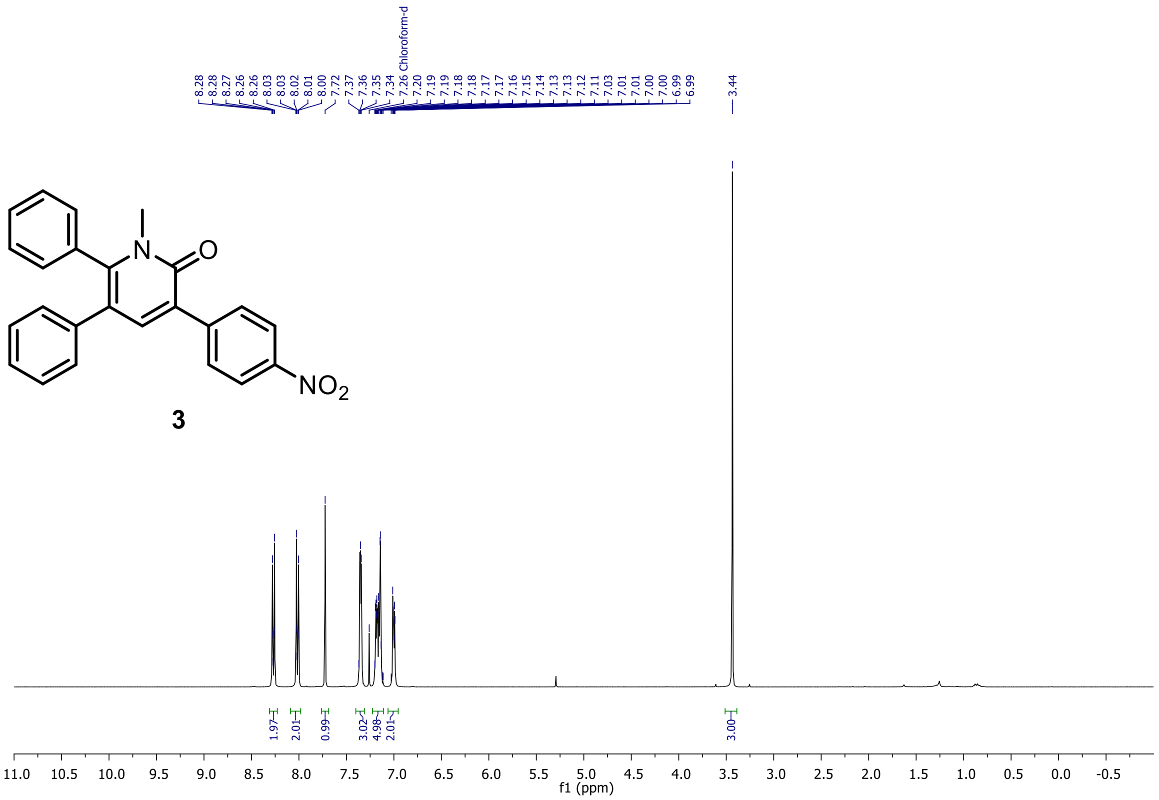

^{13}C NMR (100 MHz, $CDCl_3$)

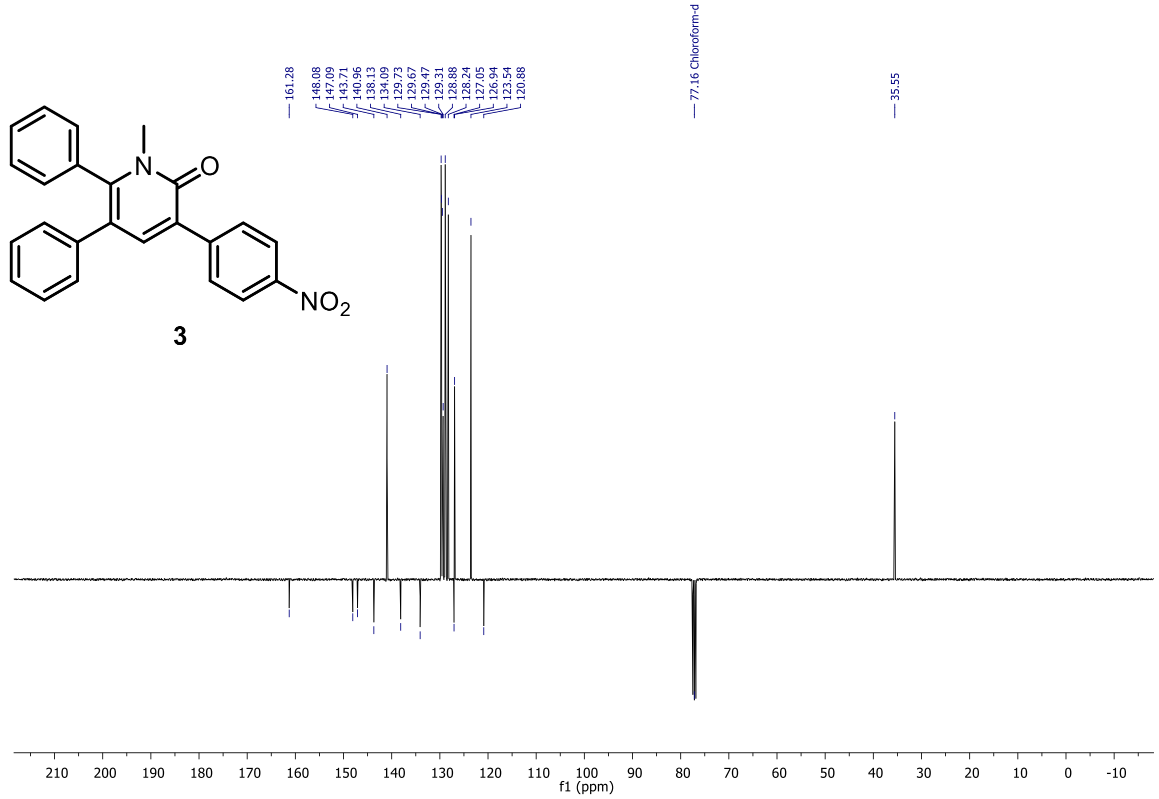

4: 1-(2-Methoxypyrrolidin-1-yl)-2-(4-nitrophenyl)prop-2-en-1-one

^{1}H NMR (600 MHz, CDCl$_3$)

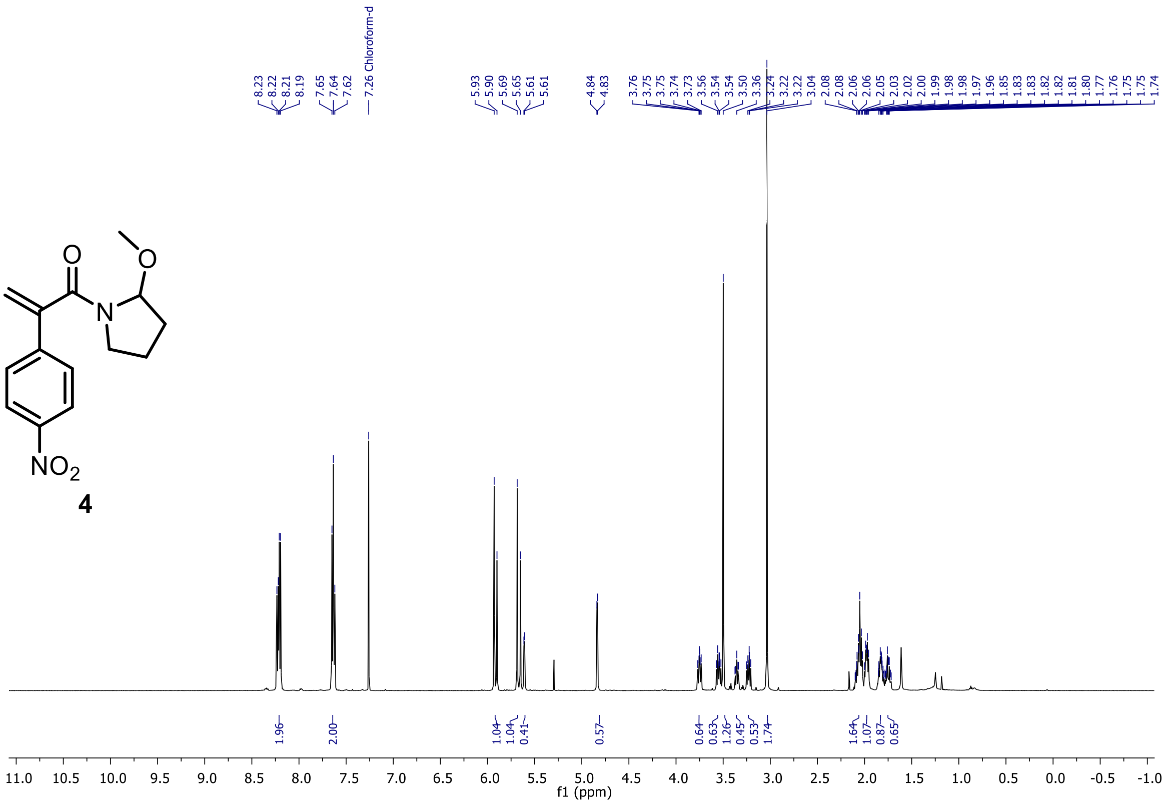

^{13}C NMR (151 MHz, CDCl$_3$)

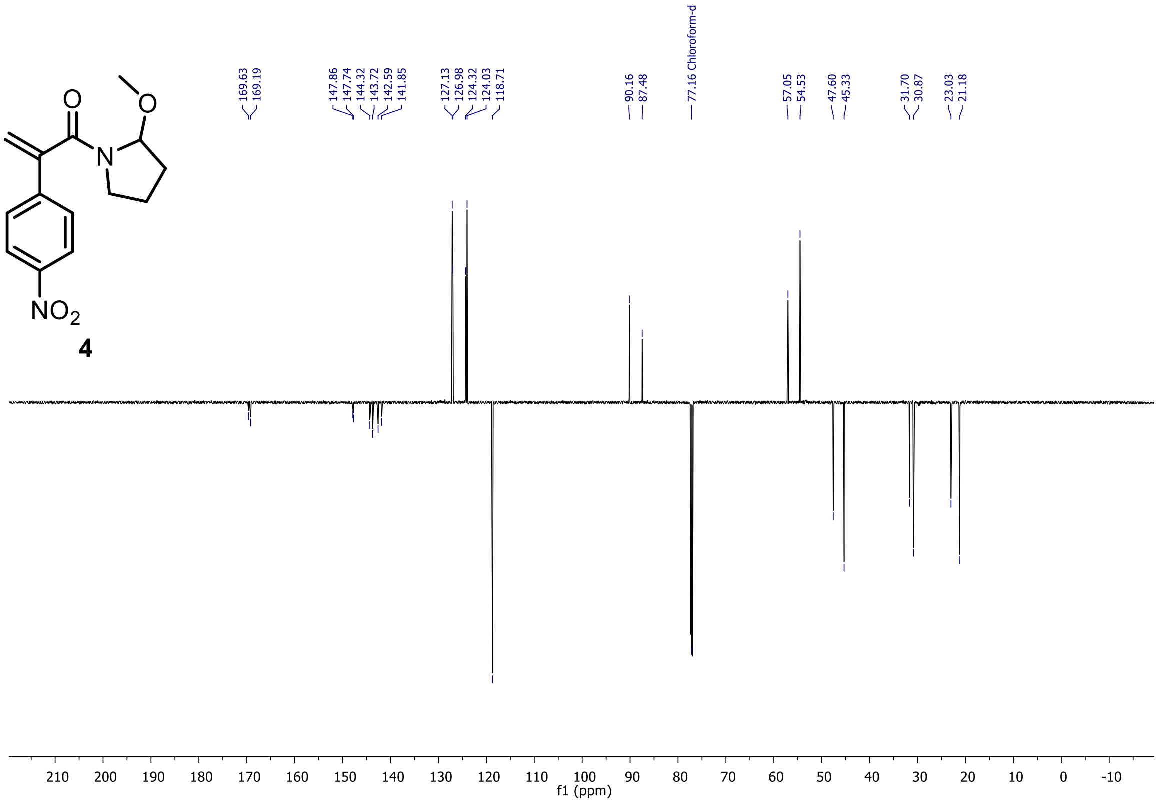

5: (*Z*)-*N*-5,5-Trimethyl-3-(4-nitrophenyl)furan-2(5*H*)-imine

^{1}H NMR (700 MHz, $CDCl_3$)

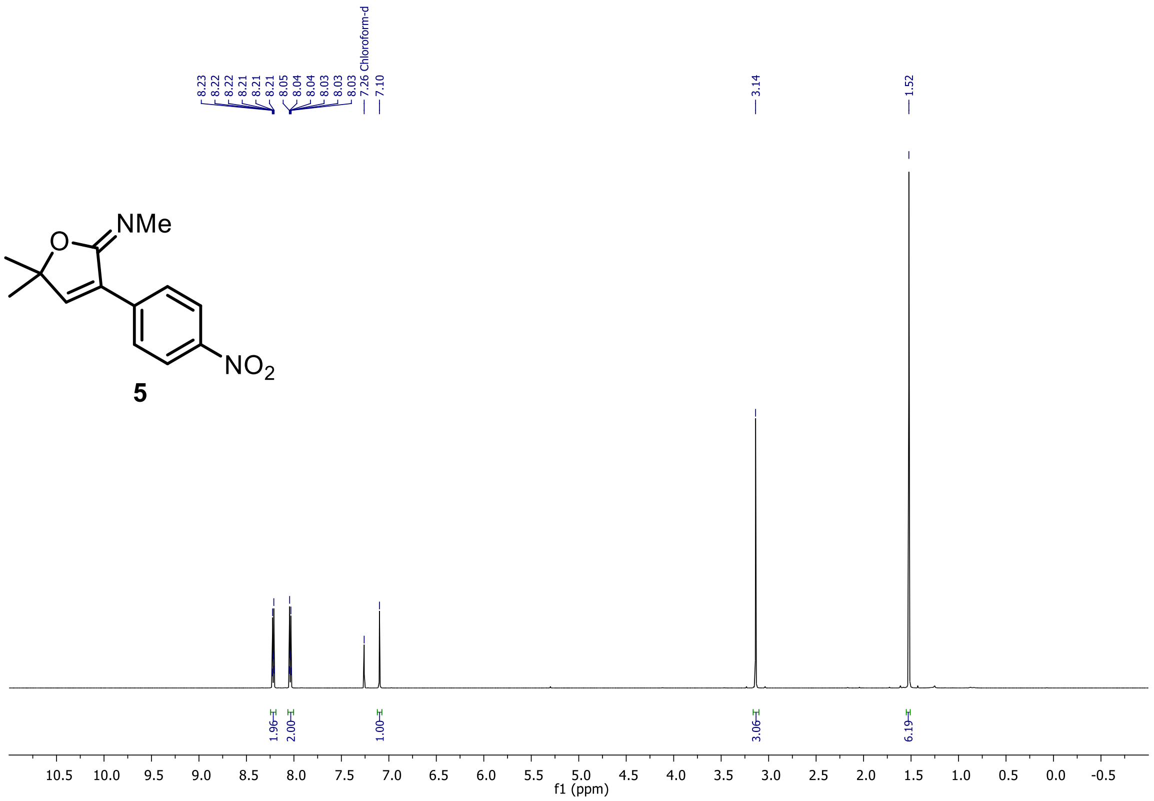

^{13}C NMR (175 MHz, $CDCl_3$)

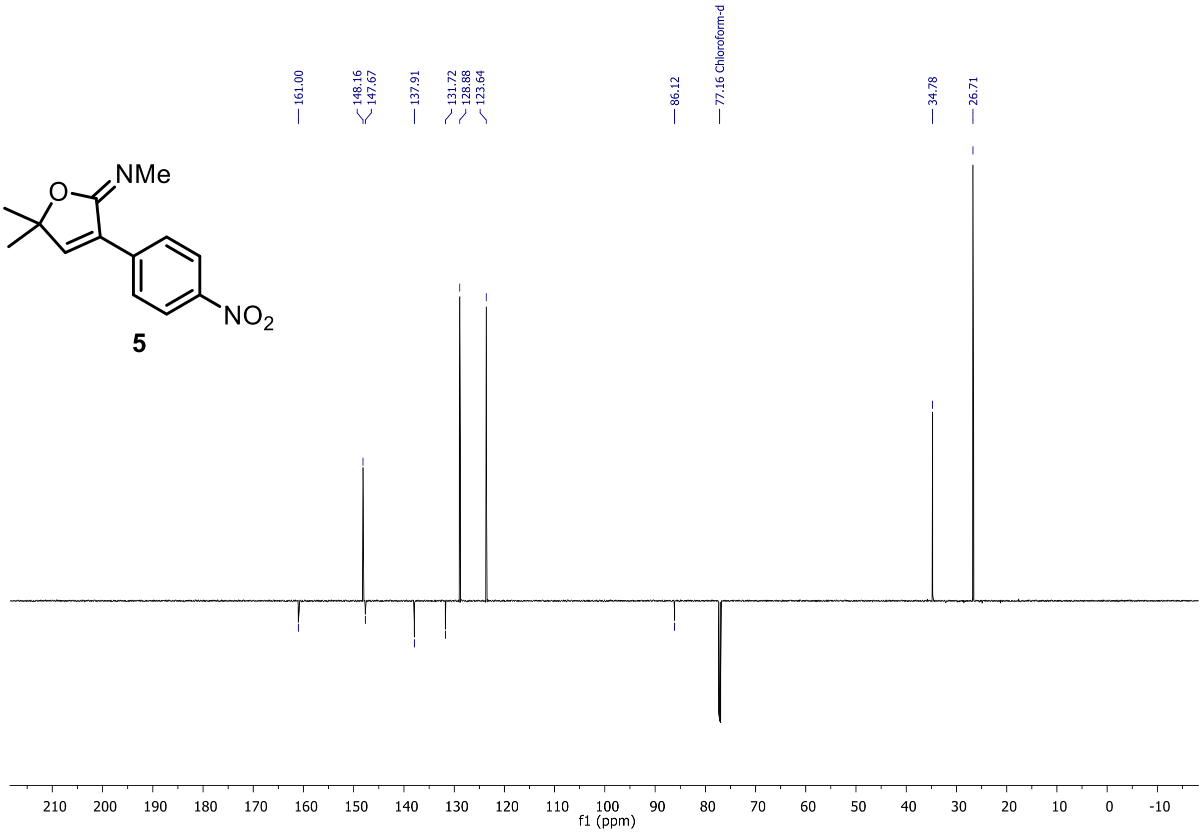

6: Ethyl-6-(4-nitrophenyl)-5-oxooctahydroindolizine-8-carboxylate

^{1}H NMR (600 MHz, $CDCl_3$)

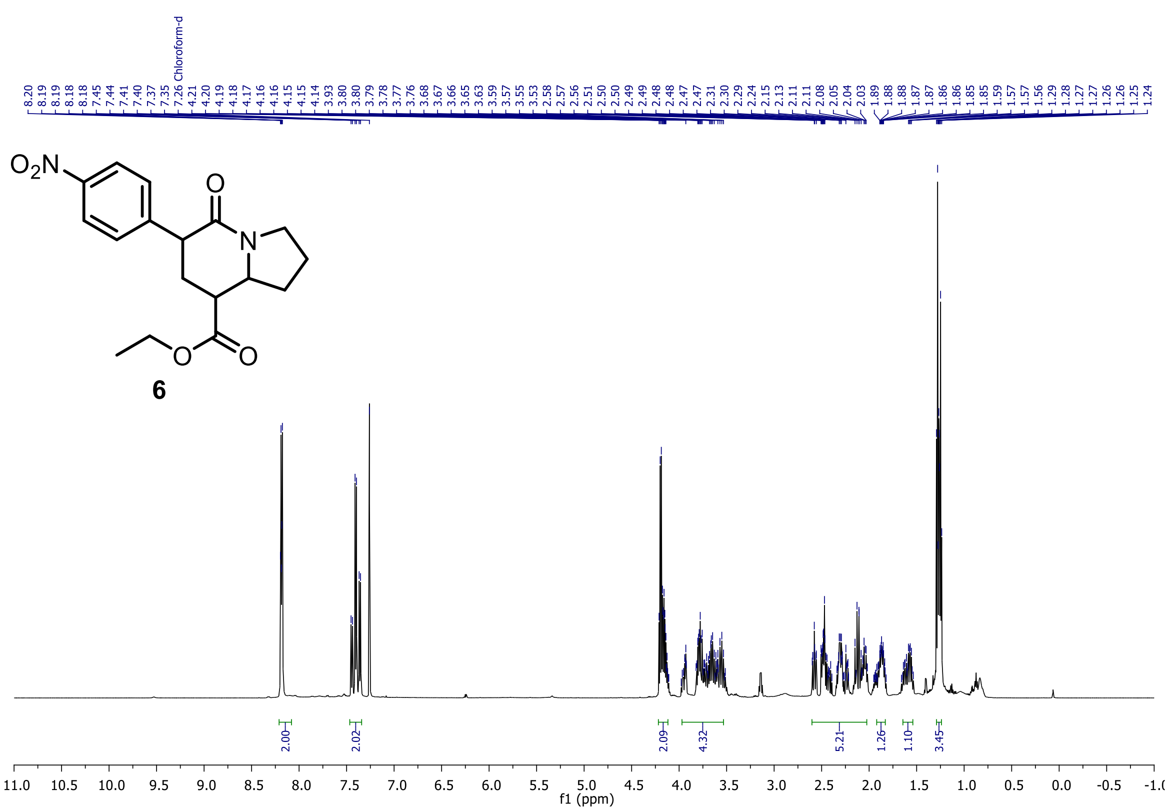

^{13}C NMR (150 MHz, $CDCl_3$)

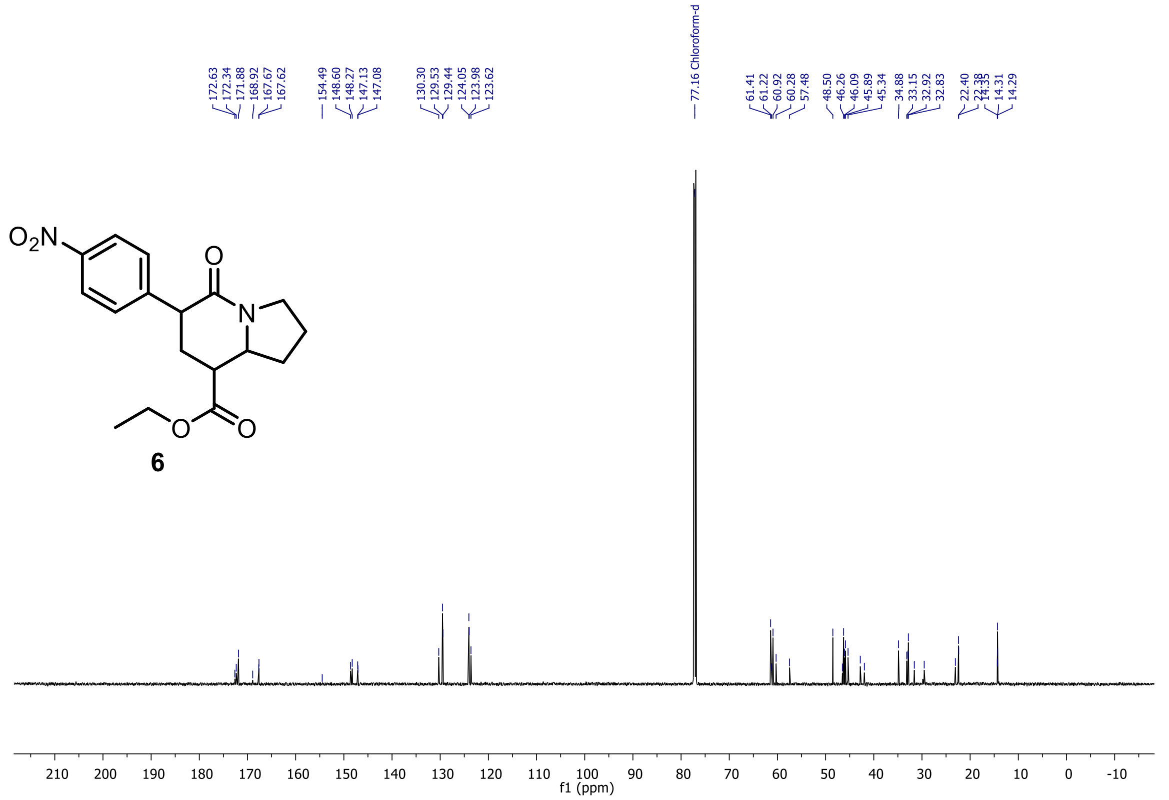

SI6: *N*-Methyl-*N*-((4-nitrophenyl)sulfonyl)propiolamide

^{1}H NMR (600 MHz, $CDCl_3$)

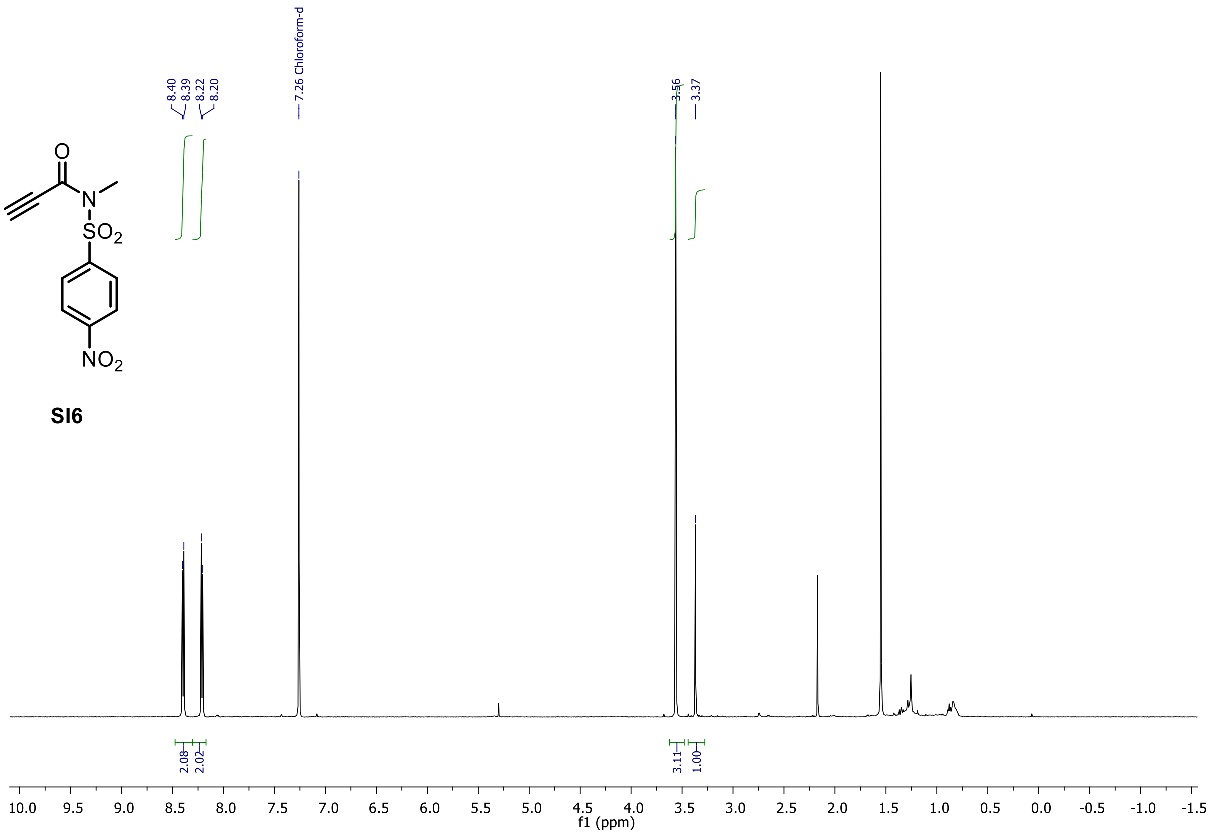

^{13}C NMR (150 MHz, $CDCl_3$)

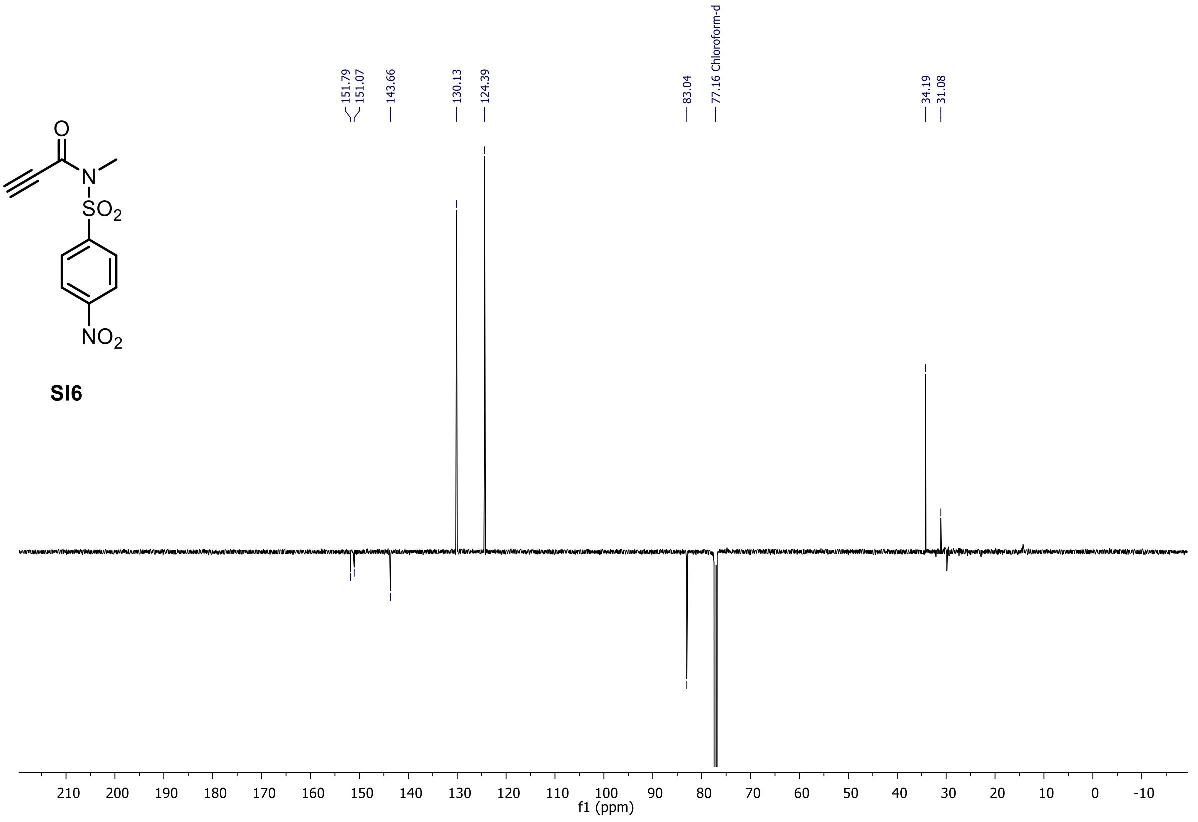

1a-*d*3: *N*-methyl-*N*-((4-nitrophenyl)sulfonyl)acrylamide-d3

^{1}H NMR (400 MHz, $CDCl_3$)

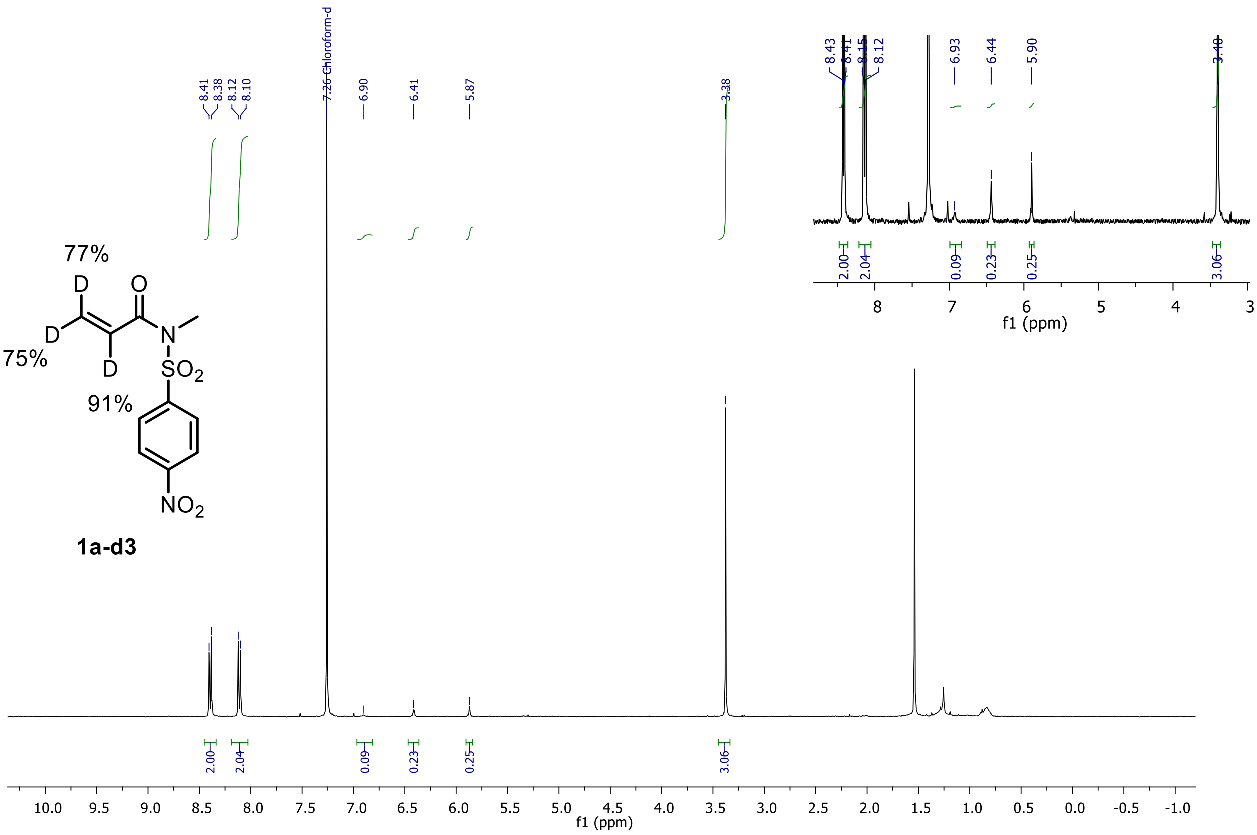

^{13}C NMR (150 MHz, $CDCl_3$)

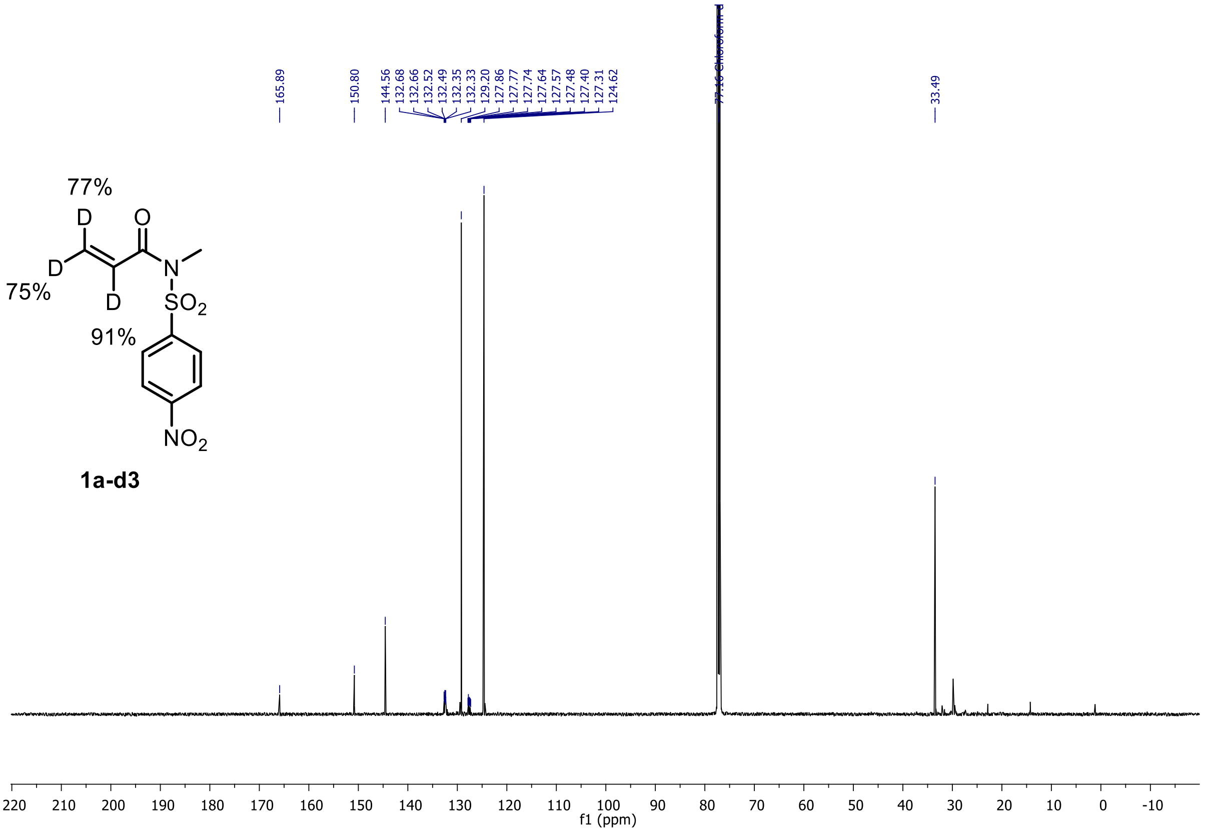

Supplement: Supplementary file 1 — Supporting Information [file ANGE-134-0-s001.pdf]
